# Using synthetic RNA to benchmark poly(A) length inference from direct RNA sequencing

**DOI:** 10.1093/gigascience/giaf098

**Published:** 2025-09-03

**Authors:** Jessie J -Y Chang, Xuan Yang, Haotian Teng, Jianshu Zhang, Benjamin Reames, Shuxin Zhang, Vincent Corbin, Lachlan J M Coin

**Affiliations:** Department of Microbiology and Immunology, University of Melbourne at The Peter Doherty Institute for Infection and Immunity, Melbourne, VIC, 3000, Australia; Department of Microbiology and Immunology, University of Melbourne at The Peter Doherty Institute for Infection and Immunity, Melbourne, VIC, 3000, Australia; Ray and Stephanie Lane Computational Biology Department, School of Computer Science, Carnegie Mellon University, Pittsburgh, PA 15213, United States; Department of Microbiology and Immunology, University of Melbourne at The Peter Doherty Institute for Infection and Immunity, Melbourne, VIC, 3000, Australia; Department of Microbiology and Immunology, University of Melbourne at The Peter Doherty Institute for Infection and Immunity, Melbourne, VIC, 3000, Australia; Department of Microbiology and Immunology, University of Melbourne at The Peter Doherty Institute for Infection and Immunity, Melbourne, VIC, 3000, Australia; Department of Microbiology and Immunology, University of Melbourne at The Peter Doherty Institute for Infection and Immunity, Melbourne, VIC, 3000, Australia; Department of Microbiology and Immunology, University of Melbourne at The Peter Doherty Institute for Infection and Immunity, Melbourne, VIC, 3000, Australia; Department of Clinical Pathology, University of Melbourne, Melbourne, VIC, 3000, Australia

**Keywords:** Oxford Nanopore Technologies, poly(A) tail, estimation, segmentation, direct RNA sequencing

## Abstract

Polyadenylation is a dynamic process that is important in cellular physiology, which has implications in messenger RNA decay rates, translation efficiency, and isoform-specific regulation. Oxford Nanopore Technologies direct RNA sequencing provides a strategy for sequencing the full-length RNA molecule and analysis of the transcriptome. Several tools are currently available for poly(A) tail length estimation, including well-established methods like *tailfindr* and *nanopolish*, as well as more recent deep learning models like *Dorado*. However, there has been limited benchmarking of the accuracy of these tools against gold-standard datasets. In this article, we present our novel deep learning poly(A) estimation tool—*BoostNano*—and compare with 3 existing tools—*tailfindr, nanopolish*, and *Dorado*. We evaluate the 4 poly(A) estimation tools, using 2 sets of synthetic *in vitro* transcribed RNA standards with known poly(A) tail lengths—Sequin (30 or 60 nucleotides) and enhanced green fluorescent protein (10–150 nucleotides) RNA. Analyzing datasets with known ground-truth values is a valuable approach to measuring the accuracy of poly(A) length estimation. The tools demonstrated length- and sample-dependent performance, and accuracy was enhanced by averaging over multiple reads via estimation of the peak of the density distribution. Overall, *Dorado* is recommended as the preferred approach due to its relatively fast runtimes, low mean error, and ease of use with integration with base-calling. These results provide a reference for poly(A) tail length estimation analysis, aiding in improving our understanding of the transcriptome and the relationship between poly(A) tail length and other transcriptional mechanisms, including transcript stability or quantification.

## Findings

### Background

Polyadenylation is a co-/post-transcriptional process in which a string of adenine nucleotides is added to the 3′ of nascent messenger RNA (mRNA) molecules by enzymes such as polyadenylate (poly(A)) polymerases (PAPs) [1–3]. In eukaryotes, the polyadenylation process begins through the recognition of the poly(A) signal (PAS) situated within the 3′ untranslated region (UTR) of the mRNA [4]. This is a 6 nt sequence motif—commonly “AAUAAA”, located approximately 10 to 30 nucleotides (nt) upstream of the poly(A) tail [5]. The polyadenylation process is mediated by the cleavage and specificity factor (CSF) complex, which is made up of 4 major subunits—specificity factor (SF), cleavage stimulation factor (CstF), and cleavage factors I and II (CFI and CFII). The SF recognizes the poly(A) signal and is required for specific cleavage and polyadenylation [6–10]. Additionally, CFI and CFII are required for accurate cleavage, and CstF enhances efficient cleavage at the poly(A) site, and for a proportion of cases, PAP is required [8, 11]. PAP extends the poly(A) tail, stimulated by CSF and poly(A) binding protein II (PABP II) within the nucleus [12, 13]. After the 5′ capping, splicing, and polyadenylation, the mRNA is exported out of the nucleus into the cytoplasm. Here, the poly(A) tail is regulated by various deadenylase complexes—including CCR4-NOT [14] and PAN2-PAN3 [15]. Traditionally, the eukaryotic non-mitochondrial mRNA poly(A) tail has been regarded to be on average ~150 to 200 nt [16], which is more associated with the initial polyadenylation stages in the nucleus. With the involvement of deadenylation in the cytoplasm, the steady state of poly(A) tails has been identified to be shorter (~50–100 nt) [17, 18]. It has also been noted that nonadenine bases can be found within poly(A) tails as well as internal poly(A) sites [19].

Polyadenylation is thought to increase the stability of the mRNA molecule [20], assist in the export of the molecule from the cell nucleus [21], and it plays a role in RNA circularization, which may enhance efficient translation of cellular mRNAs [22]. This process is increasingly recognized as a dynamic process [23] that influences the timing and degree of protein production [24, 25]. Furthermore, it is implicated in mRNA decay rates and regulation of gene expression [26, 27]. Poly(A) tails are also regarded to be dynamic in viral RNA, such as in the bovine coronavirus [23]. Currently, an ample number of studies have explored alternative polyadenylation (APA) [28–31]—the alternative usage of poly(A) sites, which leads to variable 3′ ends of transcripts derived from the same gene. However, this mechanism is commonly confused with the study of poly(A) tail lengths, and the latter is comparatively underexplored. As such, it is critical to be able to measure polyadenylation accurately using a high-throughput assay, which has the potential to enhance our understanding of the poly(A) tail length and its connections to other transcriptional and translational mechanisms.

Most existing literature on measuring the poly(A) length has utilized techniques such as PCR [23, 32], Northern blotting [33], or short-read poly(A) tail measurements such as PAL-seq [34] or TAIL-seq [17], which have clear limitations in terms of breadth of whole-transcriptome-wide detection, lengths, and arduous experimental efforts. In contrast, Oxford Nanopore Technologies (ONT) direct RNA sequencing is a simple approach for single-molecule RNA sequencing that does not require reverse transcription (other than for RNA stabilization and improving sequencing output) or PCR amplification, thus avoiding amplification bias and retaining the original base and base-modification information [35–39]. It is worth noting that the full-length cDNA synthesis step, while not required, is recommended, and the library preparation method utilizes a polythymine (poly(T))–containing adapter for sequencing. Furthermore, full-length RNA molecules can be captured in 1 read, facilitating the identification of complex splicing patterns, RNA modifications, and RNA secondary structures [40–45]. The Nanopore sequencer records changes in ionic current as RNA passes through the pore in a custom FAST5/POD5 file. These raw data are then converted into sequence data using a custom deep learning model, via the use of basecallers. Although the majority of currently available public datasets have been generated from the SQK-RNA002 Direct RNA Sequencing kit, an updated version was released via early access in November 2023 (SQK-RNA004), as well as direct RNA-specific flow cells (FLO-MIN004RA or FLO-PRO004RA). The improvements include a faster motor protein, an RNA-specific reader pore, enhanced RNA models in the *Dorado* basecaller, and an optimized library preparation method. Notably, recent iterations of *Dorado* have included the ability of RNA modification detection and poly(A) length estimations, which were only possible via third-party tools in previous years. Hence, Nanopore sequencing of native RNA provides an attractive approach for measuring single-molecule transcriptome-wide poly(A) tail length.

There have been several tools developed for estimating poly(A) tail length from the raw Nanopore signal (Table 1), including *nanopolish* [46], *tailfindr* [42], *Dorado* (developed by ONT) [47], and our in-house tool *BoostNano* (details described in Supplementary Information and Supplementary Figs. S1–S3; biotools:boostnano, RRID:SCR_026467) [48]. The tools detect the boundaries of poly(A) tails in varied ways. *tailfindr* identifies potential poly(A) stretches based on 2 rounds of defining the poly(A) tail segment, first determining rough poly(A) boundaries by thresholding the smoothened signal using a sliding window. Then, the second stage computes the mean of every 25 samples of clipped signal and shrinks the rough poly(A) boundaries via confining the raw signal slopes. *nanopolish* utilizes a hidden Markov model (HMM), where each region of the read—the sequencing adapter, RTA, poly(A) tail, and coding transcript—has 1 state contained by the HMM, in which these regions are linked sequentially, through linear-chain state transitions. Each section is deemed to have a unique emission distribution, which can be modeled by the HMM and applied on each read. *Dorado* utilizes a sliding window approach to find signal characteristics, initialized by identifying the RNA adapter sequence and determining the signal anchor point (i.e., the start of the poly(A) tail). The boundaries of the poly(A) tail are identified via analyzing around the proximity of the anchor point and understanding regions of the signal with low variance and similar mean values. *BoostNano* considers each region of the read as states like *nanopolish*. The neural network combines previous hidden states with current signal estimates to predict the signal’s state, performing segmentation (more details can be found in Supplementary Information). However, there have been limited attempts to benchmark poly(A) tail length inference using gold-standard datasets with known poly(A) tail lengths, as well as comparisons between the 2 most recent kit versions—RNA002 and RNA004.

**Table 1:** Summary of each poly(A) tail estimation tool benchmarked in this study

Tool	Description	Reference
*BoostNano*	Convolutional neural network (CNN)–recurrent neural network (RNN)–Connection-ist Temporal Classification (CTC) architecture from *Chiron* basecaller used to find boundaries of poly(A) in raw signal; basecalling not required	Teng et al., 2018. [48]
*tailfindr*	*R* tool, which uses the unaligned raw FAST5 data to estimate the poly(A) lengths via using the raw signal slope to refine the boundaries of potential poly(A) stretches and normalization with the read-specific nucleotide translocation rate; basecalling required for obtaining basecalled FAST5 with Events/Move table	Krause et al., 2019 [42]
*nanopolish*	Utilizes a predictive model in which a hidden Markov model (HMM—performs segmentation of the raw sequencing signal) and an estimator of the translocation rate are combined; basecalling required for obtaining input FASTQ	Simpson et al., 2017 [46]
*Dorado*	Searches for the boundaries in the raw signal and estimates the poly(A) tail length by considering the samples/base information, with adjustment for overestimation of the poly(A) tail; primarily a basecalling tool, incorporates the poly(A) tail estimation during the basecalling itself	Oxford Nanopore Technologies [47]

For this study, we utilize 2 classes of ground-truth datasets derived from (i) RNA Sequins—synthetic *in vitro–*transcribed (IVT) RNA, transcribed from an artificial chromosome that comprises 78 gene loci split into 2 classes, having either a 30-nucleotide (nt) (R1) or a 60 nt (R2) poly(A) tail (BioProject: PRJNA675370) [44, 49], and (ii) IVT RNA using enhanced green fluorescent protein (eGFP) constructs with a wider range of poly(A) lengths (10, 30, 40, 60, 100, and 150 nts) from the authors of *tailfindr* (ENA Project: PRJEB31806) [42]. Thus, in this study, we compare the commonly used poly(A) tail length estimation tools (*Dorado, tailfindr*, and *nanopolish*) along with our own novel tool—*BoostNano* (released for the first time via this Technical Note) in the hope of understanding and disseminating information to the wider community regarding the most appropriate tool for poly(A) length estimation.

### Performance evaluation among *BoostNano, tailfindr, nanopolish*, and *Dorado*

**Figure 1:**
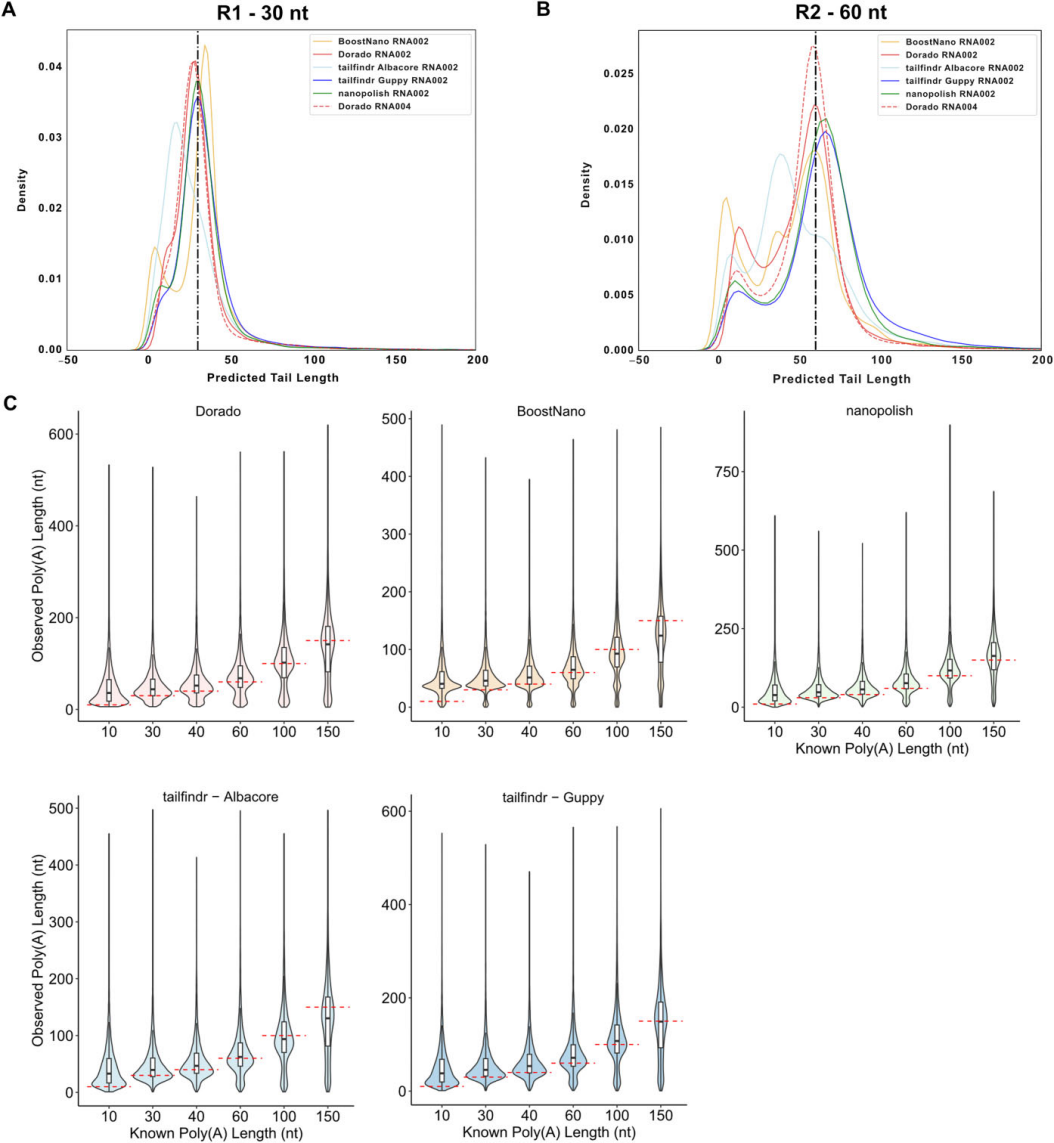
Poly(A) tail length estimates for each tool. (A, B) Predicted poly(A) tail length distributions for (A) R1 (30 nt) and (B) R2 (60 nt) Sequins. Outputs from *BoostNano* (yellow), *Dorado* (red), *tailfindr* (blue), and *nanopolish* (green). RNA002 (solid) and RNA004 (dashed) are shown as different line types. The x-axis shows the predicted poly(A) tail lengths of all reads, and the y-axis reveals the density of the poly(A) tail lengths. nt: nucleotides. Black vertical dashed lines indicate the known lengths. (C) RNA002 poly(A) tail estimates of eGFP synthetic RNA from the study by Krause et al. [42], ranging between 10 and 150 nt in poly(A) length. Red dashed lines indicate the corresponding known poly(A) lengths.

To compare the estimation performance of *BoostNano, tailfindr* v1.4, *nanopolish* v0.13.3, and *Dorado* v0.9.0, we tested these tools on 2 Sequin testing sets with known poly(A) tail lengths: R1 set with 30 nt tails and R2 set with 60 nt tails, as well as eGFP synthetic RNA with poly(A) tails (10–150 nt) (Fig. 1A–C and Supplementary Data S1) [49]. First, to estimate the accuracy of each method, we visualized the density distributions for each dataset (Fig. 1A–C). The 4 methods displayed a similar pattern in the density distribution, with a prominent normal-like peak near the expected poly(A) length, but also with an overrepresentation of shorter poly(A) tails, ranging from approximately ~0 to 20 nt (Fig. 1A, B). For R2 Sequins, we observed a clear multimodal distribution in all tools, with a trimodal distribution with *BoostNano* (Fig. 1B). In contrast, the R1 Sequin estimates presented with either a multimodal distribution (*nanopolish* and *BoostNano*) or a shoulder peak adjacent to the main peak, which was least evident in RNA004 *Dorado* data (Fig. 1A). In the eGFP datasets, the bimodal distributions appeared mostly in datasets with ≥40 nt poly(A) tails (Fig. 1C). *BoostNano* showed trimodal distributions in 60 and 80 nt datasets, and with an extreme overestimation with the 10 nt dataset. These results suggest that as poly(A) length increases, the distribution becomes more likely to be multimodal, making means and medians potentially misleading as measures of average poly(A) length.

**Figure 2:**
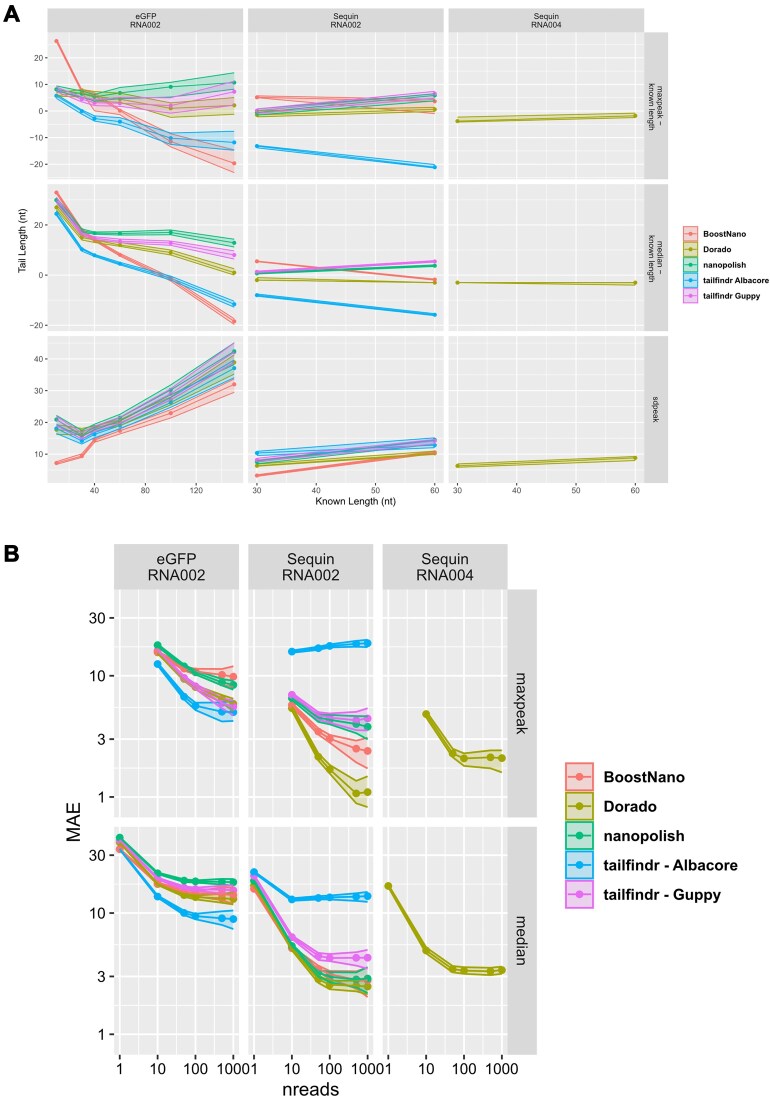
Tail length differences compared with known lengths and mean absolute error (MAE) per dataset and tool. (A) Tail length differences in nt of maxpeak and medians compared with the known length across the full range of known lengths. The x-axis represents the known length (10–150 nt for eGFP, 30 nt/60 nt for Sequins), and the y-axis represents the difference between estimated and known tail length (rows 1, 2) or the estimated standard deviation of the peak (row 3). The 95% confidence intervals are shown as ribbons. (B) Mean absolute error (MAE) of estimates of tail length across windows of nreads utilizing either maxpeak or median. The MAE is calculated as the average of the absolute difference to known lengths across all windows. The 95% confidence intervals are also shown in ribbon form. The x-axis represents the window size, and the y-axis represents the MAE.

We then attempted to obtain a single estimate of tail length from the distributions in Fig. 1 for each tool and each known tail length. We used 2 approaches. First, a simple median, which is understood to be robust to deviations from normality. However, given the multimodal nature of the distributions, we also estimated the value that maximized the probability density function, which we call “maxpeak” (Supplementary Data S1). We investigated the differences between these estimates and the known values (Fig. 2A), revealing a tendency for tools to overestimate short tails, particularly for the eGFP dataset. This analysis also revealed that the max-peak approach provided more accurate estimates than the median approach. *Dorado* showed less length-dependent errors than other methods, particularly combined with maxpeak estimation of tail length. We also observed that the correlation between methods increased as the number of reads included in the maxpeak statistic increased (Supplementary Figs. S4–S6). We estimated the standard deviation of the main peak of the probability distribution function by calculating its full width at half-maximum (Fig. 2A). As expected, this width increased as the known tail length increased. *BoostNano* was observed to have the tightest peak (meaning that more reads had values close to the maxpeak value).

Next, we investigated the accuracy of each tool at the read and grouped-read level. For read sets with 10 or more reads, we utilized either the median or maxpeak to generate an averaged estimate, as above. Then we calculated the mean absolute error (MAE) between the estimated and known lengths (Fig. 2B, Supplementary Fig. S7, Supplementary Data S2). We observed that averaging over reads can lead to substantial improvements in accuracy (plateauing at 100 reads), with maxpeak providing more accurate estimates than using the median. Out of curiosity, we estimated the poly(A) tails of the pre-basecalled eGFP FAST5 files pulled directly from ENA Project, which had been basecalled by the authors of *tailfindr* with *Albacore* v2.3.3. Upon comparison with the other tools, we noticed that this dataset revealed the lowest MAEs, despite being the oldest ONT basecaller tested (Fig. 2B). However, when we applied the same approach to Sequin datasets, the MAE was the highest out of all tools in the *Albacore*-basecalled dataset. Using bootstrap resampling, we calculated confidence intervals for each of the approaches (Fig. 2B). We also used this resampling procedure to calculate whether MAE differences between tools were statistically significant and found that most (but not all) differences are significant (*P* ≤ 4.11 × 10^–06^), as can be observed from the confidence intervals (Fig. 2B, Supplementary Data S3).

To further test each method’s ability to call poly(A) tails, we calculated the number of reads detected with the same number of input reads from the eGFP data, which were 592,571 reads (Supplementary Table S1). We found that *BoostNano* detected the greatest number of reads, and *nanopolish* detected the least number of reads in total, reads with poly(A) tails, and reads aligned to the eGFP barcodes (maximum 96,403 reads), highlighting the high sensitivity of *BoostNano*.

**Figure 3:**
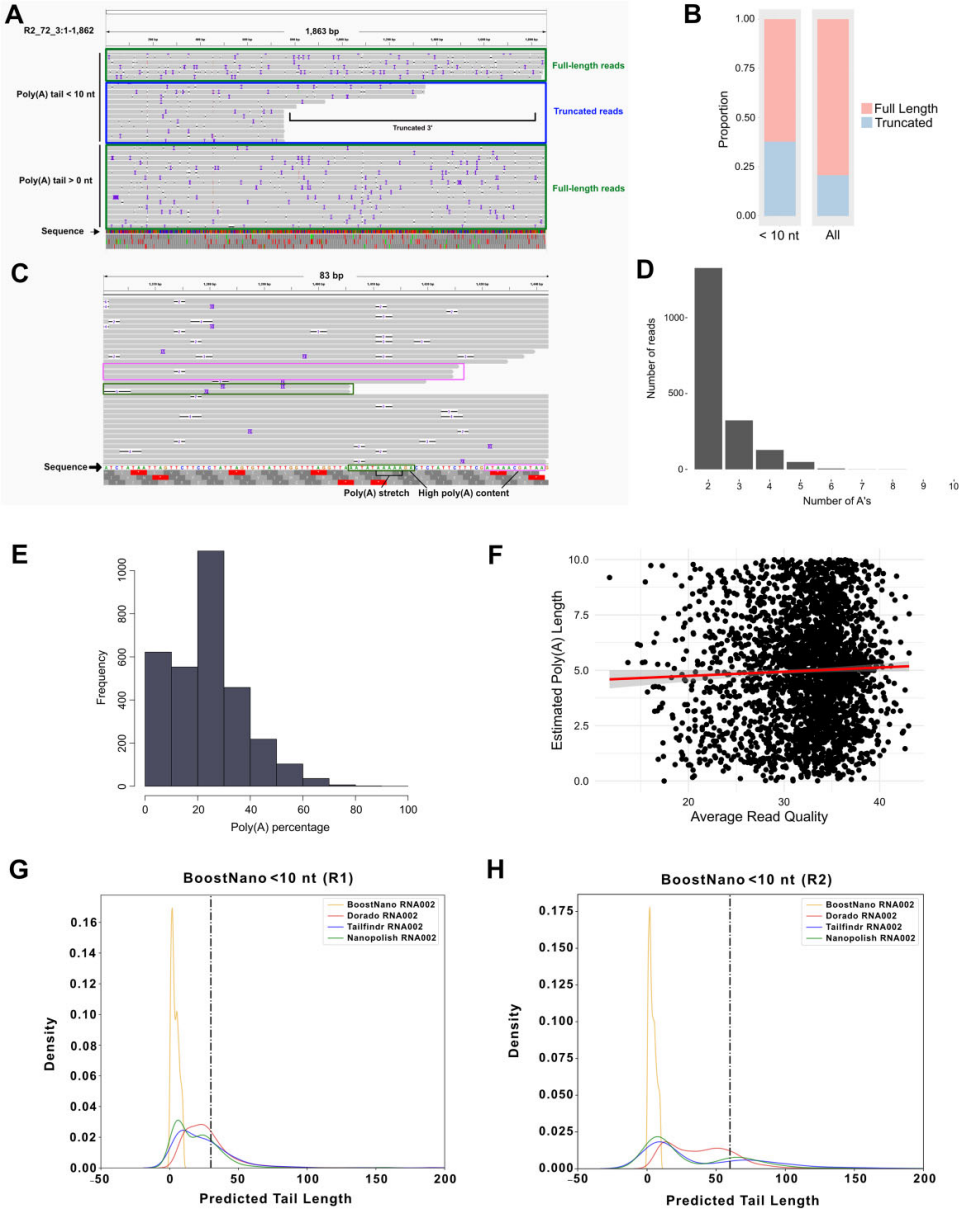
Poly(A) tails with short estimations <10 nt. (A) A representative subset of reads mapping to the R2_72_3 Sequin transcript visualized on the Integrative Genomics Viewer (IGV). The first subpanel shows a subset of reads with <10 nt poly(A) tails (estimated by *BoostNano*), showing that reads with <10 nt poly(A) tails are derived from both reads that have intact and fragmented 3′ ends. The second panel shows a representative subset of full-length reads in the full dataset with any detected poly(A) lengths >0 nt. Each gray line indicates a read. “Sequence” indicates the sequence of bases that form the transcript, where A = green, T = red, G = yellow, and C = blue. nt: nucleotides. (B) Proportion of reads with truncated vs. full-length 3′ ends in the entire combined Sequin RNA002 dataset and reads with poly(A) lengths <10 nt (estimated by *BoostNano*). (C) Truncated reads with poly(A) tails <10 nt (estimated by *BoostNano*) mapped to the R2_65_1 Sequin transcript and 3′ ends ending across an internal poly(A) stretch (green) and a stretch with high poly(A) content (pink). Each gray line indicates a read. “Sequence” indicates the sequence of bases that form the transcript, where A = green, T = red, G = yellow, and C = blue. (D) The number of adenines in the 10 nt stretch following the 3′ end of truncated reads with <10 nt poly(A) tails (estimated by *BoostNano*). (E) Percentage of adenines in the 10 nt stretch following the 3′ end of truncated reads with <10 nt poly(A) tails (estimated by *BoostNano*). (F) Average read quality scores vs. poly(A) lengths in truncated reads with <10 nt poly(A) tails (estimated by *BoostNano*), which do not meet thresholds of at least 4 adenines in a series or 60% poly(A) content in the 10 nt following the 3′ end of the mapped read. (G, H) Density plots of estimated read-lengths with <10 nt poly(A) tails (estimated by *BoostNano*) in all 4 tools in the Sequin (G) R1 and (H) R2 datasets.

We then proceeded to further understand the smaller peaks of the density distributions, which were present in almost all datasets (Fig. 1A–C). This peak was more prominent at ~0–5 nt in *BoostNano*, whereas the early peaks for *tailfindr, nanopolish*, and *Dorado* were positioned at ~5–20 nt. We hypothesized that these shorter peaks were derived from (i) fragmentation of the transcript, (ii) mispriming of internal poly(A) stretches, or (iii) degradation of the poly(A) tails. To test this, we inspected reads with <10 nt poly(A) tails (as measured by *BoostNano*) and observed that the majority (~62.2%) aligned within 20 nt of the 3′ end of the Sequin reference transcripts (Fig. 3A, B). This suggested that most of these shorter poly(A) tails occurred due to hypothetical reason (iii)—fragmentation/degradation of the poly(A) tail, which is more likely to be a sample integrity/preparation issue than an estimation defect. However, the remaining ~37.7% of reads showed truncations in the reference transcript (Fig. 3A), consistent with hypothetical reasons: (i) fragmentation of the physical RNA or (ii) mispriming. We wondered whether we could find any poly(A) stretches or high-adenine content in the sequences following the mapped 3′ end of the truncated Sequin reads, which would theoretically bind to the 10 poly(T)s of the reverse transcription adapter (RTA) in the Direct RNA Sequencing kit. A high rate of these endings would correlate to high rates of mispriming (Fig. 3C). To understand this phenomenon, we utilized the truncated dataset and isolated the 10 nt sequence following the end of the truncated Sequin reads according to the reference transcript. Then, we found the longest poly(A) stretch and the proportion of adenine bases in the 10 nt sequences. We observed that out of the truncated reads (3,088), only ~4.1%, ~1.6%, ~0.2%, ~0.03%, and 0.03% of the reads had a poly(A) stretch of at least 4, 5, 6, 7, and 8 adenines, respectively, with the longest poly(A) stretch being 8 adenines (Fig. 3D). Furthermore, only ~4.73% of the reads contained high (>50%) poly(A) content in the 10 nt downstream of their ends (Fig. 3E). Therefore, we were able to determine that mispriming was unlikely to be the main reason for the presence of these truncated transcripts with short poly(A) lengths. Finally, we isolated all reads that did not meet any of the criteria listed above (high poly(A) content/at least 4 adenine stretches in 10 nt downstream of mapped end and mapped within 20 nt of the 3′ end of reference Sequin transcript, ~93.8%) and examined their average read quality scores, as we thought this may contribute to the shorter poly(A) tail (Fig. 3F). We observed that surprisingly, most of the reads (~97.9%) showed average quality scores of >20, highlighting that poor read quality was not a prominent issue (Fig. 3F). The overall Spearman correlation between poly(A) lengths and average read quality scores showed 4 datasets (*Dorado* R2, *BoostNano* R1, *BoostNano* R2, *tailfindr* R1) with weak but positive correlations (*r* = 0.01–0.1, *P* < 0.05) and *nanopolish* R2 dataset showing a negative correlation (*r* = −0.04, *P* = 6.6e-08) (Supplementary Fig. S8). Therefore, we identified reads that were sequenced by direct RNA sequencing, which did not have proper poly(A) tails or mispriming events. Our analysis revealed that while all 4 poly(A) estimation methods consistently identified shorter poly(A) tails, *BoostNano* exhibited a narrower peak for these shorter tails, whereas *Dorado* tended to estimate longer poly(A) tails that were closer to the known values (Fig. 3G, H). Given that *tailfindr* and *nanopolish* also exhibited a peak at similar points in the density distributions as *BoostNano, Dorado* likely overestimates very short tails. Overall, the narrow peak of *BoostNano* indicates that *BoostNano* may not be suitable for estimating shorter poly(A) tails compared with the other tools.

**Figure 4:**
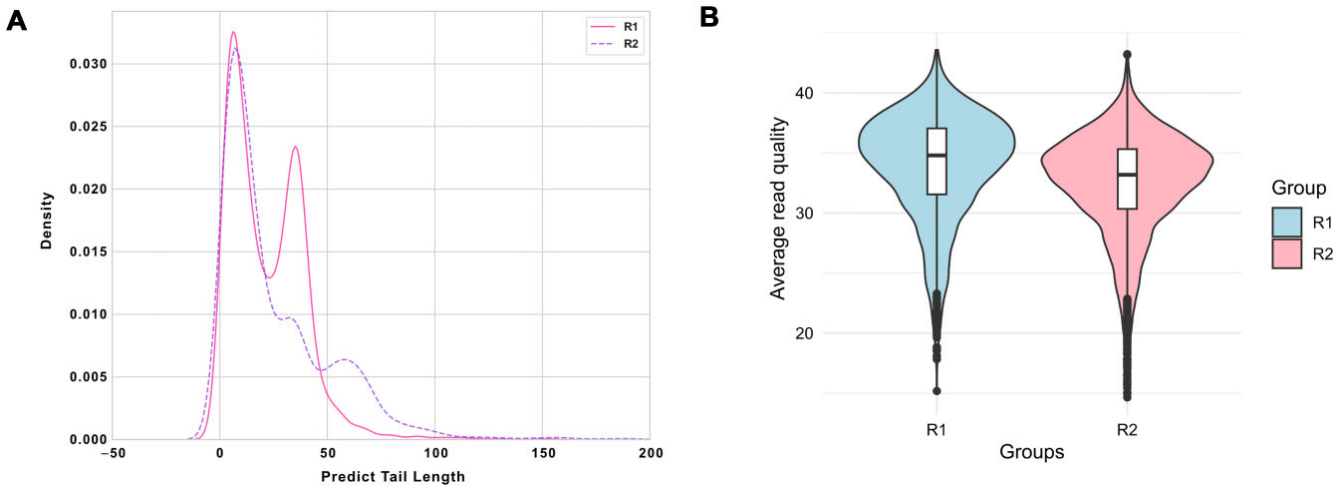
Reads that have been filtered out by *Dorado* but retained in the *BoostNano* output. (A) R1 Sequin reads are indicated in a solid pink line (known length = 30 nt), and R2 Sequin reads are indicated in a dashed purple line (known length = 60 nt). The x-axis shows the predicted poly(A) tail length in nucleotides, and the y-axis shows the density distribution. nt: nucleotide. (B) Violin plots of average read qualities of the same reads in R1 and R2 Sequin groups.

The explanations above partially explain the earlier peak (~0–20 nt) in the density distribution (Fig. 1) in all 4 methods, but *BoostNano* particularly showed the mode of the peak presenting at even shorter poly(A) tail lengths than *tailfindr, nanopolish*, and *Dorado*. As the ONT RTA used for reverse transcribing the native RNA strand has 10 poly(T) bases, it is likely that the minimum detection limit of poly(A) tails is 10 nt, which matches the ~10 nt peak with *tailfindr, nanopolish*, and *Dorado*. We hypothesized that poly(A) tails shorter than 10 nt may result from signal glitches during the signal detection, where one read may be written as multiple reads. Using *Bulkvis*, we discovered that among a total of 60,146 reads from all 7 RNA002 samples, 270 pairs of split reads were found (0.898%), with 26 pairs including 1 read in the list of reads with <10 nt poly(A) tails (Supplementary Data S4). This suggests that while read splits may partially explain the shorter poly(A) tails, other unexplained mechanisms are at play. Interestingly, upon investigating these earlier peaks, we found that *Dorado* excludes reads retained in the analysis by *BoostNano*, even though the majority of these reads were considered to have high read quality (Fig. 4A, B). While an earlier peak of <10 nt was the most prominent among reads discarded by *Dorado*, we also observed peaks of ~40 nt and ~60 nt (according to *BoostNano*) among reads discarded by *Dorado*. As mentioned above, *BoostNano* resulted in the greatest number of reads with detected poly(A) tails compared with the other 3 methods, including *Dorado* (Supplementary Table S1). Thus, *Dorado* demonstrates a more conservative approach compared to *BoostNano*.

Finally, we compared the computational time required by each method to predict the tail lengths of 4,000 reads. For *BoostNano* and *Dorado*, we used 1 graphics processing unit (GPU) with 16 G allocated RAM, while for *nanopolish* and *tailfindr*, which does not have the option to be run on a GPU, we used 1 central processing unit (CPU) with 16 G RAM and 1 thread for *nanopolish. Dorado* showed rapid computational times at just ~1 min 10 seconds, whereas *BoostNano* revealed the longest computational time at ~16 minutes 52 seconds (Table 2).

**Table 2:** Computational time efficiency to process 4,000 reads with 1 GPU/CPU. API: Application Programming Interface. Execution times include all preprocessing times such as basecalling and alignment.

Method	Execution time (4,000 reads, including basecalling, alignment)	GPU/CPU	GPU/CPU model	Processor	RAM	OS
*BoostNano*	16 min 52 s	1 GPU	80 GB A100 Nvidia GPU	Intel Xeon Gold	16 G	Linux
*Dorado*	1 min 10 s	1 GPU	80 GB A100 Nvidia GPU	Intel Xeon Gold	16 G	Linux
*tailfindr*	10 min 56 s	1 CPU	Intel Xeon Gold 6254 CPU @ 3.10 GHz	Intel Xeon Gold	16 G	Linux
*nanopolish*	2 min 5 s	1 CPU	Intel Xeon Gold 6254 CPU @ 3.10 GHz	Intel Xeon Gold	16 G	Linux

## Discussion

In this Technical Note, we assessed the predictive performance of 4 poly(A) tail length estimation tools—*tailfindr, nanopolish, BoostNano*, and *Dorado*—on 3 separate testing sets with known poly(A) tail lengths. This was explored via various methods, including manual visualization of density plots, calculating read-level and grouped read-level MAE, calculating standard deviation of the probability density function, and exploring the sensitivity of detection and execution time. When evaluating poly(A) lengths at the gene level or transcript level, the researcher may wish to utilize an average value to compare between different conditions. While medians are typically preferred over means due to the commonly skewed distribution in naturally occurring poly(A) lengths, our results highlighted that utilizing the maxpeak approach is more useful than the medians for read-sets with 10 reads or more (Fig. 2B and Supplementary Fig. S4). When using this method, we noted length- and sample-dependent rankings for the evaluated tools (Fig. 2A, Supplementary Data S1). Overall, *tailfindr* and *Dorado* were most accurate across the different lengths, and *BoostNano* performed poorest, with the exception of 60 nt tails, where *BoostNano* and *Dorado* performed well (Supplementary Data S1). Length-dependency of error rates is important to consider as polyadenylation research pertains mostly to mammalian (e.g., human) mRNA and, more recently, with viral RNA (e.g., Severe Acute Respiratory Syndrome Coronavirus 2 [SARS-CoV-2]). In mammals, poly(A) tails on mRNA undergo an initial synthesis stage, which increases to 100–200 nt, and once localized out of the nucleus, the poly(A) tail is subject to a deadenylation by CCR2-NOT and PAN2-PAN3 deandenylase complexes, in which the steady-state average poly(A) length is approximately ~50–100 nt [17]. The exceptions are in mitochondrial RNA, which have average poly(A) lengths of ~40–50 nt [50]. Furthermore, poly(A) lengths on SARS-CoV-2 RNA have been found to be approximately 45–60 nt [40, 44]. The implications of poly(A) estimation outputs can lead to different varied interpretations. If a method overestimates poly(A) tails, the researcher may overestimate other associated functions such as RNA stability, since longer poly(A) tails are commonly associated with greater stability. This is particularly detrimental when exploring differential polyadenylation, as even small changes in poly(A) tails may influence statistical tests. Thus, in general, we recommend the following: should the researcher have specific expected tail lengths for their study, they should choose the most appropriate tool based on the results of this study or similar, with the use of maxpeaks to average over read sets (*N* > 100). In more complex transcriptomes with wider variety of tail lengths, *tailfindr* or *Dorado* should be utilized.

Another factor that may be important to the researcher is time of execution. We observed that *Dorado* and *nanopolish* surpass the rate of *BoostNano* or *tailfindr* (Table 2). *BoostNano* and *tailfindr* tools provide estimation of the starting and ending positions of the poly(A) tails in event space. In contrast, *nanopolish* shows only the start positions, and this information is available from *Dorado* verbose logs. *nanopolish* and *tailfindr* require additional processing such as FAST5 basecalling and mapping, which also contribute to greater overall execution times. In light of these findings, we anticipate the adoption of *Dorado* as the default method for poly(A) tail estimation, given its rapid estimation time frame, comparable accuracy to other tools, conservative nature, and ease of integration with basecalling. One thing to note is that the community has noted remarkable differences in the performance of poly(A) tail measurements depending on the version of *Dorado* utilized, especially with versions prior to v0.5.3 having caused notable issues. Regardless, the current state of *Dorado* has favorable properties for the general user. We note that while *Dorado* is identified as the preferred tool, the researcher may prefer accuracy over time, especially in the case of low-throughput datasets. In this case, other methods like *tailfindr* may be implemented according to specific contexts.

The density distributions for the poly(A) lengths were clearly multinomial for most datasets, and this was more pronounced in longer poly(A) tail datasets (Fig. 1A–C). This phenomenon can be explained by the fact that there is a greater possibility for fragmentation for longer poly(A) tails, which can cause shorter than expected tail length peaks. This has also been noted in the *tailfindr* publication, although the commentary was referencing the cDNA data, instead of the RNA data [42]. Second, the lower peaks were particularly minimal in *Dorado* Sequin R1 datasets, which may be explained by its conservative nature in filtering out certain reads that were retained in other tools (Fig. 4A). *Dorado*’s conservative nature might be due to the tendency of the tool to base its estimation on searching for a low-variability region near an anchor point, and if such regions are undefined, this may lead to an omission of the reads. In contrast, as *BoostNano* detected the greatest number of poly(A) tails compared to all tools, we may attribute its unusual trimodal distribution to this reason. Early peaks in the poly(A) length density distribution (defined as <10 nt as measured by *BoostNano*) comprised ~68% of reads potentially affected by fragmentation/degradation of the poly(A) tail, and approximately one-third of these reads were deemed truncated and could not be accounted for by fragmentation/degradation of the poly(A) tail (Fig. 3B). Upon further investigation, ~94% of these unaccounted reads were not attributed to mispriming due to poly(A) stretches or poly(A)-rich regions in the 10 nt downstream of the 3′ end of the read (Fig. 3D, E). Incorrect segmentation was also only attributed to 26 read-pairs (Supplementary Data S4). It is currently unclear to us how these reads were able to be sequenced if the reads supposedly lacked a poly(A) tail due to truncation in the middle of the read. If truncations did occur, the 5′ end of the transcript, as opposed to the 3′ end, would be lacking. We do not believe this is due to mapping issues as reads with high-quality mappings were isolated, and we utilized synthetic RNA with well-defined references. Although seemingly unlikely, these types of reads may only be sequenced if somehow the reads were able to enter the pores without the RTA or sequencing adapters. Therefore, we suspect potential sequencing errors or incorrect adapter ligation. Further work will be required to elucidate this phenomenon, but nevertheless, this emphasizes the importance of the integrity of the input RNA.

Our study emphasizes the importance of obtaining sufficient coverage of each transcript in order to take advantage of improved poly(A) tail length estimation accuracy via averaging. We recommend obtaining at least 100× coverage of each target transcript to acquire a reliable estimate of poly(A) tail length via the maxpeak approach.

One of the limitations of this study is that we have only utilized synthetic RNA, for the purposes of procuring a ground truth. Researchers will aim to use poly(A) estimation tools for mainly real samples with more complex transcriptomes, including varying GC content, transcript and poly(A) lengths, modified bases, and coding potential unlike the synthetic RNA we explored in this study. From our understanding from previous studies regarding poly(A) lengths, we hypothesize that the poly(A) tail distribution will vary depending on these different factors. The results we reveal in this study help to understand biases observed via the use of different tools, in a controlled setting, without the effect of these variations. While this does not fully encapsulate the variability that may be seen with real samples, we hope that these data are useful for extrapolating which tool may suit the researcher best for their samples. Furthermore, generating a reliable ground-truth dataset for real samples across the transcriptome is exceedingly difficult, if not unattainable. Second, we have employed a combined dataset of 7 separate sequencing runs containing Sequins as well as 1 eGFP dataset for RNA002, and 6 sequencing runs for RNA004, with a lack of sequencing replicates. Potential batch effects may arise when visualizing complex transcriptomes with 1 sequencing replicate, which is common in many ONT direct RNA sequencing studies. Thus, this study may be extended via the use of such replicates. Our study is restricted to synthetic RNA, which have limited poly(A) length of 10–150 nt for RNA002 data, and 30 and 60 nt for RNA004 data. While the range in the RNA004 data may be shorter than biologically relevant for mammalian nonmitochondrial RNA, it fits the expected lengths for mitochondrial, viral, and plant RNA poly(A) tails [40, 44, 51, 52]. Overall, future work would benefit from expanding the range of poly(A) lengths to better mimic the distribution in real samples via synthetic and whole transcriptome data, gaining an enhanced understanding of length-specific biases in each tool and including RNA from diverse preparation methods.

In conclusion, this work demonstrates the value of synthetic RNA molecules with known poly(A) tail lengths for validating poly(A) tail estimation algorithms. As methods improve, we anticipate that these datasets will be valuable for assessing advancements in poly(A) tail estimation. *Dorado* proves to be highly efficient and accurate among the 4 tools we explored in this study. Thus, we recommend the use of this approach when performing poly(A) length analyses via implementing the maxpeak values and window averaging strategy.

## Methods

### Datasets

#### RNA002

This study utilized publicly available ONT direct RNA sequencing datasets involving SARS-CoV-2–infected continuous cell lines (Vero, Calu-3, and Caco-2) derived from our previous study, with synthetic RNA—Sequins (BioProject: PRJNA675370) [44]. Briefly, Vero (African green monkey kidney epithelia), Calu-3 (human lung adenocarcinoma epithelia), and Caco-2 (human colorectal adenocarcinoma epithelia) cells were cultured in 6-well tissue culture plates at 37°C, 5% (v/v) CO_2_. The Australian ancestral strain of SARS-CoV-2 (SARS-CoV-2/human/AUS/VIC01/2020) was used to infect these cells at a multiplicity of infection (MOI) of 0.1, and the cells were harvested at 0, 2, 24, and 48 hours post-infection (hpi). The total RNA was extracted, treated with DNAse using the Turbo DNA-free Kit (Invitrogen), and purified using the RNAClean XP magnetic beads (Beckman Coulter). Then, 6 μg total RNA for Vero cells and 3 μg total RNA for Calu-3 and Caco-2 cells were pooled, and 10% of expected mRNA (5% of total RNA) of Sequins [49] was added to each sample pool. The RNA was sequenced using the ONT Direct RNA Sequencing kit (SQK-RNA002), on R9.4.1 flow cells via the ONT MinION/GridION. For the purposes of this study, infected datasets from 24 and 48 hpi from all 3 cell lines and additionally 2 hpi from Vero cells were analyzed.

#### RNA004

Calu-3 cells were grown in 6-well tissue culture plates until 80–90% confluency and infected with 3× Australian strains of Delta, Omicron (XBB1.5), and Omicron (JN.1) SARS-CoV-2 virus in triplicate. The infected cells were incubated at 37°C, 5% (v/v) CO_2_, and harvested at 4 days post-infection (dpi). The total RNA was extracted using the RNeasy Mini Kit (Qiagen), treated with the Turbo DNA-free Kit (Invitrogen), and purified using RNAClean XP beads (Beckman Coulter). For the sequencing, 1 μg of final total RNA product + 5% of expected mRNA (5% of total RNA) of Sequin mixA were used as inputs. The 6× samples in total were sequenced—2× Delta, 3× XBB1.5, and 1× JN1 samples. The new ONT Direct RNA Sequencing kit (SQK-RNA004) was used to sequence the libraries with the following modifications to the reverse transcription step: use of the Induro Reverse Transcriptase (New England Biolabs) and incubation at 20 minutes at 55°C, then 10 minutes at 70°C. The libraries were sequenced using the kit-specific flow cells (FLO-MIN004RA, ONT) and the ONT MinION/GridION via *MinKNOW* v24.02.16 and live-basecalled using the *MinKNOW*-integrated version of *Dorado* v7.3.11. The Sequin reads can be accessed from the Data Availability section.

### Analysis

#### Basecalling and mapping

The minimum requirement for poly(A) estimation was FAST5 files. First, *Dorado* v0.9.0, which is a basecaller itself, and *BoostNano*, based on the *Chiron* basecaller, utilized raw FAST5 files as inputs. *Dorado* poly(A) estimation was carried out during the basecalling with the “–estimate-poly-a” parameter. For *nanopolish* analyses, raw FAST5 files were used for the “index” step, and *Dorado* v0.9.0 was used to generate the FASTQ files for the poly(A) estimation step. *tailfindr* requires basecalled FAST5 files for analyses with basecall group information in the FAST5. *Guppy* v6.3.2 was utilized for generating the basecalled FAST5 files. *Dorado* basecalling was incompatible with *taifindr* analyses as basecalled FAST5 files were required for the analyses. We attempted to test *tailfindr* using FAST5 files converted from POD5 files, but this generated empty results. All *Dorado* or *Guppy* basecalling was carried out via the “rna002 70bps_hac@v3” model for the RNA002 datasets and “rna004_130bps_sup@v5.1.0” model for the RNA004 datasets. No sup basecalling was available for RNA002.

For RNA002 data, an initial isolation of Sequins reads was carried out by using the live-basecalled FASTQ files merged (passed + failed) and mapped to the merged Ensembl GrCh38 human, SARS-CoV-2 (VIC01/Australia), and Sequin genomes using *minimap2* v2.26 via the parameters “-ax splice -un.” Using the read IDs, FAST5 files were isolated. For both RNA002 and RNA004 data, Sequins (R1/R2) were assigned to the reads by basecalling the FAST5 data with *Dorado* v0.9.0 and mapping the resulting FASTQ to the Sequin transcriptome with *minimap2* v2.26 with the parameters “-ax map-ont”. The reads were filtered with *Samtools* v1.16.1 “view” function with the parameters “-h -F 2308 -q 20.” The Sequin POD5/FAST5 files were isolated based on this mapping. The isolated Sequin FAST5 datasets can be accessed from the Data Availability section. The data were also additionally basecalled using *Albacore* v2.3.3, the first-generation ONT basecaller. Interestingly, *Albacore* v2.3.3 did not have an RNA002-specific config file, so we utilized the RNA001 + FLO-MIN106 combination: r941_70bps_rna_linear.cfg in attempts to replicate the methods of the *tailfindr* publication.

For further analysis, we implemented IVT synthetic RNA002 datasets generated from eGFPs from the *tailfindr* publication, which is publicly available (ENA Project: PRJEB31806). FAST5 files that were downloaded directly were basecalled via *Albacore* v2.3.3 by the *tailfindr* authors. We subsequently re-basecalled the data with *Dorado* v0.9.0 as well as *Guppy* v6.3.2 and carried out the rest of the analysis as per the Sequin analysis. For *nanopolish* analysis, the data were mapped to the pCS2 + eGFP genome from Addgene, using *minimap2* v2.26 with the parameters “-ax map-ont”. The reads downloaded were demultiplexed into different poly(A) tail lengths (10, 30, 40, 60, 100, 150 nt) by using the *seqkit* v2.5.1 “grep” function with parameters “-s –p $barcode –R 1:120 –m 1” by searching for corresponding barcode sequences in the first 120 bp of the reads (1 mismatching was allowed). Read IDs for demultiplexing can be accessed from the Data Availability section.

#### Poly(A) tail length analysis

For poly(A) tail length estimations, *Dorado* v0.9.0, *BoostNano, tailfindr* v1.4, and *nanopolish* v0.13.3 were used with the parameters outlined in Table 3 and can be accessed from the Data Availability section.

**Table 3:** Parameters for analysis

Tool	Version	Parameters	Default (Y/N)
*Dorado*	0.9.0	Model: rna004_130bps_sup@v5.1.0 for R004 rna002_70bps_hac@v3 for R002 --estimate-poly-a Default: --poly-a-config	Y
*BoostNano*	N/A	-i $1 -o $2 -m path/to/model --replace	Y
*Tailfindr*	1.4	find_tails(fast5_dir = args[1], save_dir = args[2], csv_filename = "tails.csv", num_cores = 30, basecall_group=args[4])	Y
*Nanopolish*	0.13.3	--reads=${FASTQ} --bam=${BAM} --genome=${REF} --threads=8	Y

Poly(A) truncations were visualized with the Integrative Genomics Viewer (IGV) v2.10.1 using data with <10 nt poly(A) tails according to *BoostNano* and all mapped data. All poly(A) tail truncation investigations were carried out in *R* v4.4.0 and density plots using *Python* v3.10.4.

#### Execution time calculations

Method timings were carried out using a subset of 4,000 reads, derived from Vero 2 hpi datasets, which were used for all timings. Basecalling and poly(A) tail times were added for the overall execution time. For *tailfindr* and *nanopolish*, which requires basecalled FAST5 files and FASTQ files, respectively, *Guppy* v6.3.2 was utilized.

#### Average poly(A) tail length calculations

Maxpeak was calculated as the value at which the probability density distribution achieves its maximum value. The density function was estimated using the density function from the stats package in *R*. Maxpeak and medians were calculated using only reads that were able to be detected by all methods to ensure direct comparisons.

The MAE was calculated by calculating either the median or maxpeak of reads in each nonoverlapping window of *N* reads and finding the absolute difference between this median and the ground-truth value. Then, the sum of these absolute values was divided by the number of windows. Only reads that were able to be detected by all tools were retained in the analysis. Bootstrapped *t*-tests were implemented to compare the accuracy of each tool and were carried out by resampling reads 1,000 times from the original dataset at random within each known length category (with replacement). Due to this finite number of resamples, the smallest possible nonzero *P* value is 0.001. Therefore, any reported *P* value of 0 should be interpreted as *P* < 0.001, indicating that the observed effect was consistent across all bootstrap iterations.

#### PolyA tail length effect by split reads

To detect split reads in the Sequin RNA002 reads, we first aligned Sequin reads from 7 samples from different cell types and time points (Caco [24, 48 hpi], Calu [24, 48 hpi], Vero [2, 24, 48 hpi]) to the Sequin reference genome using *Minimap2* v.2.24 with parameters “-x splice -uf -k14.” Then we used the *Dorado* v0.9.0 “summary” function to generate the basecalling summary files from ubams. Alignment report and basecalling summaries were input to *Bulkvis* (v2.0.1) [53] to find incorrectly split reads.

## Availability of Supporting Source Code and Requirements

Project name: BoostNano

Project homepage: https://github.com/haotianteng/BoostNano

Operating system(s): Platform independent

Programming language: Python

Other requirements: Pytorch

License: Mozilla Public License, v. 2.0

Biotools: boostnano

RRID:SCR_026467

## Supplementary Material

giaf098_Supplemental_Files

giaf098_Authors_Response_To_Reviewer_Comments_original_submission

giaf098_Authors_Response_To_Reviewer_Comments_Revision_1

giaf098_GIGA-D-24-00432_Original_Submission

giaf098_GIGA-D-24-00432_Revision_1

giaf098_GIGA-D-24-00432_Revision_2

giaf098_Reviewer_1_Report_Original_Submission Christoph Dieterich -- 12/20/2024

giaf098_Reviewer_1_Report_Revision_1 Christoph Dieterich -- 5/27/2025

giaf098_Reviewer_2_Report_Original_Submission Jesse Daniel Brown -- 1/7/2025

giaf098_Reviewer_3_Report_Original_Submission Paulo Gattai -- 1/7/2025

giaf098_Reviewer_3_Report_Revision_1 Paulo Gattai -- 5/6/2025

## Data Availability

The datasets supporting the results of this article are available in the NCBI repository, RNA002–BioProject: PRJNA675370. All additional supporting data are available in the *GigaScience* repository, GigaDB [54].

## Additional Files

**Supplementary Information:** Supplementary Information, Supplementary Figs. S1–S8, and Supplementary Table S1.

**Supplementary Data S1**. General table of statistical metrics, including medians, maxpeak, sdpeak, confidence intervals, and number of reads detected.

**Supplementary Data S2**. MAE results based on medians or maxpeaks for Sequin (RNA002, RNA004) and eGFP datasets per tool, averaged over window sizes *N* = 1, 10, 50, 100, 500, and 1,000.

**Supplementary Data S3**. Bootstrapped *t*-test results comparing MAE of each tool averaged over window sizes *N* = 1, 10, 50, 100, 500, and 1,000.

**Supplementary Data S4**. RNA002 read pairs split during incorrect segmentation detected by *Bulkvis*. Sequin reads from all RNA002 datasets were utilized—Caco (24, 48 hpi), Calu (24, 48 hpi), and Vero (2, 24, 48 hpi).

**Supplementary Fig. S1**. An output example of the segmentation plot using *BoostNano*. The first red line indicates the start of the adapter, the second line indicates the position of the start of the poly(A) tail, and the third line indicates the position of the end of the poly(A) tail. The y-axis is the electric current (picoAmperes [pA]), and the x-axis is the reverse of the sampling rate (1/4,000 seconds), as Nanopore sequencing samples at 4,000 events per second.

**Supplementary Fig. S2**. General model structure of *BoostNano*.

**Supplementary Fig. S3**. The summary of the linear regression model.

**Supplementary Fig. S4**. Correlation plots of each tool in the RNA002 Sequin dataset with averaging over windows with 1, 10, 50, and 100 reads via the maxpeak method. Both x- and y-axes indicate the poly(A) length. The figures indicate the MAE derived from all: all reads, 30: R1 reads, and 60: R2 reads. The figure reveals that the correlation improves as the number of reads averaged increases.

**Supplementary Fig. S5**. Correlation plots of *Dorado* in the RNA004 Sequin dataset with averaging over windows with 1, 10, 50, and 100 reads via the maxpeak method. Both x- and y-axes indicate the poly(A) length. The figures indicate the MAE derived from all: all reads, 30: R1 reads, and 60: R2 reads. The figure reveals that the correlation improves as the number of reads averaged increases.

**Supplementary Fig. S6**. Correlation plots of each tool in the RNA002 eGFP dataset with averaging over windows with 1, 10, 50, and 100 reads via the maxpeak method. Both x- and y-axes indicate the poly(A) length. The figures indicate the MAE derived from all: all reads, 10: reads with known 10 nt poly(A) tail, 30: reads with known 30 nt poly(A) tail, 40: reads with known 40 nt poly(A) tail, 60: reads with known 60 nt poly(A) tail, 100: reads with known 100 nt poly(A) tail, and 150: reads with known 150 nt poly(A) tail. The figure reveals that the correlation improves as the number of reads averaged increases.

**Supplementary Fig. S7**. MAE decreases as the number of reads averaged per window increases in eGFP and Sequin datasets. The x-axis indicates the number of reads averaged per window, and the y-axis indicates the MAE per method. Solid lines indicate the maxpeak method results, and dashed lines indicate the median method results. The results reveal that the maxpeak method is generally superior to the median method.

**Supplementary Fig. S8**. Spearman correlations between poly(A) tail lengths and average read quality in Sequin R1 and R2 datasets. Each dot represents a read. The x-axis reveals the average read quality score, and the y-axis shows the estimated poly(A) lengths for each tool used in this study.

**Supplementary Table S1**. Number of reads corresponding to each stage of detection per tool for the eGFP dataset.

## Abbreviations

CNN, convolutional neural network; CTC, Connection-ist Temporal Classification; eGFP, enhanced green fluorescent protein; HMM, hidden Markov model; hpi, hours post-infection; IVT, *in vitro* transcribed; m, minutes; MAE, mean average error; MOI, multiplicity of infection; mRNA, messenger RNA; nt, nucleotides; ONT, Oxford Nanopore Technology; Poly(A), polyadenylate; RNN, recurrent neural network; s, seconds; SD, standard deviation; UTR, untranslated region.

## References

[1] Darnell JE, Wall R, Tushinski RJ. An adenylic acid-rich sequence in messenger RNA of HeLa cells and its possible relationship to reiterated sites in DNA. Proc Natl Acad Sci USA. 1971;68(6):1321–25. 10.1073/pnas.68.6.1321. 5288381 PMC389181

[2] Lee SY, Mendecki J, Brawerman G. A polynucleotide segment rich in adenylic acid in the rapidly-labeled polyribosomal RNA component of mouse sarcoma 180 ascites cells. Proc Natl Acad Sci USA. 1971;68(6):1331–35. 10.1073/pnas.68.6.1331. 5288382 PMC389183

[3] Terns MP, Jacob ST. Role of poly(A) polymerase in the cleavage and polyadenylation of mRNA precursor. Mol Cell Biol. 1989;9(4):1435–44. 10.1128/mcb.9.4.1435. 2566910 PMC362560

[4] Proudfoot NJ, Longley JI. The 3′ terminal sequences of human alpha and beta globin messenger RNAs: comparison with rabbit globin messenger RNA. Cell. 1976;9(4, Pt. 2):733–46. 10.1016/0092-8674(76)90137-9. 1035137

[5] Proudfoot NJ, Brownlee GG. 3′ Non-coding region sequences in eukaryotic messenger RNA. Nature. 1976;263(5574):211–14. 10.1038/263211a0. 822353

[6] Bardwell VJ, Wickens M, Bienroth S, et al. Site-directed ribose methylation identifies 2'-OH groups in polyadenylation substrates critical for AAUAAA recognition and poly(A) addition. Cell. 1991;65(1):125–33. 10.1016/0092-8674(91)90414-t. 1901516

[7] Keller W, Bienroth S, Lang KM, et al. Cleavage and polyadenylation factor CPF specifically interacts with the pre-mRNA 3′ processing signal AAUAAA. EMBO J. 1991;10(13):4241–49. 10.1002/j.1460-2075.1991.tb05002.x. 1756731 PMC453176

[8] Wilusz J, Shenk T, Takagaki Y, et al. A multicomponent complex is required for the AAUAAA-dependent cross-linking of a 64-kilodalton protein to polyadenylation substrates. Mol Cell Biol. 1990;10(3):1244–48. 10.1128/mcb.10.3.1244. 2304466 PMC361011

[9] Christofori G, Keller W. 3′ cleavage and polyadenylation of mRNA precursors in vitro requires a poly(A) polymerase, a cleavage factor, and a snRNP. Cell. 1988;54(6):875–89. 10.1016/s0092-8674(88)91263-9. 2842067

[10] Gilmartin GM, Nevins JR. An ordered pathway of assembly of components required for polyadenylation site recognition and processing. Genes Dev. 1989;3(12b):2180–90. 10.1101/gad.3.12b.2180. 2628166

[11] Takagaki Y, Ryner LC, Manley JL. Four factors are required for 3′-end cleavage of pre-mRNAs. Genes Dev. 1989;3(11):1711–24. 10.1101/gad.3.11.1711. 2558045

[12] Wahle E . A novel poly(A)-binding protein acts as a specificity factor in the second phase of messenger RNA polyadenylation. Cell. 1991;66(4):759–68. 10.1016/0092-8674(91)90119-j. 1878970

[13] Winters MA, Edmonds M. A poly(A) polymerase from calf thymus. Characterization of the reaction product and the primer requirement. J Biol Chem. 1973;248(13):4763–68. 10.1016/S0021-9258(19)43730-7. 4578090

[14] Lau N-C, Kolkman A, Schaik V, et al. Human Ccr4–Not complexes contain variable deadenylase subunits. Biochem J. 2009;422(3):443–53. 10.1042/bj20090500. 19558367

[15] Wolf J, Valkov E, Allen MD, et al. Structural basis for Pan3 binding to Pan2 and its function in mRNA recruitment and deadenylation. EMBO J. 2014;33(14):1514–26. 10.15252/embj.201488373. 24872509 PMC4158885

[16] Edmonds M, Vaughan MH, Nakazato H. Polyadenylic acid sequences in the heterogeneous nuclear RNA and rapidly-labeled polyribosomal RNA of HeLa cells: possible evidence for a precursor relationship. Proc Natl Acad Sci USA. 1971;68(6):1336–40. 10.1073/pnas.68.6.1336. 5288383 PMC389184

[17] Chang H, Lim J, Ha M, et al. TAIL-seq: genome-wide determination of poly(A) tail length and 3′ end modifications. Mol Cell. 2014;53(6):1044–52. 10.1016/j.molcel.2014.02.007. 24582499

[18] Eisen TJ, Eichhorn SW, Subtelny AO, et al. The dynamics of cytoplasmic mRNA metabolism. Mol Cell. 2020;77(4):786–99. e10. 10.1016/j.molcel.2019.12.005. 31902669 PMC7265681

[19] Begik O, Diensthuber G, Liu H, et al. Nano3P-seq: transcriptome-wide analysis of gene expression and tail dynamics using end-capture nanopore cDNA sequencing. Nat Methods. 2023;20(1):75–85. 10.1038/s41592-022-01714-w. 36536091 PMC9834059

[20] Beckel-Mitchener AC . Poly(A) tail length-dependent stabilization of GAP-43 mRNA by the RNA-binding protein HuD. J Biol Chem. 2002;277(31):27996–8002. 10.1074/jbc.m201982200. 12034726

[21] Fuke H, Ohno M. Role of poly (A) tail as an identity element for mRNA nuclear export. Nucleic Acids Res. 2007;36(3):1037–49. 10.1093/nar/gkm1120. 18096623 PMC2241894

[22] Gallie DR . The cap and poly(A) tail function synergistically to regulate mRNA translational efficiency. Genes Dev. 1991;5(11):2108–16. 10.1101/gad.5.11.2108. 1682219

[23] Wu H-Y, Ke T-Y, Liao W-Y, et al. Regulation of coronaviral poly(A) tail length during infection. PLoS One. 2013;8(7):e70548. 10.1371/journal.pone.0070548. 23923003 PMC3726627

[24] Kojima S, Sher-Chen EL, Green CB. Circadian control of mRNA polyadenylation dynamics regulates rhythmic protein expression. Genes Dev. 2012;26(24):2724–36. 10.1101/gad.208306.112. 23249735 PMC3533077

[25] Biziaev N, Shuvalov A, Salman A, et al. The impact of mRNA poly(A) tail length on eukaryotic translation stages. Nucleic Acids Res. 2024;52(13):7792–808. 10.1093/nar/gkae510. 38874498 PMC11260481

[26] Passmore LA, Coller J. Roles of mRNA poly(A) tails in regulation of eukaryotic gene expression. Nat Rev Mol Cell Biol. 2022;23(2):93–106. 10.1038/s41580-021-00417-y. 34594027 PMC7614307

[27] Lima SA, Chipman LB, Nicholson AL, et al. Short poly(A) tails are a conserved feature of highly expressed genes. Nat Struct Mol Biol. 2017;24(12):1057–63. 10.1038/nsmb.3499. 29106412 PMC5877826

[28] Mayr C, Bartel DP. Widespread shortening of 3′UTRs by alternative cleavage and polyadenylation activates oncogenes in cancer cells. Cell. 2009;138(4):673–84. 10.1016/j.cell.2009.06.016. 19703394 PMC2819821

[29] Huang G, Huang S, Wang R, et al. Dynamic regulation of tandem 3′ untranslated regions in zebrafish spleen cells during immune response. 2016;196(2):715–25. 10.4049/jimmunol.1500847. 26673144

[30] Melamed ZE, López-Erauskin J, Baughn MW, et al. Premature polyadenylation-mediated loss of stathmin-2 is a hallmark of TDP-43-dependent neurodegeneration. Nat Neurosci. 2019;22(2):180–90. 10.1038/s41593-018-0293-z. 30643298 PMC6348009

[31] Rund D, Dowling C, Najjar K, et al. Two mutations in the beta-globin polyadenylylation signal reveal extended transcripts and new RNA polyadenylylation sites. 1992;89(10):4324–28. 10.1073/pnas.89.10.4324. PMC49074 1374896

[32] Shien J-H, Su Y-D, Wu H-Y. Regulation of coronaviral poly(A) tail length during infection is not coronavirus species- or host cell-specific. Virus Genes. 2014;49(3):383–92. 10.1007/s11262-014-1103-7. 25034371 PMC7089208

[33] Salles FJ, Richards WG, Strickland S. Assaying the polyadenylation state of mRNAs. Methods. 1999;17(1):38–45. 10.1006/meth.1998.0705. 10075881

[34] Subtelny AO, Eichhorn SW, Chen GR, et al. Poly(A)-tail profiling reveals an embryonic switch in translational control. Nature. 2014;508(7494):66–71. 10.1038/nature13007. 24476825 PMC4086860

[35] Garalde DR, Snell EA, Jachimowicz D, et al. Highly parallel direct RNA sequencing on an array of nanopores. Nat Methods. 2018;15(3):201–6. 10.1038/nmeth.4577. 29334379

[36] Wan YK, Hendra C, Pratanwanich PN, et al. Beyond sequencing: machine learning algorithms extract biology hidden in Nanopore signal data. Trends Genet. 2022;38(3):246–57. 10.1016/j.tig.2021.09.001. 34711425

[37] Brouze A, Krawczyk PS, Dziembowski A, et al. Measuring the tail: methods for poly(A) tail profiling. WIREs RNA. 2023;14(1):e1737. 10.1002/wrna.1737. 35617484 PMC10078590

[38] Rand AC, Jain M, Eizenga JM, et al. Mapping DNA methylation with high-throughput nanopore sequencing. Nat Methods. 2017;14(4):411–13. 10.1038/nmeth.4189. 28218897 PMC5704956

[39] Silverman JD, Bloom RJ, Jiang S, et al. Measuring and mitigating PCR bias in microbiota datasets. PLoS Comput Biol. 2021;17(7):e1009113. 10.1371/journal.pcbi.1009113. 34228723 PMC8284789

[40] Kim D, Lee J-Y, Yang J-S, et al. The architecture of SARS-CoV-2 transcriptome. Cell. 2020;181(4):914–21. e10. 10.1016/j.cell.2020.04.011. 32330414 PMC7179501

[41] de Jong LC, Cree S, Lattimore V, et al. Nanopore sequencing of full-length BRCA1 mRNA transcripts reveals co-occurrence of known exon skipping events. Breast Cancer Res. 2017;19(1):127. 10.1186/s13058-017-0919-1. 29183387 PMC5706335

[42] Krause M, Niazi AM, Labun K, et al. tailfindr: alignment-free poly(A) length measurement for Oxford nanopore RNA and DNA sequencing. RNA. 2019;25(10):1229–41. 10.1261/rna.071332.119. 31266821 PMC6800471

[43] Leger A, Amaral PP, Pandolfini L, et al. RNA modifications detection by comparative Nanopore direct RNA sequencing. Nat Commun. 2021;12(1):7198. 10.1038/s41467-021-27393-3. 34893601 PMC8664944

[44] Chang JJ, Rawlinson D, Pitt ME, et al. Transcriptional and epi-transcriptional dynamics of SARS-CoV-2 during cellular infection. Cell Rep. 2021;35(6):109108. 10.1016/j.celrep.2021.109108. 33961822 PMC8062406

[45] Aw JGA, Lim SW, Wang JX, et al. Determination of isoform-specific RNA structure with nanopore long reads. Nat Biotechnol. 2021;39(3):336–46. 10.1038/s41587-020-0712-z. 33106685

[46] Simpson JT, Workman RE, Zuzarte PC, et al. Detecting DNA cytosine methylation using nanopore sequencing. Nat Methods. 2017;14(4):407–10. 10.1038/nmeth.4184. 28218898

[47] Oxford Nanopore Technologies. Dorado (PolyACalculator). Dorado documentation. https://nanoporetech.com/software/other/dorado. Accessed 30th April 2025.

[48] Teng H, Cao MD, Hall MB, et al. Chiron: translating nanopore raw signal directly into nucleotide sequence using deep learning. Gigascience. 2018;7(5):giy037. 10.1093/gigascience/giy037. PMC5946831 29648610

[49] Hardwick SA, Chen WY, Wong T, et al. Spliced synthetic genes as internal controls in RNA sequencing experiments. Nat Methods. 2016;13(9):792–98. 10.1038/nmeth.3958. 27502218

[50] Chang JJ-Y, Gleeson J, Rawlinson D, et al. Long-read RNA sequencing identifies polyadenylation elongation and differential transcript usage of host transcripts during SARS-CoV-2 in vitro. Infection Front Immunol. 2022;13:832223. 10.3389/fimmu.2022.832223. 35464437 PMC9019466

[51] He J, Ganesamoorthy D, Chang JJ-Y, et al. Utilizing Nanopore direct RNA sequencing of blood from patients with sepsis for discovery of co- and post-transcriptional disease biomarkers. BMC Infect Dis. 2025;25(1):692. 10.1186/s12879-025-11078-z. 40355874 PMC12070577

[52] Jia J, Lu W, Liu B, et al. An atlas of plant full-length RNA reveals tissue-specific and monocots-dicots conserved regulation of poly(A) tail length. Nat Plants. 2022;8(9):1118–26. 10.1038/s41477-022-01224-9. 35982302

[53] Payne A, Holmes N, Rakyan V, et al. BulkVis: a graphical viewer for Oxford nanoporebulk FAST5 files. Bioinformatics. 2019;35(13):2193–98. 10.1093/bioinformatics/bty841. 30462145 PMC6596899

[54] Chang JJ, Yang X, Teng H, et al. Supporting data for “Using Synthetic RNA to Benchmark Poly(A) Length Inference from Direct RNA Sequencing.” GigaScience Database. 2025. 10.5524/102736.40899916

