# Supplementary material for: Using synthetic RNA to benchmark poly(A) length inference from direct RNA sequencing
Source: Gigascience. 2025 Sep 3;14:giaf098. doi: 10.1093/gigascience/giaf098 (PMC12406214; doi:10.1093/gigascience/giaf098)
Supplement: giaf098_Supplemental_Files [file giaf098_supplemental_files.zip › Supplementary_boostnano_10_06_2025_FINAL.docx]

**Supplementary Information**

**Model structure of *Boostnano***

*BoostNano* is a machine learning-based tool based on *Chiron* - an end-to-end nanopore sequencing basecaller established using a deep learning CNN+RNN+CTC structure [1], which preprocesses ONT raw reads in FAST5 files before basecalling. Unlike traditional methods that involve basecalling, FASTQ formatting, and sequence alignment, *BoostNano* directly extracts, transforms, and analyzes the raw electrical current signal information within FAST5 files in its neural network model. The process involves segmenting the adaptor, poly(A) stalls, as well as transcription from the raw signal [1]. The inner detection model of *BoostNano* consists of two branches, a main branch and a side branch, each with three conventional layers and corresponding activation functions. The signal is processed in a streaming manner. Current signal block is fed into the side branch, and the output is then concatenated with the previous hidden state processed by the main branch, predicting the current signal’s state (adapter, poly(A) stalls, or transcription). This approach significantly reduces the detection time and can process approximately 200 to 400 direct RNA reads per minute per GPU. An output example of the segmentation plot for one RNA read generated by *BoostNano* is shown in **Figure S1**. The three red-line stalls indicate the locations of the adapter, poly(A) tail starting site, and poly(A) tail ending site.


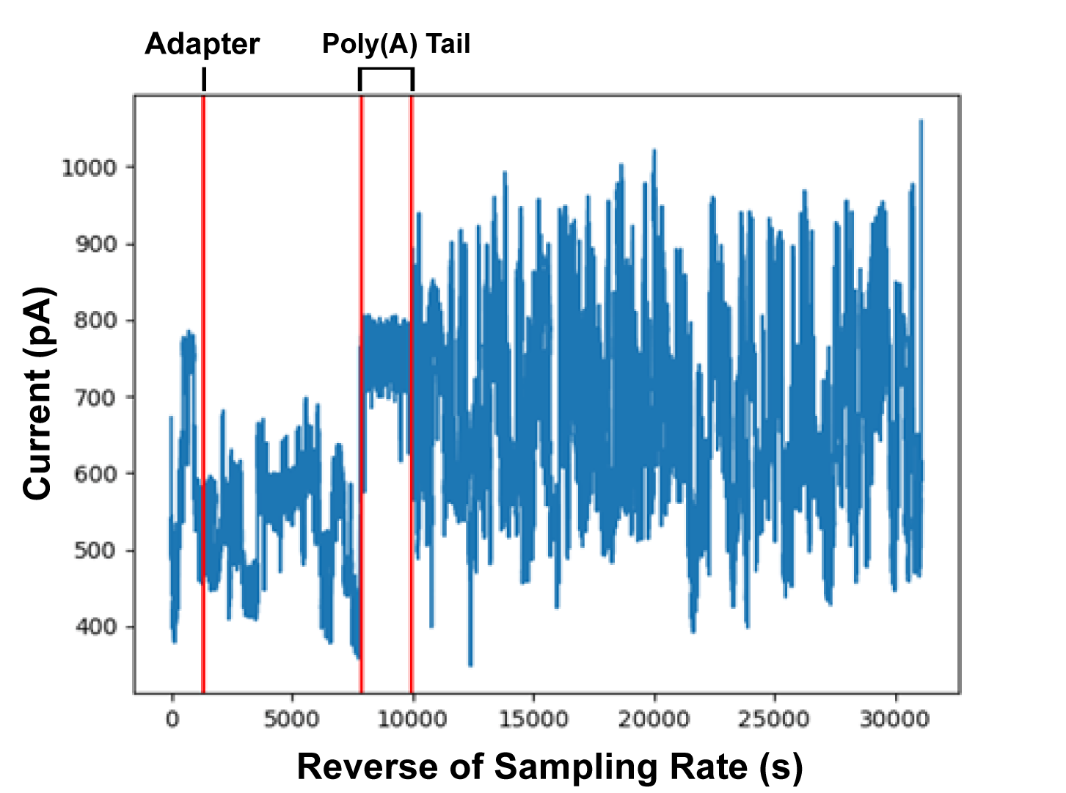


**Figure S1. An output example of the segmentation plot using *BoostNano*.** The first red line indicates the start of the adapter, second line indicates the position of the start of the poly(A) tail and the third line indicates the position of the end of the poly(A) tail. The Y-axis is the electric current (picoAmperes (pA)) and the X-axis is the reverse of sampling rate (1/4000 seconds (s)), as Nanopore sequencing samples at 4000 events per second.

The general model structure of *BoostNano* is as follows (**Figure S2**):


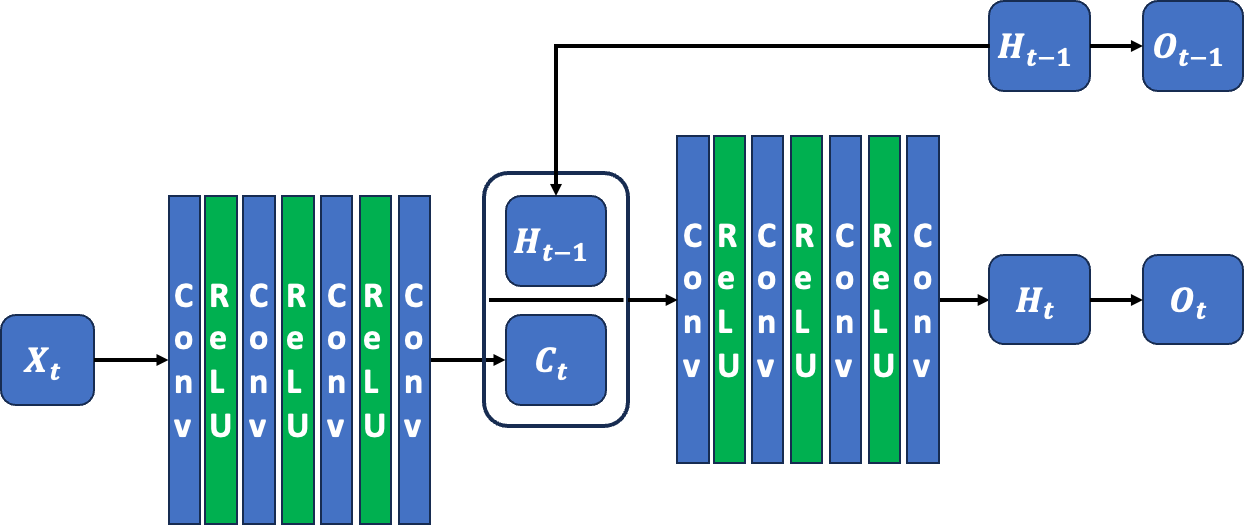


**Figure S2. General model structure of *BoostNano*.**

Given the signal $X=\{x_{t};t=1,\ldots,T\}$, *BoostNano* predicts the state of the signal at time $t$. Specifically, the state $\mathbf{h}_{t}=\{A1,A2,P,T\}$is determined, where $A1$denotes the sequencing adapter, $A2$ is the adapter added during poly(A) tailing, $P$ represents the poly(A) tail, and $T$ is the targeted transcription signal. The *BoostNano* model employs a continuous convolutional architecture. At each step, the neural network combines the previous hidden states with the current signal segments to predict the signal’s state, described as $\mathbf{h}_{t}=\mathbf{g}_{1}(\mathbf{f}(x_{t:t+k})\mid\mathbf{h}_{t-1})$ for $t>1$. Otherwise, for the initial step, it is $h_{0}=g_{2}\left( f\left( x_{0:k} \right) \right)$, where $k$ represents the width of the receptive field, and $\mathbf{f},\mathbf{g}_{1},\mathbf{g}_{2}$ are three distinct convolutional neural networks.

**Model training**

The training data was labeled by human agents using a labeling GUI we developed. During the labeling phase, each agent was required to identify the time points where transitions occurred, such as from the sequencing adapter to the poly(A) tailing adapter, then to the poly(A)-tail, and finally to the transcription RNA signal. To minimize personal bias, the data were labeled by three different human agents. The number of reads labeled by each agent was 436, 1,021, and 1,270 respectively. In total, 2,186 reads out of 2,727 passed our data review and were included in the final training dataset. Additionally, 1,165 reads were labeled and designated as the validation dataset. The models were trained using the Adam optimizer and trained for two epochs.

**Model inference**

During inference, the model outputs the probability of the signal state at each time point, and the maximum likelihood under the state Markov chain $(s\to A1\to A2\to P\to T)$ is calculated to obtain the final signal segmentation.

## **Statistical model construction**

### **Dataset selection**

For training, we used 10,000 overall reads from Vero 48 hpi dataset. The first testing subset consisted of 1,918 Sequin R1 reads from the Vero 24 hpi dataset, with a known poly(A) tail length of 30 nts. The second testing subset included 11,255 Sequin R2 reads from the Vero 24 hpi dataset with a known poly(A) tail length of 60 nt.

### **Data preprocessing and feature selection**

In order to properly train the statistical model, it was necessary to clean and preprocess the estimated outcomes from *tailfindr* and *BoostNano* for the training set. This involved removing outliers and handling missing values.

Subsequently, relevant features such as poly(A) tail starting sites, and ending sites were extracted, which were likely to have strong relationships with poly(A) tail lengths. It was crucial to ensure that the output pairs from both tools were accurately aligned, meaning that the extracted features along with the predicted tail lengths belonged to the same RNA reads.

**Poly(A) estimation**

The *BoostNano* output for each read includes essential information such as "read_id", "signal_interval", "adapter", "tail_start", "tail_end", "adapter_interval", "tail_interval", and "file_path". A linear regression models the relationship between a dependent variable (in this case, poly(A) tail length) and one or more independent variables (in this case, poly(A) tail starting and ending sites), which are estimated by *tailfindr* on the training set. This linear regression model will estimate the coefficients (weights) for each independent variable, indicating the strength and direction of their impact on the predicted poly(A) tail lengths. Once the model was trained by learning the relationship of existing data pairs from *tailfindr*, it could then estimate the tail length for new input features from *BoostNano*.

To measure how well the regression model fits the observed data values, several evaluation methods are commonly used in *R*: P-value Analysis, R-squared (Coefficient of Determination), and Adjusted R-squared. Furthermore, to compare the ability of the two tools to predict the poly(A) tail lengths accurately, we tested them on Sequin R1 and R2 datasets with known poly(A) lengths. We then used various evaluation metrics, such as Mean Absolute Error (MAE), Mean Absolute Percentage Error (MAPE), Mean Squared Error (MSE), and Root Mean Squared Error (RMSE), to assess how well the tools performed in predicting poly(A) tail lengths. These metrics provide quantitative measures of errors in the predicted values compared to the actual values in the testing subsets.

The following metrics were used to represent them:

MAE = (1/n) * Σ|y_pred - y_true|

MAPE = (1/n) * Σ(|(y_pred - y_true) / y_true|) * 100

MSE = (1/n) * Σ(y_pred - y_true)^2

RMSE = sqrt(MSE)

Note: "n" represents the number of samples in the testing subset, "y_pred" represents the predicted poly(A) tail lengths, and “y_true” represents the true poly(A) tail lengths.

To explore statistical relationships between poly(A) tail segmented starting sites and ending sites with the tail lengths, we built a linear regression model according to *tailfindr* estimation outcomes in *R*. The summary output of this model, shown in **Figure S8**, revealed that the predictors "tail_start" and "tail_end" had a significant impact on the response variable "tail_length", as indicated by their low p-values (0.0001). The relatively high R-squared and adjusted R-squared values (0.879) further suggested that a large portion of the variability in the response variable could be explained by the predictors included in the model. This indicated that the linear regression model was a good fit for the data and may be useful in predicting poly(A) tail lengths based on segmentation outcomes from *BoostNano*.


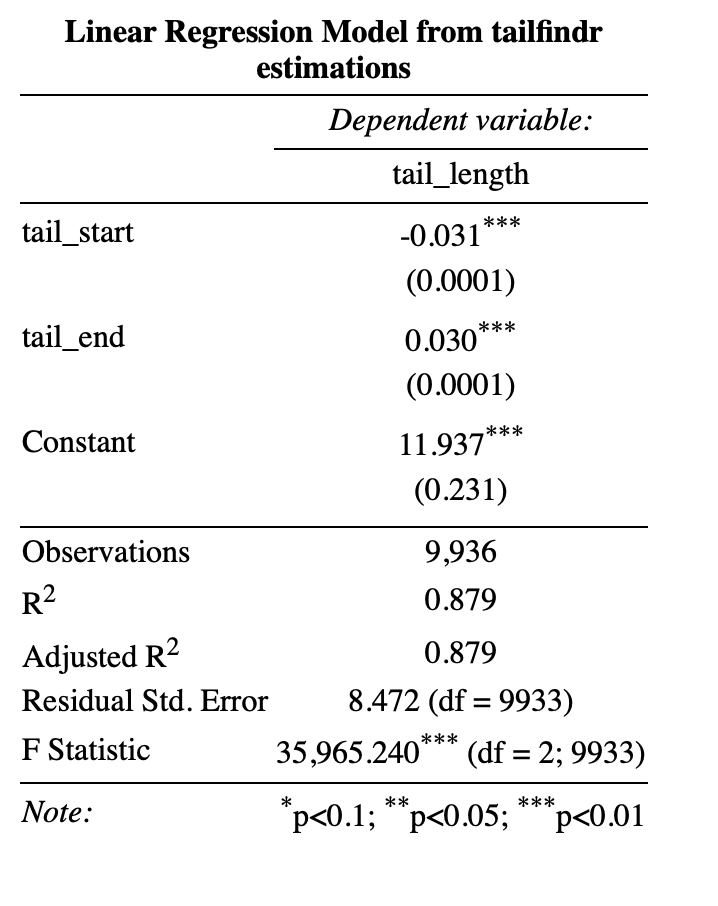


**Figure S3. The summary of the linear regression model.**

Therefore, for estimating the poly(A) tail from *BoostNano* outputs we used the following formula:

11.9374509 + tail_start * (-0.03127472) + tail_end * 0.03017431 (rounded to 2 decimal places).


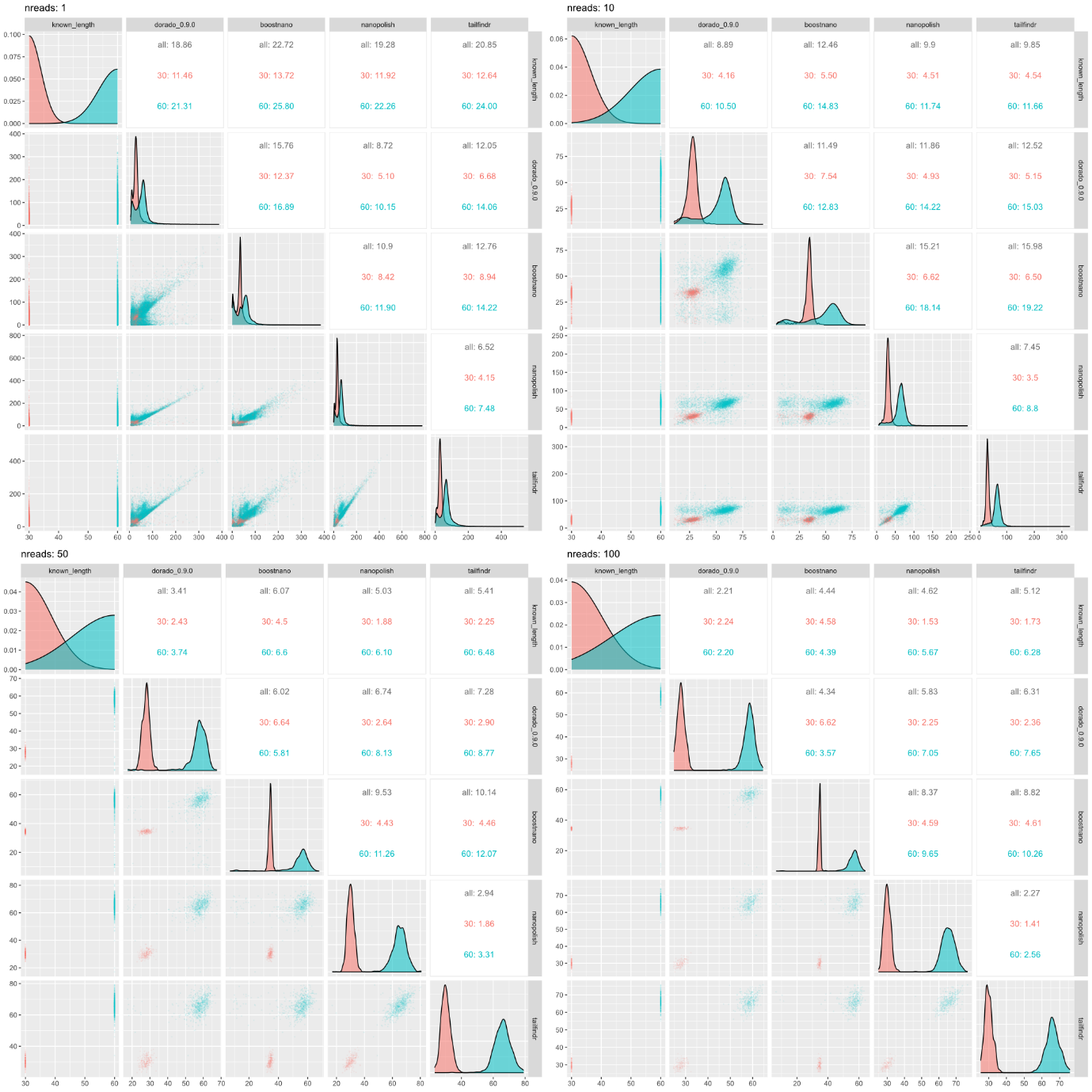


**Figure S4. Correlation plots of each tool in RNA002 Sequin dataset with averaging over windows with 1, 10, 50 and 100 reads via the maxpeak method.** Both X- and Y-axes indicate the poly(A) length. The figures indicate the MAE derived from all: all reads, 30: R1 reads, 60: R2 reads. The figure reveals that the correlation improves as the number of reads averaged increases.

**
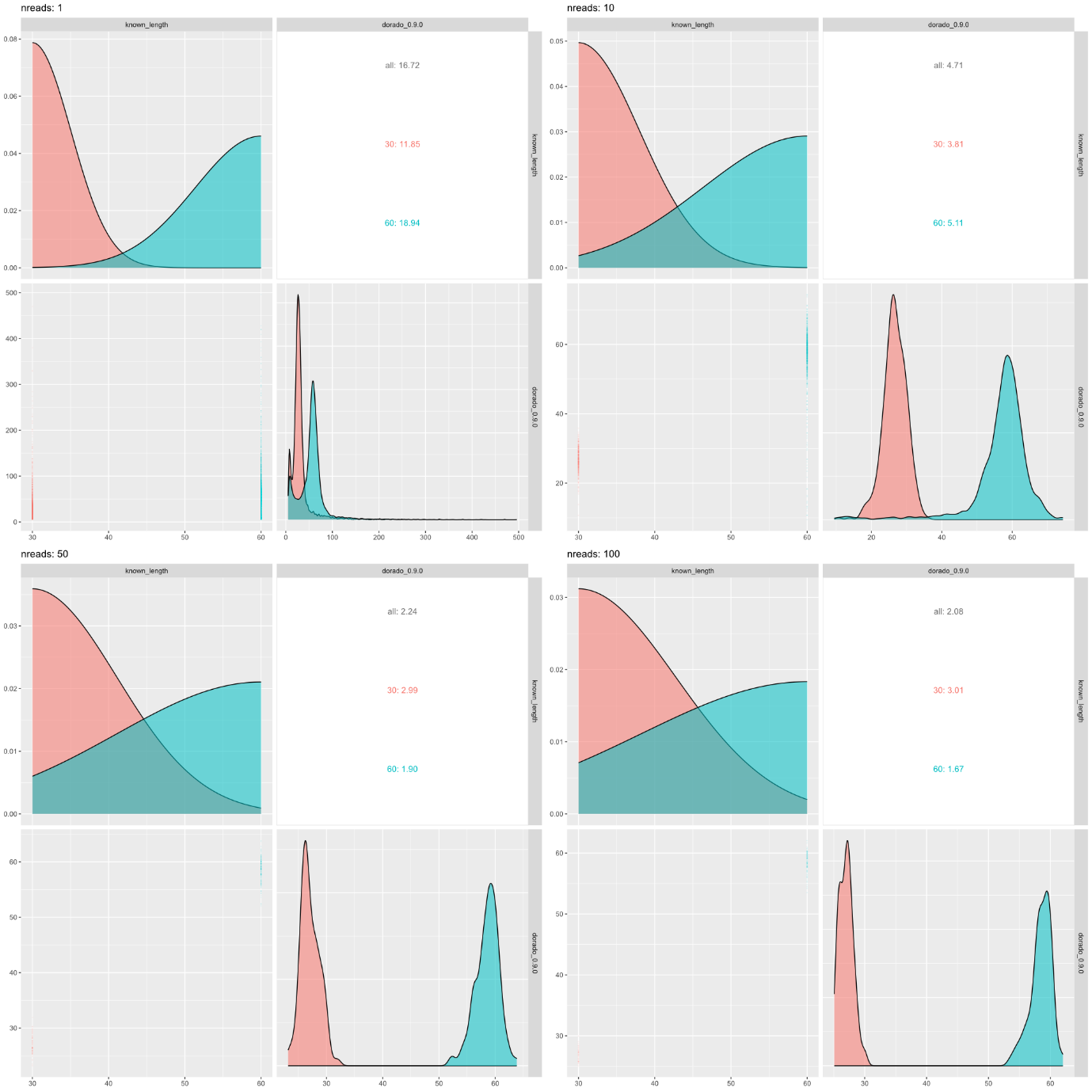
**

**Figure S5. Correlation plots of Dorado in RNA004 Sequin dataset with averaging over windows with 1, 10, 50 and 100 reads via the maxpeak method.** Both X- and Y-axes indicate the poly(A) length. The figures indicate the MAE derived from all: all reads, 30: R1 reads, 60: R2 reads. The figure reveals that the correlation improves as the number of reads averaged increases.

**
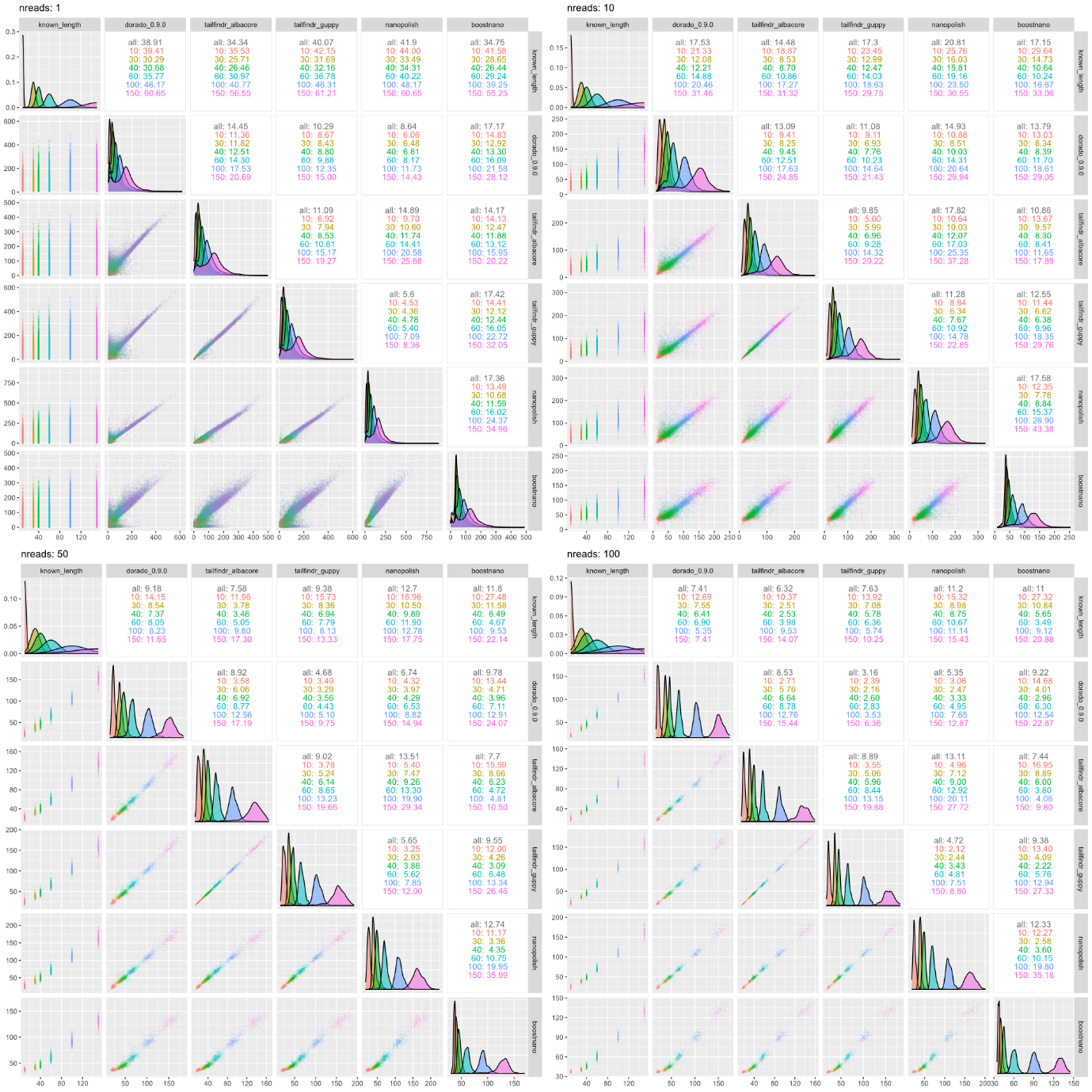
**

**Figure S6. Correlation plots of each tool in RNA002 eGFP dataset with averaging over windows with 1, 10, 50 and 100 reads via the maxpeak method.** Both X- and Y-axes indicate the poly(A) length. The figures indicate the MAE derived from all: all reads, 10: reads with known 10 nt poly(A) tail, 30: reads with known 30 nt poly(A) tail, 40: reads with known 40 nt poly(A) tail, 60: reads with known 60 nt poly(A) tail, 100: reads with known 100 nt poly(A) tail, 150: reads with known 150 nt poly(A) tail. The figure reveals that the correlation improves as the number of reads averaged increases.


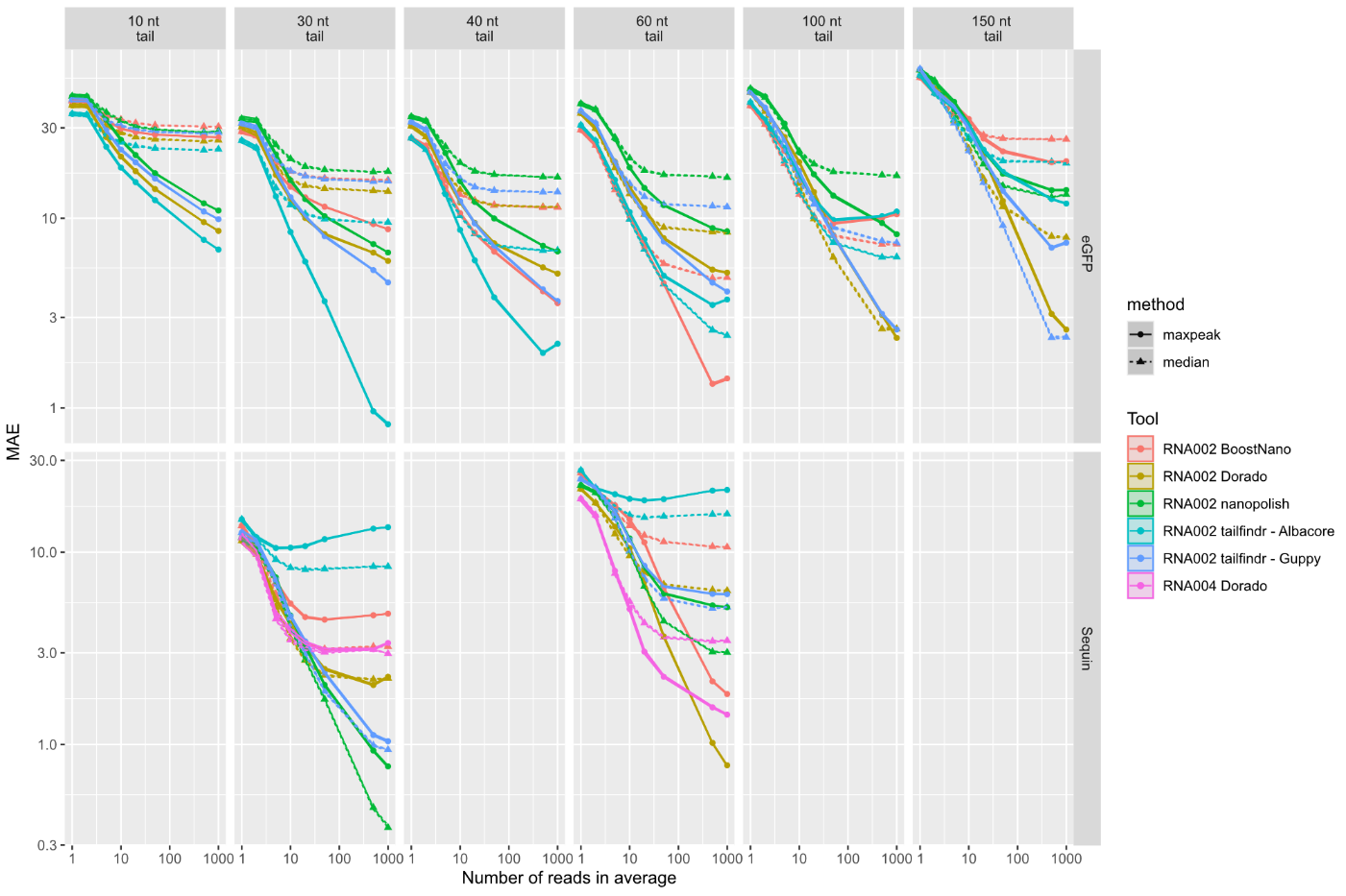


**Figure S7. MAE decreases as the number of reads averaged per window increases in eGFP and Sequin datasets.** X-axis indicates the number of reads averaged per window, and Y-axis indicates the MAE per method. Solid lines indicate the maxpeak method results and dashed lines indicate the median method results. The results reveal that the maxpeak method is generally superior to the median method.


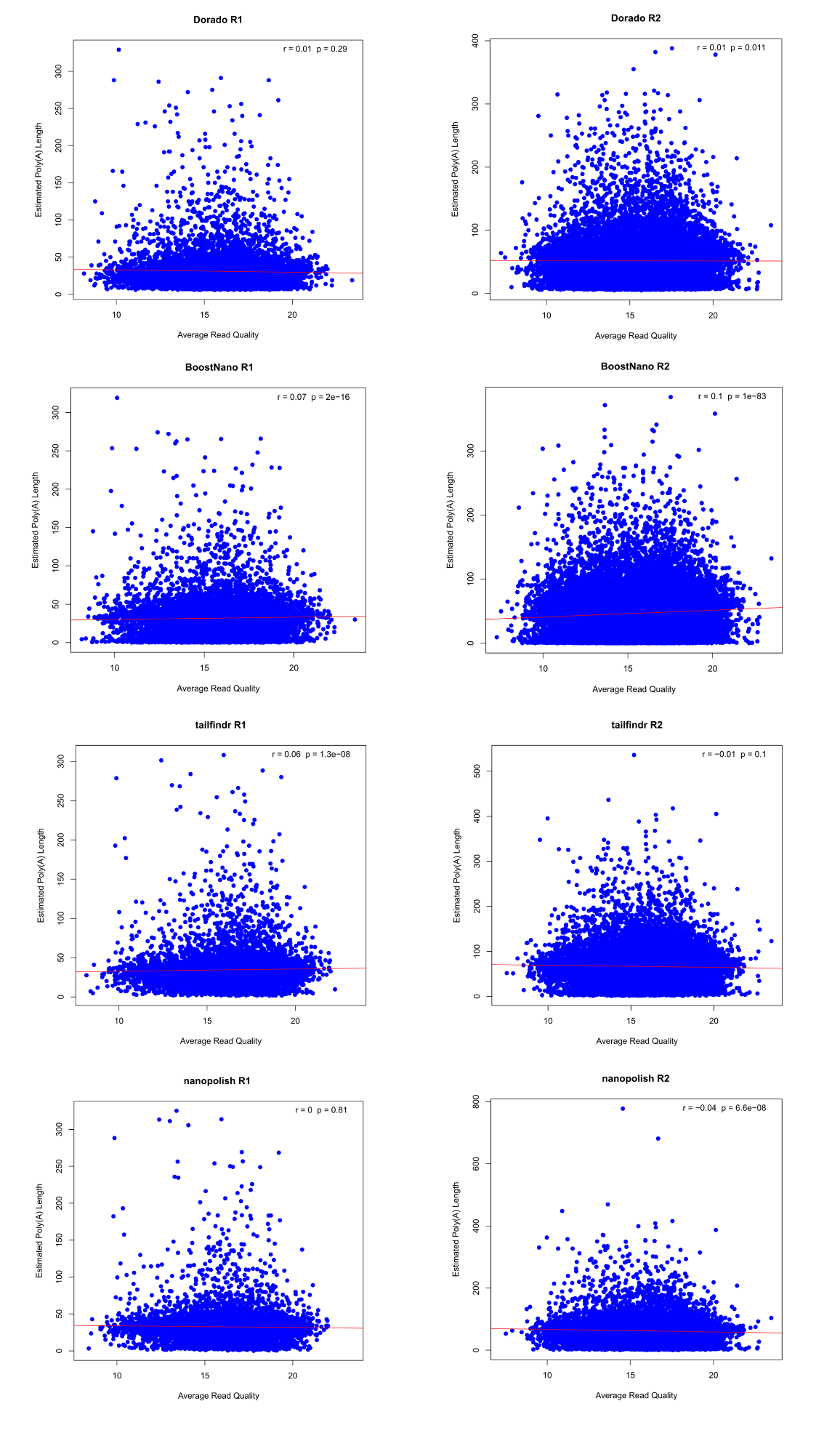


**Figure S8. Spearman correlations between poly(A) tail lengths and average read quality in Sequin R1 and R2 datasets.** Each dot represents a read. X-axis reveals the average read quality score and Y-axis shows the estimated poly(A) lengths for each tool used in this study.

**Table S1. Number of reads corresponding to each stage of detection per tool for the eGFP dataset.**

| Tool | Input reads | Reads Detected | Reads with Detected poly(A) | Reads aligned to barcode |
| --- | --- | --- | --- | --- |
| BoostNano | 592,571 | 592,571 | 591,874 (all) /589,571 (excluding < 0 nt) | 86,614 |
| Dorado | 592,571 | 530,785 | 530,785 | 85,019 |
| tailfindr Albacore | 592,571 | 588,700 | 580,525 | 85,658 |
| tailfindr Guppy | 592,571 | 588,698 | 580,525 | 85,658 |
| nanopolish | 592,571 | 539,644 | 287,626 | 67,037 |

**References**

1. Teng H, Cao MD, Hall MB, Duarte T, Wang S and Coin LJM. Chiron: translating nanopore raw signal directly into nucleotide sequence using deep learning. GigaScience. 2018;7 5 doi:10.1093/gigascience/giy037.
