# Supplementary material for: Using synthetic RNA to benchmark poly(A) length inference from direct RNA sequencing
Source: Gigascience. 2025 Sep 3;14:giaf098. doi: 10.1093/gigascience/giaf098 (PMC12406214; doi:10.1093/gigascience/giaf098)
Supplement: giaf098_GIGA-D-24-00432_Original_Submission [file giaf098_giga-d-24-00432_original_submission.pdf]

GigaScience

Using synthetic RNA to benchmark poly(A) length inference from direct RNA sequencing.

--Manuscript Draft--

Manuscript Number:	GIGA-D-24-00432
Full Title:	Using synthetic RNA to benchmark poly(A) length inference from direct RNA sequencing.
Article Type:	Technical Note
Funding Information:	National Health and Medical Research Council (GNT1195743) — Dr Lachlan Coin
Abstract:	Polyadenylation is a dynamic process which is important in cellular physiology. Oxford Nanopore Technologies direct RNA-sequencing provides a strategy for sequencing the full-length RNA molecule and analysis of the transcriptome and epi-transcriptome. There are currently several tools available for poly(A) tail length estimation, including well-established tools such as tailfindr and nanopolish, as well as two more recent deep learning models: Dorado and BoostNano. However, there has been limited benchmarking of the accuracy of these tools against gold-standard datasets. In this paper we evaluate four poly(A) estimation tools using synthetic RNA standards (Sequins), which have known poly(A) tail-lengths and provide a valuable approach to measuring the accuracy of poly(A) tail-length estimation. All four tools generate mean tail-length estimates which lie within 13% of the correct value. Overall, Dorado is recommended as the preferred approach due to its relatively fast run times, low coefficient of variation and ease of use with integration with base-calling.
Corresponding Author:	Lachlan Coin<br><br>AUSTRALIA
Corresponding Author Secondary Information:	
Corresponding Author's Institution:	
Corresponding Author's Secondary Institution:	
First Author:	Jessie J-Y Chang, PhD
First Author Secondary Information:	
Order of Authors:	Jessie J-Y Chang, PhD
	Xuan Yang, Masters of Bioinformatics
	Haotien Teng
	Benjamin Reames
	Vincent Corbin
	Lachlan Coin
Order of Authors Secondary Information:	
Additional Information:	
**Question**	**Response**
Are you submitting this manuscript to a special series or article collection?	No
**Experimental design and statistics**	Yes

Powered by Editorial Manager® and ProduXion Manager® from Aries Systems Corporation

Full details of the experimental design and statistical methods used should be given in the Methods section, as detailed in our Minimum Standards Reporting Checklist. Information essential to interpreting the data presented should be made available in the figure legends.<br><br>Have you included all the information requested in your manuscript?	
**Resources**<br><br>A description of all resources used, including antibodies, cell lines, animals and software tools, with enough information to allow them to be uniquely identified, should be included in the Methods section. Authors are strongly encouraged to cite Research Resource Identifiers (RRIDs) for antibodies, model organisms and tools, where possible.<br><br>Have you included the information requested as detailed in our Minimum Standards Reporting Checklist?	Yes
**Availability of data and materials**<br><br>All datasets and code on which the conclusions of the paper rely must be either included in your submission or deposited in publicly available repositories (where available and ethically appropriate), referencing such data using a unique identifier in the references and in the “Availability of Data and Materials” section of your manuscript.<br><br>Have you have met the above requirement as detailed in our Minimum Standards Reporting Checklist?	Yes

Powered by Editorial Manager® and ProduXion Manager® from Aries Systems Corporation

Manuscript Click here to access/download;Manuscript;Boostnano paper_14_09_24.docx

Using synthetic RNA to benchmark poly(A) length inference from direct RNA sequencing.

Jessie J-Y Chang[1] (jessiejieyouen.chang1@unimelb.edu.au), Xuan Yang[1] (xuanyang1@student.unimelb.edu.au), Haotian Teng[2] (haotiant@andrew.cmu.edu), Benjamin Reames[1] (b.reames@unimelb.edu.au), Vincent Corbin[1]* (vcorbin@unimelb.edu.au), Lachlan Coin[1,3]* (Lachlan.Coin@unimelb.edu.au)

1 Department of Microbiology and Immunology, University of Melbourne at The Peter Doherty Institute for Infection and Immunity, Melbourne, VIC, 3000, Australia

2 Ray and Stephanie Lane Computational Biology Department, School of Computer Science, Carnegie Mellon University, Pittsburgh, Pennsylvania, United States of America, 15213

3 Department of Clinical Pathology, University of Melbourne, Melbourne, VIC, 3000, Australia

*Corresponding authors: Vincent Corbin; Lachlan Coin

Abstract

Polyadenylation is a dynamic process which is important in cellular physiology. Oxford Nanopore Technologies direct RNA-sequencing provides a strategy for sequencing the full-length RNA molecule and analysis of the transcriptome and epi-transcriptome. There are currently several tools available for poly(A) tail-length estimation, including well-established tools such as *tailfindr* and *nanopolish*, as well as two more recent deep learning models: *Dorado* and *BoostNano*. However, there has been limited benchmarking of the accuracy of these tools against gold-standard datasets. In this paper we evaluate four poly(A) estimation tools using synthetic RNA standards (Sequins), which have known poly(A) tail-lengths and provide a valuable approach to measuring the accuracy of poly(A) tail-length estimation. All four tools generate mean tail-length estimates which lie within 12% of the correct value. Overall, *Dorado* is recommended as the preferred approach due to its relatively fast run times, low coefficient of variation and ease of use with integration with base-calling.

Keywords

Oxford Nanopore Technologies, poly(A) tail, estimation, segmentation, direct RNA sequencing

Findings

Background

Polyadenylation is a co-/post-transcriptional process in which a string of adenine nucleotides is added to the nascent messenger RNA molecule by enzymes such as polyadenylate (poly(A)) polymerases. This process is thought to increase the stability of the mRNA molecule [1], assist in export of the molecule from the cell nucleus [2] and is increasingly recognised as a dynamic process which influences timing and degree of protein production. As such, it is important to be able to measure polyadenylation accurately using a high-throughput assay to better understand its functional importance.

Oxford Nanopore Technologies (ONT) direct RNA-sequencing is an approach for single-molecule RNA-sequencing which does not require reverse transcription or polymerase chain reaction (PCR) amplification, thus avoiding amplification bias and retaining the original base and base-modification information [3-7]. Furthermore, full-length RNA molecules can be captured in one read, facilitating the identification of complex splicing patterns, RNA modifications and RNA secondary structures [8-13]. The nanopore sequencer records changes in ionic current as RNA passes through the pore in a custom FAST5/POD5 file. This raw data is then converted into sequence data using a custom deep learning model, such as *Dorado* or *Chiron* [14].

Nanopore sequencing of native RNA provides an attractive approach for measuring single-molecule poly(A) tail length. Thus, there have been several tools developed for estimating poly(A) tail length from raw nanopore signal (**Table 1**), including *nanopolish* [15], *tailfindr* [10], *Dorado* (developed by ONT) [16] as well as our in-house tool *BoostNano* (details described in **Supplementary Information**) [14]. There have been limited attempts to benchmark poly(A) tail length inference using gold-standard datasets with known poly(A) tail lengths.

RNA Sequins are synthetic *in vitro* transcribed RNA [17]. RNA Sequins are transcribed from an artificial chromosome which comprises 78 gene loci split into two classes which have either a 30 bp (R1) or a 60 bp (R2) poly(A) tail. We have previously sequenced several direct RNA libraries which consisted of a mixture of host cell-line RNA together with spiked-in RNA sequins (BioProject: PRJNA675370) [12]. In this paper we re-use a subset of this data to benchmark poly(A) tail length estimation using *BoostNano*, *tailfindr*, *nanopolish* and *Dorado.*

Table 1. Description of each poly(A) tail estimation tool benchmarked in this study

**Tool**	**Description**	**Reference**
BoostNano	Convolutional Neural Network (CNN)-Recurrent Neural Network (RNN)-Connection-ist Temporal Classification (CTC) architecture from *Chiron* basecaller used to find boundaries of poly(A) in raw signal	Teng et al., 2018. [14]
tailfindr	*R* tool, which uses the unaligned raw FAST5 data to estimate the poly(A) lengths via using the raw signal slope	Krause et al., 2019 [10]

	to refine the boundaries of potential poly(A) stretches and normalization with the read-specific nucleotide translocation rate	
nanopolish	Utilizes a predictive model in which a hidden Markov model (HMM - performs segmentation of the raw sequencing signal) and an estimator of the translocation rate are combined	Simpson et al., 2017 [15]
Dorado	Searches for the boundaries in the raw signal and estimates the poly(A) tail-length by taking into account the samples/base information, with adjustment for overestimation of the poly(A) tail. Primarily a basecalling tool, incorporates the poly(A) tail estimation during the basecalling itself	Oxford Nanopore Technologies [16]

Performance evaluation between *BoostNano, tailfindr, nanopolish* and *Dorado*

To compare the estimation performance of *BoostNano*, *tailfindr* v1.4, *nanopolish* v0.13.3 and *Dorado* v0.5.3, we tested these tools on two Sequin testing sets with known poly(A) tail lengths: R1 set with 30 nucleotide (nt) tails and R2 set with 60 nt tails (**Figure 1 & Table 1**) [17]. The four methods display a similar pattern in the density distribution, with a prominent normal-like peak near the expected poly(A) length, but also with a over-representation of shorter poly(A) tails, ranging at approximately ~0-10 nt (**Figure 1**). This peak was more prominent at ~0-3 nt in *BoostNano,* whereas the early peaks for *tailfindr*, *nanopolish* and *Dorado* were positioned at ~10 nt. We expected that these shorter peaks were derived from either fragmentation of the transcript, mis-priming of internal poly(A) stretches or degradation of the poly(A) tails. To test this, we inspected reads with <10 nt poly(A) tails and observed that the majority (68%) aligned within 20 nt of the 3' end of the Sequin transcripts. (**Figures 2a & b**). This suggested that the majority of these shorter poly(A) tails is due to fragmentation/degradation of the poly(A) tail. However, the remaining 32% of reads showed truncations in the middle of the reference transcript (**Figure 2a**), consistent with mis-priming or fragmentation of the physical RNA. This was supported by stretches of adenine bases observed at the 3' end of some of the truncated reads (**Figure 2c**). However, not all truncated reads showed poly(A) stretches (**Figure 2d**). Therefore, we hypothesize that some reads could have been able to be sequenced through mis-priming of these 3' ends in regions with higher A-content.

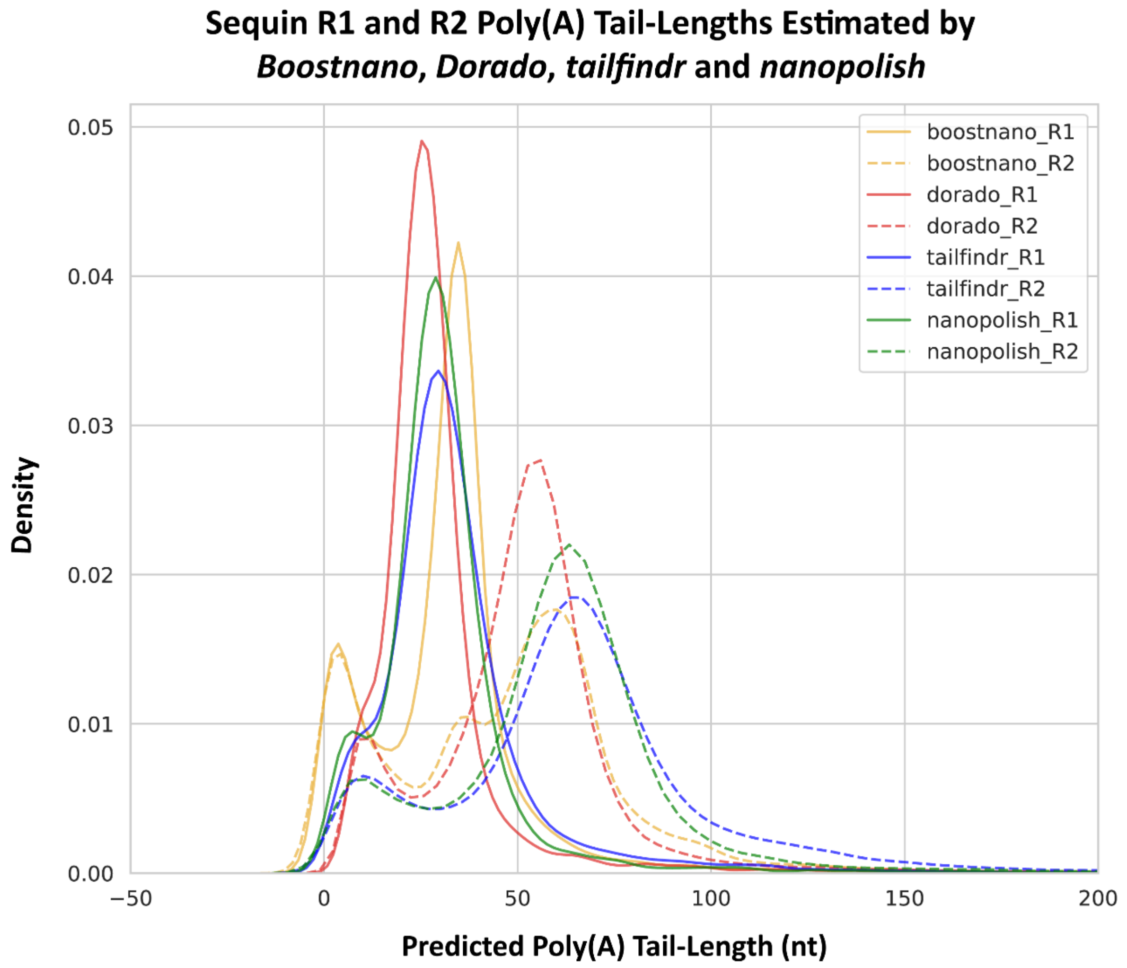

Figure 1. Density plots of estimated Sequin poly(A) tail-lengths. Outputs from *BoostNano* (yellow), *Dorado* (red), *tailfindr* (blue) and *nanopolish* (green). Expected poly(A) tail-lengths for R1 (solid lines) and R2 Sequins (dashed lines) are 30 nt and 60 nt, respectively. X-axis shows the predicted poly(A) tail-lengths of all reads and Y-axis reveals the density of the poly(A) tail-lengths.

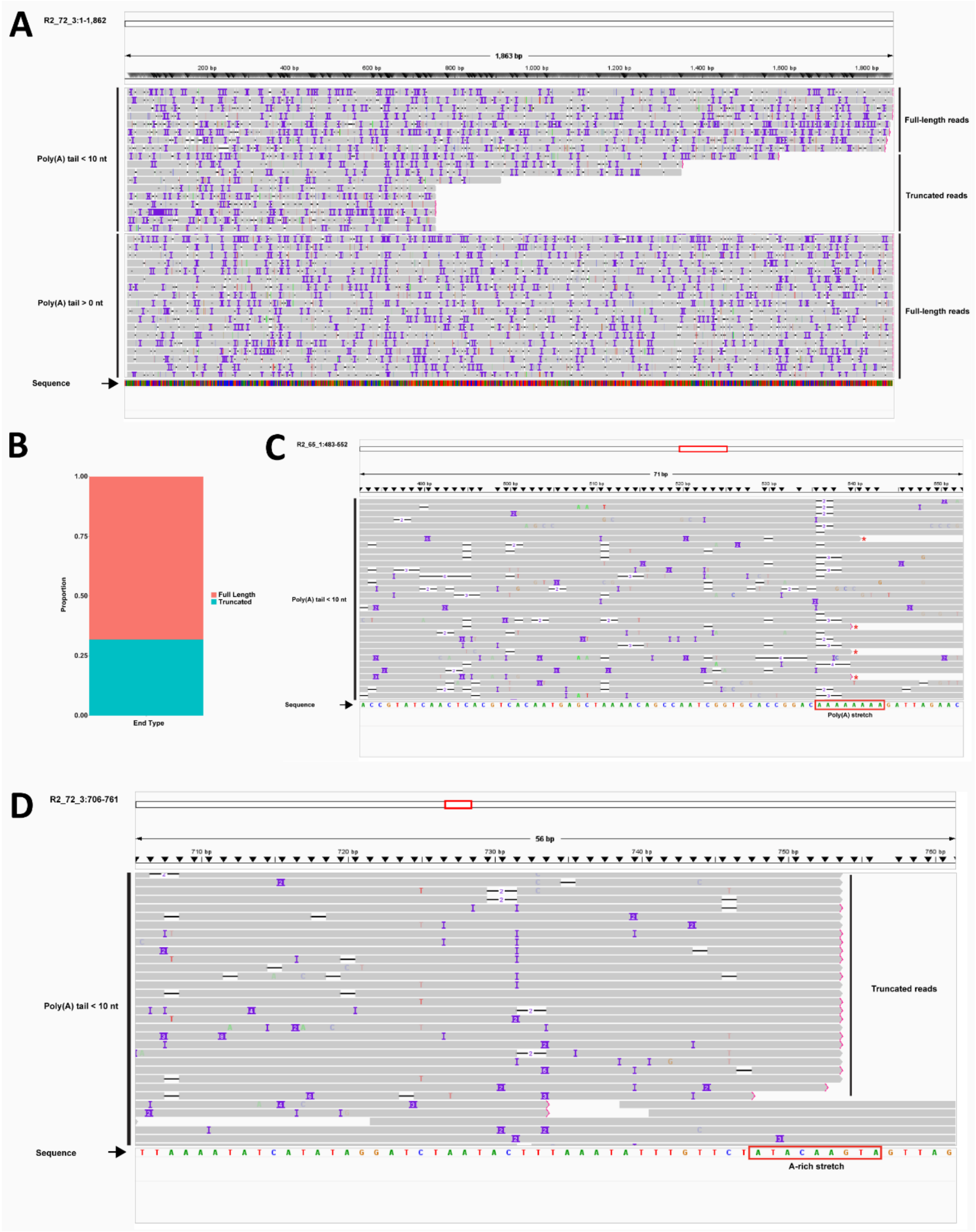

Figure 2. A representative subset of reads mapped to Sequins from the Vero 2 hpi dataset visualized with the Integrative Genomics Viewer (IGV) tool. A) A representative subset of reads mapping to the R2_72_3 Sequin transcript in Vero 2 hpi dataset. First subpanel shows a subset of reads with <10 nt poly(A) tails (estimated by *BoostNano*), showing that reads with <10 nt poly(A) tails are derived from both reads which have intact and also fragmented 3' ends. The second panel shows a representative subset of full-length reads in the entire Vero 2 hpi dataset (i.e. poly(A) tails >0 nt), which have intact 3' ends. **B)** Proportion of reads with truncated vs full-length 3' ends in the Vero 2 hpi dataset with poly(A) tail-lengths <10 nt (estimated by *BoostNano*). **C)** Truncated reads with poly(A) tails <10 nt (estimated by *BoostNano*) and 3' ends ending across an internal poly(A) stretch, mapped to R2_65_1 Sequin transcript marked with * (in red). **D)** The truncated reads with <10 nt poly(A) tails (estimated by *BoostNano*) which do not correlate to homopolymer poly(A) stretches at the 3' end, mapped to the R2_72_3 Sequin transcript. Each grey line indicates a read. "Sequence" indicates the sequence of bases which form the transcript, where A = green, T = red, G = yellow and C = blue. The red box at the top of the figure indicates the zoomed out proportion of the reference transcript.

The explanations above partially explain the earlier peak (~0-10 nt) in the density distribution (**Figure 1**) in all four methods, however, *BoostNano* particularly showed the mode of the peak presenting at even shorter poly(A) tail-lengths than *tailfindr*, *nanopolish* and *Dorado*. As the ONT reverse transcription adapter (RTA) used for reverse transcribing the native RNA strand has 10 polythymine (poly(T)) bases, it is likely that the minimum detection limit of poly(A) tails is 10 nt, which matches the ~10 nt peak with *tailfindr*, *nanopolish* and *Dorado*. We expect that the poly(A) tails shorter than 10 nt occur due to potential truncation of the poly(T) stretch of the RTA. Interestingly, upon investigating these earlier peaks, we found that *Dorado* excludes reads which are retained in the analysis by *BoostNano*, despite them being classified as passed reads (**Figure 3**). While an earlier peak of <10 nt was the most prominent amongst reads discarded by *Dorado*, we also observed peaks of ~40 nt and ~60 nt (according to *BoostNano*) amongst reads discarded by *Dorado*. Therefore, *Dorado* appears to be a more conservative approach than *BoostNano*.

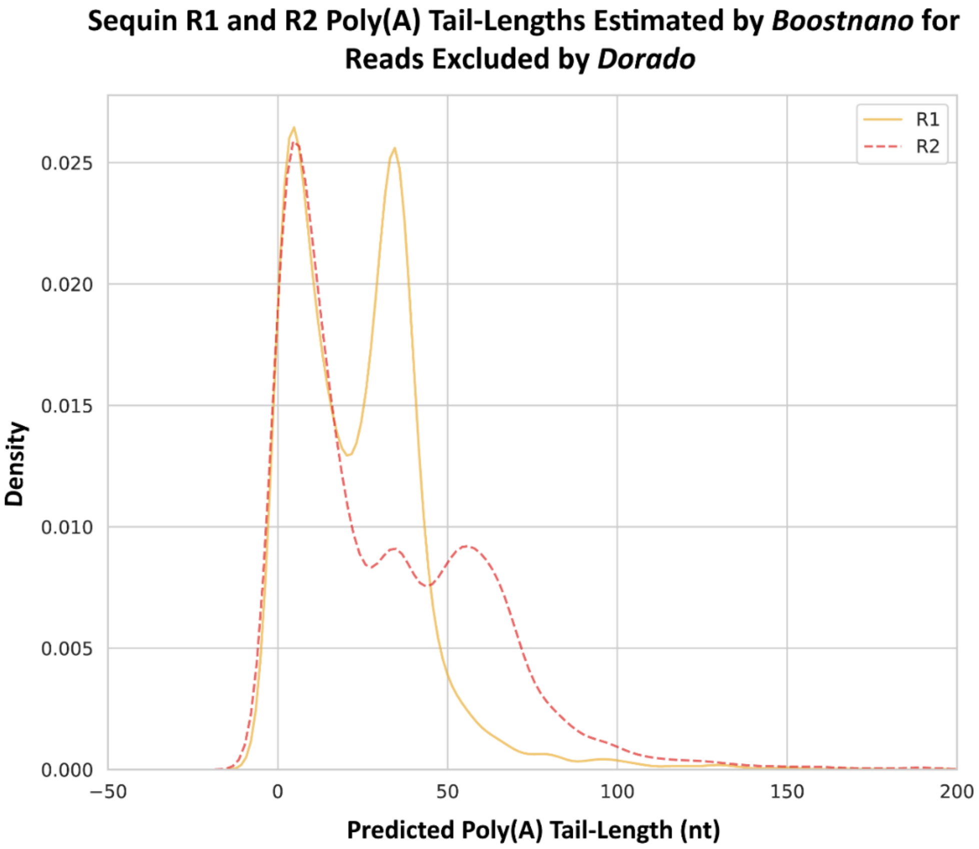

Figure 3. **Reads which have been filtered out by *Dorado* but retained in the *BoostNano* output.** R1 Sequin reads are indicated in yellow, and R2 Sequin reads are indicated in red. X-axis shows the predicted poly(A) tail length in nucleotides (nt), and the Y-axis shows the density distribution.

Following this, to estimate the accuracy of each method, we modelled the greatest peak as a normal distribution for each method and compared the mean and standard deviation (SD). The comparison was performed for both R1 and R2 Sequin sets (**Table 2**). In order to determine which normal distribution fit the peak best, we found the parameters (mean, SD) which minimize the root mean square error between the candidate normal distribution and the density distribution for an interval of 10 nt to the right of the mode. This is to remove biases due to an over-representation of the short-tailed reads. The peaks also lose their normal-like behavior for larger values. When comparing the results of the tools in both R1 and R2 testing sets, the deep learning tools - *BoostNano* and *Dorado* exhibited a more concentrated distribution around the mean, while *nanopolish* and *tailfindr* generally showed a wider spread of results (**Table 2**). With the R1 testing set, which had the shorter poly(A) tail-length, *tailfindr* showed the closest mean compared with the expected mean, and *BoostNano* showed the closest mean with the longer R2 testing set.

Table 2. Summary statistics for poly(A) tail-length estimation by four tools (*BoostNano*, *Dorado*, *tailfindr* and *nanopolish*) on two Sequin testing sets (R1 and R2).

Testing set	Method	Read Count	Mean (nt)	SD (nt)
R1 (30 nt)	*BoostNano*	14130	34.9	4.3
	Dorado	9536	26.1	5.7
	tailfindr	14106	29.3	8.8
	nanopolish	9036	28.2	7.8
R2 (60 nt)	*BoostNano*	42196	59.3	11.3
	Dorado	29568	54.5	9.5
	tailfindr	41173	65.4	12.6
	nanopolish	23645	64.1	11.0

Next, we compared the computational time required by each method to predict the tail-length of 4000 reads. For *BoostNano* and *Dorado*, we used one graphics processing unit (GPU) with 16G allocated RAM, while for *nanopolish* and *tailfindr*, which doesn't have the option to be run on GPU, we used one central processing unit (CPU) with 16G RAM and 1 thread for *nanopolish*. *Dorado* and *nanopolish* showed rapid computational times under 3 minutes (m), whereas *BoostNano* revealed the longest computational time at ~16 m and 52 seconds (s) (**Table 3**). *BoostNano* also offers the option of using the Application Programming Interface (API) call instead of the direct method, which omits the file copy implemented in the direct approach, reducing the run time to 8 m 8 s.

Table 3. Computational time efficiency to process 4000 reads with 1 GPU/CPU.

Method	Execution time (4000 reads)	GPU/CPU
BoostNano (direct)	16 m 52 s	GPU
BoostNano (API call)	8 m 8 s	GPU
Dorado	0 m 51 s	GPU
tailfindr	10 m 56 s	CPU

nanopolish	2 m 5 s	CPU

Discussion

In this technical note, we assessed the predictive performance of 4 poly(A) tail-length estimation tools - *tailfindr*, *nanopolish*, *BoostNano* and *Dorado* on two separate testing sets with known poly(A) tail-lengths. *BoostNano* and *tailfindr* tools provided estimation of the starting and ending positions of the poly(A) tails in event space while this information was absent in *Dorado* outputs. All four methods estimated the length of the tails within 1.2 SD as calculated above (**Table 2**). However, there were differences in the accuracy of the methods. On the R1 dataset, *BoostNano* showed a tighter distribution with the smallest SD, but its peak was the furthest from the correct value. *tailfindr* had the most accurate estimation but also the largest error interval. On the R2 dataset, however, the *BoostNano* estimation was the most accurate, with a fairly large SD, while *Dorado*, *nanopolish* and *tailfindr* were equally inaccurate with *Dorado* having a slightly smaller SD. Furthermore, *Boostnano* is more lenient in keeping reads for poly(A) estimation than *Dorado*. Overall, our results suggest that the four tools investigated in this study - *BoostNano*, *tailfindr*, *nanopolish* and *Dorado* have similar performance with their accuracy varying from one dataset to the other, with a potential length bias. The only obvious difference is in the speed of execution, where *Dorado* and *nanopolish* surpasses the rate of *BoostNano* or *tailfindr* (**Table 3**). Therefore, we expect *Dorado* to be implemented as the default method of poly(A) tail estimation in the near future, with the rapid estimation timeframe, comparable estimation lengths to other tools, conservative nature and the added benefit of ease of obtaining this information during basecalling.

This work demonstrates the value of having access to synthetic RNA molecules with known poly(A) tail-lengths for validating the accuracy of poly(A) tail estimation algorithms. As methods improve, we anticipate that these datasets will be valuable for assessing improvements in estimation of poly(A) tails.

Methods

Dataset

This study utilized publicly available ONT dRNA-seq datasets involving SARS-CoV-2-infected continuous cell lines (Vero, Calu-3 and Caco-2) derived from our previous study, with synthetic RNA - Sequins (BioProject: PRJNA675370) [12]. Briefly, Vero (African green monkey kidney epithelia), Calu-3 (Human lung adenocarcinoma epithelia) and Caco-2 (Human colorectal adenocarcinoma epithelia) cells were cultured in 6-well tissue culture plates at 37°C, 5% (v/v) CO_2. The Australian ancestral strain of SARS-CoV-2 (SARS-CoV-2/human/AUS/VIC01/2020) was used to infect these cells at a multiplicity of infection (MOI) of 0.1 and the cells were harvested at 0, 2, 24 and 48 hours post-infection (hpi). The total RNA was extracted and 6 µg of total RNA for Vero cells and 3 µg of total RNA for Calu-3 and Caco-2 cells + 10% of expected mRNA (5% of total RNA) of Sequins [17] were added to the sample pool. The

RNA was sequenced using the ONT Direct RNA Kit (SQK-RNA002), on R9.4.1 flow cells via the ONT MinION/GridION. For the purposes of this study, infected datasets from 24 and 48 hpi from all three cell lines and additionally 2 hpi from Vero cells were analyzed.

Availability of supporting source code and requirements

Project name: BoostNano

Project home page: https://github.com/haotianteng/BoostNano

Operating system(s): Platform independent

- Programming language: Python
- Other requirements: Pytorch
- License: Mozilla Public License, v. 2.0

Data Availability

The data sets supporting the results of this article are available in the NCBI repository, BioProject: PRJNA675370.

Declarations

List of abbreviations

Poly(A) - polyadenylate

ONT – Oxford Nanopore Technology

PCR – Polymerase Chain Reaction

HMM – Hidden Markov Model

CNN - Convolutional Neural Network

RNN - Recurrent Neural Network

CTC - Connection-ist Temporal Classification

SD – Standard deviation

m – minutes

s - seconds

Ethics approval and consent to participate

Not applicable.

Consent for publication

Not applicable.

Competing interests

LC has received funding from ONT unrelated to this work, as well as travel funding, also unrelated to this work.

Funding

This work was supported by a NHMRC-EU project grant (GNT1195743) to LC.

Authors' contributions

Conceptualization –J.J.-Y.C., H.T., V.C., L.C.

Methodology – H.T., V.C., L.C.

Software – H.T., V.C., L.C.

Validation - H.T., X.Y., V.C., L.C.

Formal analysis - H.T., B.R., X.Y., V.C., L.C.

Investigation - H.T., B.R., X.Y., V.C., L.C.

Resources - J.J.-Y.C., H.T., V.C., L.C.

Data Curation - J.J.-Y.C., V.C., L.C.

Writing - Original Draft - J.J.-Y.C., H.T., X.Y., V.C., L.C.

Writing - Review & Editing - J.J.-Y.C., H.T., X.Y., V.C., L.C.

Visualization – J.J.-Y.C., H.T., X.Y., V.C., L.C.

Supervision – V.C., L.C.

Project administration – J.J.-Y.C., V.C., L.C.

Funding acquisition – V.C., L.C.

Acknowledgements

This research was supported by The University of Melbourne's Research Computing Services and the Petascale Campus Initiative.

Authors' information

Not applicable.

References

1. Beckel-Mitchener AC. Poly(A) Tail Length-dependent Stabilization of GAP-43 mRNA by the RNA-binding Protein HuD. The Journal of Biological Chemistry. 2002;277 31:27996-8002. doi:10.1074/jbc.m201982200.
2. Fuke H and Ohno M. Role of poly (A) tail as an identity element for mRNA nuclear export. Nucleic Acids Research. 2007;36 3:1037-49. doi:10.1093/nar/gkm1120.
3. Garalde DR, Snell EA, Jachimowicz D, Sipos B, Lloyd JH, Bruce M, et al. Highly parallel direct RNA sequencing on an array of nanopores. Nature Methods. 2018;15 3:201-6. doi:10.1038/nmeth.4577.
4. Wan YK, Hendra C, Pratanwanich PN and Göke J. Beyond sequencing: machine learning algorithms extract biology hidden in Nanopore signal data. Trends in Genetics. 2022;38 3:246-57. doi:10.1016/j.tig.2021.09.001.
5. Brouze A, Krawczyk PS, Dziembowski A and Mroczek S. Measuring the tail: Methods for poly(A) tail profiling. WIREs RNA. 2023;14 1 doi:10.1002/wrna.1737.
6. Rand AC, Jain M, Eizenga JM, Musselman-Brown A, Olsen HE, Akeson M, et al. Mapping DNA methylation with high-throughput nanopore sequencing. Nature Methods. 2017;14 4:411-3. doi:10.1038/nmeth.4189.

7. Silverman JD, Bloom RJ, Jiang S, Durand HK, Dallow E, Mukherjee S, et al. Measuring and mitigating PCR bias in microbiota datasets. PLOS Computational Biology. 2021;17 7:e1009113. doi:10.1371/journal.pcbi.1009113.
8. Kim D, Lee J-Y, Yang J-S, Kim JW, Kim VN and Chang H. The Architecture of SARS-CoV-2 Transcriptome. Cell. 2020;181 4:914-21.e10. doi:10.1016/j.cell.2020.04.011.
9. de Jong LC, Cree S, Lattimore V, Wiggins GAR, Spurdle AB, kConFab I, et al. Nanopore sequencing of full-length BRCA1 mRNA transcripts reveals co-occurrence of known exon skipping events. Breast Cancer Res. 2017;19 1:127. doi:10.1186/s13058-017-0919-1.
10. Krause M, Niazi AM, Labun K, Torres Cleuren YN, Muller FS and Valen E. tailfindr: alignment-free poly(A) length measurement for Oxford Nanopore RNA and DNA sequencing. RNA. 2019;25 10:1229-41. doi:10.1261/rna.071332.119.
11. Leger A, Amaral PP, Pandolfini L, Capitanchik C, Capraro F, Miano V, et al. RNA modifications detection by comparative Nanopore direct RNA sequencing. Nature Communications. 2021;12 1 doi:10.1038/s41467-021-27393-3.
12. Chang JJ, Rawlinson D, Pitt ME, Taiaroa G, Gleeson J, Zhou C, et al. Transcriptional and epi-transcriptional dynamics of SARS-CoV-2 during cellular infection. Cell Rep. 2021;35 6:109108. doi:10.1016/j.celrep.2021.109108.
13. Aw JGA, Lim SW, Wang JX, Lambert FRP, Tan WT, Shen Y, et al. Determination of isoform-specific RNA structure with nanopore long reads. Nature Biotechnology. 2021;39 3:336-46. doi:10.1038/s41587-020-0712-z.
14. Teng H, Cao MD, Hall MB, Duarte T, Wang S and Coin LJM. Chiron: translating nanopore raw signal directly into nucleotide sequence using deep learning. GigaScience. 2018;7 5 doi:10.1093/gigascience/giy037.
15. Simpson JT, Workman RE, Zuzarte PC, David M, Dursi LJ and Timp W. Detecting DNA cytosine methylation using nanopore sequencing. Nature Methods. 2017;14 4:407-10. doi:10.1038/nmeth.4184.
16. Oxford Nanopore Technologies. Dorado (PolyACalculator).
17. Hardwick SA, Chen WY, Wong T, Deveson IW, Blackburn J, Andersen SB, et al. Spliced synthetic genes as internal controls in RNA sequencing experiments. Nature Methods. 2016;13 9:792-8. doi:10.1038/nmeth.3958.

Click here to access/download
Supplementary Material
Supplementary_boostnano_14_09_24.docx

Cover Letter

Click here to access/download;Personal Cover;cover_letter_gigascience.docx

THE UNIVERSITY OF MELBOURNE

24th September 2024

Dear Editors

Re: Submission of our manuscript Gigascience

We would like to submit the attached manuscript "*Using synthetic RNA to benchmark poly(A) length inference from direct RNA sequencing"* to *Gigascience* for consideration as a technical note.

Neural networks have proven essential to accurate base-calling – including the *Chiron* basecaller we previously published in Gigascience. However - until recently - neural networks have not been trained to directly identify poly(A) tail length. In this paper we describe a neural network approach characterising poly(A) tail length. Oxford Nanopore Technologies have also trained a neural network to characterise poly(A) tail length, which has been released as part of the *Dorado* basecaller. While these are exciting developments for poly(A) length estimation, the accuracy of these methods has not yet been explored.

In this paper we focus on benchmarking neural network based approaches to polyA length estimation with pre-existing tools *tailfindr* and *nanopolish*. We analyse synthetic RNA with known poly(A) tail length to develop a gold-standard dataset for benchmarking. We also describe our new approach *BoostNano*.

We provide estimates of the accuracy of poly(A) tail length estimation for each method and find that the neural network approaches are more accurate, and – in the case of *Dorado* – substantially faster than pre-existing approaches. We also identify a substantial portion of reads for which *Dorado* is unable to provide an estimate of poly(A) tail length, and show that this is perhaps largely due to a combination of polyA tail fragmentation and internal priming.

We believe that this manuscript will be an important point of reference for the poly(A) community, particularly as a description and evaluation of the commercial tool (*Dorado*) has not (and likely will not) be published.

Yours sincerely,

LM Coin

Lachlan Coin
Professor of Computational Biology
The Peter Doherty Institute for Infection and Immunity

Postal address:
792 Elizabeth Street, Melbourne, VIC 3000, Australia

Phone:
+61 3 8344 3831
