# Supplementary material for: Using synthetic RNA to benchmark poly(A) length inference from direct RNA sequencing
Source: Gigascience. 2025 Sep 3;14:giaf098. doi: 10.1093/gigascience/giaf098 (PMC12406214; doi:10.1093/gigascience/giaf098)
Supplement: giaf098_GIGA-D-24-00432_Revision_1 [file giaf098_giga-d-24-00432_revision_1.pdf]

# GigaScience
# Using synthetic RNA to benchmark poly(A) length inference from direct RNA sequencing
--Manuscript Draft--

| | |
|---|---|
| **Manuscript Number:** | GIGA-D-24-00432R1 |
| **Full Title:** | Using synthetic RNA to benchmark poly(A) length inference from direct RNA sequencing |
| **Article Type:** | Technical Note |
| **Funding Information:** | National Health and Medical Research Council (GNT1195743) — Dr Lachlan Coin |
| **Abstract:** | Polyadenylation is a dynamic process which is important in cellular physiology, which has implications in mRNA decay rates, translation efficiency, and isoform-specific regulation. Oxford Nanopore Technologies direct RNA-sequencing provides a strategy for sequencing the full-length RNA molecule and analysis of the transcriptome. Several tools are currently available for poly(A) tail length estimation, including well-established methods like tailfindr and nanopolish, as well as more recent deep learning models like Dorado. However, there has been limited benchmarking of the accuracy of these tools against gold-standard datasets. In this paper, we present our novel deep-learning poly(A) estimation tool – BoostNano and compare with three existing tools - tailfindr, nanopolish and Dorado. We evaluate the four poly(A) estimation tools, using two sets of synthetic in vitro-transcribed RNA standards with known poly(A) tail lengths - Sequin (30 or 60 nucleotides) and enhanced Green Fluorescent Protein (10-150 nucleotides) RNA. Analyzing datasets with known ground truth values is a valuable approach to measuring the accuracy of poly(A) length estimation. The tools demonstrated length- and sample-dependent performance, and accuracy was enhanced by averaging over multiple reads via estimation of the peak of the density distribution. Overall, Dorado is recommended as the preferred approach due to its relatively fast run times, low mean average error and ease of use with integration with base-calling. These results provide a reference for poly(A) tail length estimation analysis, aiding in improving our understanding of the transcriptome and the relationship between poly(A) tail length and other transcriptional mechanisms, including transcript stability or quantification. |
| **Corresponding Author:** | Lachlan Coin<br>University of Melbourne<br>Melbourne, AUSTRALIA |
| **Corresponding Author Secondary Information:** | |
| **Corresponding Author's Institution:** | University of Melbourne |
| **Corresponding Author's Secondary Institution:** | |
| **First Author:** | Jessie J-Y Chang, PhD |
| **First Author Secondary Information:** | |
| **Order of Authors:** | Jessie J-Y Chang, PhD<br>Xuan Yang, Masters of Bioinformatics<br>Haotian Teng<br>Jianshu Zhang<br>Benjamin Reames<br>Shuxin Zhang<br>Vincent Corbin<br>Lachlan Coin |

Powered by Editorial Manager® and ProduXion Manager® from Aries Systems Corporation

| Order of Authors Secondary Information: | |
|---|---|
| Response to Reviewers: | Dear Editor,<br><br>We sincerely appreciate the time and dedication of you and the reviewers to have spent on reviewing this manuscript.<br><br>Please kindly find our responses to reviewers' comments in blue below and please note that all grammar correction suggestions have been appropriately incorporated into the new version of the manuscript.<br><br>Please note that we have updated the Dorado dataset to include data from the latest version of Dorado v0.9.0.<br><br>External email: Please exercise caution<br><br>GIGA-D-24-00432<br>Using synthetic RNA to benchmark poly(A) length inference from direct RNA sequencing.<br>Jessie J-Y Chang; Xuan Yang; Haotien Teng; Benjamin Reames; Vincent Corbin; Lachlan Coin<br>GigaScience<br><br>Dear Dr Coin,<br><br>Happy New Year!<br><br>Your manuscript "Using synthetic RNA to benchmark poly(A) length inference from direct RNA sequencing." (GIGA-D-24-00432) has been assessed by our reviewers. Although it is of interest, we are unable to consider it for publication in its current form. The reviewers have raised a number of points which we believe would improve the manuscript and may allow a revised version to be published in GigaScience.<br><br>Their reports, together with any other comments, are below. Please also take a moment to check our website at https://www.editorialmanager.com/giga/ for any additional comments that were saved as attachments.<br><br>In addition, please register any new software application in the bio.tools and SciCrunch.org databases to receive RRID (Research Resource Identification Initiative ID) and biotoolsID identifiers, and include these in your manuscript. Computational workflows should be registered in workflowhub.eu and the DOIs cited in the relevant places in the manuscript. These will facilitate tracking, reproducibility and re-use of your tool.<br><br>If you are able to fully address these points, we would encourage you to submit a revised manuscript to GigaScience. Once you have made the necessary corrections, please submit online at:<br><br>We have added boostNano to bio.tools and SciCrunch.org databases and referred the relevant ID's in the manuscript as the Editor has kindly suggested. We have additionally noted links as appropriate to refer to data and scripts on Figshare, which will be replaced with DOIs after acceptance.<br><br>https://www.editorialmanager.com/giga/<br><br>If you have forgotten your username or password please use the "Send Login Details" link to get your login information. For security reasons, your password will be reset.<br><br>Please include a point-by-point within the 'Response to Reviewers' box in the submission system. Please ensure you describe additional experiments that were carried out and include a detailed rebuttal of any criticisms or requested revisions that you disagreed with. Please also ensure that your revised manuscript conforms to the journal style, which can be found in the Instructions for Authors on the journal homepage. If the data and code has been modified in the revision process please be sure to update the public versions of this too. |

*Powered by Editorial Manager® and ProduXion Manager® from Aries Systems Corporation*

The due date for submitting the revised version of your article is 07 Apr 2025.

I look forward to receiving your revised manuscript soon.

Best wishes,

Hongfang Zhang
GigaScience
www.gigasciencejournal.com

Reviewer reports:
Reviewer #1: In this manuscript, the authors present a benchmark to assess the performance of different tools designed for estimation of polyA tail length from Nanopore direct RNA-sequencing data. These tools include tailfindr, nanopolish, Dorado and Boost Nano.
Benchmarks on tools and algorithms to analyze Nanopore data, both third party tools and official ONT releases, are of utmost importance for the field. The use of synthetic constructs with known ground truth is recommended as well. Consequently, this study has the potential to provide a significant contribution to the field.

In the current form, I can however not recommend it for publication in GigaScience. My major concerns are:
a)Use of only RNA002 data. This chemistry is outdated and thus the Benchmark is only relevant for old, possibly already published data. A comprehensive Benchmark should also include RNA004 and available tools there (at least Dorado).

We have generated new RNA004 data of sequins for this revision. We have analysed this data using Dorado v0.9.0 in our manuscript. We attempted to use other tools with RNA004 data, but this was not possible due to incompatibility. Nevertheless, we are able to compare the estimates from RNA004 data to RNA002 data.

b) The current data set only contains two polyA tail length, which are relatively short and do not cover longer polyA tails that are common e.g. in mammalian cells. A proper Benchmark should show the performance of the analyzed tools over a range of polyA tail lengths.

We have added synthetic IVT eGFP RNA data that span over a range of polyA tail lengths derived from the developers of tailfindr. The data include RNA with poly(A) tails with 10, 30, 40, 60, 100, 150 nt.

Minor comments:
Abstract: "All four tools generate mean tail-length estimates which lie within 13% of the correct value." The value of 13% is given in the Abstract from the submission system, wherease the abstract in the Main text says 12%. Which value is correct?

We have now removed this statement from the Abstract and the Main Text as we no longer focus on mean tail length.

2) Background, first paragraph: the role of the polyA tail in RNA circularization, which is required for efficient translation of cellular mRNAs is not mentioned. Reference is missing for "is increasingly recognised as a dynamic process which influences timing and degree of protein production."

This has been moved to the second paragraph of the background - the role of the poly(A) tail in RNA circularization and the appropriate references were added (lines 65-68).

3) Background, second paragraph: Chiron seems to be a relatively old basecaller (no models for new chemistries). It should be mentioned here that it is required for BoostNano.

Chiron is not required for BoostNano, as basecalling is not necessary for using the tool. Instead, the model structure of Chiron is used as a basis for BoostNano. We have

clarified this in Table 1 – “Convolutional Neural Network (CNN)-Recurrent Neural Network (RNN)-Connection-ist Temporal Classification (CTC) architecture from Chiron basecaller used to find boundaries of poly(A) in raw signal, basecalling not required”

4) Mis-priming of internal polyA sites may an important confounding (and currently overlooked) source of errors in Nanopore sequencing. This should be quantified properly and analyzed in more detail (length of these stretches, influence of other nucleotides within the A-rich stretch, etc.). Should be done as well on whole transcriptome data with more possible mispriming sites.

We have analyzed the whole Sequin transcriptome dataset to understand and quantify priming due to internal polyA sites or fragmentation via visualising the 10 nt downstream of the 3’ end of the mapped transcript in the reference transcriptome, as the poly(T) stretch of the RTA is 10 nt long. Furthermore, we analyzed the poly(A) content by calculating the percentage of adenines in that 10 nt stretch. This information was added as Figures 3B, D-E.

5) Why do the authors think that the poly(T) stretch of the RTA might be truncated? This is composed of DNA oligos, which should be quite stable

Yes, we agree that DNA oligos are much more stable than RNA oligos and therefore should be quite stable. However, that does not mean that truncation and fragmentation cannot happen with DNA oligos. Mechanical forces, freeze-thaw and enzymatic hydrolysis can contribute to the degradation of DNA oligos. Please see the following publication discussing the effect of freeze-thaw to DNA oligos with 10T’s (Davis, O'Brie & Bentzley, 2000 - Analysis of the Degradation of Oligonucleotide Strands During the Freezing/Thawing Processes Using MALDI-MS DOI: 10.1021/ac000225s).

6) What are the parameters for filtering used by Dorado and BoostNano? Can the authors explain, why the filtered reads differ?

We have only utilized default parameters for both Dorado and BoostNano, hence no specific filtering parameter was utilized. Dorado bases its poly(A) estimation on an analysis of the signal, searching for a low variability region near its identified anchor point (typically the cDNA primer or RNA adapter) and an estimate of the translocation speed based on the move table of the read. If the low variability region or anchor point cannot be located, the reads could be filtered out. BoostNano assigns poly(A) tails to all reads.

7) Dorado seems to systematically underestimate polyA tail length. Is this true also for data generated with RNA004 chemistry and longer polyA tails?

Dorado’s poly(A) tail length estimates tend to be the most accurate - please see updated Figures 1a and 1b. When comparing RNA002 and RNA004, Dorado seems to underestimate poly(A) tails more in RNA004 data than RNA002 data.

Reviewer #2: This manuscript addresses a relevant and timely question: benchmarking poly(A) tail-length estimation tools (BoostNano, tailfindr, nanopolish, and Dorado) using synthetic RNA standards (Sequins) with known tail lengths. Poly(A) tail-length estimation is increasingly important for understanding mRNA stability, processing, and regulation at the single-molecule level. As direct RNA sequencing expands in use, reliable methods to measure poly(A) tail lengths are needed. The study's design—leveraging Sequins as a "gold standard" to benchmark tools—is strong and fills an area is need in current literature. The analysis is thorough in its basic comparisons, and the results are likely to be useful to researchers who need to choose suitable software for poly(A) tail analysis. However, the manuscript would benefit from deeper contextualization, more rigorous statistical methodology, and clearer reporting of computational details. Ensuring reproducibility and providing clearer guidance on interpreting the results in real biological contexts would strengthen the manuscript. The suggestions below are aimed at making the study more valuable to the community.
For this reason, my recommendation is Revisions ARE Needed
Introduction

| | |
|---|---|
| | Abstract: ★★★★☆ (4/5) Actually in place of the introduction, it has it strengths:<br>The introduction adequately outlines why polyadenylation is biologically important and why direct RNA sequencing provides a unique opportunity for poly(A) tail-length estimation.<br>It justifies the use of Sequins as synthetic standards, which is a robust approach to derive ground-truth tail lengths.<br><br>Areas for Improvement: The introduction could better connect poly(A) tail-length estimation to downstream applications. For instance, mention how accurate tail-length estimation could improve understanding of mRNA decay rates, translation efficiency, or isoform-specific regulation.<br><br>We have included wording to connect poly(A) estimation to downstream applications in both the abstract and the introduction (lines 32-34, 65-76).<br><br>Adding references that contextualize poly(A) tail dynamics in broader biological phenomena would help readers understand the significance.<br>For example, it is almost a necessity to cite work such as "Roles of mRNA poly(A) tails in regulation of eukaryotic gene expression" by Lori A. Passmore & Jeff Coller (2022, Nature Reviews Molecular Cell Biology) which provides a comprehensive analysis of poly(A) tail dynamics and their impact on mRNA decay, stability, and translation regulation. P & C (2022) also expands on these principles by discussing the mechanistic underpinnings of poly(A)-mediated decay and translation regulation, making it a broader and more recent contribution to polyadenylation biology, which the authors should consider.<br><br>Thank you for the suggestion – we have now referenced this work and other relevant publications in our manuscript (page 2).<br><br>Grammar of the abstract:<br>Error: "There are currently several tools available for poly(A) tail-length estimation, including well-established tools such as tailfindr and nanopolish, as well as two more recent deep learning models: Dorado and BoostNano."<br>Suggestion: "Several tools are currently available for poly(A) tail-length estimation, including well-established methods like tailfindr and nanopolish, as well as two more recent deep learning models: Dorado and BoostNano."<br><br>Error: "which lie within 12% of the correct value."<br>Suggestion: "that lie within 12% of the correct value."<br><br>Clarify the library preparation steps to avoid confusion about the "direct" nature of RNA sequencing. The text currently implies that no reverse transcription is required, but then references an ONT Reverse Transcription Adapter. Distinguish between a full-length cDNA synthesis step (not required) and the use of a poly(T)-containing adapter for sequencing library preparation.<br><br>Thank you for the suggestion. We have added amended the relevant paragraph in the Background section:<br><br>In contrast, Oxford Nanopore Technologies (ONT) direct RNA-sequencing is a simple approach for single-molecule RNA-sequencing which does not require reverse transcription (other than for RNA stabilization and improving sequencing output) or polymerase chain reaction (PCR) amplification, thus avoiding amplification bias and retaining the original base and base-modification information [35-39]. It is worth noting that the full-length cDNA synthesis step, while not required, is recommended and the library preparation method utilizes a poly(T)-containing adapter for sequencing. (lines 82-88)<br><br>Methods<br><br><br>Methods: ★★★★☆ (4/5)<br>The methods section has its strengths; the data sources and preparation (Sequins spiked into host RNA) are clearly described. Versions of tools are provided, enhancing |

reproducibility.

Areas for Improvement are statistical analysis, comparisons and tests, hardware and computation details, and understanding of run time differences.

All aspects of areas of improvement suggested by the reviewer have been addressed and improved in the newest version of the manuscript. We have modified the analysis to include calculation of mean absolute error (MAE), as well as averaging multiple reads using medians and position at which the probability density function achieves is maximum. We now include confidence intervals on all estimates which have been calculated via bootstrap resampling. Also, we added more details regarding computational details – including RAM and processors (Table 2).

Currently, the study models distributions as normal and uses mean and SD, but no normality tests or justification for these choices are presented. Consider performing normality tests or using nonparametric measures. Additionally, providing confidence intervals or other robust statistics (median, interquartile ranges) would clarify variability. For the comparisons and tests, the authors should explain why you chose root mean square error (RMSE) minimization and other metrics.
Could alternative tests, like Wilcoxon signed-rank tests or paired t-tests (Wilcocoxon: this non-parametric test is suitable for paired comparisons when the assumption of normality is not met. -useful to compare the predicted tail lengths from each tool against the expected lengths, especially if the data distribution is skewed.), be used to compare the distribution of tail-length estimates more rigorously?
Paired t-Test, because this test could be applied if the normality assumption holds, providing a straightforward way to assess whether the mean difference between predicted and expected values is statistically significant. (If so, justification should be provided for why or why not)

Our data is not normally distributed, it has a multi-modal distribution. We only use the term "normal" in the shape of the highest peak, so t-tests will not be feasible for comparing the predicted tail lengths vs expected tail lengths. We have now removed the part where we model the second peak as normal distribution, and RMSEs to alleviate any confusions. We have added confidence intervals, SDs, medians and maximum peak of the density distribution instead (Data S1, Figures 2a-b). We use bootstrap resampling to evaluate significance of one tool having smaller mean absolute error than another, thus avoiding use parametric approaches for significance testing.

There are some additional metrics to explore:
---Median Absolute Deviation (MAD): Consider adding MAD as it is robust to outliers and could complement RMSE to provide a better understanding of central tendencies and variability.
---Mean Absolute Error (MAE): MAE is another alternative that simplifies the interpretation by focusing solely on the magnitude of errors without squaring them, potentially offering more intuitive insights for readers.

We explored the MAE of each tool across windows of various sizes and explored the median or maximum peak values. The results have been added as Figures 2b and S7, as we believe this metric would be the most beneficial to the readers.

The authors should address testing for normality, explicitly stating whether normality tests were conducted on the data (e.g., Shapiro-Wilk or Kolmogorov-Smirnov tests). If normality is confirmed, justify the use of parametric tests like RMSE or t-tests. If not, justify why non-parametric tests (e.g., Wilcoxon) were not employed or discuss plans to include them in future studies.

We are no longer implementing the normal distribution strategy and therefore have not carried out the suggested normality tests.

Explain the choice of statistical methods over time by discussing how the choice of statistical tests aligns with the study's goals. For example, emphasize whether the focus was on understanding overall error distribution, tool consistency, or accuracy in predicting specific tail lengths.

Powered by Editorial Manager® and ProduXion Manager® from Aries Systems Corporation

We have implemented statistical methods to understand all of the above – overall error distribution, tool consistency and accuracy in predicting specific tail lengths. We believe that the maximum peak of density distribution and the MAE are most appropriate metrics that align with the study's goals.

The authors could use visual representations of error complementing the statistical tests with visual aids such as boxplots, violin plots, or Bland-Altman plots to illustrate the error distributions and discrepancies between predicted and actual tail lengths across tools.
(maybe supplementary)

We have added plots showing the Mean absolute error of all tools (all, R1 and R2) in Figures 2b and S7.

The authors should provide hardware and computational details like providing explicit details on the computational environment—CPU/GPU models, RAM, OS—for each tool's run. While the Git-hub read me suggests how to run the system, it lacks any details about system requirements.
Readers need this to understand runtime differences and attempt to replicate performance measurements.

We have updated the Github README to include details about the system requirements (e.g. Python version, OS). The suggested hardware and computational details have been added in Table 2.

The authors should consider tool parameterization and indicate if any specific parameters (beyond defaults) were used in tailfindr, nanopolish, Dorado, or BoostNano runs. If no changes were made from defaults, state this explicitly.

We have added a table in the Methods to add the parameters used for analysis for each of the tools we have discussed in our manuscript (Table 3).

Results

The result's strengths are that they are presented clearly, showing density distributions and discussing short-tail anomalies. The identification of Dorado as a preferred tool due to speed, integration, and conservative filtering is well-supported by the data. The study acknowledges that all tools achieve broadly similar accuracy, differing mainly in runtime and filtering criteria, which is a practical insight for users.

The results have areas for improvement:
Regrading the short-tail reads explanation, the authors attribute short (<10 nt) poly(A) tails to truncated transcripts or mis-priming. For this reason, it is suggested that the authors strengthen this discussion with additional evidence or reasoning. For instance, is there a correlation between read quality and short-tail length estimates? Do truncated reads consistently align to internal A-rich stretches?

We generated correlation plots between expected poly(A) length and average read quality per read and used Spearman's correlation analysis to investigate the relationship (Figure S8). There is indeed a weak positive correlation between poly(A) length and read quality in some datasets (e.g. dorado R2, BoostNano R1, Boostnano R2 and tailfindr R1 in Sequins). However, we have identified that the truncated reads generally have an average read quality score > 20 (Figure 3f), showing that poor read quality score is not the main cause of these truncated poly(A) tails. We have also included results to show that most truncated reads do not align to internal poly(A) stretches nor stretches with high poly(A) content (Figures 3d-e).

Multiple peaks in distributions: Some density plots (Figure 1) show multiple peaks or shoulder peaks.

Discuss potential reasons for these patterns. Are they related to tool-specific biases, read quality, or adapter/poly(T) truncation?

Powered by Editorial Manager® and ProduXion Manager® from Aries Systems Corporation

All tools in our study and all versions of these tools show either a multi-modal distribution or shoulder peaks (Figures 1a-c). This seems to indicate that this is not a tool-specific effect, except for BoostNano, which showed a clear bimodal distribution in R1 sequins and trimodal distribution in R2 sequins and eGFP datasets. We note that multiple peaks were more prominent in longer known poly(A) length RNA, and this was expected given that there is more chance for longer tails to become fragmented than shorter tails. Therefore, as the early peak presents with most tools, we believe this is a feature of the sample, instead of the tool. We discuss this in more detail on pages 5 and 12. We modelled the poly(A) length vs quality score and found a weak, positive correlation, suggesting that there is some correlation with read quality (Figure S8), however, this is unlikely to be the major reason for these patterns.

Application Context: The results focus on method performance, but it would help readers to understand how these differences might influence downstream tasks. For example, if a method overestimates poly(A) length slightly, how could this affect conclusions about RNA stability or differential tail-length analysis between experimental conditions?

We have added a paragraph regarding this in the first paragraph of the discussion section (lines 330-337).
"The implications of poly(A) estimation outputs can lead to different varied interpretations. If a method overestimates poly(A) tails, the researcher may overestimate other associated functions such as RNA stability, since longer poly(A) tails are commonly associated with greater stability. This is particularly detrimental when exploring differential polyadenylation, as even small changes in poly(A) tails may influence statistical tests. Thus, as a general, we recommend the following; should the researcher have specific expected tail lengths for their study, they should choose the most appropriate tool based on the results of this study or similar, with the use of maxpeaks to average over read sets (N>100). In more complex transcriptomes with wider variety of tail lengths, tailfindr or Dorado should be utilized."

Figures and tables:
Figure 1:

Clear density plots, but consider adding vertical lines at expected tail lengths (30 nt and 60 nt) to guide interpretation. Splitting the figure into separate panels for R1 and R2 or using insets might clarify multiple peaks.

We have added dotted vertical lines at expected tail lengths and split the figure into two separate panels for R1 and R2 as per the reviewer's recommendations (Figures 1a-b).

Figure 2:
The IGV snapshots are informative. Enhance interpretability by adding annotations (arrows or boxes) highlighting truncated vs. full-length reads. Increase font sizes for readability.

Thank you for the suggestions. Boxes to highlight truncated vs full-length reads were added to Figure 3A and we have generally increased the font sizes for readability.

Figure 3:

Useful comparison of reads filtered by Dorado but retained by BoostNano.
Add a brief note or labeling to indicate expected tail lengths.
Discuss possible reasons for Dorado's conservative filtering here or in the main text.

We have added a note to indicate the expected tail lengths in the figure legends. Dorado's conservative nature might be due to the tendency of the tool to base its estimation on searching for a low variability region near an anchor point, and if such regions are undefined, there could be an omission of the reads. This information has been added to the discussion section in the third paragraph.

Tables:
Provide definitions for abbreviations (nt, CPU, GPU) in captions. For Table 2, adding confidence intervals around the mean tail-length estimates would strengthen statistical

Powered by Editorial Manager® and ProduXion Manager® from Aries Systems Corporation

rigor. For Table 3, specify hardware details as recommended above.

The abbreviations have been defined in the table captions. We have added confidence intervals for medians and maxpeaks in Supplementary Data S1. We have added computation configurations used in Table 2 for reproducibility.

Grammar Mistakes and errors in the results section:
Results Section:
Sentence: "The four methods display a similar pattern in the density distribution, with a prominent normal-like peak near the expected poly(A) length, but also with a over-representation of shorter poly(A) tails, ranging at approximately ~0-10 nt (Figure 1)."
Issue: "a over-representation"
Correction: "an over-representation"

Sentence: "We expected that these shorter peaks were derived from either fragmentation of the transcript, mis-priming of internal poly(A) stretches or degradation of the poly(A) tails."
Issue: tense mismatch ("expected" vs. "were derived").
Correction: "We expect" -- "were derived", loses context and tense conformity-- therefore the sentence should be adjusted-
"We hypothesize that these shorter peaks are derived from either fragmentation of the transcript, mis-priming of internal poly(A) stretches, or degradation of the poly(A) tails."

Sentence: "Interestingly, upon investigating these earlier peaks, we found that Dorado excludes reads which are retained in the analysis by BoostNano, despite them being classified as passed reads (Figure 3)."
Issue: Ambiguous pronoun "them." (them could incorrectly identify three possible targets in the sentence)
Correction: "Interestingly, upon investigating these earlier peaks, we found that Dorado excludes reads retained in the analysis by BoostNano, even though these reads are classified as passed reads (Figure 3)."

Sentence: "Therefore, Dorado appears to be a more conservative approach than BoostNano."
Issue: No grammar issues, but the statement could be more precise.
Suggested improvement: "Thus, Dorado demonstrates a more conservative approach compared to BoostNano."

Sentence: "In order to determine which normal distribution fit the peak best, we found the parameters (mean, SD) which minimize the root mean square error between the candidate normal distribution and the density distribution for an interval of 10 nt to the right of the mode."
Issue: Verb tense consistency ("fit").
Correction: "To determine which normal distribution fits the peak best, ..."

Sentence: "The peaks also lose their normal-like behavior for larger values."
Issue: Could use a more formal tone. Correction: "The peaks also deviate from their normal-like behavior at larger values."

Sentence: "Next, we compared the computational time required by each method to predict the tail-length of 4000 reads."
Issue: Hyphenation of "tail-length."
Correction: "Next, we compared the computational time required by each method to predict the tail length of 4,000 reads."

We have revised all hyphenated versions of ‘tail-length’ to ‘tail length’.

Sentence: "BoostNano also offers the option of using the Application Programming Interface (API) call instead of the direct method, which omits the file copy implemented in the direct approach, reducing the run time to 8 m 8 s."
Here, the sentence is extremely overwritten which causes a lack of clarity.
Correction: "BoostNano offers an alternative API-based method, which skips the file

copy step of the direct approach, reducing the runtime to 8 minutes and 8 seconds."

Discussion

Discussion: ★★★☆☆ (3/5)
The discussion as its strengths as it correctly identifies that Dorado's advantages (speed, integration with basecalling) make it appealing as a default choice.
The authors acknowledge that all tools are within a similar accuracy range, suggesting the deciding factor may be speed or integration rather than raw performance differences.
HOWEVER- there are areas for improvement:

Further dissect the limitations of each tool. For example, BoostNano shows good SD but slightly off mean for R1; what does this mean for its use cases?
We have amended the approach for using metrics for determining accuracy – we have added medians, maxpeaks, SD, CI, MAE and raw differences instead of the mean. We have noted a length- and sample-dependency in our results, which we have discussed in the first paragraph of the discussion section. In the same paragraph we noted – "The implications of poly(A) estimation outputs can lead to different varied interpretations. If a method overestimates poly(A) tails, the researcher may overestimate other associated functions such as RNA stability, since longer poly(A) tails are commonly associated with greater stability. This is particularly detrimental when exploring differential polyadenylation, as even small changes in poly(A) tails may influence statistical tests. Thus, as a general, we recommend the following; should the researcher have specific expected tail lengths for their study, they should choose the most appropriate tool based on the results of this study or similar, with the use of maxpeaks to average over read sets (N>100). In more complex transcriptomes with wider variety of tail lengths, tailfindr or Dorado should be utilized."

Address the discrepancy between tailfindr, nanopolish, and Dorado in terms of how they define and detect poly(A) boundaries. Why does Dorado not evaluate start/end positions of poly(A) tails in event space, and how might this influence results?

The differences in determining poly(A) boundaries in each tool have now been described in the background section (lines 106-121). Apologies for the confusion, we meant that Dorado does not output start and end positions of the poly(A) tails, nor the signals, unlike the other tools. Dorado of course must calculate start and end positions for estimating poly(A) tails.
Include a brief discussion about how results might generalize to more complex transcriptomes. Real samples have varying GC content, fragment lengths, and potentially modified bases. A short commentary acknowledging these factors would show awareness that synthetic standards cannot capture the full complexity of natural RNA populations.

We added a brief discussion about the generalizability of our data in the paragraph regarding limitations of this study in the discussion section (page 13, lines 382-392).
For these reasons, it is suggested that the authors suggest future directions.
For instance, how could tool developers incorporate these findings to improve their methods? Could future benchmarking sets include a gradient of tail lengths to better understand length-specific biases?

A section of future directions for tool development and tool benchmarking has been added in the discussion section (lines 397-399).

Grammar Mistakes and errors in the discussion section:
Sentence: "BoostNano and tailfindr tools provided estimation of the starting and ending positions of the poly(A) tails in event space while this information was absent in Dorado outputs."
Issue: "provided estimation" should be "provide estimation" to align with present tense.
Correction: "BoostNano and tailfindr tools provide estimation of the starting and ending positions of the poly(A) tails in event space, while this information is absent in Dorado

outputs."

Sentence: "On the R1 dataset, BoostNano showed a tighter distribution with the smallest SD, but its peak was the furthest from the correct value."
The issue here is that the test results are still speaking about general truths leading to verb tense inconsistency; "showed" should match other verbs in the section.
Correction: "On the R1 dataset, BoostNano shows a tighter distribution with the smallest SD, but its peak is the furthest from the correct value."

Sentence: "tailfindr had the most accurate estimation but also the largest error interval."

The issue here is the verb tense mismatch; "had" should be consistent with present tense to show truth, not past truth.
Correction: "tailfindr has the most accurate estimation but also the largest error interval."

Sentence: "Furthermore, Boostnano is more lenient in keeping reads for poly(A) estimation than Dorado."

Issue: "Boostnano" capitalization error; it should be "BoostNano."
Correction: "Furthermore, BoostNano is more lenient in keeping reads for poly(A) estimation than Dorado."

Sentence: "Overall, our results suggest that the four tools investigated in this study - BoostNano, tailfindr, nanopolish and Dorado have similar performance with their accuracy varying from one dataset to the other, with a potential length bias."

Issue: Missing commas for clarity; replace "with their accuracy varying from one dataset to the other" for conciseness.
Correction: "Overall, our results suggest that the four tools investigated in this study—BoostNano, tailfindr, nanopolish, and Dorado—have similar performance, with accuracy varying across datasets and showing potential length bias."

Sentence: "Therefore, we expect Dorado to be implemented as the default method of poly(A) tail estimation in the near future, with the rapid estimation timeframe, comparable estimation lengths to other tools, conservative nature and the added benefit of ease of obtaining this information during basecalling."

There are several issues here including verbosity and lack of parallelism.
Correction: "Therefore, we expect Dorado to be implemented as the default method for poly(A) tail estimation, given its rapid estimation timeframe, comparable accuracy to other tools, conservative nature, and ease of integration with basecalling."

Sentence: "This work demonstrates the value of having access to synthetic RNA molecules with known poly(A) tail-lengths for validating the accuracy of poly(A) tail estimation algorithms."

Issue: The phrase "validating the accuracy of" could be simplified for readability.
Correction: "This work demonstrates the value of synthetic RNA molecules with known poly(A) tail lengths for validating poly(A) tail estimation algorithms."

Sentence: "As methods improve, we anticipate that these datasets will be valuable for assessing improvements in estimation of poly(A) tails."

Issue: "improvements in estimation of" is awkward.
Correction: "As methods improve, we anticipate that these datasets will be valuable for assessing advancements in poly(A) tail estimation."

References need to be added to accommodate the suggested material review, but existing references are good

We have added more relevant references to the manuscript to supplement our findings and the background/discussion.

Powered by Editorial Manager® and ProduXion Manager® from Aries Systems Corporation

NEEDS REVISION
Jesse Daniel Brown PD AASU

Note:

I previously reviewed this paper previously in Research Hub and you can read these comments via the Research Hub review page here: https://www.researchhub.com/paper/8634403/using-synthetic-rna-to-benchmark-polya-length-inference-from-direct-rna-sequencing/reviews#threadId=55398.

The original preprint linked to the Research Hub review is here: https://doi.org/10.1101/2024.10.25.620206

Reviewer #3: The manuscript presents interesting findings, but several points require clarification and further discussion to enhance transparency, statistical robustness, and reproducibility. Addressing the disdcrepancies in Figure 1, justifying the choice of statistical methods, and providing detailed computational configurations will significantly improve the manuscript. Based on the above stated, my recommendation it is by accepting for publication after a major revision.

Discrepancies in Figure 1 have been addressed in the first paragraph of section (“Performance evaluation between BoostNano, tailfindr, nanopolish and Dorado”). We utilized the maxpeak metric and bootstrap based p-values to understand the accuracy of each test and have discussed this in the manuscript (page 5, lines 158-171, page 10, lines 276-289). Detailed computational configurations have been listed in Table 2 as well as updated methods for enhancing reproducibility.

Regarding the criteria from GigaScience Journal for Technical Notes this technical note comply partially with technical improvement/utility, because while the study demonstrates that Dorado is the fastest and most conservative method, the novelty of technical improvement is somewhat limited because the tools themselves are not newly developed by the authors (exception of BoostNano). That said, the benchmarking study provides value for the research community by helping users choose the most appropriate tool for poly(A) tail-length estimation.

The reviewer declare that he does not have competing interests.

The abstract provides a concise summary of the study's aim, methodology, and key findings. However, it lacks sufficient emphasis on the broader implications of benchmarking poly(A) tail-length estimation for transcriptomics research. The abstract mentions the advantages of Dorado in terms of speed and accuracy but does not explicitly describe the limitations of other tools or potential use cases of these findings. Expanding the abstract to connect the results to downstream applications such as transcript stability analysis or isoform quantification could improve its utility.

We have added the following to the abstract to connect the results to downstream applications: “These results provide a reference for poly(A) tail length estimation analysis, aiding in improving our understanding of the transcriptome and the relationship between poly(A) tail length and other transcriptional mechanisms, including transcript stability or quantification.” (lines 32-34)

The language is clear and concise, with only minor grammatical errors. For instance: "We expect that the poly(A) tails shorter than 10 nt occur due to potential truncation…" could be rephrased as "We hypothesize that poly(A) tails shorter than 10 nt may result from truncation…". And the sentence "BoostNano also offers the option of using the Application Programming Interface (API) call instead of the direct method, which omits the file copy implemented in the direct approach, reducing the run time to 8 m 8 s." is verbose and could be simplified for readability.

The first sentence with grammatical errors has been amended (lines 281-282) and the

Powered by Editorial Manager® and ProduXion Manager® from Aries Systems Corporation

second sentence has been removed due to updates to the results and methodology of our study.

Introduction

The introduction effectively outlines the biological significance of polyadenylation and the technical challenges in poly(A) tail-length estimation. It provides a clear rationale for using synthetic RNA (Sequins) as a benchmark. However, the scope of the discussion is narrow, with limited exploration of how poly(A) length affects broader transcriptomics applications. Additionally, the references focus on tool-specific papers, but there is a gap in discussing recent advances in nanopore sequencing or polyadenylation biology. One reference could be referenced and that was not cited is Lima SA, Chipman LB, Nicholson AL, Chen YH, Yee BA, Yeo GW, Coller J, Pasquinelli AE. Short poly(A) tails are a conserved feature of highly expressed genes. Nat Struct Mol Biol. 2017 Dec;24(12):1057-1063. doi: 10.1038/nsmb.3499, this reference discusses the broader impact of poly(A) tail dynamics on mRNA decay and transcript stability, which could frame the importance of accurate estimation tools.

We have added this paper as a reference (Lima et al., 2017) as per the reviewer's suggestion in the second paragraph of the "Background" section (page 2). We have also expanded the Background to incorporate a more detailed review of the importance of studying polyadenylation dynamics, recent advances in Nanopore sequencing, and polyadenylation biology.

Methods

3.5/5.0
The methods section is well-detailed, with clear descriptions of dataset preparation, tool execution, and parameter usage. However, there is a lack of explanation regarding the statistical tests used to compare tool performances (e.g., root mean square error minimization).

We have now explained the use of maxpeak (which is the value at which the probability density function achieves its maximum) and MAE for comparing tool performances. (lines 506-514).

Additionally, the choice of gold-standard datasets (R1 and R2 Sequins) is appropriate, but the lack of diversity in tail lengths (only 30 and 60 nt) limits the generalizability of the findings. Including datasets with intermediate or extreme tail lengths could provide a more comprehensive evaluation. A discussion of potential batch effects (e.g., variability in RNA spiking or sequencing conditions) is also missing.

We have added extra datasets derived from eGFP RNA ranging from 10 nt to 150 nt poly(A) tails to improve our study. We believe that batch effects will not affect our study majorly as the same dataset was used across all tools, and our conclusions now stem from the aforementioned additional eGFP dataset from the developers of tailfindr, which adds additional confidence in our observations. However, we have added discussions about the limitation in the lack of sequencing replicates and its effect on more complex transcriptomes without a ground truth (page 13, lines 382-399).

Regarding the statistics used, the authors estimated the accuracy for each method using the mean and standard deviation. However, it is unclear why these metrics were chosen instead of alternatives such as the median and standard error of the mean. Further, the authors model the greatest peak as a normal distribution but do not clarify whether they tested for normality. To strengthen the statistical rigor, I suggest performing a normality test on the values to justify the choice of statistical measures and tests. The rationale for selecting the final statistical method should also be explained. Moreover regarding the statistics, the authors used a root mean square error (RMSE) approach to fit the normal distribution of predicted poly(A) tail lengths. This is appropriate for measuring deviations but fails to address biases in individual datasets. For instance, the difference in standard deviation (SD) between R1 and R2 sets suggests tool-specific variability that could affect downstream analyses. Incorporating additional tests, such as paired t-tests or nonparametric comparisons (e.g., Wilcoxon signed-rank test), could strengthen the statistical rigor.

Powered by Editorial Manager® and ProduXion Manager® from Aries Systems Corporation

We have now updated the metrics to include the median and the point at which the probability distribution achieves its maximum (maxpeak) where the latter metric proved to be a favorable method (page 7, Figure 2b). We no longer model the greatest peak as a normal distribution and have removed all instances of referring to this in the manuscript. We also replaced the RMSE approach with the MAE as per the reviewers' suggestions (Figure 2b). We employed bootstrap calculation on the difference between MAEs from different tools to obtain p-values (Data S3).

Finally, the methods section does not provide sufficient details about the computational configurations used (e.g., GPU or CPU specifications, processor type, RAM capacity). These details are critical for reproducibility. I recommend that the authors clearly specify the hardware and software configurations used for both GPU and CPU implementations.

We have added computation configurations as recommended in Table 2 for reproducibility.

Results

The results section is structured and includes sufficient detail to support the conclusions. The comparative analysis between tools is informative, with clear summaries in Table 2 and computational time results in Table 3. However, certain findings, such as the over-representation of short poly(A) tails (<10 nt), are not explored in depth. The analysis would benefit from a discussion of whether this phenomenon is due to RNA degradation or mispriming.

We have carried out additional analyses to explore the 10 nt stretch of sequence downstream of the 3' end of truncated reads and have identified that poly(A) tail degradation/truncation is the most prevalent reason for the short poly(A) tails. Mispriming and read quality were found only contribute to minimal number of reads (Figures 3d-f).

Additionally, while Dorado is identified as the preferred tool, the implications for specific use cases (e.g., low- versus high-throughput experiments) are not discussed.

Implications for specific use cases have been highlighted in the discussion section, and we have noted "while Dorado is identified as the preferred tool, the researcher may prefer accuracy over time, especially in the case of low-throughput datasets. In this case, other methods like tailfindr may be implemented according to specific contexts." (page 12, lines 350-352)

For the R1 test set, the expected mean is not clearly explained. The authors should include a detailed explanation of how this expected mean was derived, including the calculations or assumptions used.

The term "expected mean" was inappropriately used. This phrase was used to describe the known lengths of the Sequins (i.e. 30 nt for R1 and 60 nt for R2). This has been amended for clarity in the manuscript.

Figure 1: in Figure 1, some graphs (e.g., boostnano_R1, boostnano_R2, dorado_R2, tailfindr_R2, nanopolish_R2) display two density peaks, while others do not. It is unclear why this discrepancy exists. I recommend that the authors address this in the results section by explicitly identifying these observations and discussing possible reasons for the differences in the discussion section.

We have explicitly identified these observations in the first paragraph of section "Performance evaluation between BoostNano, tailfindr, nanopolish and Dorado", and discussed the reasons in the discussion section in the third paragraph.

Figure 2: the subset of reads visualized with IGV is useful but underexplored. Additional annotations highlighting the differences between truncated and full-length reads could improve interpretability.

Powered by Editorial Manager® and ProduXion Manager® from Aries Systems Corporation

| | |
|---|---|
| | Additional annotations to highlight the truncated and full-length reads were added to improve interpretability (Figure 3a).<br><br>Figure 3:  while the focus on discarded reads is relevant, the figure does not explore the functional consequences of these differences. Including histograms of read qualities or mapping scores could provide additional insights.<br><br>We have added violin plots of read qualities of the discarded reads as Figure 4b.<br><br>Table 1: the descriptions are adequate but could include more technical details about how each tool handles noise or variability in signal.<br><br>How each tool handles noise or variability in the signals depends on the way each model has been trained. This information is not described in detail in the current existing publicly available literature, and thus it is unfortunately difficult to describe in the manuscript. Therefore, we will omit this information in the resubmitted manuscript.<br><br>Table 2: the summary statistics are clear but lack confidence intervals for mean and SD, which would provide a better sense of variability.<br><br>We added medians and maxpeak data with confidence intervals in Data S1.<br><br>Table 3: the computational time analysis is thorough. However, it would be beneficial to provide details about hardware specifications (e.g., CPU/GPU model) for reproducibility as already suggested.<br><br>We have added computation configurations used in Table 2 for reproducibility.<br><br>Discussion<br><br>The discussion appropriately interprets the results, emphasizing Dorado's advantages in speed and accuracy. However, it misses an opportunity to provide deeper insights into the limitations of the tools evaluated, particularly BoostNano's tendency to overestimate shorter tails and tailfindr's larger error intervals. The potential impact of sequencing coverage and RNA integrity on poly(A) tail estimation is also underexplored. Besides that, in the discussion section, the authors do not address why the Dorado method does not evaluate the start and end of poly(A) tails in the event space. This omission may influence the observed differences in performance between Dorado, Boostnano, and Tailfindr. I suggest that the authors discuss this limitation and its potential impact on their findings, such as differences in processing speed.<br><br>We provide deeper insig... |
| **Additional Information:** | |
| **Question** | **Response** |
| Are you submitting this manuscript to a special series or article collection? | No |
| **Experimental design and statistics**<br><br>Full details of the experimental design and statistical methods used should be given in the Methods section, as detailed in our Minimum Standards Reporting Checklist. Information essential to interpreting the data presented should be made available in the figure legends. | Yes |

Powered by Editorial Manager® and ProduXion Manager® from Aries Systems Corporation

| | |
|---|---|
| Have you included all the information requested in your manuscript? | |
| **Resources**<br><br>A description of all resources used, including antibodies, cell lines, animals and software tools, with enough information to allow them to be uniquely identified, should be included in the Methods section. Authors are strongly encouraged to cite Research Resource Identifiers (RRIDs) for antibodies, model organisms and tools, where possible.<br><br>Have you included the information requested as detailed in our Minimum Standards Reporting Checklist? | Yes |
| **Availability of data and materials**<br><br>All datasets and code on which the conclusions of the paper rely must be either included in your submission or deposited in publicly available repositories (where available and ethically appropriate), referencing such data using a unique identifier in the references and in the "Availability of Data and Materials" section of your manuscript.<br><br>Have you have met the above requirement as detailed in our Minimum Standards Reporting Checklist? | Yes |

Manuscript
Click here to access/download;Manuscript;Boostnano paper_Apr 28.docx

# Using synthetic RNA to benchmark poly(A) length inference from direct RNA sequencing.

Jessie J-Y Chang[1], Xuan Yang[1], Haotian Teng[2], Jianshu Zhang[1], Benjamin Reames[1], Shuxin Zhang[1], Vincent Corbin[1]*, Lachlan Coin[1,3]*

1 Department of Microbiology and Immunology, University of Melbourne at The Peter Doherty Institute for Infection and Immunity, Melbourne, VIC, 3000, Australia

2 Ray and Stephanie Lane Computational Biology Department, School of Computer Science, Carnegie Mellon University, Pittsburgh, Pennsylvania, United States of America, 15213

3 Department of Clinical Pathology, University of Melbourne, Melbourne, VIC, 3000, Australia

*Corresponding authors: Vincent Corbin; Lachlan Coin

## Abstract

Polyadenylation is a dynamic process which is important in cellular physiology, which has implications in mRNA decay rates, translation efficiency, and isoform-specific regulation. Oxford Nanopore Technologies direct RNA-sequencing provides a strategy for sequencing the full-length RNA molecule and analysis of the transcriptome. Several tools are currently available for poly(A) tail length estimation, including well-established methods like *tailfindr* and *nanopolish*, as well as more recent deep learning models like *Dorado*. However, there has been limited benchmarking of the accuracy of these tools against gold-standard datasets. In this paper, we present our novel deep-learning poly(A) estimation tool – *BoostNano* and compare with three existing tools - *tailfindr*, *nanopolish* and *Dorado*. We evaluate the four poly(A) estimation tools, using two sets of synthetic *in vitro*-transcribed RNA standards with known poly(A) tail lengths - Sequin (30 or 60 nucleotides) and enhanced Green Fluorescent Protein (10-150 nucleotides) RNA. Analyzing datasets with known ground truth values is a valuable approach to measuring the accuracy of poly(A) length estimation. The tools demonstrated length- and sample-dependent performance, and accuracy was enhanced by averaging over multiple reads via estimation of the peak of the density distribution. Overall, *Dorado* is recommended as the preferred approach due to its relatively fast run times, low mean average error and ease of use with integration with base-calling. These results provide a reference for poly(A) tail length estimation analysis, aiding in improving our understanding of the transcriptome and the relationship between poly(A) tail length and other transcriptional mechanisms, including transcript stability or quantification.

## Keywords

Oxford Nanopore Technologies, poly(A) tail, estimation, segmentation, direct RNA-sequencing

## Findings

## Background

Polyadenylation is a co-/post-transcriptional process in which a string of adenine nucleotides is added to the 3’ of nascent messenger RNA (mRNA) molecules by enzymes such as polyadenylate (poly(A)) polymerases (PAPs) [1-3]. In eukaryotes, the polyadenylation process begins through the recognition of the poly(A) signal (PAS) situated within the 3’ untranslated region (UTR) of the mRNA [4]. This is a 6 nt sequence motif – commonly ‘AAUAAA’, located approximately 10-30 nt upstream of the poly(A) tail [5]. The polyadenylation process is mediated by the Cleavage and Specificity Factor (CSF) complex, which is made up of four major subunits – Specificity Factor (SF), Cleavage Stimulation Factor (CstF) and Cleavage Factors I and II (CFI & CFII). The SF recognizes the poly(A) signal and is required for specific cleavage and polyadenylation [6-10]. Additionally, CFI and CFII are required for accurate cleavage, and CstF enhances efficient cleavage at the poly(A) site and for a proportion of cases, PAP is required [8, 11]. PAP extends the poly(A) tail, stimulated by CSF and Poly(A) Binding Protein II (PABP II) within the nucleus [12, 13]. After the 5’ capping, splicing and polyadenylation, the mRNA is exported out of the nucleus into the cytoplasm. Here, the poly(A) tail is regulated by various deadenylase complexes – including CCR4-NOT [14] and PAN2-PAN3 [15]. Traditionally, the eukaryotic non-mitochondrial mRNA poly(A) tail has been regarded to be on average ~150-200 nt [16], which is more associated with the initial polyadenylation stages in the nucleus. With the involvement of deadenylation in the cytoplasm, the steady state of poly(A) tails has been identified to be shorter (~50-100 nt) [17, 18]. It has also been noted that non-adenine bases can be found within poly(A) tails as well as internal poly(A) sites [19].

Polyadenylation is thought to increase the stability of the mRNA molecule [20], assist in export of the molecule from the cell nucleus [21] and plays a role in RNA circularization, which may enhance efficient translation of cellular mRNAs [22]. This process is increasingly recognized as a dynamic process [23] which influences timing and degree of protein production [24, 25]. Furthermore, it is implicated in mRNA decay rates and regulation of gene expression [26, 27]. Poly(A) tails are also regarded to be dynamic in viral RNA, such as in the bovine coronavirus [23]. Currently, an ample number of studies have explored alternative polyadenylation (APA) [28-31] – the alternative usage of poly(A) sites which leads to variable 3’ ends of transcripts derived from the same gene. However, this mechanism is commonly confused with the study of poly(A) tail lengths and the latter is comparatively underexplored. As such, it is critical to be able to measure polyadenylation accurately using a high-throughput assay, which has the potential to enhance our understanding of poly(A) tail length and its connections to other transcriptional and translational mechanisms.

Most existing literature on measuring the poly(A) length have utilized techniques such as polymerase chain reaction (PCR) [23, 32], northern blotting [33] or short-read poly(A) tail measurements such as PAL-seq [34] or TAIL-seq [17], which have clear limitations in terms of breadth of whole-transcriptome-wide detection, lengths and also arduous experimental efforts. In contrast, Oxford Nanopore Technologies

(ONT) direct RNA-sequencing is a simple approach for single-molecule RNA-sequencing which does not require reverse transcription (other than for RNA stabilization and improving sequencing output) or PCR amplification, thus avoiding amplification bias and retaining the original base and base-modification information [35-39]. It is worth noting that the full-length cDNA synthesis step, while not required, is recommended and the library preparation method utilizes a polythymine (poly(T))-containing adapter for sequencing. Furthermore, full-length RNA molecules can be captured in one read, facilitating the identification of complex splicing patterns, RNA modifications and RNA secondary structures [40-45]. The Nanopore sequencer records changes in ionic current as RNA passes through the pore in a custom FAST5/POD5 file. This raw data is then converted into sequence data using a custom deep learning model, such as *Dorado* [46] or *Chiron* [47]. Although the majority of currently available public datasets have been generated from the SQK-RNA002 Direct RNA Sequencing kit, an updated version was released via early access in November 2023 (SQK-RNA004) as well as direct RNA-specific flow cells (FLO-MIN004RA or FLO-PRO004RA). The improvements include a faster motor protein, an RNA-specific reader pore, enhanced RNA models in the *Dorado* basecaller and an optimized library preparation method. Notably, recent iterations of *Dorado* have included the ability of RNA modification detection and poly(A) length estimations, which were only possible via third-party tools in previous years. Hence, Nanopore sequencing of native RNA provides an attractive approach for measuring single-molecule transcriptome-wide poly(A) tail length.

There have been several tools developed for estimating poly(A) tail length from raw Nanopore signal (**Table 1**), including *nanopolish* [48], *tailfindr* [42], *Dorado* (developed by ONT) [46] as well as our in-house tool *BoostNano* (details described in **Supplementary Information** and **Figures S1-3**, biotools:boostnano**,** RRID:SCR_026467) [47]. The tools detect the boundaries of poly(A) tails in varied ways. *tailfindr* identifies potential poly(A) stretches based on two rounds of defining the poly(A) tail segment, first determining rough poly(A) boundaries by thresholding the smoothened signal using a sliding window. Then, the second stage computes the mean of every 25 samples of clipped signal, and shrinks the rough poly(A) boundaries via confining the raw signal slopes. *nanopolish* utilizes a Hidden Markov Model (HMM), where each region of the read – the sequencing adapter, RTA, poly(A) tail and coding transcript each has one state contained by the HMM, in which these regions are linked sequentially, through linear-chain state transitions. Each section is deemed to have a unique emission distribution, which can be modeled by the HMM and applied on each read. *Dorado* utilizes a sliding window approach to find signal characteristics, initialized by identifying the RNA adapter sequence and determining the signal anchor point (i.e. the start of the poly(A) tail). The boundaries of the poly(A) tail are identified via analyzing around the proximity of the anchor point and understanding regions of the signal with low variance and similar mean values. *BoostNano* considers each region of the read as states like *nanopolish*. The neural network combines previous hidden states with current signal estimates to predict the signal's state, performing segmentation (more details can be found in **Supplementary Information**). However, there have been limited attempts to benchmark poly(A) tail length inference using gold-standard datasets with known poly(A) tail lengths, and comparisons between the two most-recent kit versions – RNA002 and RNA004.

**Table 1. Summary of each poly(A) tail estimation tool benchmarked in this study.**

| Tool | Description | Reference |
|---|---|---|
| *BoostNano* | Convolutional Neural Network (CNN)-Recurrent Neural Network (RNN)-Connection-ist Temporal Classification (CTC) architecture from *Chiron* basecaller used to find boundaries of poly(A) in raw signal, basecalling not required | Teng et al., 2018. [47] |
| *tailfindr* | *R* tool, which uses the unaligned raw FAST5 data to estimate the poly(A) lengths via using the raw signal slope to refine the boundaries of potential poly(A) stretches and normalization with the read-specific nucleotide translocation rate, basecalling required for obtaining basecalled FAST5 with Events/Move table | Krause et al., 2019 [42] |
| *nanopolish* | Utilizes a predictive model in which a hidden Markov model (HMM - performs segmentation of the raw sequencing signal) and an estimator of the translocation rate are combined, basecalling required for obtaining input FASTQ | Simpson et al., 2017 [48] |
| *Dorado* | Searches for the boundaries in the raw signal and estimates the poly(A) tail length by considering the samples/base information, with adjustment for overestimation of the poly(A) tail. Primarily a basecalling tool, incorporates the poly(A) tail estimation during the basecalling itself | Oxford Nanopore Technologies [46] |

For this study, we utilize two classes of ground-truth datasets derived from **1)** RNA Sequins – synthetic *in vitro*-transcribed (IVT) RNA, transcribed from an artificial chromosome which comprises 78 gene loci split into two classes, having either a 30 nucleotide (nt) (R1) or a 60 nt (R2) poly(A) tail (BioProject: PRJNA675370) [44, 49], and **2)** IVT RNA using enhanced Green Fluorescent Protein (eGFP) constructs with a wider range of poly(A) lengths (10, 30, 40, 60, 100 and 150 nts) from the authors of *tailfindr* (ENA Project: PRJEB31806) [42]. Thus, in this study, we compare the commonly-used poly(A) tail length estimation tools (*Dorado*, *tailfindr* and *nanopolish*) along with our own novel tool – *BoostNano* (released for the first time via this technical note) in hopes to understand and disseminate information to the wider community regarding the most appropriate tool for poly(A) length estimation.

## Performance evaluation between *BoostNano, tailfindr, nanopolish* and *Dorado*

To compare the estimation performance of *BoostNano*, *tailfindr* v1.4, *nanopolish* v0.13.3 and *Dorado* v0.9.0, we tested these tools on two Sequin testing sets with known poly(A) tail lengths: R1 set with 30 nt tails and R2 set with 60 nt tails, as well as eGFP synthetic RNA with poly(A) tails ranging (10-150 nt) (**Figures 1a-c & Data S1**) [49]. Firstly, to estimate the accuracy of each method, we visualized the

density distributions for each dataset (**Figures 1a-c**). The four methods displayed a similar pattern in the density distribution, with a prominent normal-like peak near the expected poly(A) length, but also with an over-representation of shorter poly(A) tails, ranging at approximately ~0-20 nt (**Figures 1a-b**). For R2 Sequins, we observed a clear multi-modal distribution in all tools, with a trimodal distribution with *BoostNano* (**Figure 1b**). In contrast, the R1 Sequin estimates presented with either a multi-modal distribution (*nanopolish* and *BoostNano)* or a shoulder peak adjacent to the main peak, which was least evident in RNA004 *Dorado* data (**Figure 1a**). In the eGFP datasets, the bimodal distributions appeared mostly in datasets with ≥40 nt poly(A) tails (**Figure 1c**). *BoostNano* showed trimodal distributions in 60 and 80 nt datasets, and with an extreme overestimation with the 10 nt dataset. These results suggest that as poly(A) length increases, the distribution becomes more likely to be multi-modal, making means and medians potentially misleading as measures of average poly(A) length.

We then attempted to obtain a single estimate of tail length from the distributions in **Figure 1** for each tool and each known tail length. We used two approaches, the first a simple median, which is understood to be robust to deviations from normality. However, given the multi-modal nature of the distributions, we also tried to estimate the value which maximized the probability density function, which we call 'maxpeak' (**Data S1**). We investigated the difference between these estimates and the known values (**Figure 2a**), revealing a tendency for tools to overestimate short tails, particularly for the eGFP dataset. This analysis also revealed that the max-peak approach provided more accurate estimates than the median approach. *Dorado* showed less length-dependent error than other methods, particularly combined with maxpeak estimation of tail length. We also observed that correlation between methods increased as the number of reads included in the maxpeak statistic increased (**Figures S4-S6**). We investigated the width of the main peak in the probability distribution function, by estimating its standard deviation from full width at half-maximum (**Figure 2a**). As expected, this width increased as the known tail length increased. *BoostNano* was observed to have the tightest peak (meaning that more reads have values close to the maxpeak value).

Next, we investigated the accuracy of each tool at the read and grouped-read level. For read sets with 10 or more reads, we utilized either the median or maxpeak to generate an averaged estimate, as above. Then we calculated the Mean Absolute Error (MAE) between the estimated and known lengths (**Figure 2a, Figure S7, Data S2**). We observed that averaging over reads can lead to substantial improvements in accuracy (plateauing at 100 reads), with maxpeak providing more accurate estimates than using the median. Out of curiosity, we estimated the poly(A) tails of the pre-basecalled eGFP FAST5 files pulled directly from ENA Project, which had been basecalled by the authors of *tailfindr* with *Albacore* v2.3.3. Upon comparison with the other tools, we noticed that this dataset revealed the lowest MAEs, despite being the oldest ONT basecaller tested (**Figure 2b**). However, when we applied the same approach to Sequin datasets, the MAE was the highest out of all tools in the *Albacore*-basecalled dataset. Using bootstrap resampling, we calculated confidence intervals for each of the approaches (**Figure 2b**). We also used this resampling procedure to calculate whether MAE differences between tools was statistically significant and found that most (but not all) differences are significant ($p \leq 4.11 \times 10^{-06}$), as can be observed from the confidence intervals (**Figure 2a, Data S3**).

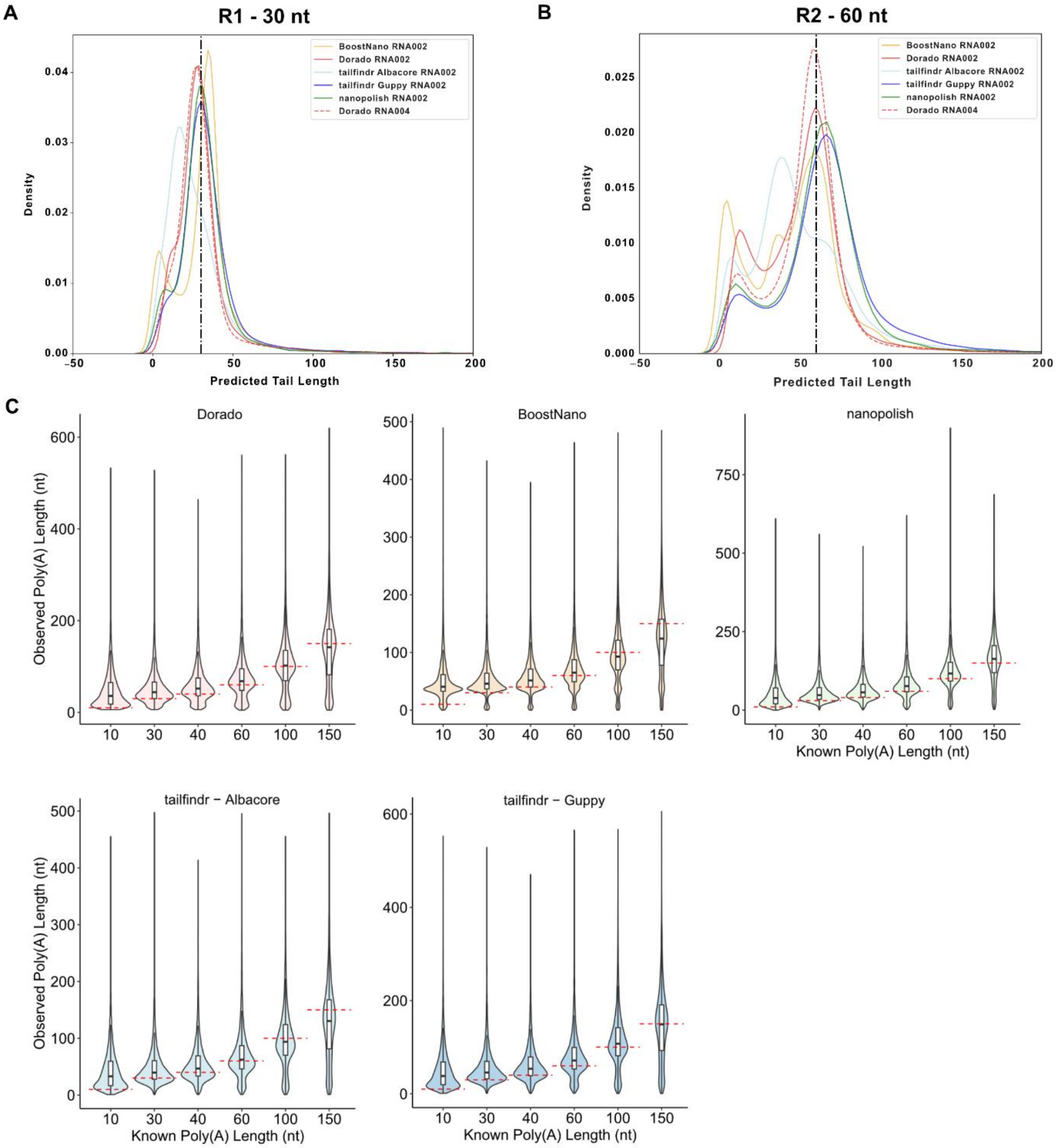


**Figure 1. Poly(A) tail length estimates for each tool. (A-B)** Predicted poly(A) tail length distributions for **A)** R1 (30 nt) and **B)** R2 (60 nt) Sequins. Outputs from *BoostNano* (yellow), *Dorado* (red), *tailfindr* (blue) and *nanopolish* (green). RNA002 (solid) and RNA004 (dashed) are shown as different line types. X-axis shows the predicted poly(A) tail lengths of all reads and Y-axis reveals the density of the poly(A) tail lengths. nt – nucleotide. **C)** Poly(A) tail estimates of eGFP synthetic RNA from the study by Krause et al. [42], ranging between 10-150 nt in poly(A) length.

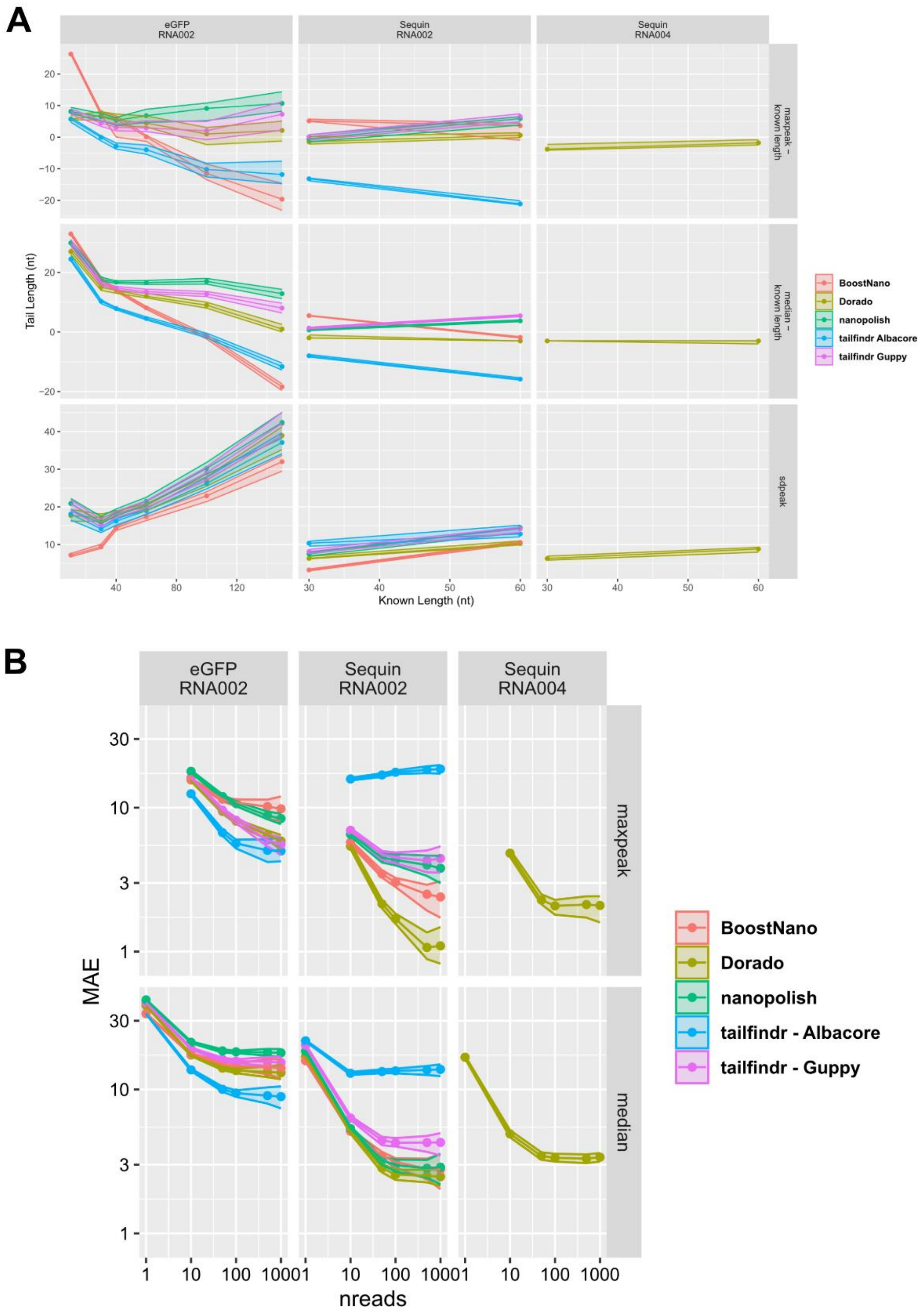


**Figure 2. Mean absolute error (MAE) and tail length differences compared with known lengths per dataset and tool. A)** Tail length differences in nt of maxpeak and medians compared with the known length across the full range of known lengths. X-axis represents the known length (10 nt – 150 nt for eGFP, 30 nt / 60 nt for Sequins) and Y-axis represents difference between estimated and known tail length (rows 1,2) or the estimated standard deviation of the peak (row 3). 95% confidence intervals are shown as ribbons. **B)** Mean absolute error of estimates of tail length across windows of nreads utilizing either maxpeak or median. The MAE is calculated as average of absolute difference to known lengths across all windows. 95% confidence intervals are also shown in ribbon form. X-axis represents the window size, and Y-axis represents the MAE.

To further test each method's ability to call poly(A) tails, we calculated the number of reads detected with the same number of input reads from the eGFP data, which were 592,571 reads (**Table S1**). We found that *BoostNano* detected the greatest number of reads and *nanopolish* detected the least number of reads in total, reads with poly(A) tails as well as reads aligned to the eGFP barcodes (maximum 96,403 reads), highlighting the high sensitivity of *BoostNano*.

We then proceeded to further understand the smaller peaks of the density distributions, which were present in almost all datasets (**Figures 1a-c**). This peak was more prominent at ~0-5 nt in *BoostNano*, whereas the early peaks for *tailfindr*, *nanopolish* and *Dorado* were positioned at ~5-20 nt. We hypothesized that these shorter peaks were derived from either **1)** fragmentation of the transcript, **2)** mis-priming of internal poly(A) stretches, or **3)** degradation of the poly(A) tails. To test this, we inspected reads with <10 nt poly(A) tails (as measured by *BoostNano*) and observed that the majority (~62.2%) aligned within 20 nt of the 3' end of the Sequin reference transcripts (**Figures 3a & b**). This suggested that most of these shorter poly(A) tails occurred due to hypothetical reason **3) -** fragmentation/degradation of the poly(A) tail, which is likely to be a sample integrity/preparation issue than an estimation defect. However, the remaining ~37.7% of reads showed truncations in the reference transcript (**Figure 3a**), consistent with hypothetical reasons **1)** fragmentation of the physical RNA or **2)** mis-priming. We wondered whether we could find any poly(A) stretches or high-adenine content in the sequences following the mapped 3' end of the truncated Sequin reads which would theoretically bind to the 10 poly(T)'s of the reverse transcription adapter (RTA) in the Direct RNA Sequencing kit. A high rate of these endings would correlate to high rates of mispriming (**Figure 3c**). To understand this phenomenon, we utilized the truncated dataset and isolated the 10 nt sequence following the end of the truncated Sequin reads according to the reference transcript. Then, we found the longest poly(A) stretch and the proportion of adenine bases in the 10 nt sequences. We observed that out of the truncated reads (3,088), only ~4.1% and ~1.6%, ~0.2%, ~0.03%, 0.03% of the reads had a poly(A) stretch of at least 4, 5, 6, 7, 8 adenines, respectively, with the longest poly(A) stretch being 8 adenines (**Figure 3d**). Furthermore, only ~4.73% of the reads contained high (> 50%) poly(A) content in the 10 nt downstream of their ends (**Figure 3e**). Therefore, we were able to determine that reason 2 (i.e. mispriming) was unlikely to be the main reason for the presence of these truncated transcripts with short poly(A) lengths. Finally, we isolated all reads which did not meet any of the criteria listed above (high poly(A) content/at least 4 adenine stretches in 10nt downstream of mapped end and mapped within 20 nt of the 3' end of reference Sequin transcript, ~93.8%), and examined their average read quality scores, as we thought this may contribute to the shorter poly(A) tail (**Figure 3f**). We observed that surprisingly, most of the reads (~97.9%) showed average quality scores of > 20, highlighting that poor read quality was not a prominent issue (**Figure 3f**). The overall Spearman correlation between poly(A) lengths and average read quality scores showed four datasets (*Dorado* R2, *BoostNano* R1, *BoostNano* R2, *tailfindr* R1) with weak but positive correlations ($r = 0.01$-$0.1$, $p < 0.05$) and *nanopolish* R2 dataset showing negative correlation ($r = -0.04$, $p = 6.6e$-$08$) (**Figure S8**). Therefore, we identified reads which were sequenced by direct RNA-sequencing which did not have proper poly(A) tails, nor mispriming events. Our analysis revealed that while all four poly(A) estimation methods consistently identified shorter poly(A) tails, *BoostNano* exhibited a narrower peak for these shorter tails, whereas *Dorado* tended to estimate longer poly(A) tails that were closer to the known values. (**Figures 3g-h**).

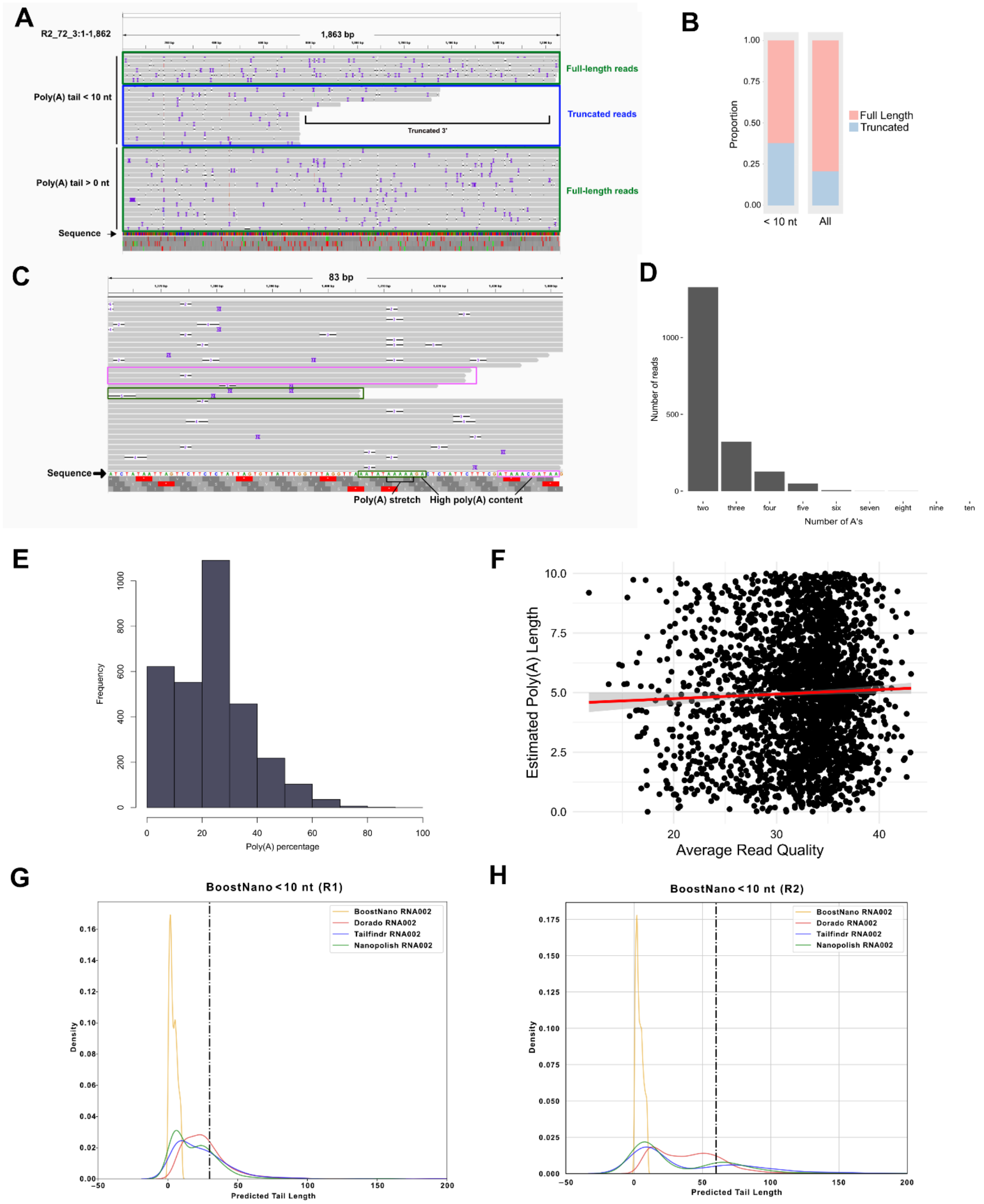


**Figure 3. Poly(A) tails with short estimations < 10 nt. A**) A representative subset of reads mapping to the R2_72_3 Sequin transcript visualized on the Integrative Genomics Viewer (IGV). The first subpanel shows a subset of reads with < 10 nt poly(A) tails (estimated by *BoostNano*), showing that reads with < 10 nt poly(A) tails are derived from both reads which have intact and fragmented 3' ends. The second panel shows a representative subset of full-length reads in the full dataset with any detected poly(A) lengths > 0 nt. Each grey line indicates a read. "Sequence" indicates the sequence of bases which form the transcript, where A = green, T = red, G = yellow and C = blue. nt – nucleotide. **B)** Proportion of reads with truncated vs full-length 3' ends in the entire combined Sequin RNA002 dataset and reads with poly(A) lengths < 10 nt (estimated by *BoostNano*). **C)** Truncated reads with poly(A) tails <10 nt (estimated by *BoostNano*) mapped to R2_65_1 Sequin transcript and 3' ends ending across an internal poly(A) stretch (green) and a stretch with high poly(A) content (pink). Each grey line indicates a read. "Sequence" indicates the sequence of bases which form the transcript, where A = green, T = red, G = yellow and C = blue. **D)** The number of adenines in the 10 nt stretch following the 3' end of truncated reads with < 10 nt poly(A) tails (estimated by *BoostNano*). **E)** Percentage of adenines in the 10 nt stretch following the 3' end of truncated reads with < 10 nt poly(A) tails (estimated by *BoostNano*). **F)** Average read quality scores vs poly(A) lengths in truncated reads with < 10 nt poly(A) tails (estimated by *BoostNano*), which do not meet thresholds of at least 4 adenines in series or 60 percent poly(A) content in the 10 nt following the 3' end of the mapped read. **(G-H)** Density plots of estimated read-lengths with < 10 nt poly(A) tails (estimated by *BoostNano*) in all four tools in the Sequin **G)** R1 and **H)** R2 datasets.

The explanations above partially explain the earlier peak (~0-20 nt) in the density distribution (**Figure 1**) in all four methods, however, *BoostNano* particularly showed the mode of the peak presenting at even shorter poly(A) tail lengths than *tailfindr*, *nanopolish* and *Dorado*. As the ONT reverse transcription adapter (RTA) used for reverse transcribing the native RNA strand has 10 poly(T) bases, it is likely that the minimum detection limit of poly(A) tails is 10 nt, which matches the ~10 nt peak with *tailfindr*, *nanopolish* and *Dorado*. We hypothesize that poly(A) tails shorter than 10 nt may result from truncation of the poly(T) stretch of the RTA. Interestingly, upon investigating these earlier peaks, we found that *Dorado* excludes reads retained in the analysis by *BoostNano*, even though the majority of these reads were considered to have high read quality (**Figures 4a & 4b**). While an earlier peak of <10 nt was the most prominent amongst reads discarded by *Dorado*, we also observed peaks of ~40 nt and ~60 nt (according to *BoostNano*) amongst reads discarded by *Dorado*. As mentioned above, *BoostNano* resulted in the greatest number of reads with detected poly(A) tails compared with the other three methods, including *Dorado* (**Table S1**). Thus, *Dorado* demonstrates a more conservative approach compared to *BoostNano*.

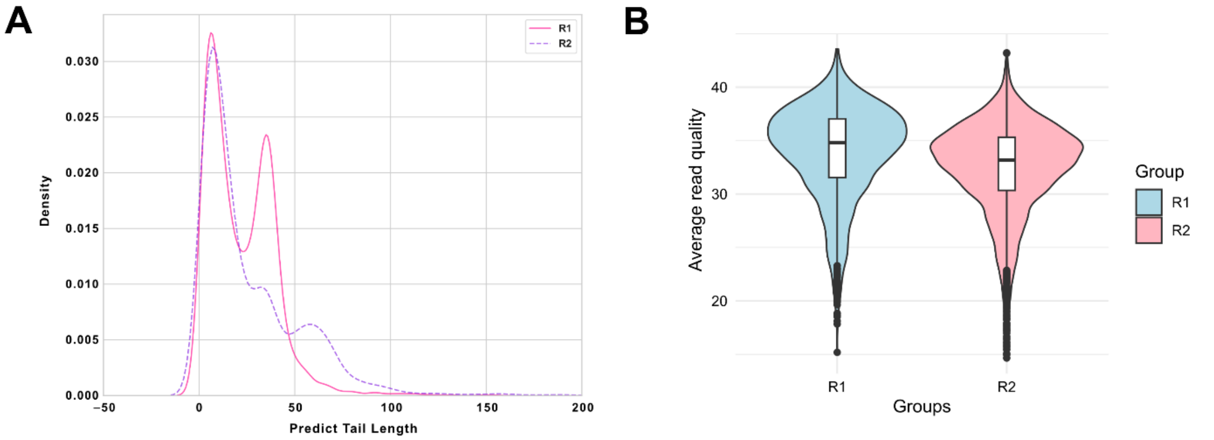


**Figure 4**. **Reads which have been filtered out by *Dorado* but retained in the *BoostNano* output. A)** R1 Sequin reads are indicated in a solid pink line (known length = 30 nt), and R2 Sequin reads are indicated in a dashed purple line (known length – 60 nt). X-axis shows the predicted poly(A) tail length in nucleotides, and the Y-axis shows the density distribution. nt – nucleotide. **B)** Violin plots of average read qualities of the same reads in R1 and R2 Sequin groups.

Finally, we compared the computational time required by each method to predict the tail lengths of 4,000 reads. For *BoostNano* and *Dorado*, we used one graphics processing unit (GPU) with 16G allocated RAM, while for *nanopolish* and *tailfindr*, which doesn't have the option to be run on GPU, we used one central processing unit (CPU) with 16G RAM and 1 thread for *nanopolish*. *Dorado* and showed rapid computational times at just ~1 m 10 seconds (s), whereas *BoostNano* revealed the longest computational time at ~16 m and 52 s (**Table 2**).

**Table 2. Computational time efficiency to process 4,000 reads with 1 GPU/CPU.** GPU - Graphics Processing Unit, CPU - Central Processing Unit, API – Application Programming Interface, s – seconds. Execution times include all pre-processing times such as basecalling and alignment.

| Method | Execution time (4000 reads, incl. basecalling, alignment) | GPU/CPU | GPU/CPU Model | Processor | RAM | OS |
|---|---|---|---|---|---|---|
| *BoostNano* | 16 m 52 s | 1 GPU | 80 GB A100 Nvidia GPU | Intel Xeon Gold | 16 G | Linux |
| *Dorado* | 1 m 10s | 1 GPU | 80 GB A100 Nvidia GPU | Intel Xeon Gold | 16 G | Linux |
| *tailfindr* | 10 m 56 s | 1 CPU | Intel Xeon Gold 6254 CPU @ 3.10GHz | Intel Xeon Gold | 16 G | Linux |
| *nanopolish* | 2 m 5 s | 1 CPU | Intel Xeons Gold 6254 CPU @ 3.10GHz | Intel Xeon Gold | 16 G | Linux |

# Discussion

In this technical note, we assessed the predictive performance of four poly(A) tail length estimation tools - *tailfindr, nanopolish, BoostNano* and *Dorado* on three separate testing sets with known poly(A) tail lengths. This was explored via various methods including manual visualization of density plots, calculating read-level and grouped read-level MAE, calculating standard deviation of the probability density function, exploring the sensitivity of detection and execution time. When evaluating poly(A) lengths at the gene-level or transcript-level, the researcher may wish to utilize an average value to compare between different conditions. While medians are typically preferred over means due to the commonly skewed distribution in naturally occurring poly(A) lengths, our results highlighted that utilizing the maxpeak approach is more useful than the medians for read-sets with 10 reads or more (**Figures 2a & S4**). When using this method, we noted length- and sample-dependent rankings for the evaluated tools (**Figure 2b**, **Data S1**). Overall, *tailfindr* and *Dorado* were most accurate across the different lengths, and *BoostNano* performed poorest, with the exception of 60 nt tails, where *BoostNano* and *Dorado* performed well (**Data S1**). Length-dependency of error rates is important to consider as polyadenylation research pertains mostly to mammalian (e.g. human) mRNA, and more recently with viral RNA (e.g. Severe Acute Respiratory Syndrome Coronavirus-2 (SARS-CoV-2)). In mammals, poly(A) tails on mRNA undergo an initial synthesis stage which increases to 100-200 nt, and once localized out of the nucleus, the poly(A) tail is subject to a deadenylation by CCR2-NOT and PAN2-PAN3 deandenylase complexes, in which the steady state average poly(A) length is approximately ~50-100 nt [17]. The exceptions are in mitochondrial RNA, which have average poly(A) lengths of ~40-50 nt [50]. Furthermore, poly(A) lengths on SARS-CoV-2 RNA have been found to be approximately 45-60 nt [40, 44]. The implications of poly(A) estimation outputs can lead to different varied interpretations. If a method overestimates poly(A) tails, the researcher may overestimate other associated functions such as RNA stability, since longer poly(A) tails are commonly associated with greater stability. This is particularly detrimental when exploring differential polyadenylation, as even small changes in poly(A) tails may influence statistical tests. Thus, as a general, we recommend the following; should the researcher have specific expected tail lengths for their study, they should choose the most appropriate tool

based on the results of this study or similar, with the use of maxpeaks to average over read sets (N>100). In more complex transcriptomes with wider variety of tail lengths, *tailfindr* or *Dorado* should be utilized.

Another factor which may be important to the researcher is time of execution. We observed that *Dorado* and *nanopolish* surpasses the rate of *BoostNano* or *tailfindr* (**Table 2**). *BoostNano* and *tailfindr* tools provide estimation of the starting and ending positions of the poly(A) tails in event space. In contrast, *nanopolish* shows only the start positions and this information is available from *Dorado* verbose logs. *nanopolish* and *tailfindr* require additional processing such as FAST5 basecalling and mapping, which also contribute to greater overall execution times. In light of these findings, we anticipate the adoption of *Dorado* as the default method for poly(A) tail estimation, given its rapid estimation timeframe, comparable accuracy to other tools, conservative nature, and ease of integration with basecalling. One thing to note is that the community has noted remarkable differences in the performance of poly(A) tail measurements depending on the version of *Dorado* utilized, especially with versions prior v0.5.3 having caused notable issues. Regardless, the current state of *Dorado* has favorable properties for the general user. We note that while *Dorado* is identified as the preferred tool, the researcher may prefer accuracy over time, especially in the case of low-throughput datasets. In this case, other methods like *tailfindr* may be implemented according to specific contexts.

The density distributions for the poly(A) lengths were clearly multi-nomial for most datasets, and this was more pronounced in longer poly(A) tail datasets (**Figures 1a-c**). This phenomenon can be explained by the fact that there is a greater possibility for fragmentation for longer poly(A) tails, which can cause shorter than expected tail length peaks. This has also been noted in the *tailfindr* publication, although the commentary was referencing the cDNA data, instead of the RNA data [42]. Secondly, the lower peaks were particularly minimal in *Dorado* Sequin R1 datasets, which may be explained by its conservative nature in filtering out certain reads which were retained in other tools (**Figure 4a**). *Dorado*'s conservative nature might be due to the tendency of the tool to base its estimation on searching for a low variability region near an anchor point, and if such regions are undefined, this may lead to an omission of the reads. In contrast, as *BoostNano* detected the greatest number of poly(A) tails compared to all tools, we may attribute its unusual trimodal distribution to this reason. Early peaks in the poly(A) length density distribution (defined as <10 nt as measured by *BoostNano)* comprised of ~68% of reads potentially affected by fragmentation/degradation of the poly(A) tail, and approximately 1/3 of these reads were deemed truncated and could not be accounted for by fragmentation/degradation of the poly(A) tail (**Figure 3b**). Upon further investigation, ~94% of these unaccounted reads were not attributed mispriming due to poly(A) stretches or poly(A)-rich regions in the 10 nt downstream of the 3' end of the read (**Figures 3d-e**). It is currently unclear to us how these reads were able to be sequenced if the reads supposedly lacked a poly(A) tail due to truncation in the middle of the read. If truncations did occur, the 5' end of the transcript as opposed to the 3' end would be lacking. We do not believe this is due to mapping issues as reads with high-quality mappings were isolated, and we utilized synthetic RNA with well-defined references. Although seemingly unlikely, these types of reads may only be sequenced if somehow the reads were able to enter the pores without the RTA or sequencing adapters. Therefore, we suspect

potential sequencing errors, or incorrect adapter ligation. Further work will be required to elucidate this phenomenon, but nevertheless, this emphasizes the importance of good RNA integrity of the input RNA.

Our study emphasizes the importance of obtaining sufficient coverage of each transcript in order to take advantage of improved polyA tail length estimation accuracy via averaging. We recommend obtaining at least 100x coverage of each target transcript to acquire a reliable estimate of poly(A) tail length via the maxpeak approach.

One of the limitations of this study is that we have only utilized synthetic RNA, for the purposes of procuring a ground truth. Researchers will aim to use poly(A) estimation tools for mainly real samples with more complex transcriptomes, including varying GC content, transcript and poly(A) lengths, modified bases, and coding potential unlike the synthetic RNA we explored in this study. From our understanding from previous studies regarding poly(A) lengths, we hypothesize that the poly(A) tail distribution will vary depending on these different factors. The results we reveal in this study helps understand biases observed via the use of different tools, in a controlled setting, without the effect of these variations. While this does not fully encapsulate the variability that may be seen with real samples, we hope that this data is useful for extrapolating which tool may suit the researcher best for their samples. Furthermore, generating a reliable ground truth dataset for real samples across the transcriptome is exceedingly difficult, if not unattainable. Secondly, we have employed a combined dataset of 7 separate sequencing runs containing Sequins as well as one eGFP dataset, with a lack of sequencing replicates. Potential batch effects may arise when visualizing complex transcriptomes with one sequencing replicate, which is common in many ONT direct RNA-sequencing studies. Thus, this study may be extended via the use of such replicates. Our study is limited to synthetic RNA which are limited in poly(A) length of 10 – 150 nt. Expanding the range of poly(A) lengths to better mimic the distribution in real samples, gaining an enhanced understanding of length-specific biases in each tool and including RNA from diverse preparation methods will enhance this study.

In conclusion, this work demonstrates the value of synthetic RNA molecules with known poly(A) tail lengths for validating poly(A) tail estimation algorithms. As methods improve, we anticipate that these datasets will be valuable for assessing advancements in poly(A) tail estimation. *Dorado* proves to be highly efficient and accurate amongst the four tools we explored in this study. Thus, we recommend the use of this approach when performing poly(A) length analyses via implementing the maxpeak values and window averaging strategy.

## Methods

### *Datasets*

### *RNA002*

This study utilized publicly available ONT direct RNA-sequencing datasets involving SARS-CoV-2-infected continuous cell lines (Vero, Calu-3 and Caco-2) derived from our previous study, with synthetic

RNA - Sequins (BioProject: PRJNA675370) [44]. Briefly, Vero (African green monkey kidney epithelia), Calu-3 (Human lung adenocarcinoma epithelia) and Caco-2 (Human colorectal adenocarcinoma epithelia) cells were cultured in 6-well tissue culture plates at 37°C, 5% (v/v) $CO_2$. The Australian ancestral strain of SARS-CoV-2 (SARS-CoV-2/human/AUS/VIC01/2020) was used to infect these cells at a multiplicity of infection (MOI) of 0.1 and the cells were harvested at 0, 2, 24 and 48 hours post-infection (hpi). The total RNA was extracted, treated with DNAse using Turbo DNA-free Kit (Invitrogen), and purified using the RNAClean XP magnetic beads (Beckman Coulter). 6 µg of total RNA for Vero cells and 3 µg of total RNA for Calu-3 and Caco-2 cells were pooled and 10% of expected mRNA (5% of total RNA) of Sequins [49] were added to each sample pool. The RNA was sequenced using the ONT Direct RNA Sequencing kit (SQK-RNA002), on R9.4.1 flow cells via the ONT MinION/GridION. For the purposes of this study, infected datasets from 24 and 48 hpi from all three cell lines and additionally 2 hpi from Vero cells were analyzed.

***RNA004***

Calu-3 cells were grown in 6-well tissue culture plates until 80-90% confluency and infected with 3 x Australian strains of Delta, Omicron (XBB1.5) and Omicron (JN.1) SARS-CoV-2 virus in triplicate. The infected cells were incubated at 37°C, 5% (v/v) $CO_2$ and harvested at 4 days post-infection (dpi). The total RNA was extracted using the RNeasy Mini Kit (Qiagen), treated with Turbo DNA-free Kit (Invitrogen) and purified using RNAClean XP beads (Beckman Coulter). For the sequencing, 1 µg of final total RNA product + 5% of expected mRNA (5% of total RNA) of Sequin mixA were used as inputs. 6 x samples in total were sequenced – 2 x Delta, 3 x XBB1.5 and 1 x JN1 samples. The new ONT Direct RNA Sequencing kit (SQK-RNA004) was used to sequence the libraries with the following modifications to the reverse transcription step; the use of the Induro Reverse Transcriptase (New England Biolabs) and incubation at 20 min at 55°C, then 10 min at 70°C. The libraries were sequenced using the kit-specific flow cells (FLO-MIN004RA, ONT) and sequenced using the ONT MinION/GridION via *MinKNOW* v24.02.16 and live-basecalled using the *MinKNOW*-integrated version of *Dorado* v7.3.11. The Sequin reads have been made available publicly on Figshare: https://figshare.com/s/89dbc444ebbd132e8958 (see 'Analysis' for methods, will be updated with public DOI after publication).

## *Analysis*

The minimum requirement for poly(A) estimation was FAST5 files. Firstly, *Dorado* v0.9.0, which is a basecaller itself, and *BoostNano*, based on the *Chiron* basecaller, utilized raw FAST5 files as inputs. *Dorado* poly(A) estimation was carried out during the basecalling with the '--estimate-poly-a' parameter. For *nanopolish* analyses, raw FAST5 files were used for the 'index' step, and *Dorado* v0.9.0 used to generate the FASTQ files for the poly(A) estimation step. *tailfindr* requires basecalled FAST5 files for analyses with basecall group information in the FAST5. *Guppy* v6.3.2 was utilized for generating the basecalled FAST5 files. *Dorado* basecalling was incompatible with *taifindr* analyses as basecalled FAST5 files were required for the analyses. We attempted to test *tailfindr* using FAST5 files converted from POD5 files, however, this generated empty results. All *Dorado* or *Guppy* basecalling was carried out using the basecalled via the 'rna002 70bps_hac@v3' model for the RNA002 datasets, and

‘rna004_130bps_sup@v5.1.0’ model for the RNA004 datasets. No sup basecalling was available for RNA002.

For RNA002 data, an initial isolation of Sequins read was carried out by using the live-basecalled FASTQ files merged (passed + failed) and mapped to the merged Ensembl GrCh38 human, SARS-CoV-2 (VIC01/Australia) and Sequin genomes using *minimap2* v2.26 via the parameters ‘-ax splice -un’. Using the read id’s, FAST5 files were isolated. For both RNA002 and RNA004 data, Sequins (R1/R2) were assigned to the reads by basecalling the FAST5 data with *Dorado* v0.9.0 and mapping the resulting FASTQ to the Sequin transcriptome with *minimap2* v2.26 with the parameters ‘-ax map-ont’. The reads were filtered with *Samtools* v1.16.1 ‘view’ function with the parameters ‘-h -F 2308 -q 20’. The Sequin POD5/FAST5 files were isolated based on this mapping. The isolated Sequin FAST5 datasets have been uploaded to Figshare: https://figshare.com/s/288b39a2a52c0d199462. The data was also additionally basecalled using *Albacore* v2.3.3, the first generation ONT basecaller. Interestingly, *Albacore* v2.3.3 did not have an RNA002-specific config file, so we utilized the RNA001 + FLO-MIN106 combination: r941_70bps_rna_linear.cfg in attempts to replicate the methods of the *tailfindr* publication.

For further analysis, we implemented IVT synthetic RNA002 datasets generated from eGFPs from the *tailfindr* publication, which is publicly available (ENA Project: PRJEB31806). FAST5 files which were downloaded directly were basecalled already via *Albacore* v2.3.3 by the *tailfindr* authors. We subsequently re-basecalled the data with *Dorado* v0.9.0 as well as *Guppy* v6.3.2 and carried out the rest of the analysis as per the Sequin analysis. For *nanopolish* analysis, the data was mapped to the pCS2 + eGFP genome from Addgene, using *minimap2* v2.26 with the parameters ‘-ax map-ont’. The reads downloaded were demultiplexed into different poly(A) tail lengths -- (10, 30, 40, 60, 100, 150nt) by using the *seqkit* v2.5.1 ‘grep’ function with parameters ‘-s –p $barcode –R 1:120 –m 1’ by searching for corresponding barcode sequences in the first 120 bp of the reads (one mismatching was allowed). Read IDs for demultiplexing have been added to Figshare: https://figshare.com/s/60a9ffda712545f66a66.

For poly(A) tail length estimations, *Dorado* v0.9.0, *BoostNano*, *tailfindr* v1.4 and *nanopolish* v0.13.3 were used with the parameters outlined in **Table 3** and full scripts have been uploaded to Figshare: https://figshare.com/s/89dbc444ebbd132e8958 (will be updated with public DOI after publication).

**Table 3. Parameters for analysis.**

| Tool | Version | Parameters | Default (Y/N) |
|---|---|---|---|
| ***Dorado*** | 0.9.0 | **Model:** rna004_130bps_sup@v5.1.0 for R004 rna002_70bps_hac@v3 for R002<br><br>--estimate-poly-a | **Y** |

| | | | |
|---|---|---|---|
| | | Default: --poly-a-config | |
| ***BoostNano*** | N/A | **-i $1 -o $2 -m path/to/model --replace** | **Y** |
| ***tailfindr*** | 1.4 | **find_tails(fast5_dir = args[1], save_dir = args[2], csv_filename = "tails.csv", num_cores = 30, basecall_group=args[4])** | **Y** |
| ***nanopolish*** | 0.13.3 | **--reads=${FASTQ} --bam=${BAM} --genome=${REF} --threads=8** | **Y** |

Poly(A) truncations were visualized with *Integrative Genomics Viewer (IGV)* v2.10.1 using data with < 10 nt poly(A) tails according to *BoostNano* and all mapped data. All poly(A) tail truncation investigations were carried out in *R* v4.4.0 and density plots using *Python* v3.10.4.

Method timings were carried out using a subset of 4,000 reads, derived from Vero 2 hpi datasets, which was used for all timings. Basecalling and poly(A) tail times were added for the overall execution time. For *tailfindr* and *nanopolish*, which requires basecalled FAST5 files and FASTQ files, respectively, *Guppy* v6.3.2 was utilized.

Maxpeak was calculated as the value at which the probability density distribution achieves its maximum value. The density function was estimated using the density function from the stats package in *R*. Maxpeak and medians were calculated using only reads which were able to be detected by all methods to ensure direct comparisons.

The MAE was calculated by calculating either the median or maxpeak of reads in each on-overlapping window of N reads and finding the absolute difference between this median and the ground truth value. Then, the sum of these absolute values was divided by the number of windows. Only reads which were able to be detected by all tools were retained in the analysis. Bootstrapped T-tests were implemented to compare the accuracy of each tool and were carried out by re-sampling reads 1,000 times from the original dataset at random within each known length category (with replacement). Due to this finite number of resamples, the smallest possible non-zero p-value is 0.001. Therefore, any reported p-value of 0 should be interpreted as $p < 0.001$, indicating that the observed effect was consistent across all bootstrap iterations.

**Supplementary files**

**Supplementary Information.** Supplementary Information, Figures S1-8, Table S1.

**Supplementary Data S1.** General table of statistical metrics including medians, maxpeak, confidence intervals, and number of reads detected.

**Supplementary Data S2.** MAE results based on medians or maxpeaks for Sequin (RNA002, RNA004) and eGFP datasets per tool, averaged over window sizes N = 1, 10, 50, 100, 500, 1000.

**Supplementary Data S3.** Bootstrapped T-test results comparing MAE of each tool averaged over window sizes N = 1, 10, 50, 100, 500, 1000.

## Availability of supporting source code and requirements

Project name: BoostNano

Project home page: https://github.com/haotianteng/BoostNano

Biotools: boostnano

RRID: SCR_026467

Operating system(s): Platform independent

- Programming language: Python
- Other requirements: Pytorch
- License: Mozilla Public License, v. 2.0

## Data Availability

The datasets supporting the results of this article are available in the NCBI repository, RNA002 - BioProject: PRJNA675370 and RNA004 – Figshare: https://figshare.com/s/89dbc444ebbd132e8958.

## Declarations

## List of abbreviations

Poly(A) - Polyadenylate

ONT – Oxford Nanopore Technology

PCR – Polymerase Chain Reaction

HMM – Hidden Markov Model

CNN - Convolutional Neural Network

RNN - Recurrent Neural Network

CTC - Connection-ist Temporal Classification

SD – Standard deviation

m – minutes

s – seconds

MOI – Multiplicity of Infection

hpi – Hours post-infection

UTR – untranslated region

MAE – Mean average error

mRNA – messenger RNA

IVT – *in vitro* transcribed

eGFP – enhanced Green Fluorescent Protein

## Ethics approval and consent to participate

Not applicable.

## Consent for publication

Not applicable.

## Competing interests

LC has received funding from ONT unrelated to this work, as well as travel funding, also unrelated to this work.

## Funding

This work was supported by a NHMRC-EU project grant (GNT1195743) to LC.

## Authors' contributions

Conceptualization – J.J.-Y.C., H.T., V.C., L.C.

Methodology – H.T., V.C., L.C.

Software – H.T., V.C., L.C.

Validation - J.J.-Y.C., H.T., X.Y., V.C., L.C.

Formal analysis – J.J.-Y.C., X.Y., H.T., J.Z., B.R., V.C., L.C.

Investigation - J.J.-Y.C., X.Y., H.T., J.Z., B.R., S.Z., V.C., L.C.

Resources - J.J.-Y.C., H.T., V.C., L.C.

Data Curation - J.J.-Y.C., V.C., L.C.

Writing - Original Draft - J.J.-Y.C., X.Y., H.T., V.C., L.C.

Writing - Review & Editing - J.J.-Y.C., X.Y., H.T., J.Z., V.C., L.C.

Visualization – J.J.-Y.C., X.Y., H.T., J.Z., V.C., L.C.

Supervision – V.C., L.C.

Project administration – J.J.-Y.C., V.C., L.C.

Funding acquisition – V.C., L.C.

## Acknowledgements

This research was supported by The University of Melbourne's Research Computing Services and the Petascale Campus Initiative.

## Authors' information

Not applicable.

## References

1. Darnell JE, Wall R and Tushinski RJ. An Adenylic Acid-Rich Sequence in Messenger RNA of HeLa Cells and Its Possible Relationship to Reiterated Sites in DNA. 1971;68 6:1321-5. doi:10.1073/pnas.68.6.1321.
2. Lee SY, Mendecki J and Brawerman G. A Polynucleotide Segment Rich in Adenylic Acid in the Rapidly-Labeled Polyribosomal RNA Component of Mouse Sarcoma 180 Ascites Cells. 1971;68 6:1331-5. doi:10.1073/pnas.68.6.1331.

3. Terns MP and Jacob ST. Role of poly(A) polymerase in the cleavage and polyadenylation of mRNA precursor. 1989;9 4:1435-44. doi:10.1128/mcb.9.4.1435.
4. Proudfoot NJ and Longley JI. The 3' terminal sequences of human alpha and beta globin messenger RNAs: comparison with rabbit globin messenger RNA. Cell. 1976;9 4 PT 2:733-46. doi:10.1016/0092-8674(76)90137-9.
5. Proudfoot NJ and Brownlee GG. 3′ Non-coding region sequences in eukaryotic messenger RNA. Nature. 1976;263 5574:211-4. doi:10.1038/263211a0.
6. Bardwell VJ, Wickens M, Bienroth S, Keller W, Sproat BS and Lamond AI. Site-directed ribose methylation identifies 2'-OH groups in polyadenylation substrates critical for AAUAAA recognition and poly(A) addition. Cell. 1991;65 1:125-33. doi:10.1016/0092-8674(91)90414-t.
7. Keller W, Bienroth S, Lang KM and Christofori G. Cleavage and polyadenylation factor CPF specifically interacts with the pre-mRNA 3′ processing signal AAUAAA. The EMBO Journal. 1991;10 13:4241-9. doi:10.1002/j.1460-2075.1991.tb05002.x.
8. Wilusz J, Shenk T, Takagaki Y and Manley JL. A multicomponent complex is required for the AAUAAA-dependent cross-linking of a 64-kilodalton protein to polyadenylation substrates. Molecular and Cellular Biology. 1990;10 3:1244-8. doi:10.1128/mcb.10.3.1244.
9. Christofori G and Keller W. 3' cleavage and polyadenylation of mRNA precursors in vitro requires a poly(A) polymerase, a cleavage factor, and a snRNP. Cell. 1988;54 6:875-89. doi:10.1016/s0092-8674(88)91263-9.
10. Gilmartin GM and Nevins JR. An ordered pathway of assembly of components required for polyadenylation site recognition and processing. Genes & Development. 1989;3 12b:2180-90. doi:10.1101/gad.3.12b.2180.
11. Takagaki Y, Ryner LC and Manley JL. Four factors are required for 3'-end cleavage of pre-mRNAs. Genes & Development. 1989;3 11:1711-24. doi:10.1101/gad.3.11.1711.
12. Wahle E. A novel poly(A)-binding protein acts as a specificity factor in the second phase of messenger RNA polyadenylation. Cell. 1991;66 4:759-68. doi:10.1016/0092-8674(91)90119-j.
13. Winters MA and Edmonds M. A poly(A) polymerase from calf thymus. Characterization of the reaction product and the primer requirement. J Biol Chem. 1973;248 13:4763-8.
14. Lau N-C, Kolkman A, Schaik V, A., M., Frederik, Mulder W, Klaas, Pijnappel P, M., W., W., Heck R, J., Albert, et al. Human Ccr4–Not complexes contain variable deadenylase subunits. Biochemical Journal. 2009;422 3:443-53. doi:10.1042/bj20090500.
15. Wolf J, Valkov E, Allen MD, Meineke B, Gordiyenko Y, Mclaughlin SH, et al. Structural basis for Pan3 binding to Pan2 and its function in mRNA recruitment and deadenylation. The EMBO Journal. 2014;33 14:1514-26. doi:10.15252/embj.201488373.
16. Edmonds M, Vaughan MH and Nakazato H. Polyadenylic Acid Sequences in the Heterogeneous Nuclear RNA and Rapidly-Labeled Polyribosomal RNA of HeLa Cells: Possible Evidence for a Precursor Relationship. 1971;68 6:1336-40. doi:10.1073/pnas.68.6.1336.
17. Chang H, Lim J, Ha M and Kim N, V. TAIL-seq: Genome-wide Determination of Poly(A) Tail Length and 3′ End Modifications. Molecular Cell. 2014;53 6:1044-52. doi:10.1016/j.molcel.2014.02.007.
18. Eisen TJ, Eichhorn SW, Subtelny AO, Lin KS, McGeary SE, Gupta S, et al. The Dynamics of Cytoplasmic mRNA Metabolism. Mol Cell. 2020;77 4:786-99 e10. doi:10.1016/j.molcel.2019.12.005.
19. Begik O, Diensthuber G, Liu H, Delgado-Tejedor A, Kontur C, Niazi AM, et al. Nano3P-seq: transcriptome-wide analysis of gene expression and tail dynamics using end-capture nanopore cDNA sequencing. Nature Methods. 2023;20 1:75-85. doi:10.1038/s41592-022-01714-w.
20. Beckel-Mitchener AC. Poly(A) Tail Length-dependent Stabilization of GAP-43 mRNA by the RNA-binding Protein HuD. The Journal of Biological Chemistry. 2002;277 31:27996-8002. doi:10.1074/jbc.m201982200.

21. Fuke H and Ohno M. Role of poly (A) tail as an identity element for mRNA nuclear export. Nucleic Acids Research. 2007;36 3:1037-49. doi:10.1093/nar/gkm1120.
22. Gallie DR. The cap and poly(A) tail function synergistically to regulate mRNA translational efficiency. Genes Dev. 1991;5 11:2108-16. doi:10.1101/gad.5.11.2108.
23. Wu H-Y, Ke T-Y, Liao W-Y and Chang N-Y. Regulation of Coronaviral Poly(A) Tail Length during Infection. PLoS ONE. 2013;8 7:e70548. doi:10.1371/journal.pone.0070548.
24. Kojima S, Sher-Chen EL and Green CB. Circadian control of mRNA polyadenylation dynamics regulates rhythmic protein expression. Genes & Development. 2012;26 24:2724-36. doi:10.1101/gad.208306.112.
25. Biziaev N, Shuvalov A, Salman A, Egorova T, Shuvalova E and Alkalaeva E. The impact of mRNA poly(A) tail length on eukaryotic translation stages. Nucleic Acids Research. 2024;52 13:7792-808. doi:10.1093/nar/gkae510.
26. Passmore LA and Coller J. Roles of mRNA poly(A) tails in regulation of eukaryotic gene expression. Nature Reviews Molecular Cell Biology. 2022;23 2:93-106. doi:10.1038/s41580-021-00417-y.
27. Lima SA, Chipman LB, Nicholson AL, Chen Y-H, Yee BA, Yeo GW, et al. Short poly(A) tails are a conserved feature of highly expressed genes. Nature Structural & Molecular Biology. 2017;24 12:1057-63. doi:10.1038/nsmb.3499.
28. Mayr C and Bartel DP. Widespread Shortening of 3′UTRs by Alternative Cleavage and Polyadenylation Activates Oncogenes in Cancer Cells. Cell. 2009;138 4:673-84. doi:10.1016/j.cell.2009.06.016.
29. Huang G, Huang S, Wang R, Yan X, Li Y, Feng Y, et al. Dynamic Regulation of Tandem 3' Untranslated Regions in Zebrafish Spleen Cells during Immune Response. 2016;196 2:715-25. doi:10.4049/jimmunol.1500847.
30. Melamed ZE, López-Erauskin J, Baughn MW, Zhang O, Drenner K, Sun Y, et al. Premature polyadenylation-mediated loss of stathmin-2 is a hallmark of TDP-43-dependent neurodegeneration. Nature Neuroscience. 2019;22 2:180-90. doi:10.1038/s41593-018-0293-z.
31. Rund D, Dowling C, Najjar K, Rachmilewitz EA, Kazazian HH and Oppenheim A. Two mutations in the beta-globin polyadenylylation signal reveal extended transcripts and new RNA polyadenylylation sites. 1992;89 10:4324-8. doi:10.1073/pnas.89.10.4324.
32. Shien J-H, Su Y-D and Wu H-Y. Regulation of coronaviral poly(A) tail length during infection is not coronavirus species- or host cell-specific. Virus Genes. 2014;49 3:383-92. doi:10.1007/s11262-014-1103-7.
33. Salles FJ, Richards WG and Strickland S. Assaying the polyadenylation state of mRNAs. Methods. 1999;17 1:38-45. doi:10.1006/meth.1998.0705.
34. Subtelny AO, Eichhorn SW, Chen GR, Sive H and Bartel DP. Poly(A)-tail profiling reveals an embryonic switch in translational control. Nature. 2014;508 7494:66-71. doi:10.1038/nature13007.
35. Garalde DR, Snell EA, Jachimowicz D, Sipos B, Lloyd JH, Bruce M, et al. Highly parallel direct RNA sequencing on an array of nanopores. Nature Methods. 2018;15 3:201-6. doi:10.1038/nmeth.4577.
36. Wan YK, Hendra C, Pratanwanich PN and Göke J. Beyond sequencing: machine learning algorithms extract biology hidden in Nanopore signal data. Trends in Genetics. 2022;38 3:246-57. doi:10.1016/j.tig.2021.09.001.
37. Brouze A, Krawczyk PS, Dziembowski A and Mroczek S. Measuring the tail: Methods for poly(A) tail profiling. WIREs RNA. 2023;14 1 doi:10.1002/wrna.1737.
38. Rand AC, Jain M, Eizenga JM, Musselman-Brown A, Olsen HE, Akeson M, et al. Mapping DNA methylation with high-throughput nanopore sequencing. Nature Methods. 2017;14 4:411-3. doi:10.1038/nmeth.4189.

39. Silverman JD, Bloom RJ, Jiang S, Durand HK, Dallow E, Mukherjee S, et al. Measuring and mitigating PCR bias in microbiota datasets. PLOS Computational Biology. 2021;17 7:e1009113. doi:10.1371/journal.pcbi.1009113.
40. Kim D, Lee J-Y, Yang J-S, Kim JW, Kim VN and Chang H. The Architecture of SARS-CoV-2 Transcriptome. Cell. 2020;181 4:914-21.e10. doi:10.1016/j.cell.2020.04.011.
41. de Jong LC, Cree S, Lattimore V, Wiggins GAR, Spurdle AB, kConFab I, et al. Nanopore sequencing of full-length BRCA1 mRNA transcripts reveals co-occurrence of known exon skipping events. Breast Cancer Res. 2017;19 1:127. doi:10.1186/s13058-017-0919-1.
42. Krause M, Niazi AM, Labun K, Torres Cleuren YN, Muller FS and Valen E. tailfindr: alignment-free poly(A) length measurement for Oxford Nanopore RNA and DNA sequencing. RNA. 2019;25 10:1229-41. doi:10.1261/rna.071332.119.
43. Leger A, Amaral PP, Pandolfini L, Capitanchik C, Capraro F, Miano V, et al. RNA modifications detection by comparative Nanopore direct RNA sequencing. Nature Communications. 2021;12 1 doi:10.1038/s41467-021-27393-3.
44. Chang JJ, Rawlinson D, Pitt ME, Taiaroa G, Gleeson J, Zhou C, et al. Transcriptional and epi-transcriptional dynamics of SARS-CoV-2 during cellular infection. Cell Rep. 2021;35 6:109108. doi:10.1016/j.celrep.2021.109108.
45. Aw JGA, Lim SW, Wang JX, Lambert FRP, Tan WT, Shen Y, et al. Determination of isoform-specific RNA structure with nanopore long reads. Nature Biotechnology. 2021;39 3:336-46. doi:10.1038/s41587-020-0712-z.
46. Oxford Nanopore Technologies. Dorado (PolyACalculator).
47. Teng H, Cao MD, Hall MB, Duarte T, Wang S and Coin LJM. Chiron: translating nanopore raw signal directly into nucleotide sequence using deep learning. GigaScience. 2018;7 5 doi:10.1093/gigascience/giy037.
48. Simpson JT, Workman RE, Zuzarte PC, David M, Dursi LJ and Timp W. Detecting DNA cytosine methylation using nanopore sequencing. Nature Methods. 2017;14 4:407-10. doi:10.1038/nmeth.4184.
49. Hardwick SA, Chen WY, Wong T, Deveson IW, Blackburn J, Andersen SB, et al. Spliced synthetic genes as internal controls in RNA sequencing experiments. Nature Methods. 2016;13 9:792-8. doi:10.1038/nmeth.3958.
50. Chang JJ-Y, Gleeson J, Rawlinson D, De Paoli-Iseppi R, Zhou C, Mordant FL, et al. Long-Read RNA Sequencing Identifies Polyadenylation Elongation and Differential Transcript Usage of Host Transcripts During SARS-CoV-2 In Vitro Infection. Front Immunol. 2022;13 doi:10.3389/fimmu.2022.832223.

Figure 1

Click here to access/download;Figure;Figure1.tiff

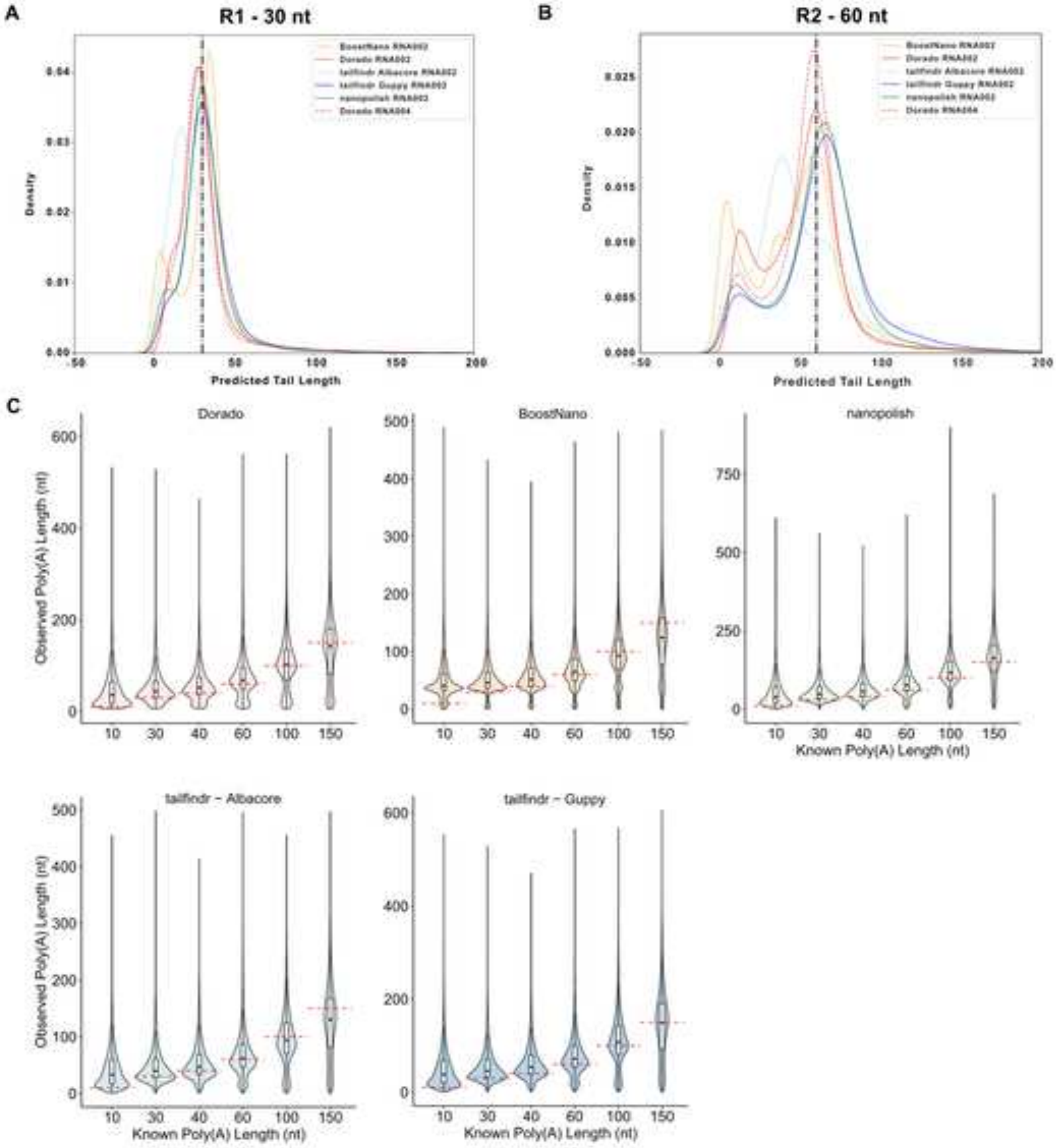

Figure 2 Click here to access/download;Figure;Figure 2.tiff

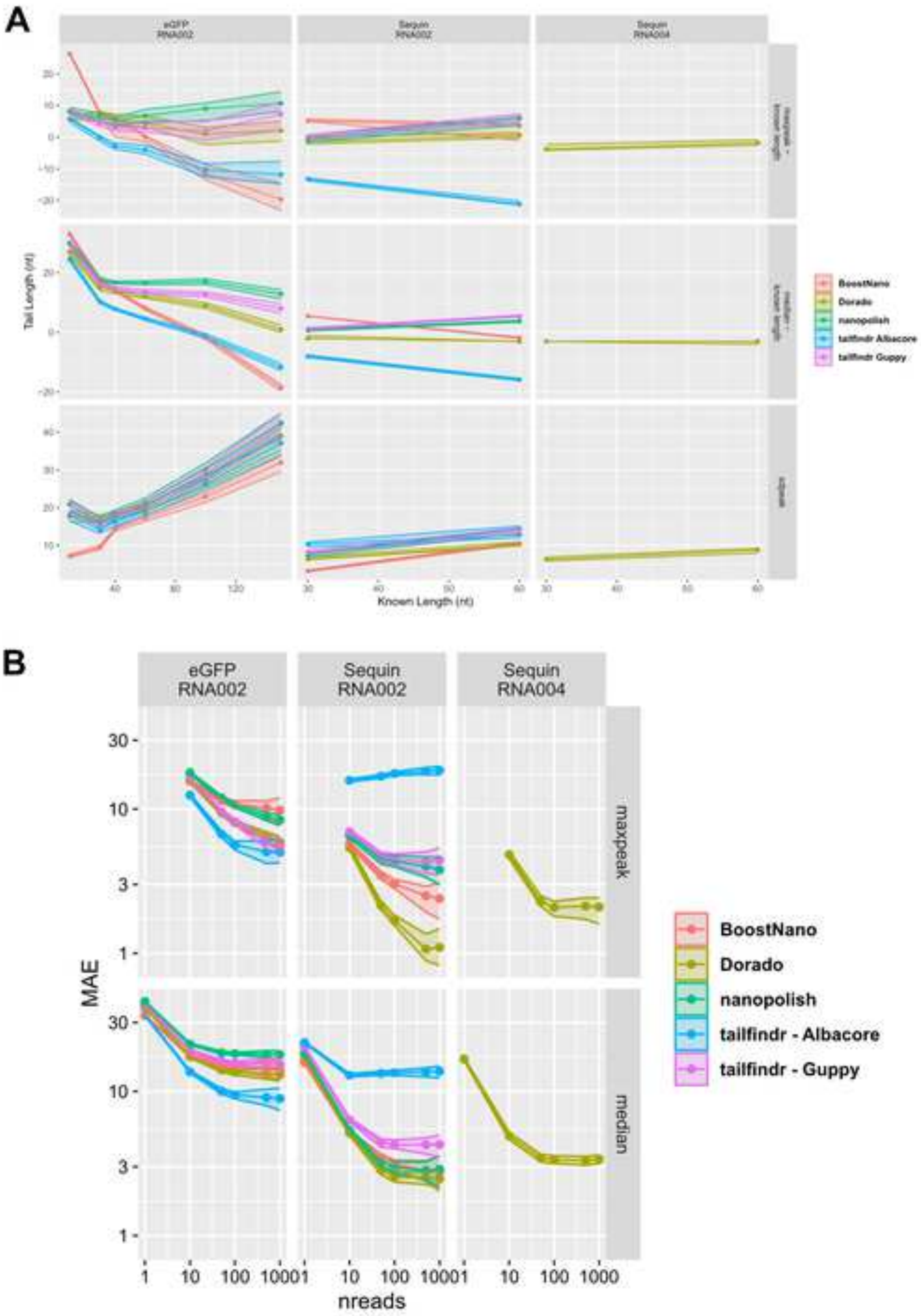

Figure 3

Click here to access/download;Figure;Figure 3.tiff

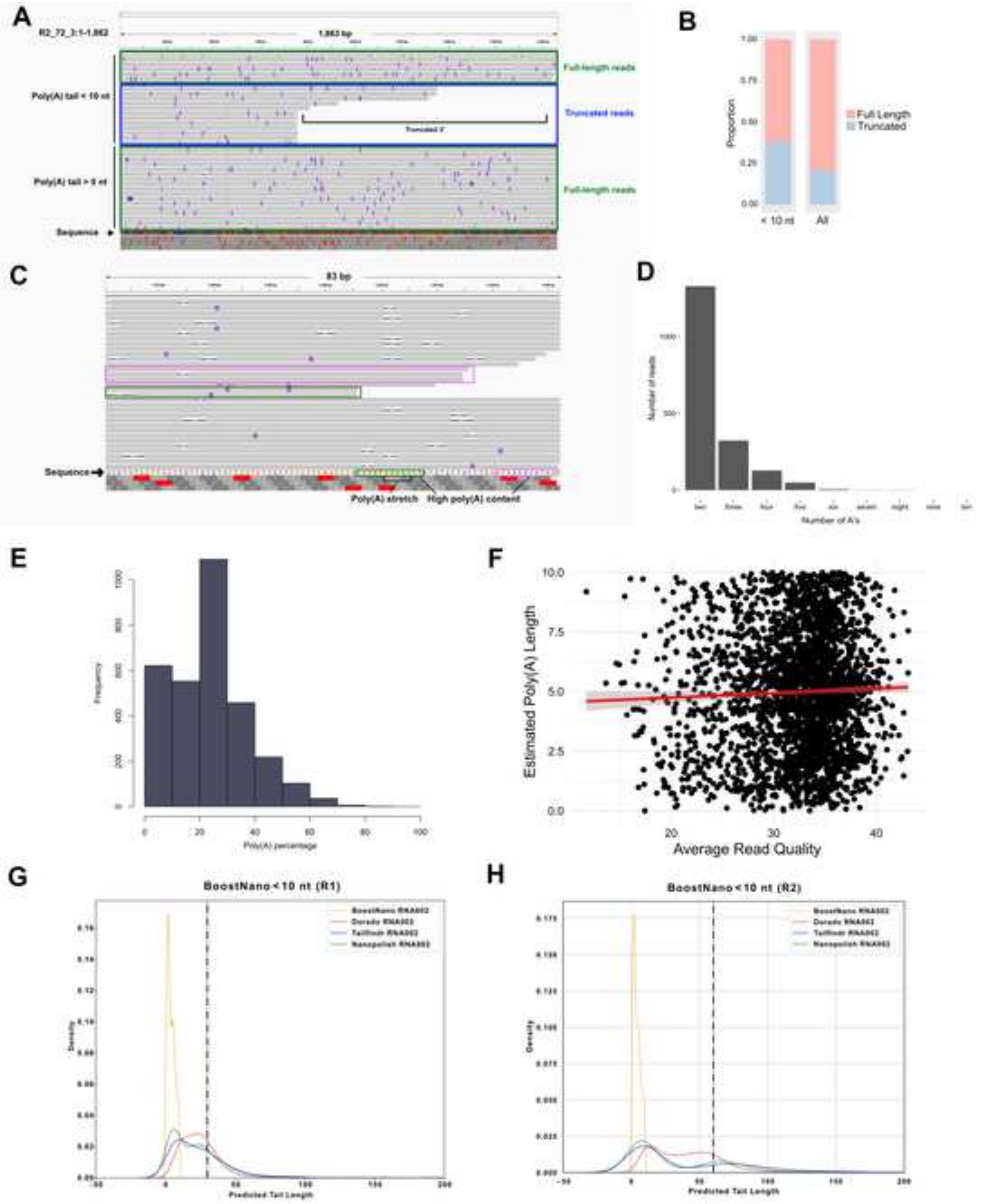

Figure 4

Click here to access/download;Figure;Figure 4.tiff

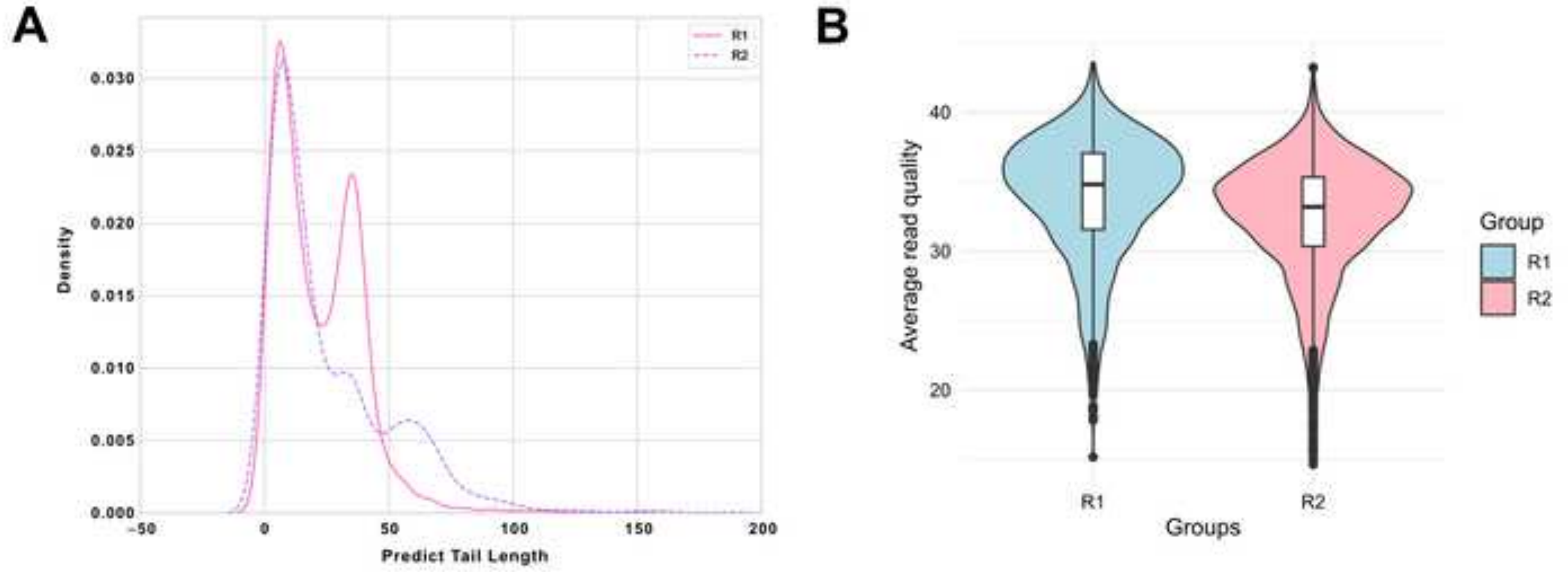

Click here to access/download
**Supplementary Material**
Supplementary_boostnano_07_01_25_v2_FINAL.docx

Supplementary Data S1

Click here to access/download
**Supplementary Material**
Supplementary Data S1.xlsx

Supplementary Data S2

Click here to access/download
**Supplementary Material**
Supplementary Data S2.xlsx

Supplementary Data S3

Click here to access/download
**Supplementary Material**
Supplementary Data S3.xlsx

Cover Letter

Click here to access/download;Personal Cover;cover_letter_gigascience.docx

THE UNIVERSITY OF MELBOURNE

24th September 2024

Dear Editors

**Re: Submission of our manuscript Gigascience**

We would like to submit the attached manuscript *"Using synthetic RNA to benchmark poly(A) length inference from direct RNA sequencing"* to *Gigascience* for consideration as a technical note.

Neural networks have proven essential to accurate base-calling – including the *Chiron* basecaller we previously published in Gigascience. However - until recently - neural networks have not been trained to directly identify poly(A) tail length. In this paper we describe a neural network approach characterising poly(A) tail length. Oxford Nanopore Technologies have also trained a neural network to characterise poly(A) tail length, which has been released as part of the *Dorado* basecaller. While these are exciting developments for poly(A) length estimation, the accuracy of these methods has not yet been explored.

In this paper we focus on benchmarking neural network based approaches to polyA length estimation with pre-existing tools *tailfindr* and *nanopolish*. We analyse synthetic RNA with known poly(A) tail length to develop a gold-standard dataset for benchmarking. We also describe our new approach *BoostNano*.

We provide estimates of the accuracy of poly(A) tail length estimation for each method and find that the neural network approaches are more accurate, and – in the case of *Dorado* – substantially faster than pre-existing approaches. We also identify a substantial portion of reads for which *Dorado* is unable to provide an estimate of poly(A) tail length, and show that this is perhaps largely due to a combination of polyA tail fragmentation and internal priming.

We believe that this manuscript will be an important point of reference for the poly(A) community, particularly as a description and evaluation of the commercial tool (*Dorado*) has not (and likely will not) be published.

Yours sincerely,

**Lachlan Coin**
Professor of Computational Biology
The Peter Doherty Institute for Infection and Immunity

**Postal address:**
**792 Elizabeth Street, Melbourne, VIC 3000, Australia**

**Phone:**
**+61 3 8344 3831**

Dear Editor,

We sincerely appreciate the time and dedication of you and the reviewers to have spent on reviewing this manuscript.

Please kindly find our responses to reviewers' comments in blue below and please note that all grammar correction suggestions have been appropriately incorporated into the new version of the manuscript.

Please note that we have updated the Dorado dataset to include data from the latest version of Dorado v0.9.0.

External email: Please exercise caution

GIGA-D-24-00432

Using synthetic RNA to benchmark poly(A) length inference from direct RNA sequencing.

Jessie J-Y Chang; Xuan Yang; Haotien Teng; Benjamin Reames; Vincent Corbin; Lachlan Coin

GigaScience

Dear Dr Coin,

Happy New Year!

Your manuscript "Using synthetic RNA to benchmark poly(A) length inference from direct RNA sequencing." (GIGA-D-24-00432) has been assessed by our reviewers. Although it is of interest, we are unable to consider it for publication in its current form. The reviewers have raised a number of points which we believe would improve the manuscript and may allow a revised version to be published in GigaScience.

Their reports, together with any other comments, are below. Please also take a moment to check our website at https://www.editorialmanager.com/giga/ for any additional comments that were saved as attachments.

In addition, please register any new software application in the bio.tools and SciCrunch.org databases to receive RRID (Research Resource Identification Initiative ID) and biotoolsID identifiers, and include these in your manuscript. Computational workflows should be

registered in workflowhub.eu and the DOIs cited in the relevant places in the manuscript. These will facilitate tracking, reproducibility and re-use of your tool.

If you are able to fully address these points, we would encourage you to submit a revised manuscript to GigaScience. Once you have made the necessary corrections, please submit online at:

We have added boostNano to bio.tools and SciCrunch.org databases and referred the relevant ID's in the manuscript as the Editor has kindly suggested. We have additionally noted links as appropriate to refer to data and scripts on Figshare, which will be replaced with DOIs after acceptance.

https://www.editorialmanager.com/giga/

If you have forgotten your username or password please use the "Send Login Details" link to get your login information. For security reasons, your password will be reset.

Please include a point-by-point within the 'Response to Reviewers' box in the submission system. Please ensure you describe additional experiments that were carried out and include a detailed rebuttal of any criticisms or requested revisions that you disagreed with. Please also ensure that your revised manuscript conforms to the journal style, which can be found in the Instructions for Authors on the journal homepage. If the data and code has been modified in the revision process please be sure to update the public versions of this too.

The due date for submitting the revised version of your article is 07 Apr 2025.

I look forward to receiving your revised manuscript soon.

Best wishes,

Hongfang Zhang

GigaScience

www.gigasciencejournal.com

Reviewer reports:

Reviewer #1: In this manuscript, the authors present a benchmark to assess the performance of different tools designed for estimation of polyA tail length from Nanopore

direct RNA-sequencing data. These tools include tailfindr, nanopolish, Dorado and Boost Nano.

Benchmarks on tools and algorithms to analyze Nanopore data, both third party tools and official ONT releases, are of utmost importance for the field. The use of synthetic constructs with known ground truth is recommended as well. Consequently, this study has the potential to provide a significant contribution to the field.

In the current form, I can however not recommend it for publication in GigaScience. My major concerns are:

a) Use of only RNA002 data. This chemistry is outdated and thus the Benchmark is only relevant for old, possibly already published data. A comprehensive Benchmark should also include RNA004 and available tools there (at least Dorado).

We have generated new RNA004 data of sequins for this revision. We have analysed this data using *Dorado* v0.9.0 in our manuscript. We attempted to use other tools with RNA004 data, but this was not possible due to incompatibility. Nevertheless, we are able to compare the estimates from RNA004 data to RNA002 data.

b) The current data set only contains two polyA tail length, which are relatively short and do not cover longer polyA tails that are common e.g. in mammalian cells. A proper Benchmark should show the performance of the analyzed tools over a range of polyA tail lengths.

We have added synthetic IVT eGFP RNA data that span over a range of polyA tail lengths derived from the developers of tailfindr. The data include RNA with poly(A) tails with 10, 30, 40, 60, 100, 150 nt.

Minor comments:

Abstract: "All four tools generate mean tail-length estimates which lie within 13% of the correct value." The value of 13% is given in the Abstract from the submission system, wherease the abstract in the Main text says 12%. Which value is correct?

We have now removed this statement from the Abstract and the Main Text as we no longer focus on mean tail length.

2) Background, first paragraph: the role of the polyA tail in RNA circularization, which is required for efficient translation of cellular mRNAs is not mentioned. Reference is missing for "is increasingly recognised as a dynamic process which influences timing and degree of protein production."

This has been moved to the second paragraph of the background - the role of the poly(A) tail in RNA circularization and the appropriate references were added (lines 65-68).

3) Background, second paragraph: Chiron seems to be a relatively old basecaller (no models for new chemistries). It should be mentioned here that it is required for BoostNano.

Chiron is not required for BoostNano, as basecalling is not necessary for using the tool. Instead, the model structure of Chiron is used as a basis for BoostNano. We have clarified this in Table 1 – "Convolutional Neural Network (CNN)-Recurrent Neural Network (RNN)-Connection-ist Temporal Classification (CTC) architecture from Chiron basecaller used to find boundaries of poly(A) in raw signal, basecalling not required"

4) Mis-priming of internal polyA sites may an important confounding (and currently overlooked) source of errors in Nanopore sequencing. This should be quantified properly and analyzed in more detail (length of these stretches, influence of other nucleotides within the A-rich stretch, etc.). Should be done as well on whole transcriptome data with more possible mispriming sites.

We have analyzed the whole Sequin transcriptome dataset to understand and quantify priming due to internal polyA sites or fragmentation via visualising the 10 nt downstream of the 3' end of the mapped transcript in the reference transcriptome, as the poly(T) stretch of the RTA is 10 nt long. Furthermore, we analyzed the poly(A) content by calculating the percentage of adenines in that 10 nt stretch. This information was added as Figures 3B, D-E.

5) Why do the authors think that the poly(T) stretch of the RTA might be truncated? This is composed of DNA oligos, which should be quite stable

Yes, we agree that DNA oligos are much more stable than RNA oligos and therefore should be quite stable. However, that does not mean that truncation and fragmentation cannot happen with DNA oligos. Mechanical forces, freeze-thaw and enzymatic hydrolysis can contribute to the degradation of DNA oligos. Please see the following publication discussing the effect of freeze-thaw to DNA oligos with 10T's (Davis, O'Brie & Bentzley, 2000 - Analysis of the Degradation of Oligonucleotide Strands During the Freezing/Thawing Processes Using MALDI-MS DOI: 10.1021/ac000225s).

6) What are the parameters for filtering used by Dorado and BoostNano? Can the authors explain, why the filtered reads differ?

We have only utilized default parameters for both Dorado and BoostNano, hence no specific filtering parameter was utilized. Dorado bases its poly(A) estimation on an analysis of the

signal, searching for a low variability region near its identified anchor point (typically the cDNA primer or RNA adapter) and an estimate of the translocation speed based on the move table of the read. If the low variability region or anchor point cannot be located, the reads could be filtered out. BoostNano assigns poly(A) tails to all reads.

7) Dorado seems to systematically underestimate polyA tail length. Is this true also for data generated with RNA004 chemistry and longer polyA tails?

Dorado's poly(A) tail length estimates tend to be the most accurate - please see updated Figures 1a and 1b. When comparing RNA002 and RNA004, Dorado seems to underestimate poly(A) tails more in RNA004 data than RNA002 data.

Reviewer #2: This manuscript addresses a relevant and timely question: benchmarking poly(A) tail-length estimation tools (BoostNano, tailfindr, nanopolish, and Dorado) using synthetic RNA standards (Sequins) with known tail lengths. Poly(A) tail-length estimation is increasingly important for understanding mRNA stability, processing, and regulation at the single-molecule level. As direct RNA sequencing expands in use, reliable methods to measure poly(A) tail lengths are needed. The study's design—leveraging Sequins as a "gold standard" to benchmark tools—is strong and fills an area is need in current literature. The analysis is thorough in its basic comparisons, and the results are likely to be useful to researchers who need to choose suitable software for poly(A) tail analysis. However, the manuscript would benefit from deeper contextualization, more rigorous statistical methodology, and clearer reporting of computational details. Ensuring reproducibility and providing clearer guidance on interpreting the results in real biological contexts would strengthen the manuscript. The suggestions below are aimed at making the study more valuable to the community.

For this reason, my recommendation is Revisions ARE Needed

Introduction

Abstract: ★★★★☆ (4/5) Actually in place of the introduction, it has it strengths:

The introduction adequately outlines why polyadenylation is biologically important and why direct RNA sequencing provides a unique opportunity for poly(A) tail-length estimation.

It justifies the use of Sequins as synthetic standards, which is a robust approach to derive ground-truth tail lengths.

Areas for Improvement: The introduction could better connect poly(A) tail-length estimation to downstream applications. For instance, mention how accurate tail-length estimation could improve understanding of mRNA decay rates, translation efficiency, or isoform-specific regulation.

We have included wording to connect poly(A) estimation to downstream applications in both the abstract and the introduction (lines 32-34, 65-76).

Adding references that contextualize poly(A) tail dynamics in broader biological phenomena would help readers understand the significance.

For example, it is almost a necessity to cite work such as "Roles of mRNA poly(A) tails in regulation of eukaryotic gene expression" by Lori A. Passmore & Jeff Coller (2022, Nature Reviews Molecular Cell Biology) which provides a comprehensive analysis of poly(A) tail dynamics and their impact on mRNA decay, stability, and translation regulation. P & C (2022) also expands on these principles by discussing the mechanistic underpinnings of poly(A)-mediated decay and translation regulation, making it a broader and more recent contribution to polyadenylation biology, which the authors should consider.

Thank you for the suggestion – we have now referenced this work and other relevant publications in our manuscript (page 2).

Grammar of the abstract:

Error: "There are currently several tools available for poly(A) tail-length estimation, including well-established tools such as tailfindr and nanopolish, as well as two more recent deep learning models: Dorado and BoostNano."

- ✓ Suggestion: "Several tools are currently available for poly(A) tail-length estimation, including well-established methods like tailfindr and nanopolish, as well as two more recent deep learning models: Dorado and BoostNano."

Error: "which lie within 12% of the correct value."

- ✓ Suggestion: "that lie within 12% of the correct value."

Clarify the library preparation steps to avoid confusion about the "direct" nature of RNA sequencing. The text currently implies that no reverse transcription is required, but then references an ONT Reverse Transcription Adapter. Distinguish between a full-length cDNA synthesis step (not required) and the use of a poly(T)-containing adapter for sequencing library preparation.

Thank you for the suggestion. We have added amended the relevant paragraph in the Background section:

In contrast, Oxford Nanopore Technologies (ONT) direct RNA-sequencing is a simple approach for single-molecule RNA-sequencing which does not require reverse transcription (other than for RNA stabilization and improving sequencing output) or polymerase chain reaction (PCR) amplification, thus avoiding amplification bias and retaining the original base

and base-modification information [35-39]. It is worth noting that the full-length cDNA synthesis step, while not required, is recommended and the library preparation method utilizes a poly(T)-containing adapter for sequencing. (lines 82-88)

Methods

Methods: ★★★★☆ (4/5)

The methods section has its strengths; the data sources and preparation (Sequins spiked into host RNA) are clearly described. Versions of tools are provided, enhancing reproducibility.

Areas for Improvement are statistical analysis, comparisons and tests, hardware and computation details, and understanding of run time differences.

All aspects of areas of improvement suggested by the reviewer have been addressed and improved in the newest version of the manuscript. We have modified the analysis to include calculation of mean absolute error (MAE), as well as averaging multiple reads using medians and position at which the probability density function achieves is maximum. We now include confidence intervals on all estimates which have been calculated via bootstrap resampling. Also, we added more details regarding computational details – including RAM and processors (Table 2).

Currently, the study models distributions as normal and uses mean and SD, but no normality tests or justification for these choices are presented. Consider performing normality tests or using nonparametric measures. Additionally, providing confidence intervals or other robust statistics (median, interquartile ranges) would clarify variability.

For the comparisons and tests, the authors should explain why you chose root mean square error (RMSE) minimization and other metrics.

Could alternative tests, like Wilcoxon signed-rank tests or paired t-tests (Wilcocoxon: this non-parametric test is suitable for paired comparisons when the assumption of normality is not met. -useful to compare the predicted tail lengths from each tool against the expected lengths, especially if the data distribution is skewed.), be used to compare the distribution of tail-length estimates more rigorously?

Paired t-Test, because this test could be applied if the normality assumption holds, providing a straightforward way to assess whether the mean difference between predicted and expected values is statistically significant. (If so, justification should be provided for why or why not)

Our data is not normally distributed, it has a multi-modal distribution. We only use the term “normal” in the shape of the highest peak, so t-tests will not be feasible for comparing the

predicted tail lengths vs expected tail lengths. We have now removed the part where we model the second peak as normal distribution, and RMSEs to alleviate any confusions. We have added confidence intervals, SDs, medians and maximum peak of the density distribution instead (Data S1, Figures 2a-b). We use bootstrap resampling to evaluate significance of one tool having smaller mean absolute error than another, thus avoiding use parametric approaches for significance testing.

There are some additional metrics to explore:

---Median Absolute Deviation (MAD): Consider adding MAD as it is robust to outliers and could complement RMSE to provide a better understanding of central tendencies and variability.

---Mean Absolute Error (MAE): MAE is another alternative that simplifies the interpretation by focusing solely on the magnitude of errors without squaring them, potentially offering more intuitive insights for readers.

We explored the MAE of each tool across windows of various sizes and explored the median or maximum peak values. The results have been added as Figures 2b and S7, as we believe this metric would be the most beneficial to the readers.

The authors should address testing for normality, explicitly stating whether normality tests were conducted on the data (e.g., Shapiro-Wilk or Kolmogorov-Smirnov tests). If normality is confirmed, justify the use of parametric tests like RMSE or t-tests. If not, justify why non-parametric tests (e.g., Wilcoxon) were not employed or discuss plans to include them in future studies.

We are no longer implementing the normal distribution strategy and therefore have not carried out the suggested normality tests.

Explain the choice of statistical methods over time by discussing how the choice of statistical tests aligns with the study's goals. For example, emphasize whether the focus was on understanding overall error distribution, tool consistency, or accuracy in predicting specific tail lengths.

We have implemented statistical methods to understand all of the above – overall error distribution, tool consistency and accuracy in predicting specific tail lengths. We believe that the maximum peak of density distribution and the MAE are most appropriate metrics that align with the study's goals.

The authors could use visual representations of error complementing the statistical tests with visual aids such as boxplots, violin plots, or Bland-Altman plots to illustrate the error distributions and discrepancies between predicted and actual tail lengths across tools.

(maybe supplementary)

We have added plots showing the Mean absolute error of all tools (all, R1 and R2) in Figures 2b and S7.

The authors should provide hardware and computational details like providing explicit details on the computational environment—CPU/GPU models, RAM, OS—for each tool's run. While the Git-hub read me suggests how to run the system, it lacks any details about system requirements.

Readers need this to understand runtime differences and attempt to replicate performance measurements.

We have updated the Github README to include details about the system requirements (e.g. Python version, OS). The suggested hardware and computational details have been added in Table 2.

The authors should consider tool parameterization and indicate if any specific parameters (beyond defaults) were used in tailfindr, nanopolish, Dorado, or BoostNano runs. If no changes were made from defaults, state this explicitly.

We have added a table in the Methods to add the parameters used for analysis for each of the tools we have discussed in our manuscript (Table 3).

Results

The result's strengths are that they are presented clearly, showing density distributions and discussing short-tail anomalies. The identification of Dorado as a preferred tool due to speed, integration, and conservative filtering is well-supported by the data. The study acknowledges that all tools achieve broadly similar accuracy, differing mainly in runtime and filtering criteria, which is a practical insight for users.

The results have areas for improvement:

Regrading the short-tail reads explanation, the authors attribute short (<10 nt) poly(A) tails to truncated transcripts or mis-priming. For this reason, it is suggested that the authors strengthen this discussion with additional evidence or reasoning. For instance, is there a correlation between read quality and short-tail length estimates? Do truncated reads consistently align to internal A-rich stretches?

We generated correlation plots between expected poly(A) length and average read quality per read and used Spearman's correlation analysis to investigate the relationship (Figure S8). There is indeed a weak positive correlation between poly(A) length and read quality in some datasets (e.g. dorado R2, BoostNano R1, Boostnano R2 and tailfindr R1 in Sequins). However, we have identified that the truncated reads generally have an average read quality score > 20 (Figure 3f), showing that poor read quality score is not the main cause of these truncated poly(A) tails.  We have also included results to show that most truncated reads do not align to internal poly(A) stretches nor stretches with high poly(A) content (Figures 3d-e).

Multiple peaks in distributions: Some density plots (Figure 1) show multiple peaks or shoulder peaks.

Discuss potential reasons for these patterns. Are they related to tool-specific biases, read quality, or adapter/poly(T) truncation?

All tools in our study and all versions of these tools show either a multi-modal distribution or shoulder peaks (Figures 1a-c). This seems to indicate that this is not a tool-specific effect, except for *BoostNano,* which showed a clear bimodal distribution in R1 sequins and trimodal distribution in R2 sequins and eGFP datasets. We note that multiple peaks were more prominent in longer known poly(A) length RNA, and this was expected given that there is more chance for longer tails to become fragmented than shorter tails. Therefore, as the early peak presents with most tools, we believe this is a feature of the sample, instead of the tool. We discuss this in more detail on pages 5 and 12. We modelled the poly(A) length vs quality score and found a weak, positive correlation, suggesting that there is some correlation with read quality (Figure S8), however, this is unlikely to be the major reason for these patterns.

Application Context: The results focus on method performance, but it would help readers to understand how these differences might influence downstream tasks. For example, if a method overestimates poly(A) length slightly, how could this affect conclusions about RNA stability or differential tail-length analysis between experimental conditions?

We have added a paragraph regarding this in the first paragraph of the discussion section (lines 330-337).

"The implications of poly(A) estimation outputs can lead to different varied interpretations. If a method overestimates poly(A) tails, the researcher may overestimate other associated functions such as RNA stability, since longer poly(A) tails are commonly associated with greater stability. This is particularly detrimental when exploring differential polyadenylation, as even small changes in poly(A) tails may influence statistical tests. Thus, as a general, we recommend the following; should the researcher have specific expected tail lengths for their study, they should choose the most appropriate tool based on the results of this study or similar, with the use of maxpeaks to average over read sets (N>100). In more complex transcriptomes with wider variety of tail lengths, tailfindr or Dorado should be utilized."

Figures and tables:

Figure 1:

Clear density plots, but consider adding vertical lines at expected tail lengths (30 nt and 60 nt) to guide interpretation. Splitting the figure into separate panels for R1 and R2 or using insets might clarify multiple peaks.

We have added dotted vertical lines at expected tail lengths and split the figure into two separate panels for R1 and R2 as per the reviewer's recommendations (Figures 1a-b).

Figure 2:

The IGV snapshots are informative. Enhance interpretability by adding annotations (arrows or boxes) highlighting truncated vs. full-length reads. Increase font sizes for readability.

Thank you for the suggestions. Boxes to highlight truncated vs full-length reads were added to Figure 3A and we have generally increased the font sizes for readability.

Figure 3:

Useful comparison of reads filtered by Dorado but retained by BoostNano.

Add a brief note or labeling to indicate expected tail lengths.

Discuss possible reasons for Dorado's conservative filtering here or in the main text.

We have added a note to indicate the expected tail lengths in the figure legends. Dorado's conservative nature might be due to the tendency of the tool to base its estimation on searching for a low variability region near an anchor point, and if such regions are undefined, there could be an omission of the reads. This information has been added to the discussion section in the third paragraph.

Tables:

Provide definitions for abbreviations (nt, CPU, GPU) in captions. For Table 2, adding confidence intervals around the mean tail-length estimates would strengthen statistical rigor. For Table 3, specify hardware details as recommended above.

The abbreviations have been defined in the table captions. We have added confidence intervals for medians and maxpeaks in Supplementary Data S1. We have added computation configurations used in Table 2 for reproducibility.

Grammar Mistakes and errors in the results section:

Results Section:

Sentence: "The four methods display a similar pattern in the density distribution, with a prominent normal-like peak near the expected poly(A) length, but also with a over-representation of shorter poly(A) tails, ranging at approximately ~0-10 nt (Figure 1)."

Issue: "a over-representation"

- ✓ Correction: "an over-representation"

Sentence: "We expected that these shorter peaks were derived from either fragmentation of the transcript, mis-priming of internal poly(A) stretches or degradation of the poly(A) tails."

Issue: tense mismatch ("expected" vs. "were derived").

- ✓ Correction: "We expect" -- "were derived", loses context and tense conformity-- therefore the sentence should be adjusted-

"We hypothesize that these shorter peaks are derived from either fragmentation of the transcript, mis-priming of internal poly(A) stretches, or degradation of the poly(A) tails."

Sentence: "Interestingly, upon investigating these earlier peaks, we found that Dorado excludes reads which are retained in the analysis by BoostNano, despite them being classified as passed reads (Figure 3)."

Issue: Ambiguous pronoun "them." (them could incorrectly identify three possible targets in the sentence)

- ✓ Correction: "Interestingly, upon investigating these earlier peaks, we found that Dorado excludes reads retained in the analysis by BoostNano, even though these reads are classified as passed reads (Figure 3)."

Sentence: "Therefore, Dorado appears to be a more conservative approach than BoostNano."

Issue: No grammar issues, but the statement could be more precise.

- ✓ Suggested improvement: "Thus, Dorado demonstrates a more conservative approach compared to BoostNano."

Sentence: "In order to determine which normal distribution fit the peak best, we found the parameters (mean, SD) which minimize the root mean square error between the candidate

normal distribution and the density distribution for an interval of 10 nt to the right of the mode."

Issue: Verb tense consistency ("fit").

✓ Correction: "To determine which normal distribution fits the peak best, ..."

Sentence: "The peaks also lose their normal-like behavior for larger values."

- ✓ Issue: Could use a more formal tone. Correction: "The peaks also deviate from their normal-like behavior at larger values."

Sentence: "Next, we compared the computational time required by each method to predict the tail-length of 4000 reads."

Issue: Hyphenation of "tail-length."

Correction: "Next, we compared the computational time required by each method to predict the tail length of 4,000 reads."

We have revised all hyphenated versions of ‘tail-length’ to ‘tail length’.

Sentence: "BoostNano also offers the option of using the Application Programming Interface (API) call instead of the direct method, which omits the file copy implemented in the direct approach, reducing the run time to 8 m 8 s."

Here, the sentence is extremely overwritten which causes a lack of clarity.

- ✓ Correction: "BoostNano offers an alternative API-based method, which skips the file copy step of the direct approach, reducing the runtime to 8 minutes and 8 seconds."

Discussion

Discussion: ★★★☆☆ (3/5)

The discussion as its strengths as it correctly identifies that Dorado's advantages (speed, integration with basecalling) make it appealing as a default choice.

The authors acknowledge that all tools are within a similar accuracy range, suggesting the deciding factor may be speed or integration rather than raw performance differences.

HOWEVER- there are areas for improvement:

Further dissect the limitations of each tool. For example, BoostNano shows good SD but slightly off mean for R1; what does this mean for its use cases?

We have amended the approach for using metrics for determining accuracy – we have added medians, maxpeaks, SD, CI, MAE and raw differences instead of the mean. We have noted a length- and sample-dependency in our results, which we have discussed in the first paragraph of the discussion section. In the same paragraph we noted – “The implications of poly(A) estimation outputs can lead to different varied interpretations. If a method overestimates poly(A) tails, the researcher may overestimate other associated functions such as RNA stability, since longer poly(A) tails are commonly associated with greater stability. This is particularly detrimental when exploring differential polyadenylation, as even small changes in poly(A) tails may influence statistical tests. Thus, as a general, we recommend the following; should the researcher have specific expected tail lengths for their study, they should choose the most appropriate tool based on the results of this study or similar, with the use of maxpeaks to average over read sets (N>100). In more complex transcriptomes with wider variety of tail lengths, tailfindr or Dorado should be utilized.”

Address the discrepancy between tailfindr, nanopolish, and Dorado in terms of how they define and detect poly(A) boundaries. Why does Dorado not evaluate start/end positions of poly(A) tails in event space, and how might this influence results?

The differences in determining poly(A) boundaries in each tool have now been described in the background section (lines 106-121). Apologies for the confusion, we meant that Dorado does not output start and end positions of the poly(A) tails, nor the signals, unlike the other tools. Dorado of course must calculate start and end positions for estimating poly(A) tails.

Include a brief discussion about how results might generalize to more complex transcriptomes. Real samples have varying GC content, fragment lengths, and potentially modified bases. A short commentary acknowledging these factors would show awareness that synthetic standards cannot capture the full complexity of natural RNA populations.

We added a brief discussion about the generalizability of our data in the paragraph regarding limitations of this study in the discussion section (page 13, lines 382-392).

For these reasons, it is suggested that the authors suggest future directions.

For instance, how could tool developers incorporate these findings to improve their methods? Could future benchmarking sets include a gradient of tail lengths to better understand length-specific biases?

A section of future directions for tool development and tool benchmarking has been added in the discussion section (lines 397-399).

Grammar Mistakes and errors in the discussion section:

Sentence: "BoostNano and tailfindr tools provided estimation of the starting and ending positions of the poly(A) tails in event space while this information was absent in Dorado outputs."

Issue: "provided estimation" should be "provide estimation" to align with present tense.

✓ Correction: "BoostNano and tailfindr tools provide estimation of the starting and ending positions of the poly(A) tails in event space, while this information is absent in Dorado outputs."

Sentence: "On the R1 dataset, BoostNano showed a tighter distribution with the smallest SD, but its peak was the furthest from the correct value."

The issue here is that the test results are still speaking about general truths leading to verb tense inconsistency; "showed" should match other verbs in the section.

- ✓ Correction: "On the R1 dataset, BoostNano shows a tighter distribution with the smallest SD, but its peak is the furthest from the correct value."

Sentence: "tailfindr had the most accurate estimation but also the largest error interval."

The issue here is the verb tense mismatch; "had" should be consistent with present tense to show truth, not past truth.

- ✓ Correction: "tailfindr has the most accurate estimation but also the largest error interval."

Sentence: "Furthermore, Boostnano is more lenient in keeping reads for poly(A) estimation than Dorado."

Issue: "Boostnano" capitalization error; it should be "BoostNano."

- ✓ Correction: "Furthermore, BoostNano is more lenient in keeping reads for poly(A) estimation than Dorado."

Sentence: "Overall, our results suggest that the four tools investigated in this study - BoostNano, tailfindr, nanopolish and Dorado have similar performance with their accuracy varying from one dataset to the other, with a potential length bias."

Issue: Missing commas for clarity; replace "with their accuracy varying from one dataset to the other" for conciseness.

- ✓ Correction: "Overall, our results suggest that the four tools investigated in this study—BoostNano, tailfindr, nanopolish, and Dorado—have similar performance, with accuracy varying across datasets and showing potential length bias."

Sentence: "Therefore, we expect Dorado to be implemented as the default method of poly(A) tail estimation in the near future, with the rapid estimation timeframe, comparable estimation lengths to other tools, conservative nature and the added benefit of ease of obtaining this information during basecalling."

There are several issues here including verbosity and lack of parallelism.

- ✓ Correction: "Therefore, we expect Dorado to be implemented as the default method for poly(A) tail estimation, given its rapid estimation timeframe, comparable accuracy to other tools, conservative nature, and ease of integration with basecalling."

Sentence: "This work demonstrates the value of having access to synthetic RNA molecules with known poly(A) tail-lengths for validating the accuracy of poly(A) tail estimation algorithms."

Issue: The phrase "validating the accuracy of" could be simplified for readability.

- ✓ Correction: "This work demonstrates the value of synthetic RNA molecules with known poly(A) tail lengths for validating poly(A) tail estimation algorithms."

Sentence: "As methods improve, we anticipate that these datasets will be valuable for assessing improvements in estimation of poly(A) tails."

Issue: "improvements in estimation of" is awkward.

- ✓ Correction: "As methods improve, we anticipate that these datasets will be valuable for assessing advancements in poly(A) tail estimation."

References need to be added to accommodate the suggested material review, but existing references are good

We have added more relevant references to the manuscript to supplement our findings and the background/discussion.

NEEDS REVISION

Jesse Daniel Brown PD AASU

Note:

I previously reviewed this paper previously in Research Hub and you can read these comments via the Research Hub review page here: https://www.researchhub.com/paper/8634403/using-synthetic-rna-to-benchmark-polya-length-inference-from-direct-rna-sequencing/reviews#threadId=55398.

The original preprint linked to the Research Hub review is here: https://doi.org/10.1101/2024.10.25.620206

Reviewer #3: The manuscript presents interesting findings, but several points require clarification and further discussion to enhance transparency, statistical robustness, and reproducibility. Addressing the disdcrepancies in Figure 1, justifying the choice of statistical methods, and providing detailed computational configurations will significantly improve the manuscript. Based on the above stated, my recommendation it is by accepting for publication after a major revision.

Discrepancies in Figure 1 have been addressed in the first paragraph of section ("Performance evaluation between BoostNano, tailfindr, nanopolish and Dorado"). We utilized the maxpeak metric and bootstrap based p-values to understand the accuracy of each test and have discussed this in the manuscript (page 5, lines 158-171, page 10, lines 276-289). Detailed computational configurations have been listed in Table 2 as well as updated methods for enhancing reproducibility.

Regarding the criteria from GigaScience Journal for Technical Notes this technical note comply partially with technical improvement/utility, because while the study demonstrates that Dorado is the fastest and most conservative method, the novelty of technical improvement is somewhat limited because the tools themselves are not newly developed by the authors (exception of BoostNano). That said, the benchmarking study provides value for the research community by helping users choose the most appropriate tool for poly(A) tail-length estimation.

The reviewer declare that he does not have competing interests.

The abstract provides a concise summary of the study's aim, methodology, and key findings. However, it lacks sufficient emphasis on the broader implications of benchmarking poly(A) tail-length estimation for transcriptomics research. The abstract mentions the advantages of Dorado in terms of speed and accuracy but does not explicitly describe the limitations of other tools or potential use cases of these findings. Expanding the abstract to connect the results to downstream applications such as transcript stability analysis or isoform quantification could improve its utility.

We have added the following to the abstract to connect the results to downstream applications: “These results provide a reference for poly(A) tail length estimation analysis, aiding in improving our understanding of the transcriptome and the relationship between poly(A) tail length and other transcriptional mechanisms, including transcript stability or quantification.” (lines 32-34)

The language is clear and concise, with only minor grammatical errors. For instance: "We expect that the poly(A) tails shorter than 10 nt occur due to potential truncation…" could be rephrased as "We hypothesize that poly(A) tails shorter than 10 nt may result from truncation…". And the sentence "BoostNano also offers the option of using the Application Programming Interface (API) call instead of the direct method, which omits the file copy implemented in the direct approach, reducing the run time to 8 m 8 s." is verbose and could be simplified for readability.

The first sentence with grammatical errors has been amended (lines 281-282) and the second sentence has been removed due to updates to the results and methodology of our study.

Introduction

The introduction effectively outlines the biological significance of polyadenylation and the technical challenges in poly(A) tail-length estimation. It provides a clear rationale for using synthetic RNA (Sequins) as a benchmark. However, the scope of the discussion is narrow, with limited exploration of how poly(A) length affects broader transcriptomics applications. Additionally, the references focus on tool-specific papers, but there is a gap in discussing recent advances in nanopore sequencing or polyadenylation biology. One reference could be referenced and that was not cited is Lima SA, Chipman LB, Nicholson AL, Chen YH, Yee BA, Yeo GW, Coller J, Pasquinelli AE. Short poly(A) tails are a conserved feature of highly expressed genes. Nat Struct Mol Biol. 2017 Dec;24(12):1057-1063. doi: 10.1038/nsmb.3499, this reference discusses the broader impact of poly(A) tail dynamics on mRNA decay and transcript stability, which could frame the importance of accurate estimation tools.

We have added this paper as a reference (Lima et al., 2017) as per the reviewer’s suggestion in the second paragraph of the “Background” section (page 2). We have also expanded the Background to incorporate a more detailed review of the importance of studying polyadenylation dynamics, recent advances in Nanopore sequencing, and polyadenylation biology.

Methods

3.5/5.0

The methods section is well-detailed, with clear descriptions of dataset preparation, tool execution, and parameter usage. However, there is a lack of explanation regarding the statistical tests used to compare tool performances (e.g., root mean square error minimization).

We have now explained the use of maxpeak (which is the value at which the probability density function achieves its maximum) and MAE for comparing tool performances. (lines 506-514).

Additionally, the choice of gold-standard datasets (R1 and R2 Sequins) is appropriate, but the lack of diversity in tail lengths (only 30 and 60 nt) limits the generalizability of the findings. Including datasets with intermediate or extreme tail lengths could provide a more comprehensive evaluation. A discussion of potential batch effects (e.g., variability in RNA spiking or sequencing conditions) is also missing.

We have added extra datasets derived from eGFP RNA ranging from 10 nt to 150 nt poly(A) tails to improve our study. We believe that batch effects will not affect our study majorly as the same dataset was used across all tools, and our conclusions now stem from the aforementioned additional eGFP dataset from the developers of *tailfindr*, which adds additional confidence in our observations. However, we have added discussions about the limitation in the lack of sequencing replicates and its effect on more complex transcriptomes without a ground truth (page 13, lines 382-399).

Regarding the statistics used, the authors estimated the accuracy for each method using the mean and standard deviation. However, it is unclear why these metrics were chosen instead of alternatives such as the median and standard error of the mean. Further, the authors model the greatest peak as a normal distribution but do not clarify whether they tested for normality. To strengthen the statistical rigor, I suggest performing a normality test on the values to justify the choice of statistical measures and tests. The rationale for selecting the final statistical method should also be explained. Moreover regarding the statistics, the authors used a root mean square error (RMSE) approach to fit the normal distribution of predicted poly(A) tail lengths. This is appropriate for measuring deviations but fails to address biases in individual datasets. For instance, the difference in standard deviation (SD) between R1 and R2 sets suggests tool-specific variability that could affect downstream analyses. Incorporating additional tests, such as paired t-tests or nonparametric comparisons (e.g., Wilcoxon signed-rank test), could strengthen the statistical rigor.

We have now updated the metrics to include the median and the point at which the probability distribution achieves its maximum (maxpeak) where the latter metric proved to be a favorable method (page 7, Figure 2b). We no longer model the greatest peak as a normal distribution and have removed all instances of referring to this in the manuscript. We also replaced the RMSE approach with the MAE as per the reviewers' suggestions (Figure 2b). We employed bootstrap calculation on the difference between MAEs from different tools to obtain p-values (Data S3).

Finally, the methods section does not provide sufficient details about the computational configurations used (e.g., GPU or CPU specifications, processor type, RAM capacity). These details are critical for reproducibility. I recommend that the authors clearly specify the hardware and software configurations used for both GPU and CPU implementations.

We have added computation configurations as recommended in Table 2 for reproducibility.

Results

The results section is structured and includes sufficient detail to support the conclusions. The comparative analysis between tools is informative, with clear summaries in Table 2 and computational time results in Table 3. However, certain findings, such as the over-representation of short poly(A) tails (<10 nt), are not explored in depth. The analysis would benefit from a discussion of whether this phenomenon is due to RNA degradation or mispriming.

We have carried out additional analyses to explore the 10 nt stretch of sequence downstream of the 3' end of truncated reads and have identified that poly(A) tail degradation/truncation is the most prevalent reason for the short poly(A) tails. Mispriming and read quality were found only contribute to minimal number of reads (Figures 3d-f).

Additionally, while Dorado is identified as the preferred tool, the implications for specific use cases (e.g., low- versus high-throughput experiments) are not discussed.

Implications for specific use cases have been highlighted in the discussion section, and we have noted "while Dorado is identified as the preferred tool, the researcher may prefer accuracy over time, especially in the case of low-throughput datasets. In this case, other methods like tailfindr may be implemented according to specific contexts." (page 12, lines 350-352)

For the R1 test set, the expected mean is not clearly explained. The authors should include a detailed explanation of how this expected mean was derived, including the calculations or assumptions used.

The term "expected mean" was inappropriately used. This phrase was used to describe the known lengths of the Sequins (i.e. 30 nt for R1 and 60 nt for R2). This has been amended for clarity in the manuscript.

Figure 1: in Figure 1, some graphs (e.g., boostnano_R1, boostnano_R2, dorado_R2, tailfindr_R2, nanopolish_R2) display two density peaks, while others do not. It is unclear why this discrepancy exists. I recommend that the authors address this in the results section by explicitly identifying these observations and discussing possible reasons for the differences in the discussion section.

We have explicitly identified these observations in the first paragraph of section “Performance evaluation between BoostNano, tailfindr, nanopolish and Dorado”, and discussed the reasons in the discussion section in the third paragraph.

Figure 2: the subset of reads visualized with IGV is useful but underexplored. Additional annotations highlighting the differences between truncated and full-length reads could improve interpretability.

Additional annotations to highlight the truncated and full-length reads were added to improve interpretability (Figure 3a).

Figure 3:  while the focus on discarded reads is relevant, the figure does not explore the functional consequences of these differences. Including histograms of read qualities or mapping scores could provide additional insights.

We have added violin plots of read qualities of the discarded reads as Figure 4b.

Table 1: the descriptions are adequate but could include more technical details about how each tool handles noise or variability in signal.

How each tool handles noise or variability in the signals depends on the way each model has been trained. This information is not described in detail in the current existing publicly available literature, and thus it is unfortunately difficult to describe in the manuscript. Therefore, we will omit this information in the resubmitted manuscript.

Table 2: the summary statistics are clear but lack confidence intervals for mean and SD, which would provide a better sense of variability.

We added medians and maxpeak data with confidence intervals in Data S1.

Table 3: the computational time analysis is thorough. However, it would be beneficial to provide details about hardware specifications (e.g., CPU/GPU model) for reproducibility as already suggested.

We have added computation configurations used in Table 2 for reproducibility.

Discussion

The discussion appropriately interprets the results, emphasizing Dorado's advantages in speed and accuracy. However, it misses an opportunity to provide deeper insights into the limitations of the tools evaluated, particularly BoostNano's tendency to overestimate shorter tails and tailfindr's larger error intervals. The potential impact of sequencing coverage and RNA integrity on poly(A) tail estimation is also underexplored. Besides that, in the discussion section, the authors do not address why the Dorado method does not evaluate the start and end of poly(A) tails in the event space. This omission may influence the observed differences in performance between Dorado, Boostnano, and Tailfindr. I suggest that the authors discuss this limitation and its potential impact on their findings, such as differences in processing speed.

We provide deeper insights into limitations of the tools studied, and we provide the following recommendation based on these tool-based limitations in the first paragraph of the discussion section "Thus, as a general, we recommend the following; should the researcher have specific expected tail lengths for their study, they should choose the most appropriate tool based on the results of this study or similar, with the use of maxpeaks to average over read sets (N>100). In more complex transcriptomes with wider variety of tail lengths, tailfindr or Dorado should be utilized."

We have added a paragraph (paragraph 4) in the discussion section discussing the potential impact of sequencing coverage and RNA integrity on poly(A) tail estimation.

In the initial submission, we noted that the Dorado method does not output the start and end of poly(A) tails, rather than commenting on whether the method does not evaluate the start and end of the poly(A) tails in the event space. In fact, Dorado does measure the signal start and end to calculate the poly(A) tail. However, we have taken the reviewer's comments into account and described the lack of output in the discussion, but it is more likely that additional processing times influence execution times (paragraph 2 of discussion section).

Finally, the authors could elaborate on how these findings influence future tool development or integration into transcriptomics workflows.

A section of future directions for tool development and tool benchmarking has been added in lines 395-399: "Expanding the range of poly(A) lengths to better mimic the distribution in real samples, gaining an enhanced understanding of length-specific biases in each tool and including RNA from diverse preparation methods will enhance this study."

Note:

I previously reviewed this paper previously in Research Hub and you can read these comments via the Research Hub review page here: https://www.researchhub.com/paper/8634403/using-synthetic-rna-to-benchmark-polya-length-inference-from-direct-rna-sequencing/reviews#threadId=55398.

The original preprint linked to the Research Hub review is here: https://doi.org/10.1101/2024.10.25.620206.

--

Please also take a moment to check our website at https://www.editorialmanager.com/giga/l.asp?i=198534&l=HCIFXYLQ for any additional comments that were saved as attachments. Please note that as GigaScience has a policy of open peer review, you will be able to see the names of the reviewers.
