# Supplementary material for: Using synthetic RNA to benchmark poly(A) length inference from direct RNA sequencing
Source: Gigascience. 2025 Sep 3;14:giaf098. doi: 10.1093/gigascience/giaf098 (PMC12406214; doi:10.1093/gigascience/giaf098)
Supplement: giaf098_GIGA-D-24-00432_Revision_2 [file giaf098_giga-d-24-00432_revision_2.pdf]

GigaScience
Using synthetic RNA to benchmark poly(A) length inference from direct RNA sequencing
--Manuscript Draft--

**Manuscript Number:**	GIGA-D-24-00432R2
**Full Title:**	Using synthetic RNA to benchmark poly(A) length inference from direct RNA sequencing
**Article Type:**	Technical Note
**Funding Information:**	National Health and Medical Research Council (GNT1195743) — Dr Lachlan Coin
**Abstract:**	Polyadenylation is a dynamic process which is important in cellular physiology, which has implications in mRNA decay rates, translation efficiency, and isoform-specific regulation. Oxford Nanopore Technologies direct RNA-sequencing provides a strategy for sequencing the full-length RNA molecule and analysis of the transcriptome. Several tools are currently available for poly(A) tail length estimation, including well-established methods like tailfindr and nanopolish, as well as more recent deep learning models like Dorado. However, there has been limited benchmarking of the accuracy of these tools against gold-standard datasets. In this paper, we present our novel deep-learning poly(A) estimation tool – BoostNano and compare with three existing tools - tailfindr, nanopolish and Dorado. We evaluate the four poly(A) estimation tools, using two sets of synthetic in vitro-transcribed RNA standards with known poly(A) tail lengths - Sequin (30 or 60 nucleotides) and enhanced Green Fluorescent Protein (10-150 nucleotides) RNA. Analyzing datasets with known ground truth values is a valuable approach to measuring the accuracy of poly(A) length estimation. The tools demonstrated length- and sample-dependent performance, and accuracy was enhanced by averaging over multiple reads via estimation of the peak of the density distribution. Overall, Dorado is recommended as the preferred approach due to its relatively fast run times, low mean average error and ease of use with integration with base-calling. These results provide a reference for poly(A) tail length estimation analysis, aiding in improving our understanding of the transcriptome and the relationship between poly(A) tail length and other transcriptional mechanisms, including transcript stability or quantification.
**Corresponding Author:**	Lachlan Coin<br>University of Melbourne<br>Melbourne, AUSTRALIA
**Corresponding Author Secondary Information:**	
**Corresponding Author's Institution:**	University of Melbourne
**Corresponding Author's Secondary Institution:**	
**First Author:**	Jessie J-Y Chang, PhD
**First Author Secondary Information:**	
**Order of Authors:**	Jessie J-Y Chang, PhD
	Xuan Yang, Masters of Bioinformatics
	Haotian Teng
	Jianshu Zhang
	Benjamin Reames
	Shuxin Zhang
	Vincent Corbin
	Lachlan Coin

Powered by Editorial Manager® and ProduXion Manager® from Aries Systems Corporation

**Order of Authors Secondary Information:**	
**Response to Reviewers:**	Dear Editor,<br><br>We sincerely appreciate the time and dedication of you and the reviewers to have spent on reviewing this manuscript.<br><br>Please kindly find our responses to reviewer #1's comments in blue below.<br><br>Reviewer reports:<br><br>Reviewer #1: In the revised version the authors present a significantly improved benchmark of tools to estimate polyA tail lengths in Nanopore direct RNA-sequencing data. While they include additional data, the full set is still lacking. Most important would be to include more on RNA004, as most readers will be mainly interested in this. Some of the newly integrated analyses are not properly explained, as outlined below. The authors now included a RNA004 data set containing the sequins with two different polyA tail lengths (30 and 60). For a full benchmark that is useful for the field, it would be however required to generate data with a greater dynamic range. On the one hand, the authors show some issues in detection of short polyA tails, on the other hand longer polyA tails are of biological significance.<br><br>We understand the importance of expanding the RNA004 dataset and agree with the reviewer that doing so would indeed allow an enhanced understanding of the poly(A) estimation by the different tools. For generating our RNA002 and RNA004 datasets, we have used Sequins which were available as a product. Unfortunately, we are unable to generate synthetic RNA with longer poly(A) tails as we do not possess the capability to do this in our own laboratory. Thus, we instead, expanded the dataset in the RNA002 data (10nt – 150 nt poly(A) tails), using publicly available datasets, as per the reviewer's original recommendations. Despite the shorter tails on the RNA004 datasets, we believe that the data is still useful for understanding the polyadenylome and particularly useful for mitochondrial, viral and plant RNA poly(A) tails, which are commonly within the ranges of the Sequin poly(A) tails. We have added a sentence in the limitations section to clarify this. "While the range for the RNA004 data may be shorter than biologically relevant in mammalian non-mitochondrial RNA, the range fits the expected lengths for mitochondrial, viral and plant RNA poly(A) tails. [PMID: 35982302, PMID: 33961822, PMID: 32330414, PMID: 40355874] (lines 387-389)<br><br><br>Is the new Figure 1C, first panel for GFP transcripts sequenced on RNA002 and analyzed with Dorado? How does such an analysis look for RNA004?<br><br>Yes, Figure 1C shows the eGFP transcripts sequenced on RNA002 and analysed with Dorado, which were publicly available. We have clarified this in the Figure 1C legends. Regrettably, we are unable to generate the same data for RNA004 as we lack capacity in our laboratory to generate synthetic IVT RNA.<br><br>Introduction: "This raw data is then converted into sequence data using a custom deep learning model, such as Dorado [46] or Chiron [47]". This is what a basecaller does. It is still misleading to mention Chiron as a single tool here but not mentioning Guppy or Rodan basecallers. I understand that it is used in BoostNano, but it should be mentioned only in this context or the other basecallers should be included in the list as well.<br><br>We have modified this statement and removed the basecaller examples (Dorado and Chiron):<br>"This raw data is then converted into sequence data using a custom deep learning model, via the use of basecallers." (lines 91-92)<br><br><br>The new Figures 3B, D, E need to be explained better. Why are the authors only focusing only on reads, where BoostNano estimates a polyA tail length < 10 nts? It would be sufficient to determine the number of As/ percent A at the read ends of truncated reads. Can the authors provide some whole transcriptome data analysis? This would have a much higher impact for the readers.

Powered by Editorial Manager® and ProduXion Manager® from Aries Systems Corporation

	The rationale for including Figure 3, is for understanding the smaller peaks (< 10nt) as seen by the distribution plots in all datasets and all poly(A) estimation methods, as this was an unexpected result. As we wanted to isolate the reads < 10 nt, we had to choose one method to base our threshold, and we chose BoostNano as it showed the most prominent first peak. As a benchmarking manuscript with a focus on synthetic RNA to use ground truth datasets, we believe that adding results using whole transcriptomes is out of the scope of this manuscript. While this may be beneficial for the readers, investigating the polyadenylome in an organism will likely require the amount of work to synthesize a separate publication, and may not aid deeply in terms of comparing the variation between the tools. We have recently published some work regarding the poly(A) distributions using RNA002 called with Dorado in human blood mRNA (PMID: 40355874) and have recognized that short poly(A) tails are also shown through these datasets (~<10 nt), highlighting that shorter poly(A) tails are also found in vivo. We wish to note that many of the datasets available using direct RNA-sequencing are derived from RNA002 and it is still important to understand the performance differences suitable for RNA002 data. We have added the following future directions to the limitations section: "Overall, future work would benefit from expanding the range of poly(A) lengths to better mimic the distribution in real samples via synthetic and whole transcriptome data, gaining an enhanced understanding of length-specific biases in each tool and including RNA from diverse preparation methods." (lines 389-392).<br><br>The authors still argue that the RTA adapter may be degraded and now cite the work by Davis et al to support this. However, in this work they show that especially T-mers are stable over at least 70 freeze-and-thaw cycles. Based on the kit size of six reactions, the RTA should undergo maximal 5 freeze-and-thaw cycles and should be considered stable. Thus, I recommend to remove this statement.<br><br>We have removed this statement. In its place, we investigated the alternative idea of split-reads during the signal detection stage: "We hypothesized that poly(A) tails shorter than 10 nt may result from signal glitches during the signal detection, where one read may be written as multiple reads. Using Bulkvis, we discovered that among a total of 60,146 reads from all 7 RNA002 samples, 270 pairs of split reads were found (0.898%), with 26 pairs including one read in the list of reads with < 10 nt poly(A) tails (Data S4). This suggests that while read splits may partially explain the shorter poly(A) tails, other unexplained mechanisms are at play." (lines 269-274)<br><br>I don't understand Figures 3G and H. The authors select reads, for which BoostNano estimates very short polyA tails. For these reads, the other tools estimate polyA tail lengths which are closer to the expected length. Strictly spoken, this shows mainly that BoostNano should not be considered for estimating short polyA tail lengths.<br><br>The distribution plots show that the other tools (especially Nanopolish and Tailfindr) also exhibit a peak at a similar point as BoostNano if we take the maximum peak of density. However, we agree with the reviewer that BoostNano does indeed perform worst in terms of short poly(A) tails. We have added/amended to the following: "Our analysis revealed that while all four poly(A) estimation methods consistently identified shorter poly(A) tails, BoostNano exhibited a narrower peak for these shorter tails, whereas Dorado tended to estimate longer poly(A) tails that were closer to the known values (Figures 3g-h). Given that tailfindr and nanopolish also exhibited a peak at similar points in the density distributions as BoostNano, Dorado likely overestimates very short tails. Overall, the narrow peak of BoostNano indicates that BoostNano may not be suitable for estimating shorter poly(A) tails compared with the other tools." (lines 239-245).
**Additional Information:**	
**Question**	**Response**
Are you submitting this manuscript to a special series or article collection?	No
**Experimental design and statistics**	Yes

Powered by Editorial Manager® and ProduXion Manager® from Aries Systems Corporation

Full details of the experimental design and statistical methods used should be given in the Methods section, as detailed in our Minimum Standards Reporting Checklist. Information essential to interpreting the data presented should be made available in the figure legends.<br><br>Have you included all the information requested in your manuscript?	
**Resources**<br><br>A description of all resources used, including antibodies, cell lines, animals and software tools, with enough information to allow them to be uniquely identified, should be included in the Methods section. Authors are strongly encouraged to cite Research Resource Identifiers (RRIDs) for antibodies, model organisms and tools, where possible.<br><br>Have you included the information requested as detailed in our Minimum Standards Reporting Checklist?	Yes
**Availability of data and materials**<br><br>All datasets and code on which the conclusions of the paper rely must be either included in your submission or deposited in publicly available repositories (where available and ethically appropriate), referencing such data using a unique identifier in the references and in the "Availability of Data and Materials" section of your manuscript.<br><br>Have you have met the above requirement as detailed in our Minimum Standards Reporting Checklist?	Yes

Powered by Editorial Manager® and ProduXion Manager® from Aries Systems Corporation

Manuscript
Click here to access/download;Manuscript;Boostnano_paper_10_06_2025_FI

Using synthetic RNA to benchmark poly(A) length inference from direct RNA sequencing.

Jessie J-Y Chang[1], Xuan Yang[1], Haotian Teng[2], Jianshu Zhang[1], Benjamin Reames[1], Shuxin Zhang[1], Vincent Corbin[1]*, Lachlan Coin[1,3]*

1 Department of Microbiology and Immunology, University of Melbourne at The Peter Doherty Institute for Infection and Immunity, Melbourne, VIC, 3000, Australia

2 Ray and Stephanie Lane Computational Biology Department, School of Computer Science, Carnegie Mellon University, Pittsburgh, Pennsylvania, United States of America, 15213

3 Department of Clinical Pathology, University of Melbourne, Melbourne, VIC, 3000, Australia

*Corresponding authors: Vincent Corbin; Lachlan Coin

Abstract

Polyadenylation is a dynamic process which is important in cellular physiology, which has implications in mRNA decay rates, translation efficiency, and isoform-specific regulation. Oxford Nanopore Technologies direct RNA-sequencing provides a strategy for sequencing the full-length RNA molecule and analysis of the transcriptome. Several tools are currently available for poly(A) tail length estimation, including well-established methods like *tailfindr* and *nanopolish*, as well as more recent deep learning models like *Dorado*. However, there has been limited benchmarking of the accuracy of these tools against gold-standard datasets. In this paper, we present our novel deep-learning poly(A) estimation tool – *BoostNano* and compare with three existing tools - *tailfindr*, *nanopolish* and *Dorado*. We evaluate the four poly(A) estimation tools, using two sets of synthetic *in vitro*-transcribed RNA standards with known poly(A) tail lengths - Sequin (30 or 60 nucleotides) and enhanced Green Fluorescent Protein (10-150 nucleotides) RNA. Analyzing datasets with known ground truth values is a valuable approach to measuring the accuracy of poly(A) length estimation. The tools demonstrated length- and sample-dependent performance, and accuracy was enhanced by averaging over multiple reads via estimation of the peak of the density distribution. Overall, *Dorado* is recommended as the preferred approach due to its relatively fast run times, low mean average error and ease of use with integration with base-calling. These results provide a reference for poly(A) tail length estimation analysis, aiding in improving our understanding of the transcriptome and the relationship between poly(A) tail length and other transcriptional mechanisms, including transcript stability or quantification.

Keywords

Oxford Nanopore Technologies, poly(A) tail, estimation, segmentation, direct RNA-sequencing

Findings

Background

Polyadenylation is a co-/post-transcriptional process in which a string of adenine nucleotides is added to the 3’ of nascent messenger RNA (mRNA) molecules by enzymes such as polyadenylate (poly(A)) polymerases (PAPs) [1-3]. In eukaryotes, the polyadenylation process begins through the recognition of the poly(A) signal (PAS) situated within the 3’ untranslated region (UTR) of the mRNA [4]. This is a 6 nt sequence motif – commonly ‘AAUAAA’, located approximately 10-30 nt upstream of the poly(A) tail [5]. The polyadenylation process is mediated by the Cleavage and Specificity Factor (CSF) complex, which is made up of four major subunits – Specificity Factor (SF), Cleavage Stimulation Factor (CstF) and Cleavage Factors I and II (CFI & CFII). The SF recognizes the poly(A) signal and is required for specific cleavage and polyadenylation [6-10]. Additionally, CFI and CFII are required for accurate cleavage, and CstF enhances efficient cleavage at the poly(A) site and for a proportion of cases, PAP is required [8, 11]. PAP extends the poly(A) tail, stimulated by CSF and Poly(A) Binding Protein II (PABP II) within the nucleus [12, 13]. After the 5’ capping, splicing and polyadenylation, the mRNA is exported out of the nucleus into the cytoplasm. Here, the poly(A) tail is regulated by various deadenylase complexes – including CCR4-NOT [14] and PAN2-PAN3 [15]. Traditionally, the eukaryotic non-mitochondrial mRNA poly(A) tail has been regarded to be on average ~150-200 nt [16], which is more associated with the initial polyadenylation stages in the nucleus. With the involvement of deadenylation in the cytoplasm, the steady state of poly(A) tails has been identified to be shorter (~50-100 nt) [17, 18]. It has also been noted that non-adenine bases can be found within poly(A) tails as well as internal poly(A) sites [19].

Polyadenylation is thought to increase the stability of the mRNA molecule [20], assist in export of the molecule from the cell nucleus [21] and plays a role in RNA circularization, which may enhance efficient translation of cellular mRNAs [22]. This process is increasingly recognized as a dynamic process [23] which influences timing and degree of protein production [24, 25]. Furthermore, it is implicated in mRNA decay rates and regulation of gene expression [26, 27]. Poly(A) tails are also regarded to be dynamic in viral RNA, such as in the bovine coronavirus [23]. Currently, an ample number of studies have explored alternative polyadenylation (APA) [28-31] – the alternative usage of poly(A) sites which leads to variable 3’ ends of transcripts derived from the same gene. However, this mechanism is commonly confused with the study of poly(A) tail lengths and the latter is comparatively underexplored. As such, it is critical to be able to measure polyadenylation accurately using a high-throughput assay, which has the potential to enhance our understanding of the poly(A) tail length and its connections to other transcriptional and translational mechanisms.

Most existing literature on measuring the poly(A) length have utilized techniques such as polymerase chain reaction (PCR) [23, 32], northern blotting [33] or short-read poly(A) tail measurements such as PAL-seq [34] or TAIL-seq [17], which have clear limitations in terms of breadth of whole-transcriptome-wide detection, lengths and also arduous experimental efforts. In contrast, Oxford Nanopore Technologies

(ONT) direct RNA-sequencing is a simple approach for single-molecule RNA-sequencing which does not require reverse transcription (other than for RNA stabilization and improving sequencing output) or PCR amplification, thus avoiding amplification bias and retaining the original base and base-modification information [35-39]. It is worth noting that the full-length cDNA synthesis step, while not required, is recommended and the library preparation method utilizes a polythymine (poly(T))-containing adapter for sequencing. Furthermore, full-length RNA molecules can be captured in one read, facilitating the identification of complex splicing patterns, RNA modifications and RNA secondary structures [40-45]. The Nanopore sequencer records changes in ionic current as RNA passes through the pore in a custom FAST5/POD5 file. This raw data is then converted into sequence data using a custom deep learning model, via the use of basecallers. Although the majority of currently available public datasets have been generated from the SQK-RNA002 Direct RNA Sequencing kit, an updated version was released via early access in November 2023 (SQK-RNA004) as well as direct RNA-specific flow cells (FLO-MIN004RA or FLO-PRO004RA). The improvements include a faster motor protein, an RNA-specific reader pore, enhanced RNA models in the *Dorado* basecaller and an optimized library preparation method. Notably, recent iterations of *Dorado* have included the ability of RNA modification detection and poly(A) length estimations, which were only possible via third-party tools in previous years. Hence, Nanopore sequencing of native RNA provides an attractive approach for measuring single-molecule transcriptome-wide poly(A) tail length.

There have been several tools developed for estimating poly(A) tail length from raw Nanopore signal (**Table 1**), including *nanopolish* [46], *tailfindr* [42], *Dorado* (developed by ONT) [47] as well as our in-house tool *BoostNano* (details described in **Supplementary Information** and **Figures S1-3**, biotools:boostnano**,** RRID:SCR_026467) [48]. The tools detect the boundaries of poly(A) tails in varied ways. *tailfindr* identifies potential poly(A) stretches based on two rounds of defining the poly(A) tail segment, first determining rough poly(A) boundaries by thresholding the smoothened signal using a sliding window. Then, the second stage computes the mean of every 25 samples of clipped signal, and shrinks the rough poly(A) boundaries via confining the raw signal slopes. *nanopolish* utilizes a Hidden Markov Model (HMM), where each region of the read – the sequencing adapter, RTA, poly(A) tail and coding transcript each has one state contained by the HMM, in which these regions are linked sequentially, through linear-chain state transitions. Each section is deemed to have a unique emission distribution, which can be modeled by the HMM and applied on each read. *Dorado* utilizes a sliding window approach to find signal characteristics, initialized by identifying the RNA adapter sequence and determining the signal anchor point (i.e. the start of the poly(A) tail). The boundaries of the poly(A) tail are identified via analyzing around the proximity of the anchor point and understanding regions of the signal with low variance and similar mean values. *BoostNano* considers each region of the read as states like *nanopolish*. The neural network combines previous hidden states with current signal estimates to predict the signal's state, performing segmentation (more details can be found in **Supplementary Information**). However, there have been limited attempts to benchmark poly(A) tail length inference using gold-standard datasets with known poly(A) tail lengths, and comparisons between the two most-recent kit versions – RNA002 and RNA004.

Table 1. Summary of each poly(A) tail estimation tool benchmarked in this study.

Tool	Description	Reference
BoostNano	Convolutional Neural Network (CNN)-Recurrent Neural Network (RNN)-Connection-ist Temporal Classification (CTC) architecture from *Chiron* basecaller used to find boundaries of poly(A) in raw signal, basecalling not required	Teng et al., 2018. [48]
tailfindr	*R* tool, which uses the unaligned raw FAST5 data to estimate the poly(A) lengths via using the raw signal slope to refine the boundaries of potential poly(A) stretches and normalization with the read-specific nucleotide translocation rate, basecalling required for obtaining basecalled FAST5 with Events/Move table	Krause et al., 2019 [42]
nanopolish	Utilizes a predictive model in which a hidden Markov model (HMM - performs segmentation of the raw sequencing signal) and an estimator of the translocation rate are combined, basecalling required for obtaining input FASTQ	Simpson et al., 2017 [46]
Dorado	Searches for the boundaries in the raw signal and estimates the poly(A) tail length by considering the samples/base information, with adjustment for overestimation of the poly(A) tail. Primarily a basecalling tool, incorporates the poly(A) tail estimation during the basecalling itself	Oxford Nanopore Technologies [47]

For this study, we utilize two classes of ground-truth datasets derived from **1)** RNA Sequins – synthetic *in vitro*-transcribed (IVT) RNA, transcribed from an artificial chromosome which comprises 78 gene loci split into two classes, having either a 30 nucleotide (nt) (R1) or a 60 nt (R2) poly(A) tail (BioProject: PRJNA675370) [44, 49], and **2)** IVT RNA using enhanced Green Fluorescent Protein (eGFP) constructs with a wider range of poly(A) lengths (10, 30, 40, 60, 100 and 150 nts) from the authors of *tailfindr* (ENA Project: PRJEB31806) [42]. Thus, in this study, we compare the commonly-used poly(A) tail length estimation tools (*Dorado*, *tailfindr* and *nanopolish*) along with our own novel tool – *BoostNano* (released for the first time via this technical note) in hopes to understand and disseminate information to the wider community regarding the most appropriate tool for poly(A) length estimation.

Performance evaluation between *BoostNano, tailfindr, nanopolish* and *Dorado*

To compare the estimation performance of *BoostNano*, *tailfindr* v1.4, *nanopolish* v0.13.3 and *Dorado* v0.9.0, we tested these tools on two Sequin testing sets with known poly(A) tail lengths: R1 set with 30 nt tails and R2 set with 60 nt tails, as well as eGFP synthetic RNA with poly(A) tails ranging (10-150 nt) (**Figures 1a-c & Data S1**) [49]. Firstly, to estimate the accuracy of each method, we visualized the

density distributions for each dataset (**Figures 1a-c**). The four methods displayed a similar pattern in the density distribution, with a prominent normal-like peak near the expected poly(A) length, but also with an over-representation of shorter poly(A) tails, ranging at approximately ~0-20 nt (**Figures 1a-b**). For R2 Sequins, we observed a clear multi-modal distribution in all tools, with a trimodal distribution with *BoostNano* (**Figure 1b**). In contrast, the R1 Sequin estimates presented with either a multi-modal distribution (*nanopolish* and *BoostNano)* or a shoulder peak adjacent to the main peak, which was least evident in RNA004 *Dorado* data (**Figure 1a**). In the eGFP datasets, the bimodal distributions appeared mostly in datasets with ≥40 nt poly(A) tails (**Figure 1c**). *BoostNano* showed trimodal distributions in 60 and 80 nt datasets, and with an extreme overestimation with the 10 nt dataset. These results suggest that as poly(A) length increases, the distribution becomes more likely to be multi-modal, making means and medians potentially misleading as measures of average poly(A) length.

We then attempted to obtain a single estimate of tail length from the distributions in **Figure 1** for each tool and each known tail length. We used two approaches, the first a simple median, which is understood to be robust to deviations from normality. However, given the multi-modal nature of the distributions, we also tried to estimate the value which maximized the probability density function, which we call 'maxpeak' (**Data S1**). We investigated the difference between these estimates and the known values (**Figure 2a**), revealing a tendency for tools to overestimate short tails, particularly for the eGFP dataset. This analysis also revealed that the max-peak approach provided more accurate estimates than the median approach. *Dorado* showed less length-dependent error than other methods, particularly combined with maxpeak estimation of tail length. We also observed that correlation between methods increased as the number of reads included in the maxpeak statistic increased (**Figures S4-S6**). We investigated the width of the main peak in the probability distribution function, by estimating its standard deviation from full width at half-maximum (**Figure 2a**). As expected, this width increased as the known tail length increased. *BoostNano* was observed to have the tightest peak (meaning that more reads had values close to the maxpeak value).

Next, we investigated the accuracy of each tool at the read and grouped-read level. For read sets with 10 or more reads, we utilized either the median or maxpeak to generate an averaged estimate, as above. Then we calculated the Mean Absolute Error (MAE) between the estimated and known lengths (**Figure 2b, Figure S7, Data S2**). We observed that averaging over reads can lead to substantial improvements in accuracy (plateauing at 100 reads), with maxpeak providing more accurate estimates than using the median. Out of curiosity, we estimated the poly(A) tails of the pre-basecalled eGFP FAST5 files pulled directly from ENA Project, which had been basecalled by the authors of *tailfindr* with *Albacore* v2.3.3. Upon comparison with the other tools, we noticed that this dataset revealed the lowest MAEs, despite being the oldest ONT basecaller tested (**Figure 2b**). However, when we applied the same approach to Sequin datasets, the MAE was the highest out of all tools in the *Albacore*-basecalled dataset. Using bootstrap resampling, we calculated confidence intervals for each of the approaches (**Figure 2b**). We also used this resampling procedure to calculate whether MAE differences between tools were statistically significant and found that most (but not all) differences are significant ($p \leq 4.11 \times 10^{-06}$), as can be observed from the confidence intervals (**Figure 2b, Data S3**).

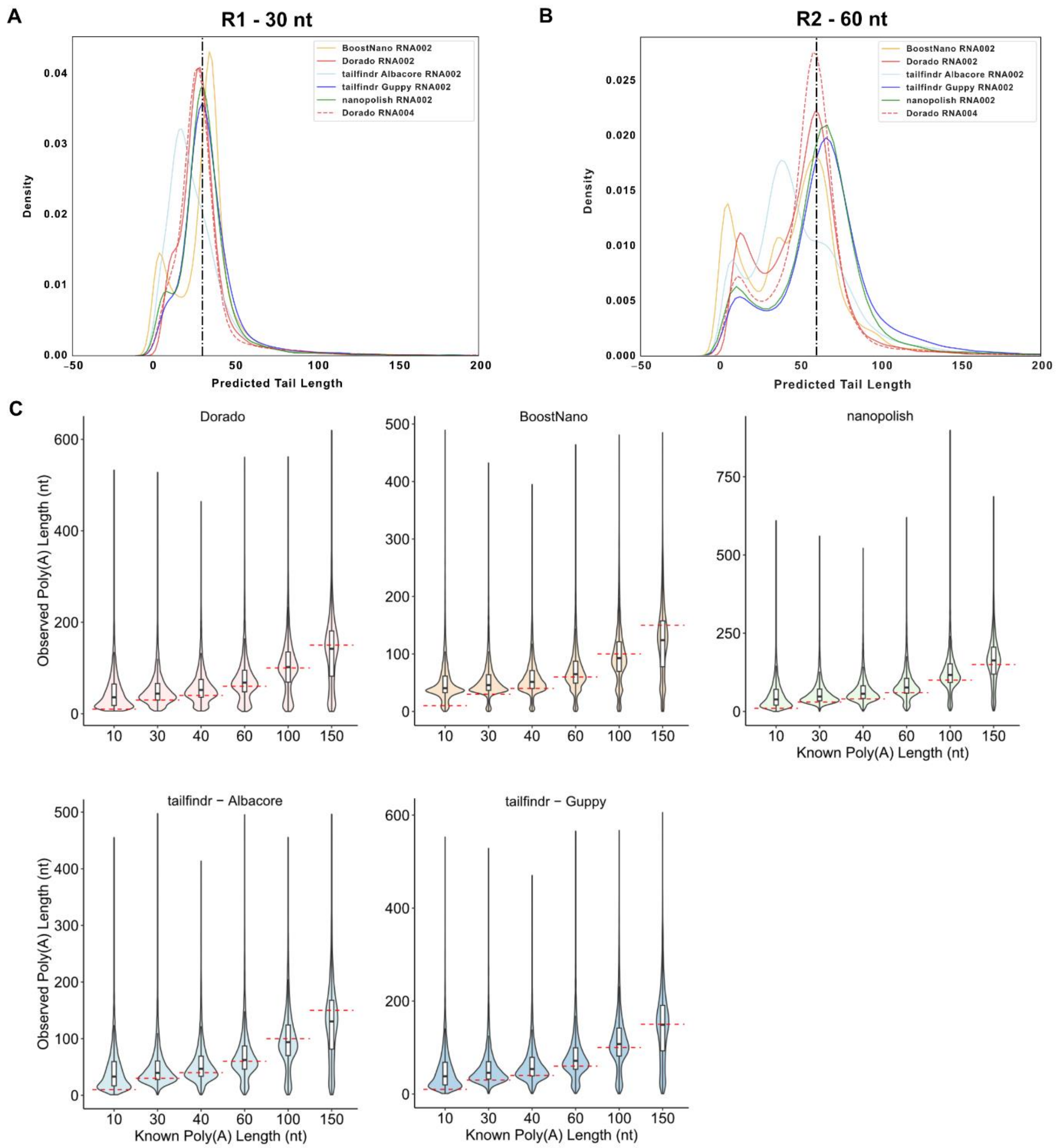

Figure 1. Poly(A) tail length estimates for each tool. (A-B) Predicted poly(A) tail length distributions for **A)** R1 (30 nt) and **B)** R2 (60 nt) Sequins. Outputs from *BoostNano* (yellow), *Dorado* (red), *tailfindr* (blue) and *nanopolish* (green). RNA002 (solid) and RNA004 (dashed) are shown as different line types. X-axis shows the predicted poly(A) tail lengths of all reads and Y-axis reveals the density of the poly(A) tail lengths. nt – nucleotide. Black vertical dashed lines indicate the known lengths. **C)** RNA002 poly(A) tail estimates of eGFP synthetic RNA from the study by Krause et al. [42], ranging between 10-150 nt in poly(A) length. Red dashed lines indicate the corresponding known poly(A) lengths.

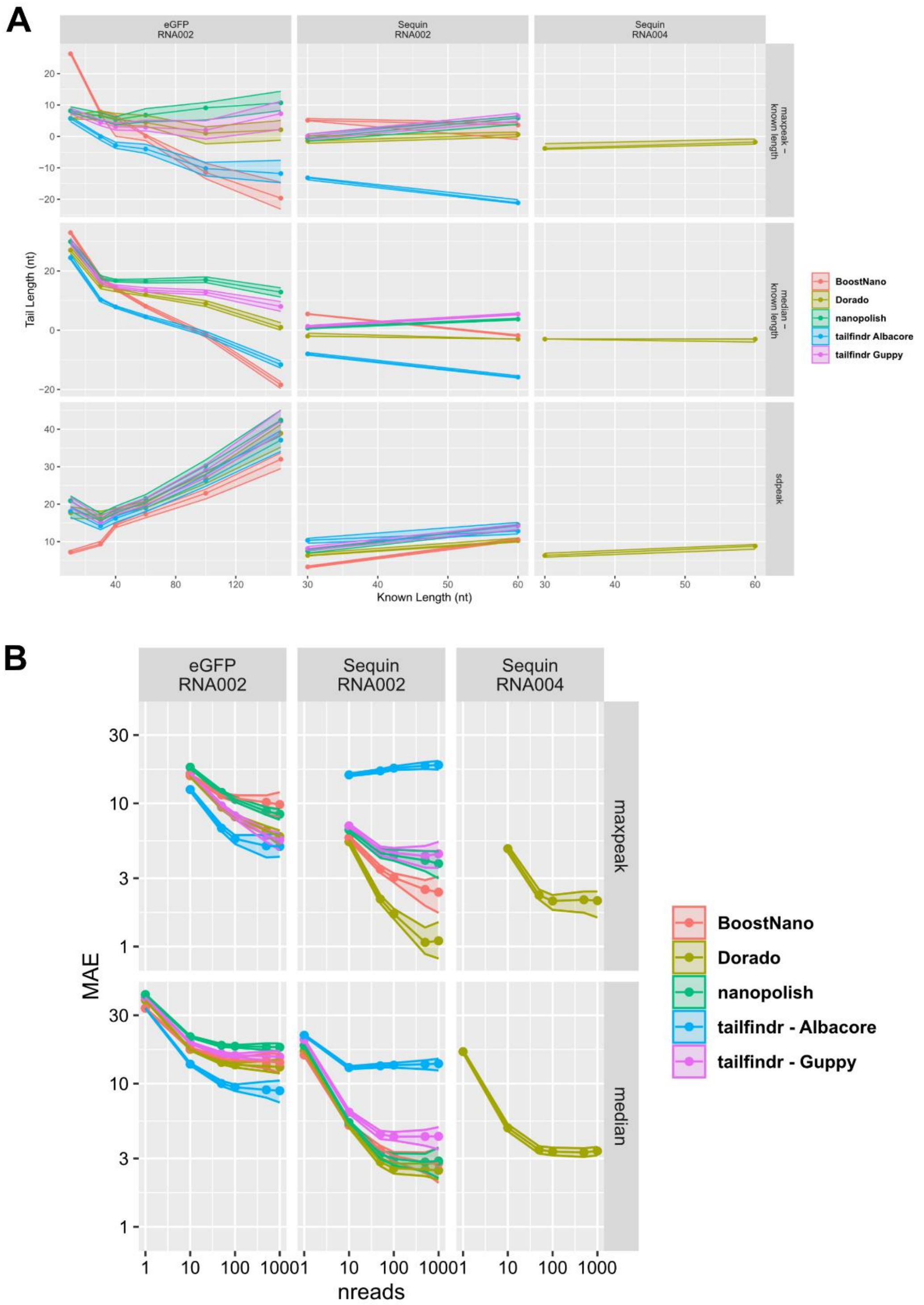

Figure 2. Tail length differences compared with known lengths and Mean absolute error (MAE) per dataset and tool. A) Tail length differences in nt of maxpeak and medians compared with the known length across the full range of known lengths. X-axis represents the known length (10 nt – 150 nt for eGFP, 30 nt / 60 nt for Sequins) and Y-axis represents difference between estimated and known tail length (rows 1,2) or the estimated standard deviation of the peak (row 3). 95% confidence intervals are shown as ribbons. **B)** Mean absolute error (MAE) of estimates of tail length across windows of nreads utilizing either maxpeak or median. The MAE is calculated as average of absolute difference to known lengths across all windows. 95% confidence intervals are also shown in ribbon form. X-axis represents the window size, and Y-axis represents the MAE.

To further test each method's ability to call poly(A) tails, we calculated the number of reads detected with the same number of input reads from the eGFP data, which were 592,571 reads (**Table S1**). We found that *BoostNano* detected the greatest number of reads and *nanopolish* detected the least number of reads in total, reads with poly(A) tails as well as reads aligned to the eGFP barcodes (maximum 96,403 reads), highlighting the high sensitivity of *BoostNano*.

We then proceeded to further understand the smaller peaks of the density distributions, which were present in almost all datasets (**Figures 1a-c**). This peak was more prominent at ~0-5 nt in *BoostNano*, whereas the early peaks for *tailfindr*, *nanopolish* and *Dorado* were positioned at ~5-20 nt. We hypothesized that these shorter peaks were derived from either **1)** fragmentation of the transcript, **2)** mis-priming of internal poly(A) stretches, or **3)** degradation of the poly(A) tails. To test this, we inspected reads with <10 nt poly(A) tails (as measured by *BoostNano*) and observed that the majority (~62.2%) aligned within 20 nt of the 3' end of the Sequin reference transcripts (**Figures 3a & b**). This suggested that most of these shorter poly(A) tails occurred due to hypothetical reason **3) -** fragmentation/degradation of the poly(A) tail, which is likely to be a sample integrity/preparation issue than an estimation defect. However, the remaining ~37.7% of reads showed truncations in the reference transcript (**Figure 3a**), consistent with hypothetical reasons **1)** fragmentation of the physical RNA or **2)** mis-priming. We wondered whether we could find any poly(A) stretches or high-adenine content in the sequences following the mapped 3' end of the truncated Sequin reads which would theoretically bind to the 10 poly(T)'s of the reverse transcription adapter (RTA) in the Direct RNA Sequencing kit. A high rate of these endings would correlate to high rates of mispriming (**Figure 3c**). To understand this phenomenon, we utilized the truncated dataset and isolated the 10 nt sequence following the end of the truncated Sequin reads according to the reference transcript. Then, we found the longest poly(A) stretch and the proportion of adenine bases in the 10 nt sequences. We observed that out of the truncated reads (3,088), only ~4.1% and ~1.6%, ~0.2%, ~0.03%, 0.03% of the reads had a poly(A) stretch of at least 4, 5, 6, 7, 8 adenines, respectively, with the longest poly(A) stretch being 8 adenines (**Figure 3d**). Furthermore, only ~4.73% of the reads contained high (>50%) poly(A) content in the 10 nt downstream of their ends (**Figure 3e**). Therefore, we were able to determine that reason 2 (i.e. mispriming) was unlikely to be the main reason for the presence of these truncated transcripts with short poly(A) lengths. Finally, we isolated all reads which did not meet any of the criteria listed above (high poly(A) content/at least 4 adenine stretches in 10nt downstream of mapped end and mapped within 20 nt of the 3' end of reference Sequin transcript, ~93.8%), and examined their average read quality scores, as we thought this may contribute to the shorter poly(A) tail (**Figure 3f**). We observed that surprisingly, most of the reads (~97.9%) showed average quality scores of >20, highlighting that poor read quality was not a prominent issue (**Figure 3f**). The overall Spearman correlation between poly(A) lengths and average read quality scores showed four datasets (*Dorado* R2, *BoostNano* R1, *BoostNano* R2, *tailfindr* R1) with weak but positive correlations ($r = 0.01\text{-}0.1$, $p < 0.05$) and *nanopolish* R2 dataset showing negative correlation ($r = -0.04$, $p = 6.6\text{e-}08$) (**Figure S8**). Therefore, we identified reads which were sequenced by direct RNA-sequencing which did not have proper poly(A) tails, nor mispriming events. Our analysis revealed that while all four poly(A) estimation methods consistently identified shorter poly(A) tails, *BoostNano* exhibited a narrower peak for these shorter tails, whereas *Dorado* tended to estimate longer poly(A) tails that were closer to the known values (**Figures 3g-h**). Given that *tailfindr* and *nanopolish* also exhibited a peak at similar points in the

density distributions as *BoostNano*, *Dorado* likely overestimates very short tails. Overall, the narrow peak of *BoostNano* indicates that *BoostNano* may not be suitable for estimating shorter poly(A) tails compared with the other tools.

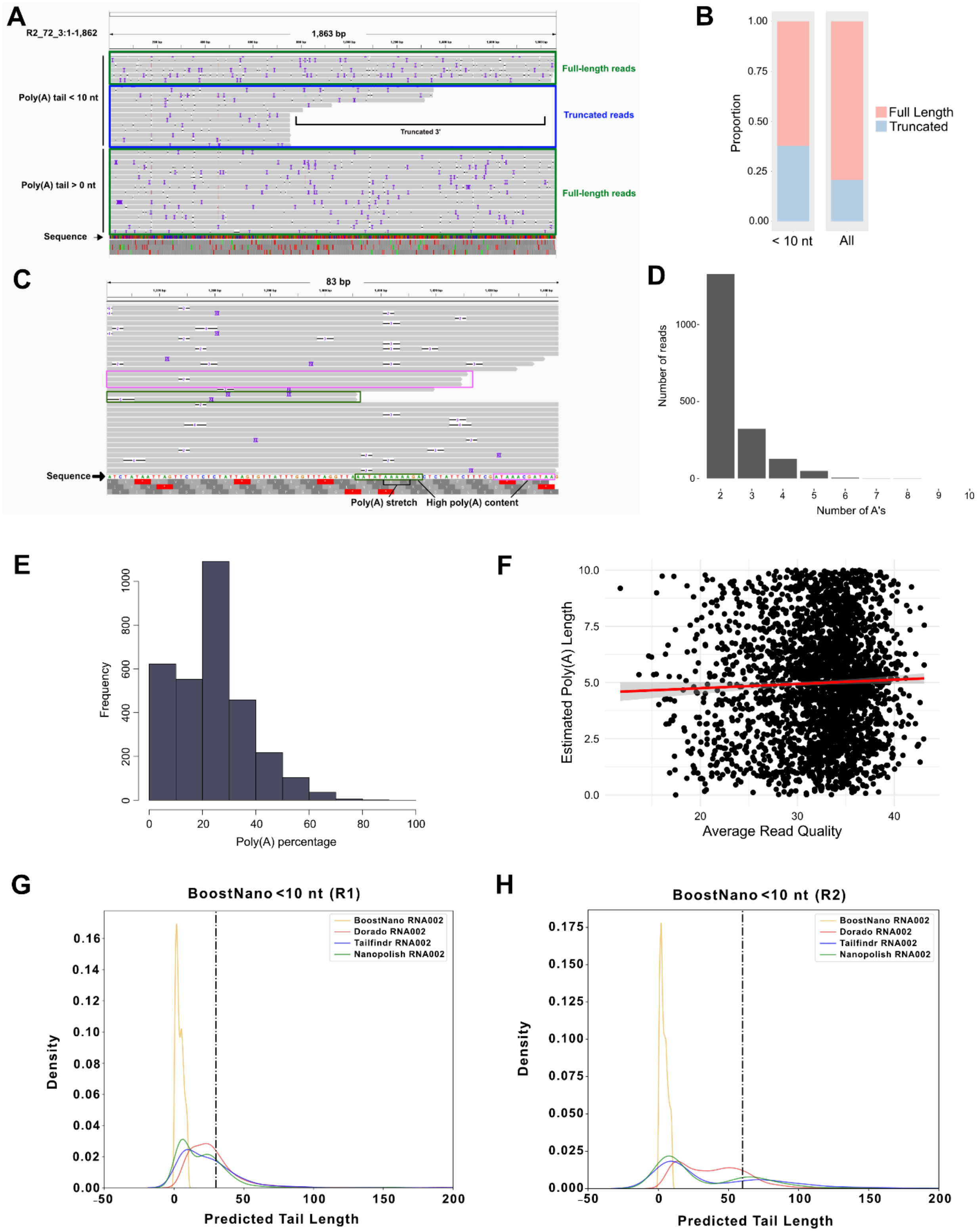

A
R2_72_3:1-1,862
1,863 bp
Poly(A) tail < 10 nt
Poly(A) tail > 0 nt
Sequence
Full-length reads
Truncated reads
Truncated 3'
Full-length reads
B
Proportion
1.00
0.75
0.50
0.25
0.00
< 10 nt
All
Full Length
Truncated
C
83 bp
Sequence
Poly(A) stretch
High poly(A) content
D
Number of reads
1000
500
0
2 3 4 5 6 7 8 9 10
Number of A's
E
Frequency
0 200 400 600 800 1000
0 20 40 60 80 100
Poly(A) percentage
F
Estimated Poly(A) Length
10.0 7.5 5.0 2.5 0.0
20 30 40
Average Read Quality
G
BoostNano <10 nt (R1)
BoostNano RNA002
Dorado RNA002
Tailfindr RNA002
Nanopolish RNA002
Density
Predicted Tail Length
H
BoostNano <10 nt (R2)
BoostNano RNA002
Dorado RNA002
Tailfindr RNA002
Nanopolish RNA002
Density
Predicted Tail Length

Figure 3. Poly(A) tails with short estimations < 10 nt. A) A representative subset of reads mapping to the R2_72_3 Sequin transcript visualized on the Integrative Genomics Viewer (IGV). The first subpanel shows a subset of reads with < 10 nt poly(A) tails (estimated by *BoostNano*), showing that reads with < 10 nt poly(A) tails are derived from both reads which have intact and fragmented 3' ends. The second panel shows a representative subset of full-length reads in the full dataset with any detected poly(A) lengths > 0 nt. Each grey line indicates a read. "Sequence" indicates the sequence of bases which form the transcript, where A = green, T = red, G = yellow and C = blue. nt – nucleotide. **B)** Proportion of reads with truncated vs full-length 3' ends in the entire combined Sequin RNA002 dataset and reads with poly(A) lengths < 10 nt (estimated by *BoostNano*). **C)** Truncated reads with poly(A) tails <10 nt (estimated by *BoostNano*) mapped to R2_65_1 Sequin transcript and 3' ends ending across an internal poly(A) stretch (green) and a stretch with high poly(A) content (pink). Each grey line indicates a read. "Sequence" indicates the sequence of bases which form the transcript, where A = green, T = red, G = yellow and C = blue. **D)** The number of adenines in the 10 nt stretch following the 3' end of truncated reads with < 10 nt poly(A) tails (estimated by *BoostNano*). **E)** Percentage of adenines in the 10 nt stretch following the 3' end of truncated reads with < 10 nt poly(A) tails (estimated by *BoostNano*). **F)** Average read quality scores vs poly(A) lengths in truncated reads with < 10 nt poly(A) tails (estimated by *BoostNano*), which do not meet thresholds of at least 4 adenines in series or 60 percent poly(A) content in the 10 nt following the 3' end of the mapped read. **(G-H)** Density plots of estimated read-lengths with < 10 nt poly(A) tails (estimated by *BoostNano*) in all four tools in the Sequin **G)** R1 and **H)** R2 datasets.

The explanations above partially explain the earlier peak (~0-20 nt) in the density distribution (**Figure 1**) in all four methods, however, *BoostNano* particularly showed the mode of the peak presenting at even shorter poly(A) tail lengths than *tailfindr*, *nanopolish* and *Dorado*. As the ONT RTA used for reverse transcribing the native RNA strand has 10 poly(T) bases, it is likely that the minimum detection limit of poly(A) tails is 10 nt, which matches the ~10 nt peak with *tailfindr*, *nanopolish* and *Dorado*. We hypothesized that poly(A) tails shorter than 10 nt may result from signal glitches during the signal detection, where one read may be written as multiple reads. Using *Bulkvis*, we discovered that among a total of 60,146 reads from all 7 RNA002 samples, 270 pairs of split reads were found (0.898%), with 26 pairs including one read in the list of reads with <10 nt poly(A) tails (**Data S4**). This suggests that while read splits may partially explain the shorter poly(A) tails, other unexplained mechanisms are at play. Interestingly, upon investigating these earlier peaks, we found that *Dorado* excludes reads retained in the analysis by *BoostNano*, even though the majority of these reads were considered to have high read quality (**Figures 4a & 4b**). While an earlier peak of <10 nt was the most prominent amongst reads discarded by *Dorado*, we also observed peaks of ~40 nt and ~60 nt (according to *BoostNano*) amongst reads discarded by *Dorado*. As mentioned above, *BoostNano* resulted in the greatest number of reads with detected poly(A) tails compared with the other three methods, including *Dorado* (**Table S1**). Thus, *Dorado* demonstrates a more conservative approach compared to *BoostNano*.

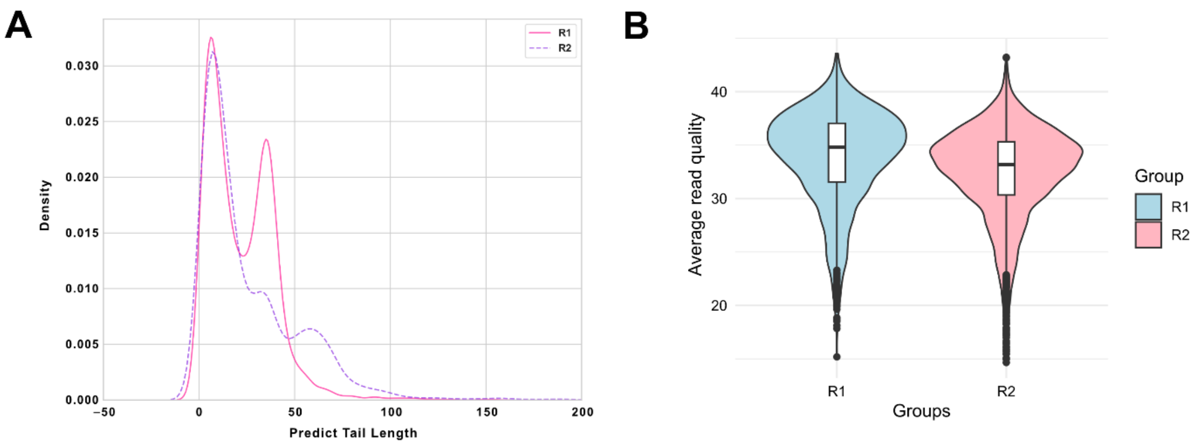

Figure 4. **Reads which have been filtered out by *Dorado* but retained in the *BoostNano* output. A)** R1 Sequin reads are indicated in a solid pink line (known length = 30 nt), and R2 Sequin reads are indicated in a dashed purple line (known length – 60 nt). X-axis shows the predicted poly(A) tail length in nucleotides, and the Y-axis shows the density distribution. nt – nucleotide. **B)** Violin plots of average read qualities of the same reads in R1 and R2 Sequin groups.

Finally, we compared the computational time required by each method to predict the tail lengths of 4,000 reads. For *BoostNano* and *Dorado*, we used one graphics processing unit (GPU) with 16G allocated RAM, while for *nanopolish* and *tailfindr*, which doesn't have the option to be run on GPU, we used one central processing unit (CPU) with 16G RAM and 1 thread for *nanopolish*. *Dorado* and showed rapid computational times at just ~1 m 10 seconds (s), whereas *BoostNano* revealed the longest computational time at ~16 m and 52 s (**Table 2**).

Table 2. Computational time efficiency to process 4,000 reads with 1 GPU/CPU. GPU - Graphics Processing Unit, CPU - Central Processing Unit, API – Application Programming Interface, s – seconds. Execution times include all pre-processing times such as basecalling and alignment.

Method	Execution time (4000 reads, incl. basecalling, alignment)	GPU/CPU	GPU/CPU Model	Processor	RAM	OS
BoostNano	16 m 52 s	1 GPU	80 GB A100 Nvidia GPU	Intel Xeon Gold	16 G	Linux
Dorado	1 m 10s	1 GPU	80 GB A100 Nvidia GPU	Intel Xeon Gold	16 G	Linux
tailfindr	10 m 56 s	1 CPU	Intel Xeon Gold 6254 CPU @ 3.10GHz	Intel Xeon Gold	16 G	Linux
nanopolish	2 m 5 s	1 CPU	Intel Xeon Gold 6254 CPU @ 3.10GHz	Intel Xeon Gold	16 G	Linux

Discussion

In this technical note, we assessed the predictive performance of four poly(A) tail length estimation tools - *tailfindr*, *nanopolish*, *BoostNano* and *Dorado* on three separate testing sets with known poly(A) tail lengths. This was explored via various methods including manual visualization of density plots, calculating read-level and grouped read-level MAE, calculating standard deviation of the probability density function, exploring the sensitivity of detection and execution time. When evaluating poly(A) lengths at the gene-level or transcript-level, the researcher may wish to utilize an average value to compare between different conditions. While medians are typically preferred over means due to the commonly skewed distribution in naturally occurring poly(A) lengths, our results highlighted that utilizing the maxpeak approach is more useful than the medians for read-sets with 10 reads or more (**Figures 2b & S4**). When using this method, we noted length- and sample-dependent rankings for the evaluated tools (**Figure 2a**, **Data S1**). Overall, *tailfindr* and *Dorado* were most accurate across the different lengths, and *BoostNano* performed poorest, with the exception of 60 nt tails, where *BoostNano* and *Dorado* performed well (**Data S1**). Length-dependency of error rates is important to consider as polyadenylation research pertains mostly to mammalian (e.g. human) mRNA, and more recently with viral RNA (e.g. Severe Acute Respiratory Syndrome Coronavirus-2 (SARS-CoV-2)). In mammals, poly(A) tails on mRNA undergo an initial synthesis stage which increases to 100-200 nt, and once localized out of the nucleus, the poly(A) tail is subject to a deadenylation by CCR2-NOT and PAN2-PAN3 deandenylase complexes, in which the steady state average poly(A) length is approximately ~50-

100 nt [17]. The exceptions are in mitochondrial RNA, which have average poly(A) lengths of ~40-50 nt [50]. Furthermore, poly(A) lengths on SARS-CoV-2 RNA have been found to be approximately 45-60 nt [40, 44]. The implications of poly(A) estimation outputs can lead to different varied interpretations. If a method overestimates poly(A) tails, the researcher may overestimate other associated functions such as RNA stability, since longer poly(A) tails are commonly associated with greater stability. This is particularly detrimental when exploring differential polyadenylation, as even small changes in poly(A) tails may influence statistical tests. Thus, as a general, we recommend the following; should the researcher have specific expected tail lengths for their study, they should choose the most appropriate tool based on the results of this study or similar, with the use of maxpeaks to average over read sets (N>100). In more complex transcriptomes with wider variety of tail lengths, *tailfindr* or *Dorado* should be utilized.

Another factor which may be important to the researcher is time of execution. We observed that *Dorado* and *nanopolish* surpasses the rate of *BoostNano* or *tailfindr* (**Table 2**). *BoostNano* and *tailfindr* tools provide estimation of the starting and ending positions of the poly(A) tails in event space. In contrast, *nanopolish* shows only the start positions and this information is available from *Dorado* verbose logs. *nanopolish* and *tailfindr* require additional processing such as FAST5 basecalling and mapping, which also contribute to greater overall execution times. In light of these findings, we anticipate the adoption of *Dorado* as the default method for poly(A) tail estimation, given its rapid estimation timeframe, comparable accuracy to other tools, conservative nature, and ease of integration with basecalling. One thing to note is that the community has noted remarkable differences in the performance of poly(A) tail measurements depending on the version of *Dorado* utilized, especially with versions prior v0.5.3 having caused notable issues. Regardless, the current state of *Dorado* has favorable properties for the general user. We note that while *Dorado* is identified as the preferred tool, the researcher may prefer accuracy over time, especially in the case of low-throughput datasets. In this case, other methods like *tailfindr* may be implemented according to specific contexts.

The density distributions for the poly(A) lengths were clearly multi-nomial for most datasets, and this was more pronounced in longer poly(A) tail datasets (**Figures 1a-c**). This phenomenon can be explained by the fact that there is a greater possibility for fragmentation for longer poly(A) tails, which can cause shorter than expected tail length peaks. This has also been noted in the *tailfindr* publication, although the commentary was referencing the cDNA data, instead of the RNA data [42]. Secondly, the lower peaks were particularly minimal in *Dorado* Sequin R1 datasets, which may be explained by its conservative nature in filtering out certain reads which were retained in other tools (**Figure 4a**). *Dorado*'s conservative nature might be due to the tendency of the tool to base its estimation on searching for a low variability region near an anchor point, and if such regions are undefined, this may lead to an omission of the reads. In contrast, as *BoostNano* detected the greatest number of poly(A) tails compared to all tools, we may attribute its unusual trimodal distribution to this reason. Early peaks in the poly(A) length density distribution (defined as <10 nt as measured by *BoostNano)* comprised of ~68% of reads potentially affected by fragmentation/degradation of the poly(A) tail, and approximately 1/3 of these reads were deemed truncated and could not be accounted for by fragmentation/degradation of the poly(A) tail

(**Figure 3b**). Upon further investigation, ~94% of these unaccounted reads were not attributed mispriming due to poly(A) stretches or poly(A)-rich regions in the 10 nt downstream of the 3' end of the read (**Figures 3d-e**). Incorrect segmentation also only attributed to 26 read-pairs (**Data S4**). It is currently unclear to us how these reads were able to be sequenced if the reads supposedly lacked a poly(A) tail due to truncation in the middle of the read. If truncations did occur, the 5' end of the transcript as opposed to the 3' end would be lacking. We do not believe this is due to mapping issues as reads with high-quality mappings were isolated, and we utilized synthetic RNA with well-defined references. Although seemingly unlikely, these types of reads may only be sequenced if somehow the reads were able to enter the pores without the RTA or sequencing adapters. Therefore, we suspect potential sequencing errors, or incorrect adapter ligation. Further work will be required to elucidate this phenomenon, but nevertheless, this emphasizes the importance of good RNA integrity of the input RNA.

Our study emphasizes the importance of obtaining sufficient coverage of each transcript in order to take advantage of improved poly(A) tail length estimation accuracy via averaging. We recommend obtaining at least 100x coverage of each target transcript to acquire a reliable estimate of poly(A) tail length via the maxpeak approach.

One of the limitations of this study is that we have only utilized synthetic RNA, for the purposes of procuring a ground truth. Researchers will aim to use poly(A) estimation tools for mainly real samples with more complex transcriptomes, including varying GC content, transcript and poly(A) lengths, modified bases, and coding potential unlike the synthetic RNA we explored in this study. From our understanding from previous studies regarding poly(A) lengths, we hypothesize that the poly(A) tail distribution will vary depending on these different factors. The results we reveal in this study helps understand biases observed via the use of different tools, in a controlled setting, without the effect of these variations. While this does not fully encapsulate the variability that may be seen with real samples, we hope that this data is useful for extrapolating which tool may suit the researcher best for their samples. Furthermore, generating a reliable ground truth dataset for real samples across the transcriptome is exceedingly difficult, if not unattainable. Secondly, we have employed a combined dataset of 7 separate sequencing runs containing Sequins as well as one eGFP dataset for RNA002 datasets and 6 sequencing runs for RNA004 datasets, with a lack of sequencing replicates. Potential batch effects may arise when visualizing complex transcriptomes with one sequencing replicate, which is common in many ONT direct RNA-sequencing studies. Thus, this study may be extended via the use of such replicates. Our study is limited to synthetic RNA which are limited in poly(A) length of 10 – 150 nt for RNA002 data and 30 and 60 nt for RNA004 data. While the range for the RNA004 data may be shorter than biologically relevant in mammalian non-mitochondrial RNA, the range fits the expected lengths for mitochondrial, viral and plant RNA poly(A) tails [40, 44, 51, 52]. Overall, future work would benefit from expanding the range of poly(A) lengths to better mimic the distribution in real samples via synthetic and whole transcriptome data, gaining an enhanced understanding of length-specific biases in each tool and including RNA from diverse preparation methods.

In conclusion, this work demonstrates the value of synthetic RNA molecules with known poly(A) tail lengths for validating poly(A) tail estimation algorithms. As methods improve, we anticipate that these datasets will be valuable for assessing advancements in poly(A) tail estimation. *Dorado* proves to be highly efficient and accurate amongst the four tools we explored in this study. Thus, we recommend the

use of this approach when performing poly(A) length analyses via implementing the maxpeak values and window averaging strategy.

Methods

Datasets

RNA002

This study utilized publicly available ONT direct RNA-sequencing datasets involving SARS-CoV-2-infected continuous cell lines (Vero, Calu-3 and Caco-2) derived from our previous study, with synthetic RNA - Sequins (BioProject: PRJNA675370) [44]. Briefly, Vero (African green monkey kidney epithelia), Calu-3 (Human lung adenocarcinoma epithelia) and Caco-2 (Human colorectal adenocarcinoma epithelia) cells were cultured in 6-well tissue culture plates at 37°C, 5% (v/v) CO_2. The Australian ancestral strain of SARS-CoV-2 (SARS-CoV-2/human/AUS/VIC01/2020) was used to infect these cells at a multiplicity of infection (MOI) of 0.1 and the cells were harvested at 0, 2, 24 and 48 hours post-infection (hpi). The total RNA was extracted, treated with DNAse using Turbo DNA-free Kit (Invitrogen), and purified using the RNAClean XP magnetic beads (Beckman Coulter). 6 μg of total RNA for Vero cells and 3 μg of total RNA for Calu-3 and Caco-2 cells were pooled and 10% of expected mRNA (5% of total RNA) of Sequins [49] were added to each sample pool. The RNA was sequenced using the ONT Direct RNA Sequencing kit (SQK-RNA002), on R9.4.1 flow cells via the ONT MinION/GridION. For the purposes of this study, infected datasets from 24 and 48 hpi from all three cell lines and additionally 2 hpi from Vero cells were analyzed.

RNA004

Calu-3 cells were grown in 6-well tissue culture plates until 80-90% confluency and infected with 3 x Australian strains of Delta, Omicron (XBB1.5) and Omicron (JN.1) SARS-CoV-2 virus in triplicate. The infected cells were incubated at 37°C, 5% (v/v) CO_2 and harvested at 4 days post-infection (dpi). The total RNA was extracted using the RNeasy Mini Kit (Qiagen), treated with Turbo DNA-free Kit (Invitrogen) and purified using RNAClean XP beads (Beckman Coulter). For the sequencing, 1 μg of final total RNA product + 5% of expected mRNA (5% of total RNA) of Sequin mixA were used as inputs. 6 x samples in total were sequenced – 2 x Delta, 3 x XBB1.5 and 1 x JN1 samples. The new ONT Direct RNA Sequencing kit (SQK-RNA004) was used to sequence the libraries with the following modifications to the reverse transcription step; the use of the Induro Reverse Transcriptase (New England Biolabs) and incubation at 20 min at 55°C, then 10 min at 70°C. The libraries were sequenced using the kit-specific flow cells (FLO-MIN004RA, ONT) and sequenced using the ONT MinION/GridION via *MinKNOW* v24.02.16 and live-basecalled using the *MinKNOW*-integrated version of *Dorado* v7.3.11. The Sequin reads can be accessed from Data Availability section.

Analysis

Basecalling and mapping

The minimum requirement for poly(A) estimation was FAST5 files. Firstly, *Dorado* v0.9.0, which is a basecaller itself, and *BoostNano*, based on the *Chiron* basecaller, utilized raw FAST5 files as inputs. *Dorado* poly(A) estimation was carried out during the basecalling with the '--estimate-poly-a' parameter. For *nanopolish* analyses, raw FAST5 files were used for the 'index' step, and *Dorado* v0.9.0 used to generate the FASTQ files for the poly(A) estimation step. *tailfindr* requires basecalled FAST5 files for analyses with basecall group information in the FAST5. *Guppy* v6.3.2 was utilized for generating the basecalled FAST5 files. *Dorado* basecalling was incompatible with *taifindr* analyses as basecalled FAST5 files were required for the analyses. We attempted to test *tailfindr* using FAST5 files converted from POD5 files, however, this generated empty results. All *Dorado* or *Guppy* basecalling was carried out using the basecalled via the 'rna002 70bps_hac@v3' model for the RNA002 datasets, and 'rna004_130bps_sup@v5.1.0' model for the RNA004 datasets. No sup basecalling was available for RNA002.

For RNA002 data, an initial isolation of Sequins read was carried out by using the live-basecalled FASTQ files merged (passed + failed) and mapped to the merged Ensembl GrCh38 human, SARS-CoV-2 (VIC01/Australia) and Sequin genomes using *minimap2* v2.26 via the parameters '-ax splice -un'. Using the read id's, FAST5 files were isolated. For both RNA002 and RNA004 data, Sequins (R1/R2) were assigned to the reads by basecalling the FAST5 data with *Dorado* v0.9.0 and mapping the resulting FASTQ to the Sequin transcriptome with *minimap2* v2.26 with the parameters '-ax map-ont'. The reads were filtered with *Samtools* v1.16.1 'view' function with the parameters '-h -F 2308 -q 20'. The Sequin POD5/FAST5 files were isolated based on this mapping. The isolated Sequin FAST5 datasets can be accessed from Data Availability section. The data was also additionally basecalled using *Albacore* v2.3.3, the first generation ONT basecaller. Interestingly, *Albacore* v2.3.3 did not have an RNA002-specific config file, so we utilized the RNA001 + FLO-MIN106 combination: r941_70bps_rna_linear.cfg in attempts to replicate the methods of the *tailfindr* publication.

For further analysis, we implemented IVT synthetic RNA002 datasets generated from eGFPs from the *tailfindr* publication, which is publicly available (ENA Project: PRJEB31806). FAST5 files which were downloaded directly were basecalled already via *Albacore* v2.3.3 by the *tailfindr* authors. We subsequently re-basecalled the data with *Dorado* v0.9.0 as well as *Guppy* v6.3.2 and carried out the rest of the analysis as per the Sequin analysis. For *nanopolish* analysis, the data was mapped to the pCS2 + eGFP genome from Addgene, using *minimap2* v2.26 with the parameters '-ax map-ont'. The reads downloaded were demultiplexed into different poly(A) tail lengths -- (10, 30, 40, 60, 100, 150nt) by using the *seqkit* v2.5.1 'grep' function with parameters '-s –p $barcode –R 1:120 –m 1' by searching for corresponding barcode sequences in the first 120 bp of the reads (one mismatching was allowed). Read IDs for demultiplexing can be accessed from Data Availability section.

Poly(A) tail length analysis

For poly(A) tail length estimations, *Dorado* v0.9.0, *BoostNano*, *tailfindr* v1.4 and *nanopolish* v0.13.3 were used with the parameters outlined in **Table 3** and can be accessed from Data Availability section.

Table 3. Parameters for analysis.

Tool	Version	Parameters	Default (Y/N)
Dorado	0.9.0	**Model:** rna004_130bps_sup@v5.1.0 for R004 rna002_70bps_hac@v3 for R002<br><br>--estimate-poly-a<br><br>Default: --poly-a-config	**Y**
BoostNano	N/A	**-i $1 -o $2 -m path/to/model --replace**	**Y**
tailfindr	1.4	**find_tails(fast5_dir = args[1], save_dir = args[2], csv_filename = "tails.csv", num_cores = 30, basecall_group=args[4])**	**Y**
nanopolish	0.13.3	**--reads=${FASTQ} --bam=${BAM} --genome=${REF} --threads=8**	**Y**

Poly(A) truncations were visualized with *Integrative Genomics Viewer (IGV)* v2.10.1 using data with < 10 nt poly(A) tails according to *BoostNano* and all mapped data. All poly(A) tail truncation investigations were carried out in *R* v4.4.0 and density plots using *Python* v3.10.4.

Execution time calculations

Method timings were carried out using a subset of 4,000 reads, derived from Vero 2 hpi datasets, which was used for all timings. Basecalling and poly(A) tail times were added for the overall execution time. For *tailfindr* and *nanopolish*, which requires basecalled FAST5 files and FASTQ files, respectively, *Guppy* v6.3.2 was utilized.

Average poly(A) tail length calculations

Maxpeak was calculated as the value at which the probability density distribution achieves its maximum value. The density function was estimated using the density function from the stats package in *R*. Maxpeak and medians were calculated using only reads which were able to be detected by all methods to ensure direct comparisons.

The MAE was calculated by calculating either the median or maxpeak of reads in each on-overlapping window of N reads and finding the absolute difference between this median and the ground truth value.

Then, the sum of these absolute values was divided by the number of windows. Only reads which were able to be detected by all tools were retained in the analysis. Bootstrapped T-tests were implemented to compare the accuracy of each tool and were carried out by re-sampling reads 1,000 times from the original dataset at random within each known length category (with replacement). Due to this finite number of resamples, the smallest possible non-zero p-value is 0.001. Therefore, any reported p-value of 0 should be interpreted as $p < 0.001$, indicating that the observed effect was consistent across all bootstrap iterations.

PolyA tail length effect by split reads

To detect split reads in the Sequin RNA002 reads, we first aligned Sequin reads from 7 samples from different cell types and timepoints (Caco (24, 48 hpi), Calu (24, 48 hpi), Vero (2, 24, 48 hpi)) to Sequin reference genome using *Minimap2* v.2.24 with parameters '-x splice -uf -k14'. Then we used *Dorado* v0.9.0 'summary' function to generate the basecalling summary files from ubams. Alignment report and basecalling summaries were input to *Bulkvis* (v2.0.1) [53] to find incorrectly split reads.

Supplementary files

Supplementary Information. Supplementary Information, Figures S1-8, Table S1.

Supplementary Data S1. General table of statistical metrics including medians, maxpeak, sdpeak, confidence intervals, and number of reads detected.

Supplementary Data S2. MAE results based on medians or maxpeaks for Sequin (RNA002, RNA004) and eGFP datasets per tool, averaged over window sizes N = 1, 10, 50, 100, 500, 1000.

Supplementary Data S3. Bootstrapped T-test results comparing MAE of each tool averaged over window sizes N = 1, 10, 50, 100, 500, 1000.

Supplementary Data S4. RNA002 read pairs split during incorrect segmentation detected by *Bulkvis*. Sequin reads from all RNA002 datasets were utilized - (Caco (24, 48 hpi), Calu (24, 48 hpi), Vero (2, 24, 48 hpi)).

Availability of supporting source code and requirements

Project name: BoostNano

Project home page: https://github.com/haotianteng/BoostNano

Operating system(s): Platform independent

Programming language: Python

Other requirements: Pytorch

License: Mozilla Public License, v. 2.0

Biotools: boostnano

RRID: SCR_026467

Data Availability

The datasets supporting the results of this article are available in the NCBI repository, RNA002 - BioProject: PRJNA675370. All additional supporting data are available in the GigaScience repository, GigaDB [54].

Declarations

List of abbreviations

Poly(A) - Polyadenylate

ONT – Oxford Nanopore Technology

PCR – Polymerase Chain Reaction

HMM – Hidden Markov Model

CNN - Convolutional Neural Network

RNN - Recurrent Neural Network

CTC - Connection-ist Temporal Classification

SD – Standard deviation

m – minutes

s – seconds

MOI – Multiplicity of Infection

hpi – Hours post-infection

UTR – untranslated region

MAE – Mean average error

mRNA – messenger RNA

IVT – *in vitro* transcribed

eGFP – enhanced Green Fluorescent Protein

Ethics approval and consent to participate

Not applicable.

Consent for publication

Not applicable.

Competing interests

LC has received funding from ONT unrelated to this work, as well as travel funding, also unrelated to this work.

Funding

This work was supported by a NHMRC-EU project grant (GNT1195743) to LC.

Authors' contributions

Conceptualization – J.J.-Y.C., H.T., V.C., L.C.

Methodology – H.T., V.C., L.C.

Software – H.T., V.C., L.C.

Validation - J.J.-Y.C., H.T., X.Y., V.C., L.C.

Formal analysis – J.J.-Y.C., X.Y., H.T., J.Z., B.R., V.C., L.C.

Investigation - J.J.-Y.C., X.Y., H.T., J.Z., B.R., S.Z., V.C., L.C.

Resources - J.J.-Y.C., H.T., V.C., L.C.

Data Curation - J.J.-Y.C., V.C., L.C.

Writing - Original Draft - J.J.-Y.C., X.Y., H.T., V.C., L.C.

Writing - Review & Editing - J.J.-Y.C., X.Y., H.T., J.Z., V.C., L.C.

Visualization – J.J.-Y.C., X.Y., H.T., J.Z., V.C., L.C.

Supervision – V.C., L.C.

Project administration – J.J.-Y.C., V.C., L.C.

Funding acquisition – V.C., L.C.

Acknowledgements

This research was supported by The University of Melbourne's Research Computing Services and the Petascale Campus Initiative.

Authors' information

Not applicable.

References

1. Darnell JE, Wall R and Tushinski RJ. An Adenylic Acid-Rich Sequence in Messenger RNA of HeLa Cells and Its Possible Relationship to Reiterated Sites in DNA. 1971;68 6:1321-5. doi:10.1073/pnas.68.6.1321.
2. Lee SY, Mendecki J and Brawerman G. A Polynucleotide Segment Rich in Adenylic Acid in the Rapidly-Labeled Polyribosomal RNA Component of Mouse Sarcoma 180 Ascites Cells. 1971;68 6:1331-5. doi:10.1073/pnas.68.6.1331.
3. Terns MP and Jacob ST. Role of poly(A) polymerase in the cleavage and polyadenylation of mRNA precursor. 1989;9 4:1435-44. doi:10.1128/mcb.9.4.1435.
4. Proudfoot NJ and Longley JI. The 3' terminal sequences of human alpha and beta globin messenger RNAs: comparison with rabbit globin messenger RNA. Cell. 1976;9 4 PT 2:733-46. doi:10.1016/0092-8674(76)90137-9.
5. Proudfoot NJ and Brownlee GG. 3′ Non-coding region sequences in eukaryotic messenger RNA. Nature. 1976;263 5574:211-4. doi:10.1038/263211a0.
6. Bardwell VJ, Wickens M, Bienroth S, Keller W, Sproat BS and Lamond AI. Site-directed ribose methylation identifies 2'-OH groups in polyadenylation substrates critical for AAUAAA recognition and poly(A) addition. Cell. 1991;65 1:125-33. doi:10.1016/0092-8674(91)90414-t.
7. Keller W, Bienroth S, Lang KM and Christofori G. Cleavage and polyadenylation factor CPF specifically interacts with the pre-mRNA 3′ processing signal AAUAAA. The EMBO Journal. 1991;10 13:4241-9. doi:10.1002/j.1460-2075.1991.tb05002.x.
8. Wilusz J, Shenk T, Takagaki Y and Manley JL. A multicomponent complex is required for the AAUAAA-dependent cross-linking of a 64-kilodalton protein to polyadenylation substrates. Molecular and Cellular Biology. 1990;10 3:1244-8. doi:10.1128/mcb.10.3.1244.
9. Christofori G and Keller W. 3' cleavage and polyadenylation of mRNA precursors in vitro requires a poly(A) polymerase, a cleavage factor, and a snRNP. Cell. 1988;54 6:875-89. doi:10.1016/s0092-8674(88)91263-9.
10. Gilmartin GM and Nevins JR. An ordered pathway of assembly of components required for polyadenylation site recognition and processing. Genes & Development. 1989;3 12b:2180-90. doi:10.1101/gad.3.12b.2180.
11. Takagaki Y, Ryner LC and Manley JL. Four factors are required for 3'-end cleavage of pre-mRNAs. Genes & Development. 1989;3 11:1711-24. doi:10.1101/gad.3.11.1711.
12. Wahle E. A novel poly(A)-binding protein acts as a specificity factor in the second phase of messenger RNA polyadenylation. Cell. 1991;66 4:759-68. doi:10.1016/0092-8674(91)90119-j.
13. Winters MA and Edmonds M. A poly(A) polymerase from calf thymus. Characterization of the reaction product and the primer requirement. J Biol Chem. 1973;248 13:4763-8.
14. Lau N-C, Kolkman A, Schaik V, A., M., Frederik, Mulder W, Klaas, Pijnappel P, M., W., W., Heck R, J., Albert, et al. Human Ccr4–Not complexes contain variable deadenylase subunits. Biochemical Journal. 2009;422 3:443-53. doi:10.1042/bj20090500.
15. Wolf J, Valkov E, Allen MD, Meineke B, Gordiyenko Y, Mclaughlin SH, et al. Structural basis for Pan3 binding to Pan2 and its function in mRNA recruitment and deadenylation. The EMBO Journal. 2014;33 14:1514-26. doi:10.15252/embj.201488373.
16. Edmonds M, Vaughan MH and Nakazato H. Polyadenylic Acid Sequences in the Heterogeneous Nuclear RNA and Rapidly-Labeled Polyribosomal RNA of HeLa Cells: Possible Evidence for a Precursor Relationship. 1971;68 6:1336-40. doi:10.1073/pnas.68.6.1336.

17. Chang H, Lim J, Ha M and Kim N, V. TAIL-seq: Genome-wide Determination of Poly(A) Tail Length and 3′ End Modifications. Molecular Cell. 2014;53 6:1044-52. doi:10.1016/j.molcel.2014.02.007.
18. Eisen TJ, Eichhorn SW, Subtelny AO, Lin KS, McGeary SE, Gupta S, et al. The Dynamics of Cytoplasmic mRNA Metabolism. Mol Cell. 2020;77 4:786-99 e10. doi:10.1016/j.molcel.2019.12.005.
19. Begik O, Diensthuber G, Liu H, Delgado-Tejedor A, Kontur C, Niazi AM, et al. Nano3P-seq: transcriptome-wide analysis of gene expression and tail dynamics using end-capture nanopore cDNA sequencing. Nature Methods. 2023;20 1:75-85. doi:10.1038/s41592-022-01714-w.
20. Beckel-Mitchener AC. Poly(A) Tail Length-dependent Stabilization of GAP-43 mRNA by the RNA-binding Protein HuD. The Journal of Biological Chemistry. 2002;277 31:27996-8002. doi:10.1074/jbc.m201982200.
21. Fuke H and Ohno M. Role of poly (A) tail as an identity element for mRNA nuclear export. Nucleic Acids Research. 2007;36 3:1037-49. doi:10.1093/nar/gkm1120.
22. Gallie DR. The cap and poly(A) tail function synergistically to regulate mRNA translational efficiency. Genes Dev. 1991;5 11:2108-16. doi:10.1101/gad.5.11.2108.
23. Wu H-Y, Ke T-Y, Liao W-Y and Chang N-Y. Regulation of Coronaviral Poly(A) Tail Length during Infection. PLoS ONE. 2013;8 7:e70548. doi:10.1371/journal.pone.0070548.
24. Kojima S, Sher-Chen EL and Green CB. Circadian control of mRNA polyadenylation dynamics regulates rhythmic protein expression. Genes & Development. 2012;26 24:2724-36. doi:10.1101/gad.208306.112.
25. Biziaev N, Shuvalov A, Salman A, Egorova T, Shuvalova E and Alkalaeva E. The impact of mRNA poly(A) tail length on eukaryotic translation stages. Nucleic Acids Research. 2024;52 13:7792-808. doi:10.1093/nar/gkae510.
26. Passmore LA and Coller J. Roles of mRNA poly(A) tails in regulation of eukaryotic gene expression. Nature Reviews Molecular Cell Biology. 2022;23 2:93-106. doi:10.1038/s41580-021-00417-y.
27. Lima SA, Chipman LB, Nicholson AL, Chen Y-H, Yee BA, Yeo GW, et al. Short poly(A) tails are a conserved feature of highly expressed genes. Nature Structural & Molecular Biology. 2017;24 12:1057-63. doi:10.1038/nsmb.3499.
28. Mayr C and Bartel DP. Widespread Shortening of 3′UTRs by Alternative Cleavage and Polyadenylation Activates Oncogenes in Cancer Cells. Cell. 2009;138 4:673-84. doi:10.1016/j.cell.2009.06.016.
29. Huang G, Huang S, Wang R, Yan X, Li Y, Feng Y, et al. Dynamic Regulation of Tandem 3' Untranslated Regions in Zebrafish Spleen Cells during Immune Response. 2016;196 2:715-25. doi:10.4049/jimmunol.1500847.
30. Melamed ZE, López-Erauskin J, Baughn MW, Zhang O, Drenner K, Sun Y, et al. Premature polyadenylation-mediated loss of stathmin-2 is a hallmark of TDP-43-dependent neurodegeneration. Nature Neuroscience. 2019;22 2:180-90. doi:10.1038/s41593-018-0293-z.
31. Rund D, Dowling C, Najjar K, Rachmilewitz EA, Kazazian HH and Oppenheim A. Two mutations in the beta-globin polyadenylylation signal reveal extended transcripts and new RNA polyadenylylation sites. 1992;89 10:4324-8. doi:10.1073/pnas.89.10.4324.
32. Shien J-H, Su Y-D and Wu H-Y. Regulation of coronaviral poly(A) tail length during infection is not coronavirus species- or host cell-specific. Virus Genes. 2014;49 3:383-92. doi:10.1007/s11262-014-1103-7.
33. Salles FJ, Richards WG and Strickland S. Assaying the polyadenylation state of mRNAs. Methods. 1999;17 1:38-45. doi:10.1006/meth.1998.0705.
34. Subtelny AO, Eichhorn SW, Chen GR, Sive H and Bartel DP. Poly(A)-tail profiling reveals an embryonic switch in translational control. Nature. 2014;508 7494:66-71. doi:10.1038/nature13007.

35. Garalde DR, Snell EA, Jachimowicz D, Sipos B, Lloyd JH, Bruce M, et al. Highly parallel direct RNA sequencing on an array of nanopores. Nature Methods. 2018;15 3:201-6. doi:10.1038/nmeth.4577.
36. Wan YK, Hendra C, Pratanwanich PN and Göke J. Beyond sequencing: machine learning algorithms extract biology hidden in Nanopore signal data. Trends in Genetics. 2022;38 3:246-57. doi:10.1016/j.tig.2021.09.001.
37. Brouze A, Krawczyk PS, Dziembowski A and Mroczek S. Measuring the tail: Methods for poly(A) tail profiling. WIREs RNA. 2023;14 1 doi:10.1002/wrna.1737.
38. Rand AC, Jain M, Eizenga JM, Musselman-Brown A, Olsen HE, Akeson M, et al. Mapping DNA methylation with high-throughput nanopore sequencing. Nature Methods. 2017;14 4:411-3. doi:10.1038/nmeth.4189.
39. Silverman JD, Bloom RJ, Jiang S, Durand HK, Dallow E, Mukherjee S, et al. Measuring and mitigating PCR bias in microbiota datasets. PLOS Computational Biology. 2021;17 7:e1009113. doi:10.1371/journal.pcbi.1009113.
40. Kim D, Lee J-Y, Yang J-S, Kim JW, Kim VN and Chang H. The Architecture of SARS-CoV-2 Transcriptome. Cell. 2020;181 4:914-21.e10. doi:10.1016/j.cell.2020.04.011.
41. de Jong LC, Cree S, Lattimore V, Wiggins GAR, Spurdle AB, kConFab I, et al. Nanopore sequencing of full-length BRCA1 mRNA transcripts reveals co-occurrence of known exon skipping events. Breast Cancer Res. 2017;19 1:127. doi:10.1186/s13058-017-0919-1.
42. Krause M, Niazi AM, Labun K, Torres Cleuren YN, Muller FS and Valen E. tailfindr: alignment-free poly(A) length measurement for Oxford Nanopore RNA and DNA sequencing. RNA. 2019;25 10:1229-41. doi:10.1261/rna.071332.119.
43. Leger A, Amaral PP, Pandolfini L, Capitanchik C, Capraro F, Miano V, et al. RNA modifications detection by comparative Nanopore direct RNA sequencing. Nature Communications. 2021;12 1 doi:10.1038/s41467-021-27393-3.
44. Chang JJ, Rawlinson D, Pitt ME, Taiaroa G, Gleeson J, Zhou C, et al. Transcriptional and epi-transcriptional dynamics of SARS-CoV-2 during cellular infection. Cell Rep. 2021;35 6:109108. doi:10.1016/j.celrep.2021.109108.
45. Aw JGA, Lim SW, Wang JX, Lambert FRP, Tan WT, Shen Y, et al. Determination of isoform-specific RNA structure with nanopore long reads. Nature Biotechnology. 2021;39 3:336-46. doi:10.1038/s41587-020-0712-z.
46. Simpson JT, Workman RE, Zuzarte PC, David M, Dursi LJ and Timp W. Detecting DNA cytosine methylation using nanopore sequencing. Nature Methods. 2017;14 4:407-10. doi:10.1038/nmeth.4184.
47. Oxford Nanopore Technologies. Dorado (PolyACalculator).
48. Teng H, Cao MD, Hall MB, Duarte T, Wang S and Coin LJM. Chiron: translating nanopore raw signal directly into nucleotide sequence using deep learning. GigaScience. 2018;7 5 doi:10.1093/gigascience/giy037.
49. Hardwick SA, Chen WY, Wong T, Deveson IW, Blackburn J, Andersen SB, et al. Spliced synthetic genes as internal controls in RNA sequencing experiments. Nature Methods. 2016;13 9:792-8. doi:10.1038/nmeth.3958.
50. Chang JJ-Y, Gleeson J, Rawlinson D, De Paoli-Iseppi R, Zhou C, Mordant FL, et al. Long-Read RNA Sequencing Identifies Polyadenylation Elongation and Differential Transcript Usage of Host Transcripts During SARS-CoV-2 In Vitro Infection. Front Immunol. 2022;13 doi:10.3389/fimmu.2022.832223.
51. He J, Ganesamoorthy D, Chang JJ-Y, Zhang J, Trevor SL, Gibbons KS, et al. Utilizing Nanopore direct RNA sequencing of blood from patients with sepsis for discovery of co- and post-transcriptional disease biomarkers. BMC Infectious Diseases. 2025;25 1 doi:10.1186/s12879-025-11078-z.

52. Jia J, Lu W, Liu B, Fang H, Yu Y, Mo W, et al. An atlas of plant full-length RNA reveals tissue-specific and monocots-dicots conserved regulation of poly(A) tail length. Nat Plants. 2022;8 9:1118-26. doi:10.1038/s41477-022-01224-9.
53. Payne A, Holmes N, Rakyan V and Loose M. BulkVis: a graphical viewer for Oxford nanoporebulk FAST5 files. Bioinformatics. 2019;35 13:2193-8. doi:10.1093/bioinformatics/bty841.
54. Chang JJ, Yang X, Teng H, Reames B, Corbin V, Coin LJM. Supporting data for "Using synthetic RNA to benchmark poly(A) length inference from direct RNA sequencing." GigaScience Database. 2025. https://doi.org/10.5524/102736.

Figure 1

Click here to access/download;Figure;Figure1.tiff

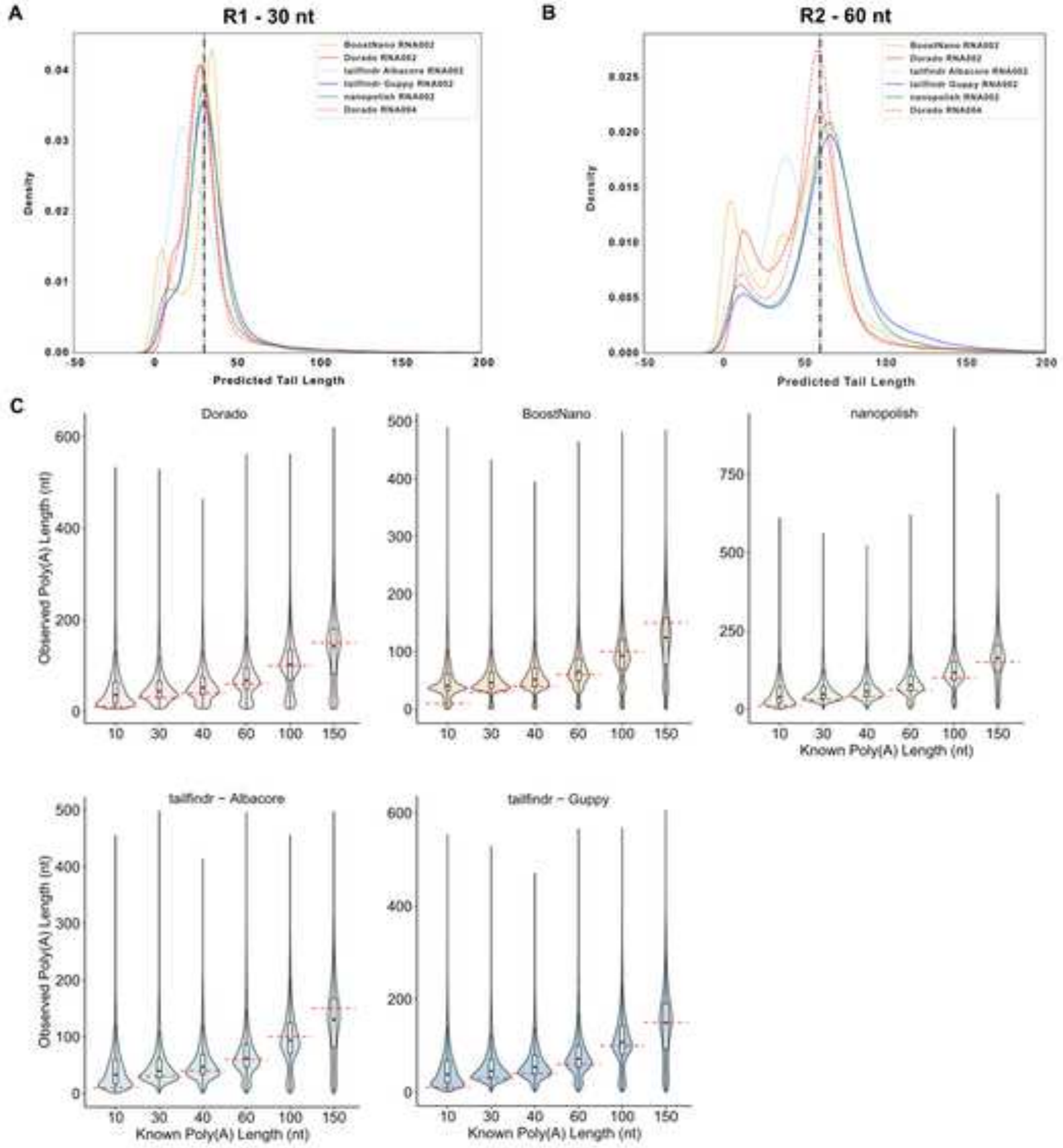

Figure 2

Click here to access/download;Figure;Figure 2.tiff

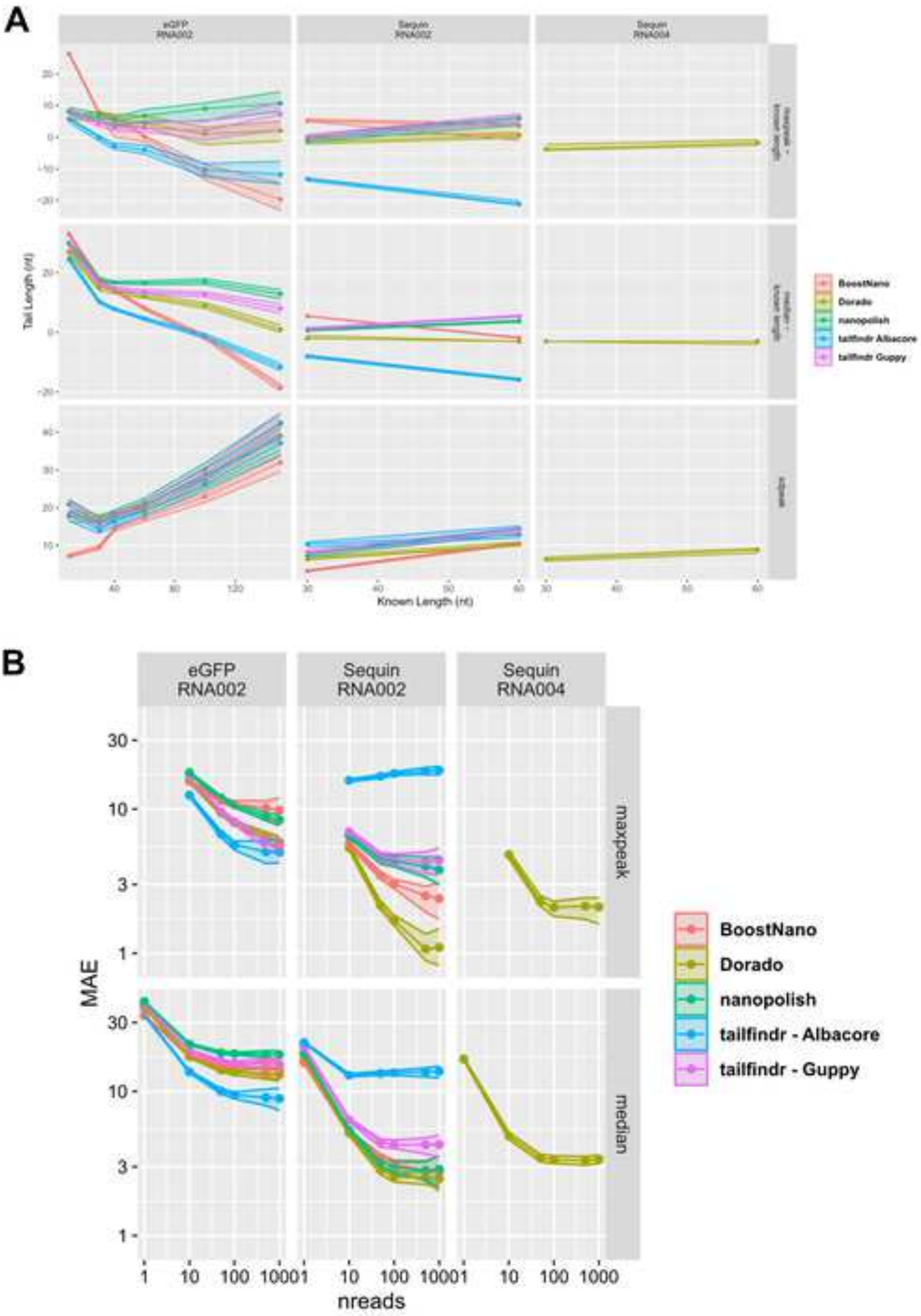

Figure 3

Click here to access/download;Figure;Figure 3.tiff

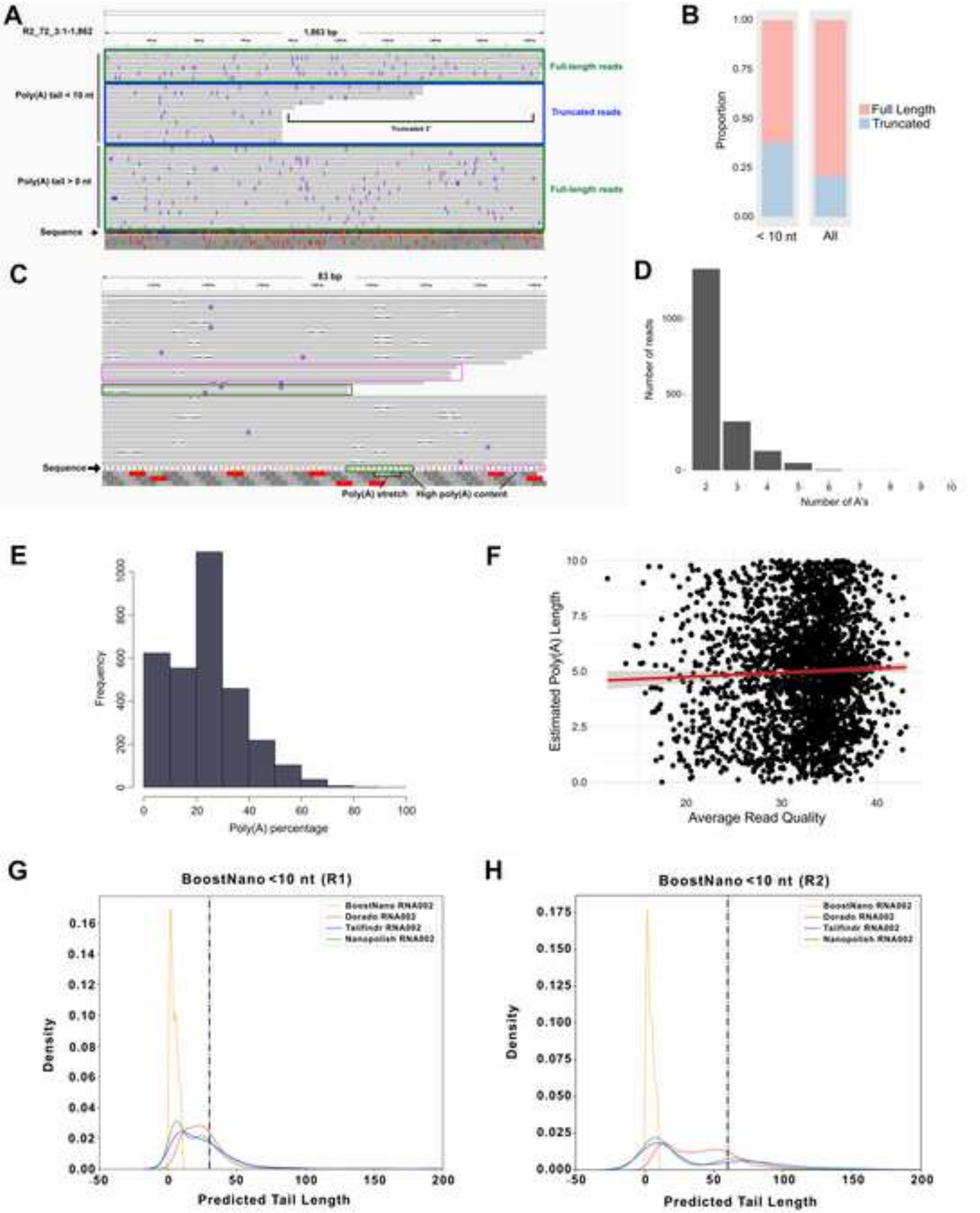

Figure 4

Click here to access/download;Figure;Figure 4.tiff

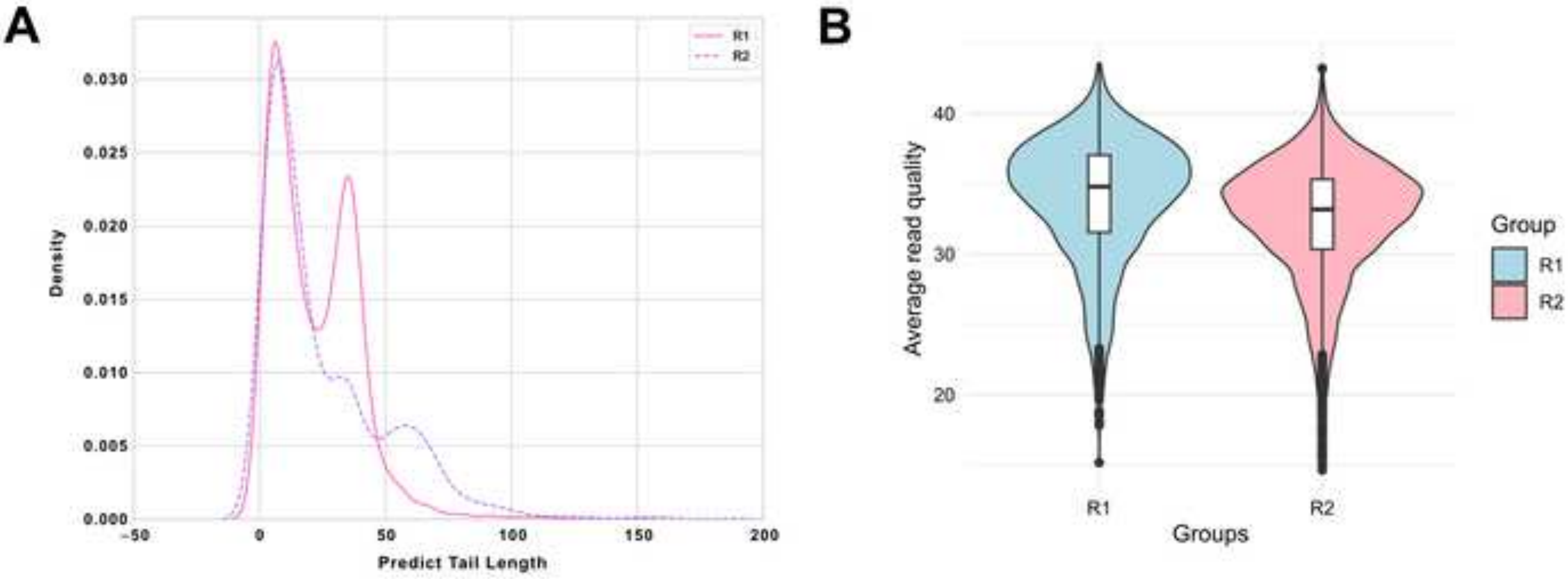

Click here to access/download
Supplementary Material
Supplementary_boostnano_10_06_2025_FINAL.docx

Supplementary Data S1

Click here to access/download
Supplementary Material
Supplementary Data S1.xlsx

Supplementary Data S2

Click here to access/download

Supplementary Material

Supplementary Data S2.xlsx

Click here to access/download
Supplementary Material
Supplementary Data S3.xlsx

Click here to access/download
Supplementary Material
Supplementary Data S4.csv

Response to Reviewers
Click here to access/download;Personal Cover;Response to reviewers round 2 FINAL.docx

Response to reviewers round 2

Dear Editor,

We sincerely appreciate the time and dedication of you and the reviewers to have spent on reviewing this manuscript.

Please kindly find our responses to reviewer #1's comments in blue below.

Reviewer reports:

Reviewer #1: In the revised version the authors present a significantly improved benchmark of tools to estimate polyA tail lengths in Nanopore direct RNA-sequencing data. While they include additional data, the full set is still lacking. Most important would be to include more on RNA004, as most readers will be mainly interested in this.
Some of the newly integrated analyses are not properly explained, as outlined below.
The authors now included a RNA004 data set containing the sequins with two different polyA tail lengths (30 and 60). For a full benchmark that is useful for the field, it would be however required to generate data with a greater dynamic range. On the one hand, the authors show some issues in detection of short polyA tails, on the other hand longer polyA tails are of biological significance.

We understand the importance of expanding the RNA004 dataset and agree with the reviewer that doing so would indeed allow an enhanced understanding of the poly(A) estimation by the different tools. For generating our RNA002 and RNA004 datasets, we have used Sequins which were available as a product. Unfortunately, we are unable to generate synthetic RNA with longer poly(A) tails as we do not possess the capability to do this in our own laboratory. Thus, we instead, expanded the dataset in the RNA002 data (10nt – 150 nt poly(A) tails), using publicly available datasets, as per the reviewer's original recommendations. Despite the shorter tails on the RNA004 datasets, we believe that the data is still useful for understanding the polyadenylome and particularly useful for mitochondrial, viral and plant RNA poly(A) tails, which are commonly within the ranges of the Sequin poly(A) tails. We have added a sentence in the limitations section to clarify this. ***"While the range for the RNA004 data may be shorter than biologically relevant in mammalian non-mitochondrial RNA, the range fits the expected lengths for mitochondrial, viral and plant RNA poly(A) tails.*** [PMID: 35982302, PMID: 33961822, PMID: 32330414, PMID: 40355874] (lines 387-389)

Is the new Figure 1C, first panel for GFP transcripts sequenced on RNA002 and analyzed with Dorado? How does such an analysis look for RNA004?

Yes, Figure 1C shows the eGFP transcripts sequenced on RNA002 and analysed with Dorado, which were publicly available. We have clarified this in the Figure 1C legends. Regrettably, we are unable to generate the same data for RNA004 as we lack capacity in our laboratory to generate synthetic IVT RNA.

Introduction: "This raw data is then converted into sequence data using a custom deep learning model, such as Dorado [46] or Chiron [47]". This is what a basecaller does. It is still misleading to mention Chiron as a single tool here but not mentioning Guppy or Rodan basecallers. I understand that it is used in BoostNano, but it should be mentioned only in this context or the other basecallers should be included in the list as well.

We have modified this statement and removed the basecaller examples (Dorado and Chiron):

"This raw data is then converted into sequence data using a custom deep learning model, via the use of basecallers." (lines 91-92)

The new Figures 3B, D, E need to be explained better. Why are the authors only focusing only on reads, where BoostNano estimates a polyA tail length < 10 nts? It would be sufficient to determine the number of As/ percent A at the read ends of truncated reads. Can the authors provide some whole transcriptome data analysis? This would have a much higher impact for the readers.

The rationale for including Figure 3, is for understanding the smaller peaks (< 10nt) as seen by the distribution plots in all datasets and all poly(A) estimation methods, as this was an unexpected result. As we wanted to isolate the reads < 10 nt, we had to choose one method to base our threshold, and we chose *BoostNano* as it showed the most prominent first peak. As a benchmarking manuscript with a focus on synthetic RNA to use ground truth datasets, we believe that adding results using whole transcriptomes is out of the scope of this manuscript. While this may be beneficial for the readers, investigating the polyadenylome in an organism will likely require the amount of work to synthesize a separate publication, and may not aid deeply in terms of comparing the variation between the tools. We have recently

published some work regarding the poly(A) distributions using RNA002 called with Dorado in human blood mRNA (PMID: 40355874) and have recognized that short poly(A) tails are also shown through these datasets (~<10 nt), highlighting that shorter poly(A) tails are also found *in vivo*. We wish to note that many of the datasets available using direct RNA-sequencing are derived from RNA002 and it is still important to understand the performance differences suitable for RNA002 data. We have added the following future directions to the limitations section: "***Overall, future work would benefit from expanding the range of poly(A) lengths to better mimic the distribution in real samples via synthetic and whole transcriptome data, gaining an enhanced understanding of length-specific biases in each tool and including RNA from diverse preparation methods."*** (lines 389-392).

The authors still argue that the RTA adapter may be degraded and now cite the work by Davis et al to support this. However, in this work they show that especially T-mers are stable over at least 70 freeze-and-thaw cycles. Based on the kit size of six reactions, the RTA should undergo maximal 5 freeze-and-thaw cycles and should be considered stable. Thus, I recommend to remove this statement.

We have removed this statement. In its place, we investigated the alternative idea of split-reads during the signal detection stage: "***We hypothesized that poly(A) tails shorter than 10 nt may result from signal glitches during the signal detection, where one read may be written as multiple reads. Using Bulkvis, we discovered that among a total of 60,146 reads from all 7 RNA002 samples, 270 pairs of split reads were found (0.898%), with 26 pairs including one read in the list of reads with < 10 nt poly(A) tails (Data S4). This suggests that while read splits may partially explain the shorter poly(A) tails, other unexplained mechanisms are at play."*** (lines 269-274)

I don't understand Figures 3G and H. The authors select reads, for which BoostNano estimates very short polyA tails. For these reads, the other tools estimate polyA tail lengths which are closer to the expected length. Strictly spoken, this shows mainly that BoostNano should not be considered for estimating short polyA tail lengths.

The distribution plots show that the other tools (especially Nanopolish and Tailfindr) also exhibit a peak at a similar point as BoostNano if we take the maximum peak of density. However, we agree with the reviewer that BoostNano does indeed perform worst in terms of short poly(A) tails. We have added/amended to the following: "*Our analysis revealed that while all four poly(A) estimation methods consistently identified shorter poly(A) tails, BoostNano exhibited a narrower peak for these shorter tails, whereas Dorado tended to estimate longer poly(A) tails that were closer*

to the known values (Figures 3g-h). ***Given that tailfindr and nanopolish also exhibited a peak at similar points in the density distributions as BoostNano, Dorado likely overestimates very short tails. Overall, the narrow peak of BoostNano indicates that BoostNano may not be suitable for estimating shorter poly(A) tails compared with the other tools."*** (lines 239-245).
