# Supplementary material for: Using synthetic RNA to benchmark poly(A) length inference from direct RNA sequencing
Source: Gigascience. 2025 Sep 3;14:giaf098. doi: 10.1093/gigascience/giaf098 (PMC12406214; doi:10.1093/gigascience/giaf098)
Supplement: giaf098_Authors_Response_To_Reviewer_Comments_original_submission [file giaf098_authors_response_to_reviewer_comments_original_submission.pdf]

Author's Response To Reviewer Comments

Dear Editor,

We sincerely appreciate the time and dedication of you and the reviewers to have spent on reviewing this manuscript.

Please kindly find our responses to reviewers' comments in blue below and please note that all grammar correction suggestions have been appropriately incorporated into the new version of the manuscript.

Please note that we have updated the Dorado dataset to include data from the latest version of Dorado v0.9.0.

External email: Please exercise caution

GIGA-D-24-00432
Using synthetic RNA to benchmark poly(A) length inference from direct RNA sequencing.
Jessie J-Y Chang; Xuan Yang; Haotien Teng; Benjamin Reames; Vincent Corbin; Lachlan Coin
GigaScience

Dear Dr Coin,

Happy New Year!

Your manuscript "Using synthetic RNA to benchmark poly(A) length inference from direct RNA sequencing." (GIGA-D-24-00432) has been assessed by our reviewers. Although it is of interest, we are unable to consider it for publication in its current form. The reviewers have raised a number of points which we believe would improve the manuscript and may allow a revised version to be published in GigaScience.

Their reports, together with any other comments, are below. Please also take a moment to check our website at https://www.editorialmanager.com/giga/ for any additional comments that were saved as attachments.

In addition, please register any new software application in the bio.tools and SciCrunch.org databases to receive RRID (Research Resource Identification Initiative ID) and biotoolsID identifiers, and include these in your manuscript. Computational workflows should be registered in workflowhub.eu and the DOIs cited in the relevant places in the manuscript. These will facilitate tracking, reproducibility and re-use of your tool.

If you are able to fully address these points, we would encourage you to submit a revised manuscript to GigaScience. Once you have made the necessary corrections, please submit online at:

We have added boostNano to bio.tools and SciCrunch.org databases and referred the relevant ID's in the manuscript as the Editor has kindly suggested. We have additionally noted links as appropriate to refer to data and scripts on Figshare, which will be replaced with DOIs after acceptance.

https://www.editorialmanager.com/giga/

If you have forgotten your username or password please use the "Send Login Details" link to get your login information. For security reasons, your password will be reset.

Please include a point-by-point within the 'Response to Reviewers' box in the submission system. Please ensure you describe additional experiments that were carried out and include a detailed rebuttal of any criticisms or requested revisions that you disagreed with. Please also ensure that your revised manuscript conforms to the journal style, which can be found in the Instructions for Authors on the journal homepage. If the data and code has been modified in the revision process please be sure to update the public versions

of this too.

The due date for submitting the revised version of your article is 07 Apr 2025.

I look forward to receiving your revised manuscript soon.

Best wishes,

Hongfang Zhang
GigaScience
www.gigasciencejournal.com

Reviewer reports:
Reviewer #1: In this manuscript, the authors present a benchmark to assess the performance of different tools designed for estimation of polyA tail length from Nanopore direct RNA-sequencing data. These tools include tailfindr, nanopolish, Dorado and Boost Nano.
Benchmarks on tools and algorithms to analyze Nanopore data, both third party tools and official ONT releases, are of utmost importance for the field. The use of synthetic constructs with known ground truth is recommended as well. Consequently, this study has the potential to provide a significant contribution to the field.

In the current form, I can however not recommend it for publication in GigaScience. My major concerns are:
a) Use of only RNA002 data. This chemistry is outdated and thus the Benchmark is only relevant for old, possibly already published data. A comprehensive Benchmark should also include RNA004 and available tools there (at least Dorado).

We have generated new RNA004 data of sequins for this revision. We have analysed this data using Dorado v0.9.0 in our manuscript. We attempted to use other tools with RNA004 data, but this was not possible due to incompatibility. Nevertheless, we are able to compare the estimates from RNA004 data to RNA002 data.

b) The current data set only contains two polyA tail length, which are relatively short and do not cover longer polyA tails that are common e.g. in mammalian cells. A proper Benchmark should show the performance of the analyzed tools over a range of polyA tail lengths.

We have added synthetic IVT eGFP RNA data that span over a range of polyA tail lengths derived from the developers of tailfindr. The data include RNA with poly(A) tails with 10, 30, 40, 60, 100, 150 nt.

Minor comments:
Abstract: "All four tools generate mean tail-length estimates which lie within 13% of the correct value." The value of 13% is given in the Abstract from the submission system, wherease the abstract in the Main text says 12%. Which value is correct?

We have now removed this statement from the Abstract and the Main Text as we no longer focus on mean tail length.

2) Background, first paragraph: the role of the polyA tail in RNA circularization, which is required for efficient translation of cellular mRNAs is not mentioned. Reference is missing for "is increasingly recognised as a dynamic process which influences timing and degree of protein production."

This has been moved to the second paragraph of the background - the role of the poly(A) tail in RNA circularization and the appropriate references were added (lines 65-68).

3) Background, second paragraph: Chiron seems to be a relatively old basecaller (no models for new chemistries). It should be mentioned here that it is required for BoostNano.

Chiron is not required for BoostNano, as basecalling is not necessary for using the tool. Instead, the model

structure of Chiron is used as a basis for BoostNano. We have clarified this in Table 1 – “Convolutional Neural Network (CNN)-Recurrent Neural Network (RNN)-Connection-ist Temporal Classification (CTC) architecture from Chiron basecaller used to find boundaries of poly(A) in raw signal, basecalling not required”

4) Mis-priming of internal polyA sites may an important confounding (and currently overlooked) source of errors in Nanopore sequencing. This should be quantified properly and analyzed in more detail (length of these stretches, influence of other nucleotides within the A-rich stretch, etc.). Should be done as well on whole transcriptome data with more possible mispriming sites.

We have analyzed the whole Sequin transcriptome dataset to understand and quantify priming due to internal polyA sites or fragmentation via visualising the 10 nt downstream of the 3’ end of the mapped transcript in the reference transcriptome, as the poly(T) stretch of the RTA is 10 nt long. Furthermore, we analyzed the poly(A) content by calculating the percentage of adenines in that 10 nt stretch. This information was added as Figures 3B, D-E.

5) Why do the authors think that the poly(T) stretch of the RTA might be truncated? This is composed of DNA oligos, which should be quite stable

Yes, we agree that DNA oligos are much more stable than RNA oligos and therefore should be quite stable. However, that does not mean that truncation and fragmentation cannot happen with DNA oligos. Mechanical forces, freeze-thaw and enzymatic hydrolysis can contribute to the degradation of DNA oligos. Please see the following publication discussing the effect of freeze-thaw to DNA oligos with 10T’s (Davis, O'Brie & Bentzley, 2000 - Analysis of the Degradation of Oligonucleotide Strands During the Freezing/Thawing Processes Using MALDI-MS DOI: 10.1021/ac000225s).

6) What are the parameters for filtering used by Dorado and BoostNano? Can the authors explain, why the filtered reads differ?

We have only utilized default parameters for both Dorado and BoostNano, hence no specific filtering parameter was utilized. Dorado bases its poly(A) estimation on an analysis of the signal, searching for a low variability region near its identified anchor point (typically the cDNA primer or RNA adapter) and an estimate of the translocation speed based on the move table of the read. If the low variability region or anchor point cannot be located, the reads could be filtered out. BoostNano assigns poly(A) tails to all reads.

7) Dorado seems to systematically underestimate polyA tail length. Is this true also for data generated with RNA004 chemistry and longer polyA tails?

Dorado’s poly(A) tail length estimates tend to be the most accurate - please see updated Figures 1a and 1b. When comparing RNA002 and RNA004, Dorado seems to underestimate poly(A) tails more in RNA004 data than RNA002 data.

Reviewer #2: This manuscript addresses a relevant and timely question: benchmarking poly(A) tail-length estimation tools (BoostNano, tailfindr, nanopolish, and Dorado) using synthetic RNA standards (Sequins) with known tail lengths. Poly(A) tail-length estimation is increasingly important for understanding mRNA stability, processing, and regulation at the single-molecule level. As direct RNA sequencing expands in use, reliable methods to measure poly(A) tail lengths are needed. The study's design—leveraging Sequins as a "gold standard" to benchmark tools—is strong and fills an area is need in current literature. The analysis is thorough in its basic comparisons, and the results are likely to be useful to researchers who need to choose suitable software for poly(A) tail analysis. However, the manuscript would benefit from deeper contextualization, more rigorous statistical methodology, and clearer reporting of computational details. Ensuring reproducibility and providing clearer guidance on interpreting the results in real biological contexts would strengthen the manuscript. The suggestions below are aimed at making the study more valuable to the community.
For this reason, my recommendation is Revisions ARE Needed

Introduction

Abstract: ★★★★☆ (4/5) Actually in place of the introduction, it has it strengths:
The introduction adequately outlines why polyadenylation is biologically important and why direct RNA sequencing provides a unique opportunity for poly(A) tail-length estimation.
It justifies the use of Sequins as synthetic standards, which is a robust approach to derive ground-truth tail lengths.

Areas for Improvement: The introduction could better connect poly(A) tail-length estimation to downstream applications. For instance, mention how accurate tail-length estimation could improve understanding of mRNA decay rates, translation efficiency, or isoform-specific regulation.

We have included wording to connect poly(A) estimation to downstream applications in both the abstract and the introduction (lines 32-34, 65-76).

Adding references that contextualize poly(A) tail dynamics in broader biological phenomena would help readers understand the significance.
For example, it is almost a necessity to cite work such as "Roles of mRNA poly(A) tails in regulation of eukaryotic gene expression" by Lori A. Passmore & Jeff Coller (2022, Nature Reviews Molecular Cell Biology) which provides a comprehensive analysis of poly(A) tail dynamics and their impact on mRNA decay, stability, and translation regulation. P & C (2022) also expands on these principles by discussing the mechanistic underpinnings of poly(A)-mediated decay and translation regulation, making it a broader and more recent contribution to polyadenylation biology, which the authors should consider.

Thank you for the suggestion – we have now referenced this work and other relevant publications in our manuscript (page 2).

Grammar of the abstract:
Error: "There are currently several tools available for poly(A) tail-length estimation, including well-established tools such as tailfindr and nanopolish, as well as two more recent deep learning models: Dorado and BoostNano."
⎱ Suggestion: "Several tools are currently available for poly(A) tail-length estimation, including well-established methods like tailfindr and nanopolish, as well as two more recent deep learning models: Dorado and BoostNano."

Error: "which lie within 12% of the correct value."
⎱ Suggestion: "that lie within 12% of the correct value."

Clarify the library preparation steps to avoid confusion about the "direct" nature of RNA sequencing. The text currently implies that no reverse transcription is required, but then references an ONT Reverse Transcription Adapter. Distinguish between a full-length cDNA synthesis step (not required) and the use of a poly(T)-containing adapter for sequencing library preparation.

Thank you for the suggestion. We have added amended the relevant paragraph in the Background section:

In contrast, Oxford Nanopore Technologies (ONT) direct RNA-sequencing is a simple approach for single-molecule RNA-sequencing which does not require reverse transcription (other than for RNA stabilization and improving sequencing output) or polymerase chain reaction (PCR) amplification, thus avoiding amplification bias and retaining the original base and base-modification information [35-39]. It is worth noting that the full-length cDNA synthesis step, while not required, is recommended and the library preparation method utilizes a poly(T)-containing adapter for sequencing. (lines 82-88)

Methods

Methods: ★★★★☆ (4/5)
The methods section has its strengths; the data sources and preparation (Sequins spiked into host RNA)

are clearly described. Versions of tools are provided, enhancing reproducibility.

Areas for Improvement are statistical analysis, comparisons and tests, hardware and computation details, and understanding of run time differences.

All aspects of areas of improvement suggested by the reviewer have been addressed and improved in the newest version of the manuscript. We have modified the analysis to include calculation of mean absolute error (MAE), as well as averaging multiple reads using medians and position at which the probability density function achieves is maximum. We now include confidence intervals on all estimates which have been calculated via bootstrap resampling. Also, we added more details regarding computational details – including RAM and processors (Table 2).

Currently, the study models distributions as normal and uses mean and SD, but no normality tests or justification for these choices are presented. Consider performing normality tests or using nonparametric measures. Additionally, providing confidence intervals or other robust statistics (median, interquartile ranges) would clarify variability.
For the comparisons and tests, the authors should explain why you chose root mean square error (RMSE) minimization and other metrics.
Could alternative tests, like Wilcoxon signed-rank tests or paired t-tests (Wilcocoxon: this non-parametric test is suitable for paired comparisons when the assumption of normality is not met. -useful to compare the predicted tail lengths from each tool against the expected lengths, especially if the data distribution is skewed.), be used to compare the distribution of tail-length estimates more rigorously?
Paired t-Test, because this test could be applied if the normality assumption holds, providing a straightforward way to assess whether the mean difference between predicted and expected values is statistically significant. (If so, justification should be provided for why or why not)

Our data is not normally distributed, it has a multi-modal distribution. We only use the term “normal” in the shape of the highest peak, so t-tests will not be feasible for comparing the predicted tail lengths vs expected tail lengths. We have now removed the part where we model the second peak as normal distribution, and RMSEs to alleviate any confusions. We have added confidence intervals, SDs, medians and maximum peak of the density distribution instead (Data S1, Figures 2a-b). We use bootstrap resampling to evaluate significance of one tool having smaller mean absolute error than another, thus avoiding use parametric approaches for significance testing.

There are some additional metrics to explore:
---Median Absolute Deviation (MAD): Consider adding MAD as it is robust to outliers and could complement RMSE to provide a better understanding of central tendencies and variability.
---Mean Absolute Error (MAE): MAE is another alternative that simplifies the interpretation by focusing solely on the magnitude of errors without squaring them, potentially offering more intuitive insights for readers.

We explored the MAE of each tool across windows of various sizes and explored the median or maximum peak values. The results have been added as Figures 2b and S7, as we believe this metric would be the most beneficial to the readers.

The authors should address testing for normality, explicitly stating whether normality tests were conducted on the data (e.g., Shapiro-Wilk or Kolmogorov-Smirnov tests). If normality is confirmed, justify the use of parametric tests like RMSE or t-tests. If not, justify why non-parametric tests (e.g., Wilcoxon) were not employed or discuss plans to include them in future studies.

We are no longer implementing the normal distribution strategy and therefore have not carried out the suggested normality tests.

Explain the choice of statistical methods over time by discussing how the choice of statistical tests aligns with the study's goals. For example, emphasize whether the focus was on understanding overall error distribution, tool consistency, or accuracy in predicting specific tail lengths.

We have implemented statistical methods to understand all of the above – overall error distribution, tool consistency and accuracy in predicting specific tail lengths. We believe that the maximum peak of density distribution and the MAE are most appropriate metrics that align with the study’s goals.

The authors could use visual representations of error complementing the statistical tests with visual aids such as boxplots, violin plots, or Bland-Altman plots to illustrate the error distributions and discrepancies between predicted and actual tail lengths across tools.
(maybe supplementary)

We have added plots showing the Mean absolute error of all tools (all, R1 and R2) in Figures 2b and S7.

The authors should provide hardware and computational details like providing explicit details on the computational environment—CPU/GPU models, RAM, OS—for each tool's run. While the Git-hub read me suggests how to run the system, it lacks any details about system requirements.
Readers need this to understand runtime differences and attempt to replicate performance measurements.

We have updated the Github README to include details about the system requirements (e.g. Python version, OS). The suggested hardware and computational details have been added in Table 2.

The authors should consider tool parameterization and indicate if any specific parameters (beyond defaults) were used in tailfindr, nanopolish, Dorado, or BoostNano runs. If no changes were made from defaults, state this explicitly.

We have added a table in the Methods to add the parameters used for analysis for each of the tools we have discussed in our manuscript (Table 3).

Results

The result's strengths are that they are presented clearly, showing density distributions and discussing short-tail anomalies. The identification of Dorado as a preferred tool due to speed, integration, and conservative filtering is well-supported by the data. The study acknowledges that all tools achieve broadly similar accuracy, differing mainly in runtime and filtering criteria, which is a practical insight for users.

The results have areas for improvement:
Regrading the short-tail reads explanation, the authors attribute short (<10 nt) poly(A) tails to truncated transcripts or mis-priming. For this reason, it is suggested that the authors strengthen this discussion with additional evidence or reasoning. For instance, is there a correlation between read quality and short-tail length estimates? Do truncated reads consistently align to internal A-rich stretches?

We generated correlation plots between expected poly(A) length and average read quality per read and used Spearman’s correlation analysis to investigate the relationship (Figure S8). There is indeed a weak positive correlation between poly(A) length and read quality in some datasets (e.g. dorado R2, BoostNano R1, Boostnano R2 and tailfindr R1 in Sequins). However, we have identified that the truncated reads generally have an average read quality score > 20 (Figure 3f), showing that poor read quality score is not the main cause of these truncated poly(A) tails. We have also included results to show that most truncated reads do not align to internal poly(A) stretches nor stretches with high poly(A) content (Figures 3d-e).

Multiple peaks in distributions: Some density plots (Figure 1) show multiple peaks or shoulder peaks.

Discuss potential reasons for these patterns. Are they related to tool-specific biases, read quality, or adapter/poly(T) truncation?

All tools in our study and all versions of these tools show either a multi-modal distribution or shoulder peaks (Figures 1a-c). This seems to indicate that this is not a tool-specific effect, except for BoostNano, which showed a clear bimodal distribution in R1 sequins and trimodal distribution in R2 sequins and eGFP datasets. We note that multiple peaks were more prominent in longer known poly(A) length RNA, and this

was expected given that there is more chance for longer tails to become fragmented than shorter tails. Therefore, as the early peak presents with most tools, we believe this is a feature of the sample, instead of the tool. We discuss this in more detail on pages 5 and 12. We modelled the poly(A) length vs quality score and found a weak, positive correlation, suggesting that there is some correlation with read quality (Figure S8), however, this is unlikely to be the major reason for these patterns.

Application Context: The results focus on method performance, but it would help readers to understand how these differences might influence downstream tasks. For example, if a method overestimates poly(A) length slightly, how could this affect conclusions about RNA stability or differential tail-length analysis between experimental conditions?

We have added a paragraph regarding this in the first paragraph of the discussion section (lines 330-337). “The implications of poly(A) estimation outputs can lead to different varied interpretations. If a method overestimates poly(A) tails, the researcher may overestimate other associated functions such as RNA stability, since longer poly(A) tails are commonly associated with greater stability. This is particularly detrimental when exploring differential polyadenylation, as even small changes in poly(A) tails may influence statistical tests. Thus, as a general, we recommend the following; should the researcher have specific expected tail lengths for their study, they should choose the most appropriate tool based on the results of this study or similar, with the use of maxpeaks to average over read sets (N>100). In more complex transcriptomes with wider variety of tail lengths, tailfindr or Dorado should be utilized.”

Figures and tables:
Figure 1:

Clear density plots, but consider adding vertical lines at expected tail lengths (30 nt and 60 nt) to guide interpretation. Splitting the figure into separate panels for R1 and R2 or using insets might clarify multiple peaks.

We have added dotted vertical lines at expected tail lengths and split the figure into two separate panels for R1 and R2 as per the reviewer’s recommendations (Figures 1a-b).

Figure 2:
The IGV snapshots are informative. Enhance interpretability by adding annotations (arrows or boxes) highlighting truncated vs. full-length reads. Increase font sizes for readability.

Thank you for the suggestions. Boxes to highlight truncated vs full-length reads were added to Figure 3A and we have generally increased the font sizes for readability.

Figure 3:

Useful comparison of reads filtered by Dorado but retained by BoostNano.
Add a brief note or labeling to indicate expected tail lengths.
Discuss possible reasons for Dorado's conservative filtering here or in the main text.

We have added a note to indicate the expected tail lengths in the figure legends. Dorado’s conservative nature might be due to the tendency of the tool to base its estimation on searching for a low variability region near an anchor point, and if such regions are undefined, there could be an omission of the reads. This information has been added to the discussion section in the third paragraph.

Tables:
Provide definitions for abbreviations (nt, CPU, GPU) in captions. For Table 2, adding confidence intervals around the mean tail-length estimates would strengthen statistical rigor. For Table 3, specify hardware details as recommended above.

The abbreviations have been defined in the table captions. We have added confidence intervals for medians and maxpeaks in Supplementary Data S1. We have added computation configurations used in Table 2 for reproducibility.

Grammar Mistakes and errors in the results section:
Results Section:
Sentence: "The four methods display a similar pattern in the density distribution, with a prominent normal-like peak near the expected poly(A) length, but also with a over-representation of shorter poly(A) tails, ranging at approximately ~0-10 nt (Figure 1)."
Issue: "a over-representation"
⎱ Correction: "an over-representation"

Sentence: "We expected that these shorter peaks were derived from either fragmentation of the transcript, mis-priming of internal poly(A) stretches or degradation of the poly(A) tails."
Issue: tense mismatch ("expected" vs. "were derived").
⎱ Correction: "We expect" -- "were derived", loses context and tense conformity-- therefore the sentence should be adjusted-
"We hypothesize that these shorter peaks are derived from either fragmentation of the transcript, mis-priming of internal poly(A) stretches, or degradation of the poly(A) tails."

Sentence: "Interestingly, upon investigating these earlier peaks, we found that Dorado excludes reads which are retained in the analysis by BoostNano, despite them being classified as passed reads (Figure 3)."
Issue: Ambiguous pronoun "them." (them could incorrectly identify three possible targets in the sentence)
⎱ Correction: "Interestingly, upon investigating these earlier peaks, we found that Dorado excludes reads retained in the analysis by BoostNano, even though these reads are classified as passed reads (Figure 3)."

Sentence: "Therefore, Dorado appears to be a more conservative approach than BoostNano."
Issue: No grammar issues, but the statement could be more precise.
⎱ Suggested improvement: "Thus, Dorado demonstrates a more conservative approach compared to BoostNano."

Sentence: "In order to determine which normal distribution fit the peak best, we found the parameters (mean, SD) which minimize the root mean square error between the candidate normal distribution and the density distribution for an interval of 10 nt to the right of the mode."
Issue: Verb tense consistency ("fit").
⎱ Correction: "To determine which normal distribution fits the peak best, ..."

Sentence: "The peaks also lose their normal-like behavior for larger values."
⎱ Issue: Could use a more formal tone. Correction: "The peaks also deviate from their normal-like behavior at larger values."

Sentence: "Next, we compared the computational time required by each method to predict the tail-length of 4000 reads."
Issue: Hyphenation of "tail-length."
Correction: "Next, we compared the computational time required by each method to predict the tail length of 4,000 reads."

We have revised all hyphenated versions of ‘tail-length’ to ‘tail length’.

Sentence: "BoostNano also offers the option of using the Application Programming Interface (API) call instead of the direct method, which omits the file copy implemented in the direct approach, reducing the run time to 8 m 8 s."
Here, the sentence is extremely overwritten which causes a lack of clarity.
⎱ Correction: "BoostNano offers an alternative API-based method, which skips the file copy step of the direct approach, reducing the runtime to 8 minutes and 8 seconds."

Discussion

Discussion: ★★★☆☆ (3/5)
The discussion as its strengths as it correctly identifies that Dorado's advantages (speed, integration with basecalling) make it appealing as a default choice.
The authors acknowledge that all tools are within a similar accuracy range, suggesting the deciding factor may be speed or integration rather than raw performance differences.
HOWEVER- there are areas for improvement:

Further dissect the limitations of each tool. For example, BoostNano shows good SD but slightly off mean for R1; what does this mean for its use cases?
We have amended the approach for using metrics for determining accuracy – we have added medians, maxpeaks, SD, CI, MAE and raw differences instead of the mean. We have noted a length- and sample-dependency in our results, which we have discussed in the first paragraph of the discussion section. In the same paragraph we noted – "The implications of poly(A) estimation outputs can lead to different varied interpretations. If a method overestimates poly(A) tails, the researcher may overestimate other associated functions such as RNA stability, since longer poly(A) tails are commonly associated with greater stability. This is particularly detrimental when exploring differential polyadenylation, as even small changes in poly(A) tails may influence statistical tests. Thus, as a general, we recommend the following; should the researcher have specific expected tail lengths for their study, they should choose the most appropriate tool based on the results of this study or similar, with the use of maxpeaks to average over read sets (N>100). In more complex transcriptomes with wider variety of tail lengths, tailfindr or Dorado should be utilized."

Address the discrepancy between tailfindr, nanopolish, and Dorado in terms of how they define and detect poly(A) boundaries. Why does Dorado not evaluate start/end positions of poly(A) tails in event space, and how might this influence results?

The differences in determining poly(A) boundaries in each tool have now been described in the background section (lines 106-121). Apologies for the confusion, we meant that Dorado does not output start and end positions of the poly(A) tails, nor the signals, unlike the other tools. Dorado of course must calculate start and end positions for estimating poly(A) tails.
Include a brief discussion about how results might generalize to more complex transcriptomes. Real samples have varying GC content, fragment lengths, and potentially modified bases. A short commentary acknowledging these factors would show awareness that synthetic standards cannot capture the full complexity of natural RNA populations.

We added a brief discussion about the generalizability of our data in the paragraph regarding limitations of this study in the discussion section (page 13, lines 382-392).
For these reasons, it is suggested that the authors suggest future directions.
For instance, how could tool developers incorporate these findings to improve their methods? Could future benchmarking sets include a gradient of tail lengths to better understand length-specific biases?

A section of future directions for tool development and tool benchmarking has been added in the discussion section (lines 397-399).

Grammar Mistakes and errors in the discussion section:
Sentence: "BoostNano and tailfindr tools provided estimation of the starting and ending positions of the poly(A) tails in event space while this information was absent in Dorado outputs."
Issue: "provided estimation" should be "provide estimation" to align with present tense.
⎫ Correction: "BoostNano and tailfindr tools provide estimation of the starting and ending positions of the poly(A) tails in event space, while this information is absent in Dorado outputs."

Sentence: "On the R1 dataset, BoostNano showed a tighter distribution with the smallest SD, but its peak was the furthest from the correct value."
The issue here is that the test results are still speaking about general truths leading to verb tense inconsistency; "showed" should match other verbs in the section.
⎫ Correction: "On the R1 dataset, BoostNano shows a tighter distribution with the smallest SD, but its peak

is the furthest from the correct value."

Sentence: "tailfindr had the most accurate estimation but also the largest error interval."

The issue here is the verb tense mismatch; "had" should be consistent with present tense to show truth, not past truth.
⎱ Correction: "tailfindr has the most accurate estimation but also the largest error interval."

Sentence: "Furthermore, Boostnano is more lenient in keeping reads for poly(A) estimation than Dorado."

Issue: "Boostnano" capitalization error; it should be "BoostNano."
⎱ Correction: "Furthermore, BoostNano is more lenient in keeping reads for poly(A) estimation than Dorado."

Sentence: "Overall, our results suggest that the four tools investigated in this study - BoostNano, tailfindr, nanopolish and Dorado have similar performance with their accuracy varying from one dataset to the other, with a potential length bias."

Issue: Missing commas for clarity; replace "with their accuracy varying from one dataset to the other" for conciseness.
⎱ Correction: "Overall, our results suggest that the four tools investigated in this study—BoostNano, tailfindr, nanopolish, and Dorado—have similar performance, with accuracy varying across datasets and showing potential length bias."

Sentence: "Therefore, we expect Dorado to be implemented as the default method of poly(A) tail estimation in the near future, with the rapid estimation timeframe, comparable estimation lengths to other tools, conservative nature and the added benefit of ease of obtaining this information during basecalling."

There are several issues here including verbosity and lack of parallelism.
⎱ Correction: "Therefore, we expect Dorado to be implemented as the default method for poly(A) tail estimation, given its rapid estimation timeframe, comparable accuracy to other tools, conservative nature, and ease of integration with basecalling."

Sentence: "This work demonstrates the value of having access to synthetic RNA molecules with known poly(A) tail-lengths for validating the accuracy of poly(A) tail estimation algorithms."

Issue: The phrase "validating the accuracy of" could be simplified for readability.
⎱ Correction: "This work demonstrates the value of synthetic RNA molecules with known poly(A) tail lengths for validating poly(A) tail estimation algorithms."

Sentence: "As methods improve, we anticipate that these datasets will be valuable for assessing improvements in estimation of poly(A) tails."

Issue: "improvements in estimation of" is awkward.
⎱ Correction: "As methods improve, we anticipate that these datasets will be valuable for assessing advancements in poly(A) tail estimation."

References need to be added to accommodate the suggested material review, but existing references are good

We have added more relevant references to the manuscript to supplement our findings and the background/discussion.

NEEDS REVISION
Jesse Daniel Brown PD AASU

Note:

I previously reviewed this paper previously in Research Hub and you can read these comments via the Research Hub review page here: https://www.researchhub.com/paper/8634403/using-synthetic-rna-to-benchmark-polya-length-inference-from-direct-rna-sequencing/reviews#threadId=55398.

The original preprint linked to the Research Hub review is here: https://doi.org/10.1101/2024.10.25.620206

Reviewer #3: The manuscript presents interesting findings, but several points require clarification and further discussion to enhance transparency, statistical robustness, and reproducibility. Addressing the disdcrepancies in Figure 1, justifying the choice of statistical methods, and providing detailed computational configurations will significantly improve the manuscript. Based on the above stated, my recommendation it is by accepting for publication after a major revision.

Discrepancies in Figure 1 have been addressed in the first paragraph of section ("Performance evaluation between BoostNano, tailfindr, nanopolish and Dorado"). We utilized the maxpeak metric and bootstrap based p-values to understand the accuracy of each test and have discussed this in the manuscript (page 5, lines 158-171, page 10, lines 276-289). Detailed computational configurations have been listed in Table 2 as well as updated methods for enhancing reproducibility.

Regarding the criteria from GigaScience Journal for Technical Notes this technical note comply partially with technical improvement/utility, because while the study demonstrates that Dorado is the fastest and most conservative method, the novelty of technical improvement is somewhat limited because the tools themselves are not newly developed by the authors (exception of BoostNano). That said, the benchmarking study provides value for the research community by helping users choose the most appropriate tool for poly(A) tail-length estimation.

The reviewer declare that he does not have competing interests.

The abstract provides a concise summary of the study's aim, methodology, and key findings. However, it lacks sufficient emphasis on the broader implications of benchmarking poly(A) tail-length estimation for transcriptomics research. The abstract mentions the advantages of Dorado in terms of speed and accuracy but does not explicitly describe the limitations of other tools or potential use cases of these findings. Expanding the abstract to connect the results to downstream applications such as transcript stability analysis or isoform quantification could improve its utility.

We have added the following to the abstract to connect the results to downstream applications: "These results provide a reference for poly(A) tail length estimation analysis, aiding in improving our understanding of the transcriptome and the relationship between poly(A) tail length and other transcriptional mechanisms, including transcript stability or quantification." (lines 32-34)

The language is clear and concise, with only minor grammatical errors. For instance: "We expect that the poly(A) tails shorter than 10 nt occur due to potential truncation…" could be rephrased as "We hypothesize that poly(A) tails shorter than 10 nt may result from truncation…". And the sentence "BoostNano also offers the option of using the Application Programming Interface (API) call instead of the direct method, which omits the file copy implemented in the direct approach, reducing the run time to 8 m 8 s." is verbose and could be simplified for readability.

The first sentence with grammatical errors has been amended (lines 281-282) and the second sentence has been removed due to updates to the results and methodology of our study.

Introduction

The introduction effectively outlines the biological significance of polyadenylation and the technical

challenges in poly(A) tail-length estimation. It provides a clear rationale for using synthetic RNA (Sequins) as a benchmark. However, the scope of the discussion is narrow, with limited exploration of how poly(A) length affects broader transcriptomics applications. Additionally, the references focus on tool-specific papers, but there is a gap in discussing recent advances in nanopore sequencing or polyadenylation biology. One reference could be referenced and that was not cited is Lima SA, Chipman LB, Nicholson AL, Chen YH, Yee BA, Yeo GW, Coller J, Pasquinelli AE. Short poly(A) tails are a conserved feature of highly expressed genes. Nat Struct Mol Biol. 2017 Dec;24(12):1057-1063. doi: 10.1038/nsmb.3499, this reference discusses the broader impact of poly(A) tail dynamics on mRNA decay and transcript stability, which could frame the importance of accurate estimation tools.

We have added this paper as a reference (Lima et al., 2017) as per the reviewer's suggestion in the second paragraph of the "Background" section (page 2). We have also expanded the Background to incorporate a more detailed review of the importance of studying polyadenylation dynamics, recent advances in Nanopore sequencing, and polyadenylation biology.

Methods

3.5/5.0
The methods section is well-detailed, with clear descriptions of dataset preparation, tool execution, and parameter usage. However, there is a lack of explanation regarding the statistical tests used to compare tool performances (e.g., root mean square error minimization).

We have now explained the use of maxpeak (which is the value at which the probability density function achieves its maximum) and MAE for comparing tool performances. (lines 506-514).

Additionally, the choice of gold-standard datasets (R1 and R2 Sequins) is appropriate, but the lack of diversity in tail lengths (only 30 and 60 nt) limits the generalizability of the findings. Including datasets with intermediate or extreme tail lengths could provide a more comprehensive evaluation. A discussion of potential batch effects (e.g., variability in RNA spiking or sequencing conditions) is also missing.

We have added extra datasets derived from eGFP RNA ranging from 10 nt to 150 nt poly(A) tails to improve our study. We believe that batch effects will not affect our study majorly as the same dataset was used across all tools, and our conclusions now stem from the aforementioned additional eGFP dataset from the developers of tailfindr, which adds additional confidence in our observations. However, we have added discussions about the limitation in the lack of sequencing replicates and its effect on more complex transcriptomes without a ground truth (page 13, lines 382-399).

Regarding the statistics used, the authors estimated the accuracy for each method using the mean and standard deviation. However, it is unclear why these metrics were chosen instead of alternatives such as the median and standard error of the mean. Further, the authors model the greatest peak as a normal distribution but do not clarify whether they tested for normality. To strengthen the statistical rigor, I suggest performing a normality test on the values to justify the choice of statistical measures and tests. The rationale for selecting the final statistical method should also be explained. Moreover regarding the statistics, the authors used a root mean square error (RMSE) approach to fit the normal distribution of predicted poly(A) tail lengths. This is appropriate for measuring deviations but fails to address biases in individual datasets. For instance, the difference in standard deviation (SD) between R1 and R2 sets suggests tool-specific variability that could affect downstream analyses. Incorporating additional tests, such as paired t-tests or nonparametric comparisons (e.g., Wilcoxon signed-rank test), could strengthen the statistical rigor.

We have now updated the metrics to include the median and the point at which the probability distribution achieves its maximum (maxpeak) where the latter metric proved to be a favorable method (page 7, Figure 2b). We no longer model the greatest peak as a normal distribution and have removed all instances of referring to this in the manuscript. We also replaced the RMSE approach with the MAE as per the reviewers' suggestions (Figure 2b). We employed bootstrap calculation on the difference between MAEs from different tools to obtain p-values (Data S3).

Finally, the methods section does not provide sufficient details about the computational configurations used (e.g., GPU or CPU specifications, processor type, RAM capacity). These details are critical for reproducibility. I recommend that the authors clearly specify the hardware and software configurations used for both GPU and CPU implementations.

We have added computation configurations as recommended in Table 2 for reproducibility.

Results

The results section is structured and includes sufficient detail to support the conclusions. The comparative analysis between tools is informative, with clear summaries in Table 2 and computational time results in Table 3. However, certain findings, such as the over-representation of short poly(A) tails (<10 nt), are not explored in depth. The analysis would benefit from a discussion of whether this phenomenon is due to RNA degradation or mispriming.

We have carried out additional analyses to explore the 10 nt stretch of sequence downstream of the 3' end of truncated reads and have identified that poly(A) tail degradation/truncation is the most prevalent reason for the short poly(A) tails. Mispriming and read quality were found only contribute to minimal number of reads (Figures 3d-f).

Additionally, while Dorado is identified as the preferred tool, the implications for specific use cases (e.g., low- versus high-throughput experiments) are not discussed.

Implications for specific use cases have been highlighted in the discussion section, and we have noted "while Dorado is identified as the preferred tool, the researcher may prefer accuracy over time, especially in the case of low-throughput datasets. In this case, other methods like tailfindr may be implemented according to specific contexts." (page 12, lines 350-352)

For the R1 test set, the expected mean is not clearly explained. The authors should include a detailed explanation of how this expected mean was derived, including the calculations or assumptions used.

The term "expected mean" was inappropriately used. This phrase was used to describe the known lengths of the Sequins (i.e. 30 nt for R1 and 60 nt for R2). This has been amended for clarity in the manuscript.

Figure 1: in Figure 1, some graphs (e.g., boostnano_R1, boostnano_R2, dorado_R2, tailfindr_R2, nanopolish_R2) display two density peaks, while others do not. It is unclear why this discrepancy exists. I recommend that the authors address this in the results section by explicitly identifying these observations and discussing possible reasons for the differences in the discussion section.

We have explicitly identified these observations in the first paragraph of section "Performance evaluation between BoostNano, tailfindr, nanopolish and Dorado", and discussed the reasons in the discussion section in the third paragraph.

Figure 2: the subset of reads visualized with IGV is useful but underexplored. Additional annotations highlighting the differences between truncated and full-length reads could improve interpretability.

Additional annotations to highlight the truncated and full-length reads were added to improve interpretability (Figure 3a).

Figure 3: while the focus on discarded reads is relevant, the figure does not explore the functional consequences of these differences. Including histograms of read qualities or mapping scores could provide additional insights.

We have added violin plots of read qualities of the discarded reads as Figure 4b.

Table 1: the descriptions are adequate but could include more technical details about how each tool

handles noise or variability in signal.

How each tool handles noise or variability in the signals depends on the way each model has been trained. This information is not described in detail in the current existing publicly available literature, and thus it is unfortunately difficult to describe in the manuscript. Therefore, we will omit this information in the resubmitted manuscript.

Table 2: the summary statistics are clear but lack confidence intervals for mean and SD, which would provide a better sense of variability.

We added medians and maxpeak data with confidence intervals in Data S1.

Table 3: the computational time analysis is thorough. However, it would be beneficial to provide details about hardware specifications (e.g., CPU/GPU model) for reproducibility as already suggested.

We have added computation configurations used in Table 2 for reproducibility.

Discussion

The discussion appropriately interprets the results, emphasizing Dorado's advantages in speed and accuracy. However, it misses an opportunity to provide deeper insights into the limitations of the tools evaluated, particularly BoostNano's tendency to overestimate shorter tails and tailfindr's larger error intervals. The potential impact of sequencing coverage and RNA integrity on poly(A) tail estimation is also underexplored. Besides that, in the discussion section, the authors do not address why the Dorado method does not evaluate the start and end of poly(A) tails in the event space. This omission may influence the observed differences in performance between Dorado, Boostnano, and Tailfindr. I suggest that the authors discuss this limitation and its potential impact on their findings, such as differences in processing speed.

We provide deeper insights into limitations of the tools studied, and we provide the following recommendation based on these tool-based limitations in the first paragraph of the discussion section "Thus, as a general, we recommend the following; should the researcher have specific expected tail lengths for their study, they should choose the most appropriate tool based on the results of this study or similar, with the use of maxpeaks to average over read sets (N>100). In more complex transcriptomes with wider variety of tail lengths, tailfindr or Dorado should be utilized."
We have added a paragraph (paragraph 4) in the discussion section discussing the potential impact of sequencing coverage and RNA integrity on poly(A) tail estimation.

In the initial submission, we noted that the Dorado method does not output the start and end of poly(A) tails, rather than commenting on whether the method does not evaluate the start and end of the poly(A) tails in the event space. In fact, Dorado does measure the signal start and end to calculate the poly(A) tail. However, we have taken the reviewer's comments into account and described the lack of output in the discussion, but it is more likely that additional processing times influence execution times (paragraph 2 of discussion section).

Finally, the authors could elaborate on how these findings influence future tool development or integration into transcriptomics workflows.

A section of future directions for tool development and tool benchmarking has been added in lines 395-399: "Expanding the range of poly(A) lengths to better mimic the distribution in real samples, gaining an enhanced understanding of length-specific biases in each tool and including RNA from diverse preparation methods will enhance this study."

Note:

I previously reviewed this paper previously in Research Hub and you can read these comments via the Research Hub review page here: https://www.researchhub.com/paper/8634403/using-synthetic-rna-to-benchmark-polya-length-inference-from-direct-rna-sequencing/reviews#threadId=55398.

The original preprint linked to the Research Hub review is here:
https://doi.org/10.1101/2024.10.25.620206.

--
Please also take a moment to check our website at
https://www.editorialmanager.com/giga/l.asp?i=198534&l=HCIFXYLQ for any additional comments that were saved as attachments. Please note that as GigaScience has a policy of open peer review, you will be able to see the names of the reviewers.

Cookie Preference Center

We use cookies which are necessary to make our site work. We may also use additional cookies to analyze, improve and personalize our content and your digital experience. For more information, see our Cookie Policy.

You may choose not to allow some types of cookies. However, blocking some types may impact your experience of our site and the services we are able to offer. See the different category headings below to find out more or change your settings.

Allow all

Manage Consent Preferences

Strictly Necessary Cookies

Always active

These cookies are necessary for the website to function and cannot be switched off in our systems. They are usually only set in response to actions made by you which amount to a request for services, such as setting your privacy preferences, logging in or filling in forms. You can set your browser to block or alert you about these cookies, but some parts of the site will not then work.

Functional Cookies

These cookies enable the website to provide enhanced functionality and personalisation. They may be set by us or by third party providers whose services we have added to our pages. If you do not allow these cookies then some or all of these services may not function properly.

Performance Cookies

These cookies allow us to count visits and traffic sources so we can measure and improve the performance of our site. They help us to know which pages are the most and least popular and see how visitors move around the site.

Targeting Cookies

These cookies may be set through our site by our advertising partners. They may be used by those companies to build a profile of your interests and show you relevant adverts on other sites. If you do not allow these cookies, you will experience less targeted advertising.

Cookie List

Clear

Apply Cancel
Consent Leg.Interest

Confirm my choices

Powered by onetrus
