# Supplementary material for: Using synthetic RNA to benchmark poly(A) length inference from direct RNA sequencing
Source: Gigascience. 2025 Sep 3;14:giaf098. doi: 10.1093/gigascience/giaf098 (PMC12406214; doi:10.1093/gigascience/giaf098)
Supplement: giaf098_Authors_Response_To_Reviewer_Comments_Revision_1 [file giaf098_authors_response_to_reviewer_comments_revision_1.pdf]

Author's Response To Reviewer Comments

Dear Editor,

We sincerely appreciate the time and dedication of you and the reviewers to have spent on reviewing this manuscript.

Please kindly find our responses to reviewer #1's comments in blue below.

Reviewer reports:

Reviewer #1: In the revised version the authors present a significantly improved benchmark of tools to estimate polyA tail lengths in Nanopore direct RNA-sequencing data. While they include additional data, the full set is still lacking. Most important would be to include more on RNA004, as most readers will be mainly interested in this.
Some of the newly integrated analyses are not properly explained, as outlined below.
The authors now included a RNA004 data set containing the sequins with two different polyA tail lengths (30 and 60). For a full benchmark that is useful for the field, it would be however required to generate data with a greater dynamic range. On the one hand, the authors show some issues in detection of short polyA tails, on the other hand longer polyA tails are of biological significance.

We understand the importance of expanding the RNA004 dataset and agree with the reviewer that doing so would indeed allow an enhanced understanding of the poly(A) estimation by the different tools. For generating our RNA002 and RNA004 datasets, we have used Sequins which were available as a product. Unfortunately, we are unable to generate synthetic RNA with longer poly(A) tails as we do not possess the capability to do this in our own laboratory. Thus, we instead, expanded the dataset in the RNA002 data (10nt – 150 nt poly(A) tails), using publicly available datasets, as per the reviewer's original recommendations. Despite the shorter tails on the RNA004 datasets, we believe that the data is still useful for understanding the polyadenylome and particularly useful for mitochondrial, viral and plant RNA poly(A) tails, which are commonly within the ranges of the Sequin poly(A) tails. We have added a sentence in the limitations section to clarify this. "While the range for the RNA004 data may be shorter than biologically relevant in mammalian non-mitochondrial RNA, the range fits the expected lengths for mitochondrial, viral and plant RNA poly(A) tails. [PMID: 35982302, PMID: 33961822, PMID: 32330414, PMID: 40355874] (lines 387-389)

Is the new Figure 1C, first panel for GFP transcripts sequenced on RNA002 and analyzed with Dorado? How does such an analysis look for RNA004?

Yes, Figure 1C shows the eGFP transcripts sequenced on RNA002 and analysed with Dorado, which were publicly available. We have clarified this in the Figure 1C legends. Regrettably, we are unable to generate the same data for RNA004 as we lack capacity in our laboratory to generate synthetic IVT RNA.

Introduction: "This raw data is then converted into sequence data using a custom deep learning model, such as Dorado [46] or Chiron [47]". This is what a basecaller does. It is still misleading to mention Chiron as a single tool here but not mentioning Guppy or Rodan basecallers. I understand that it is used in BoostNano, but it should be mentioned only in this context or the other basecallers should be included in the list as well.

We have modified this statement and removed the basecaller examples (Dorado and Chiron):
"This raw data is then converted into sequence data using a custom deep learning model, via the use of basecallers." (lines 91-92)

The new Figures 3B, D, E need to be explained better. Why are the authors only focusing only on reads,

where BoostNano estimates a polyA tail length < 10 nts? It would be sufficient to determine the number of As/ percent A at the read ends of truncated reads. Can the authors provide some whole transcriptome data analysis? This would have a much higher impact for the readers.

The rationale for including Figure 3, is for understanding the smaller peaks (< 10nt) as seen by the distribution plots in all datasets and all poly(A) estimation methods, as this was an unexpected result. As we wanted to isolate the reads < 10 nt, we had to choose one method to base our threshold, and we chose BoostNano as it showed the most prominent first peak. As a benchmarking manuscript with a focus on synthetic RNA to use ground truth datasets, we believe that adding results using whole transcriptomes is out of the scope of this manuscript. While this may be beneficial for the readers, investigating the polyadenylome in an organism will likely require the amount of work to synthesize a separate publication, and may not aid deeply in terms of comparing the variation between the tools. We have recently published some work regarding the poly(A) distributions using RNA002 called with Dorado in human blood mRNA (PMID: 40355874) and have recognized that short poly(A) tails are also shown through these datasets (~<10 nt), highlighting that shorter poly(A) tails are also found in vivo. We wish to note that many of the datasets available using direct RNA-sequencing are derived from RNA002 and it is still important to understand the performance differences suitable for RNA002 data. We have added the following future directions to the limitations section: “Overall, future work would benefit from expanding the range of poly(A) lengths to better mimic the distribution in real samples via synthetic and whole transcriptome data, gaining an enhanced understanding of length-specific biases in each tool and including RNA from diverse preparation methods.” (lines 389-392).

The authors still argue that the RTA adapter may be degraded and now cite the work by Davis et al to support this. However, in this work they show that especially T-mers are stable over at least 70 freeze-and-thaw cycles. Based on the kit size of six reactions, the RTA should undergo maximal 5 freeze-and-thaw cycles and should be considered stable. Thus, I recommend to remove this statement.

We have removed this statement. In its place, we investigated the alternative idea of split-reads during the signal detection stage: “We hypothesized that poly(A) tails shorter than 10 nt may result from signal glitches during the signal detection, where one read may be written as multiple reads. Using Bulkvis, we discovered that among a total of 60,146 reads from all 7 RNA002 samples, 270 pairs of split reads were found (0.898%), with 26 pairs including one read in the list of reads with < 10 nt poly(A) tails (Data S4). This suggests that while read splits may partially explain the shorter poly(A) tails, other unexplained mechanisms are at play.” (lines 269-274)

I don't understand Figures 3G and H. The authors select reads, for which BoostNano estimates very short polyA tails. For these reads, the other tools estimate polyA tail lengths which are closer to the expected length. Strictly spoken, this shows mainly that BoostNano should not be considered for estimating short polyA tail lengths.

The distribution plots show that the other tools (especially Nanopolish and Tailfindr) also exhibit a peak at a similar point as BoostNano if we take the maximum peak of density. However, we agree with the reviewer that BoostNano does indeed perform worst in terms of short poly(A) tails. We have added/amended to the following: “Our analysis revealed that while all four poly(A) estimation methods consistently identified shorter poly(A) tails, BoostNano exhibited a narrower peak for these shorter tails, whereas Dorado tended to estimate longer poly(A) tails that were closer to the known values (Figures 3g-h). Given that tailfindr and nanopolish also exhibited a peak at similar points in the density distributions as BoostNano, Dorado likely overestimates very short tails. Overall, the narrow peak of BoostNano indicates that BoostNano may not be suitable for estimating shorter poly(A) tails compared with the other tools.” (lines 239-245).

Cookie Preference Center

We use cookies which are necessary to make our site work. We may also use additional cookies to analyze, improve and personalize our content and your digital experience. For more information, see our Cookie Policy.

You may choose not to allow some types of cookies. However, blocking some types may impact your experience of our site and the services we are able to offer. See the different category headings below to find out more or change your settings.

Allow all

Manage Consent Preferences

Strictly Necessary Cookies

Always active

These cookies are necessary for the website to function and cannot be switched off in our systems. They are usually only set in response to actions made by you which amount to a request for services, such as setting your privacy preferences, logging in or filling in forms. You can set your browser to block or alert you about these cookies, but some parts of the site will not then work.

Functional Cookies

These cookies enable the website to provide enhanced functionality and personalisation. They may be set by us or by third party providers whose services we have added to our pages. If you do not allow these cookies then some or all of these services may not function properly.

Performance Cookies

These cookies allow us to count visits and traffic sources so we can measure and improve the performance of our site. They help us to know which pages are the most and least popular and see how visitors move around the site.

Targeting Cookies

These cookies may be set through our site by our advertising partners. They may be used by those companies to build a profile of your interests and show you relevant adverts on other sites. If you do not allow these cookies, you will experience less targeted advertising.

Cookie List

Clear

Apply Cancel

Consent Leg.Interest

Confirm my choices

Powered by onetrust
