# Supplementary material for: Using synthetic RNA to benchmark poly(A) length inference from direct RNA sequencing
Source: Gigascience. 2025 Sep 3;14:giaf098. doi: 10.1093/gigascience/giaf098 (PMC12406214; doi:10.1093/gigascience/giaf098)
Supplement: giaf098_Reviewer_1_Report_Original_Submission — Christoph Dieterich -- 12/20/2024 [file giaf098_reviewer_1_report_original_submission.pdf]

Reviewer Report

Title: Using synthetic RNA to benchmark poly(A) length inference from direct RNA sequencing

Version: Original Submission **Date:** 12/20/2024

Reviewer name: Christoph Dieterich

Reviewer Comments to Author:

In this manuscript, the authors present a benchmark to assess the performance of different tools designed for estimation of polyA tail length from Nanopore direct RNA-sequencing data. These tools include tailfindr, nanopolish, Dorado and Boost Nano.Benchmarks on tools and algorithms to analyze Nanopore data, both third party tools and official ONT releases, are of utmost importance for the field. The use of synthetic constructs with known ground truth is recommended as well. Consequently, this study has the potential to provide a significant contribution to the field.In the current form, I can however not recommend it for publication in GigaScience. My major concerns are:a) Use of only RNA002 data. This chemistry is outdated and thus the Benchmark is only relevant for old, possibly already published data. A comprehensive Benchmark should also include RNA004 and available tools there (at least Dorado).b) The current data set only contains two polyA tail length, which are relatively short and do not cover longer polyA tails that are common e.g. in mammalian cells. A proper Benchmark should show the performance of the analyzed tools over a range of polyA tail lengths.Minor comments:1) Abstract: "All four tools generate mean tail-length estimates which lie within 13% of the correct value." The value of 13% is given in the Abstract from the submission system, wherease the abstract in the Main text says 12%. Which value is correct?2) Background, first paragraph: the role of the polyA tail in RNA circularization, which is required for efficient translation of cellular mRNAs is not mentioned. Reference is missing for "is increasingly recognised as a dynamic process which influences timing and degree of protein production."3) Background, second paragraph: Chiron seems to be a relatively old basecaller (no models for new chemistries). It should be mentioned here that it is required for BoostNano.4) Mis-priming of internal polyA sites may an important confounding (and currently overlooked) source of errors in Nanopore sequencing. This should be quantified properly and analyzed in more detail (length of these stretches, influence of other nucleotides within the A-rich stretch, etc.). Should be done as well on whole transcriptome data with more possible mispriming sites.5) Why do the authors think that the poly(T) stretch of the RTA might be truncated? This is composed of DNA oligos, which should be quite stable6) What are the parameters for filtering used by Dorado and BoostNano? Can the authors explain, why the filtered reads differ?7) Dorado seems to systematically underestimate polyA tail length. Is this true also for data generated with RNA004 chemistry and longer polyA tails?

Level of Interest

Please indicate how interesting you found the manuscript: Choose an item.

Quality of Written English

Please indicate the quality of language in the manuscript: Choose an item.

Declaration of Competing Interests

Please complete a declaration of competing interests, considering the following questions:

- Have you in the past five years received reimbursements, fees, funding, or salary from an organisation that may in any way gain or lose financially from the publication of this manuscript, either now or in the future?
- Do you hold any stocks or shares in an organisation that may in any way gain or lose financially from the publication of this manuscript, either now or in the future?
- Do you hold or are you currently applying for any patents relating to the content of the manuscript?
- Have you received reimbursements, fees, funding, or salary from an organization that holds or has applied for patents relating to the content of the manuscript?
- Do you have any other financial competing interests?
- Do you have any non-financial competing interests in relation to this paper?

If you can answer no to all of the above, write 'I declare that I have no competing interests' below. If your reply is yes to any, please give details below.

I declare that I have no competing interests

I agree to the open peer review policy of the journal. I understand that my name will be included on my report to the authors and, if the manuscript is accepted for publication, my named report including any attachments I upload will be posted on the website along with the authors' responses. I agree for my report to be made available under an Open Access Creative Commons CC-BY license (http://creativecommons.org/licenses/by/4.0/). I understand that any comments which I do not wish to be included in my named report can be included as confidential comments to the editors, which will not be published.

Choose an item.

To further support our reviewers, we have joined with Publons, where you can gain additional credit to further highlight your hard work (see: https://publons.com/journal/530/gigascience). On publication of this paper, your review will be automatically added to Publons, you can then choose whether or not to claim your Publons credit. I understand this statement.

Yes Choose an item.
