# Supplementary material for: Using synthetic RNA to benchmark poly(A) length inference from direct RNA sequencing
Source: Gigascience. 2025 Sep 3;14:giaf098. doi: 10.1093/gigascience/giaf098 (PMC12406214; doi:10.1093/gigascience/giaf098)
Supplement: giaf098_Reviewer_1_Report_Revision_1 — Christoph Dieterich -- 5/27/2025 [file giaf098_reviewer_1_report_revision_1.pdf]

Reviewer Report

Title: Using synthetic RNA to benchmark poly(A) length inference from direct RNA sequencing

Version: Revision 1 **Date:** 5/27/2025

Reviewer name: Christoph Dieterich

Reviewer Comments to Author:

In the revised version the authors present a significantly improved benchmark of tools to estimate polyA tail lengths in Nanopore direct RNA-sequencing data. While they include additional data, the full set is still lacking. Most important would be to include more on RNA004, as most readers will be mainly interested in this.Some of the newly integrated analyses are not properly explained, as outlined below.The authors now included a RNA004 data set containing the sequins with two different polyA tail lengths (30 and 60). For a full benchmark that is useful for the field, it would be however required to generate data with a greater dynamic range. On the one hand, the authors show some issues in detection of short polyA tails, on the other hand longer polyA tails are of biological significance.Is the new Figure 1C, first panel for GFP transcripts sequenced on RNA002 and analyzed with Dorado? How does such an analysis look for RNA004?Introduction: "This raw data is then converted into sequence data using a custom deep learning model, such as Dorado [46] or Chiron [47]". This is what a basecaller does. It is still misleading to mention Chiron as a single tool here but not mentioning Guppy or Rodan basecallers. I understand that it is used in BoostNano, but it should be mentioned only in this context or the other basecallers should be included in the list as well.The new Figures 3B, D, E need to be explained better. Why are the authors only focusing only on reads, where BoostNano estimates a polyA tail length < 10 nts? It would be sufficient to determine the number of As/ percent A at the read ends of truncated reads. Can the authors provide some whole transcriptome data analysis? This would have a much higher impact for the readers.The authors still argue that the RTA adapter may be degraded and now cite the work by Davis et al to support this. However, in this work they show that especially T-mers are stable over at least 70 freeze-and-thaw cycles. Based on the kit size of six reactions, the RTA should undergo maximal 5 freeze-and-thaw cycles and should be considered stable. Thus, I recommend to remove this statement.I don't understand Figures 3G and H. The authors select reads, for which BoostNano estimates very short polyA tails. For these reads, the other tools estimate polyA tail lengths which are closer to the expected length. Strictly spoken, this shows mainly that BoostNano should not be considered for estimating short polyA tail lengths.

Level of Interest

Please indicate how interesting you found the manuscript: Choose an item.

Quality of Written English

Please indicate the quality of language in the manuscript: Choose an item.

Declaration of Competing Interests

Please complete a declaration of competing interests, considering the following questions:

- Have you in the past five years received reimbursements, fees, funding, or salary from an organisation that may in any way gain or lose financially from the publication of this manuscript, either now or in the future?
- Do you hold any stocks or shares in an organisation that may in any way gain or lose financially from the publication of this manuscript, either now or in the future?
- Do you hold or are you currently applying for any patents relating to the content of the manuscript?
- Have you received reimbursements, fees, funding, or salary from an organization that holds or has applied for patents relating to the content of the manuscript?
- Do you have any other financial competing interests?
- Do you have any non-financial competing interests in relation to this paper?

If you can answer no to all of the above, write 'I declare that I have no competing interests' below. If your reply is yes to any, please give details below.

I declare that I have no competing interests

I agree to the open peer review policy of the journal. I understand that my name will be included on my report to the authors and, if the manuscript is accepted for publication, my named report including any attachments I upload will be posted on the website along with the authors' responses. I agree for my report to be made available under an Open Access Creative Commons CC-BY license (http://creativecommons.org/licenses/by/4.0/). I understand that any comments which I do not wish to be included in my named report can be included as confidential comments to the editors, which will not be published.

Choose an item.

To further support our reviewers, we have joined with Publons, where you can gain additional credit to further highlight your hard work (see: https://publons.com/journal/530/gigascience). On publication of this paper, your review will be automatically added to Publons, you can then choose whether or not to claim your Publons credit. I understand this statement.

Yes Choose an item.
