# Supplementary material for: Using synthetic RNA to benchmark poly(A) length inference from direct RNA sequencing
Source: Gigascience. 2025 Sep 3;14:giaf098. doi: 10.1093/gigascience/giaf098 (PMC12406214; doi:10.1093/gigascience/giaf098)
Supplement: giaf098_Reviewer_2_Report_Original_Submission — Jesse Daniel Brown -- 1/7/2025 [file giaf098_reviewer_2_report_original_submission.pdf]

**Reviewer Report**

**Title: Using synthetic RNA to benchmark poly(A) length inference from direct RNA sequencing**

**Version: Original Submission** **Date:** 1/7/2025

**Reviewer name: Jesse Daniel Brown**

**Reviewer Comments to Author:**

This manuscript addresses a relevant and timely question: benchmarking poly(A) tail-length estimation tools (BoostNano, tailfindr, nanopolish, and Dorado) using synthetic RNA standards (Sequins) with known tail lengths. Poly(A) tail-length estimation is increasingly important for understanding mRNA stability, processing, and regulation at the single-molecule level. As direct RNA sequencing expands in use, reliable methods to measure poly(A) tail lengths are needed. The study's desiignâ€”leveraging Sequins as a "gold standard" to benchmark toolsâ€”is strong and fills an area is need in current literature. The analysis is thorough in its basic comparisons, and the results are likely to be useful to researchers who need to choose suitable software for poly(A) tail analysis. However, the manuscript would benefit from deeper contextualization, more rigorous statistical methodology, and clearer reporting of computational details. Ensuring reproducibility and providing clearer guidance on interpreting the results in real biological contexts would strengthen the mannuscript. The suggestions below are aimed at making the study more valuable to the community.For this reason, my recommendation is Revisions ARE NeededIntroductionAbstract: â˜…â˜…â˜…â˜…â˜† (4/5) Actually in place of the introduction, it has it strengths:The introduction adequately outlines why polyadenylation is biologically important and why direct RNA sequencing provides a unique opportunity for poly(A) tail-length estimation.It justifies the use of Sequins as synthetic standards, which is a robust approach to derive ground-truth tail lengths.Areas for Improvement:The introduction could better connect poly(A) tail-length estimation to downstream applications. For instance, mention how accurate tail-length estimation could improve understanding of mRNA decay rates, translation efficiency, or isoform-specific regulation.Adding references that contextualize poly(A) tail dynamics in broader biological phenomena would help readers understand the significance.For example, it is almost a necessity to cite work such as "Roles of mRNA poly(A) tails in regulation of eukaryotic gene expression" by Lori A. Passmore & Jeff Coller (2022, Nature Reviews Molecular Cell Biology) which provides a comprehensive analysis of poly(A) tail dynamics and their impact on mRNA decay, stability, and translation regulation. P & C (2022) also expands on these principles by discussing the mechanistic underpinnings of poly(A)-mediated decay and translation regulation, making it a broader and more recent contribution to polyadenylation biology, which the authors should consider.Grammar of the abstract:Error: "There are currently several tools available for poly(A) tail-length estimation, including well-established tools such as tailfindr and nanopolish, as well as two more recent deep learning models: Dorado and BoostNano."Suggestion: "Several tools are currently available for poly(A) tail-length estimation, including well-established methods like tailfindr and nanopolish, as well as two more recent deep learning models: Dorado and BoostNano."Error: "which lie within 12% of the correct value."Suggestion: "that lie within 12% of the correct value."Clarify the library preparation steps to avoid confusion about the "direct" nature of RNA

sequencing. The text currently implies that no reverse transcription is required, but then references an ONT Reverse Transcription Adapter. Distinguish between a full-length cDNA synthesis step (not required) and the use of a poly(T)-containing adapter for sequencing library preparation.MethodsMethods: â˜…â˜…â˜…â˜…â˜† (4/5)The methods section has its strengths; the data sources and preparation (Sequins spiked into host RNA) are clearly described. Versions of tools are provided, enhancing reproducibility.Areas for Improvement are statistical analysis, comparisons and tests, hardware and computation details, and understanding of run time differences.Currently, the study models distributions as normal and uses mean and SD, but no normality tests or justification for these choices are presented. Consider performing normality tests or using nonparametric measures. Additionally, providing confidence intervals or other robust statistics (median, interquartile ranges) would clarify variability.For the comparisons and tests, the authors should explain why you chose root mean square error (RMSE) minimization and other metrics.Could alternative tests, like Wilcoxon signed-rank tests or paired t-tests (Wilcocoxon: this non-parametric test is suitable for paired comparisons when the assumption of normality is not met. -useful to compare the predicted tail lengths from each tool against the expected lengths, especially if the data distribution is skewed.), be used to compare the distribution of tail-length estimates more rigorously?Paired t-Test, because this test could be applied if the normality assumption holds, providing a straightforward way to assess whether the mean difference between predicted and expected values is statistically significant. (If so, justification should be provided for why or why not)There are some additional metrics to explore:---Median Absolute Deviation (MAD): Consider adding MAD as it is robust to outliers and could complement RMSE to provide a better understanding of central tendencies and variability.---Mean Absolute Error (MAE): MAE is another alternative that simplifies the interpretation by focusing solely on the magnitude of errors without squaring them, potentially offering more intuitive insights for readers.The authors should address testing for normality, explicitly stating whether normality tests were conducted on the data (e.g., Shapiro-Wilk or Kolmogorov-Smirnov tests). If normality is confirmed, justify the use of parametric tests like RMSE or t-tests. If not, justify why non-parametric tests (e.g., Wilcoxon) were not employed or discuss plans to include them in future studies.Explain the choice of statistical methods over time by discussing how the choice of statistical tests aligns with the study's goals. For example, emphasize whether the focus was on understanding overall error distribution, tool consistency, or accuracy in predicting specific tail lengths.The authors could use visual representations of error complementing the statistical tests with visual aids such as boxplots, violin plots, or Bland-Altman plots to illustrate the error distributions and discrepancies between predicted and actual tail lengths across tools.The authors should provide hardware and computational details like providing explicit details on the computational environmentâ€”CPU/GPU models, RAM, OSâ€”for each tool's run. While the Git-hub read me suggests how to run the system, it lacks any details about system requirements.Readers need this to understand runtime differences and attempt to replicate performance measurements.The authors should consider tool parameterization and indicate if any specific parameters (beyond defaults) were used in tailfindr, nanopolish, Dorado, or BoostNano runs. If no changes were made from defaults, state this explicitly.ResultsThe result's strengths are that they are presented clearly, showing density distributions and discussing short-tail anomalies. The identification of Dorado as a preferred tool due to speed, integration, and conservative filtering is well-supported by the data. The study acknowledges that all tools achieve broadly similar accuracy, differing mainly in runtime and filtering criteria, which is a

practical insight for users.The results have areas for improvement:Regrading the short-tail reads explanation, the authors attribute short (<10 nt) poly(A) tails to truncated transcripts or mis-priming. For this reason, it is suggested that the authors strengthen this discussion with additional evidence or reasoning. For instance, is there a correlation between read quality and short-tail length estimates? Do truncated reads consistently align to internal A-rich stretches?Multiple peaks in distributions: Some density plots (Figure 1) show multiple peaks or shoulder peaks.Discuss potential reasons for these patterns. Are they related to tool-specific biases, read quality, or adapter/poly(T) truncation?Application Context: The results focus on method performance, but it would help readers to understand how these differences might influence downstream tasks. For example, if a method overestimates poly(A) length slightly, how could this affect conclusions about RNA stability or differential tail-length analysis between experimental conditions?Figures and tables:Figure 1:Clear density plots, but consider adding vertical lines at expected tail lengths (30 nt and 60 nt) to guide interpretation. Splitting the figure into separate panels for R1 and R2 or using insets might clarify multiple peaks.Figure 2:The IGV snapshots are informative. Enhance interpretability by adding annotations (arrows or boxes) highlighting truncated vs. full-length reads. Increase font sizes for readability.Figure 3:Useful comparison of reads filtered by Dorado but retained by BoostNano.Add a brief note or labeling to indicate expected tail lengths.Discuss possible reasons for Dorado's conservative filtering here or in the main text.Tables:Provide definitions for abbreviations (nt, CPU, GPU) in captions. For Table 2, adding confidence intervals around the mean tail-length estimates would strengthen statistical rigor. For Table 3, specify hardware details as recommended above.Grammar Mistakes and errors in the results section:Results Section:Sentence: "The four methods display a similar pattern in the density distribution, with a prominent normal-like peak near the expected poly(A) length, but also with a over-representation of shorter poly(A) tails, ranging at approximately ~0-10 nt (Figure 1)."Issue: "a over-representation"Correction: "an over-representation"Sentence: "We expected that these shorter peaks were derived from either fragmentation of the transcript, mis-priming of internal poly(A) stretches or degradation of the poly(A) tails."Issue: tense mismatch ("expected" vs. "were derived").Correction: "We expect" -- "were derived", loses context and tense contformity-- therefore the sentence should be adjusted-"We hypothesize that these shorter peaks are derived from either fragmentation of the transcript, mis-priming of internal poly(A) stretches, or degradation of the poly(A) tails."Sentence: "Interestingly, upon investigating these earlier peaks, we found that Dorado excludes reads which are retained in the analysis by BoostNano, despite them being classified as passed reads (Figure 3)."Issue: Ambiguous pronoun "them." (them could incorrectly identify three possible targets in the sentence)Correction: "Interestingly, upon investigating these earlier peaks, we found that Dorado excludes reads retained in the analysis by BoostNano, even though these reads are classified as passed reads (Figure 3)."Sentence: "Therefore, Dorado appears to be a more conservative approach than BoostNano."Issue: No grammar issues, but the statement could be more precise.Suggested improvement: "Thus, Dorado demonstrates a more conservative approach compared to BoostNano."Sentence: "In order to determine which normal distribution fit the peak best, we found the parameters (mean, SD) which minimize the root mean square error between the candidate normal distribution and the density distribution for an interval of 10 nt to the right of the mode."Issue: Verb tense consistency ("fit").Correction: "To determine which normal distribution fits the peak best, ..."Sentence: "The peaks also lose their normal-like behavior for larger values."Issue: Could use a more formal tone. Correction: "The peaks also deviate from their

normal-like behavior at larger values."Sentence: "Next, we compared the computational time required by each method to predict the tail-length of 4000 reads."Issue: Hyphenation of "tail-length."Correction: "Next, we compared the computational time required by each method to predict the tail length of 4,000 reads."Sentence: "BoostNano also offers the option of using the Application Programming Interface (API) call instead of the direct method, which omits the file copy implemented in the direct approach, reducing the run time to 8 m 8 s."Here, the sentence is extremely overwritten which cuases a lack of clarity.Correction: "BoostNano offers an alternative API-based method, which skips the file copy step of the direct approach, reducing the runtime to 8 minutes and 8 seconds."DiscussionDiscussion: â˜…â˜…â˜…â˜†â˜† (3/5)The discussion as its strengths as it correctly identifies that Dorado's advantages (speed, integration with basecalling) make it appealing as a default choice.The authors acknowledge that all tools are within a similar accuracy range, suggesting the deciding factor may be speed or integration rather than raw performance differences.HOWEVER- there are areas for improvement:Further dissect the limitations of each tool. For example, BoostNano shows good SD but slightly off mean for R1; what does this mean for its use cases?Address the discrepancy between tailfindr, nanopolish, and Dorado in terms of how they define and detect poly(A) boundaries. Why does Dorado not evaluate start/end positions of poly(A) tails in event space, and how might this influence results?Include a brief discussion about how results might generalize to more complex transcriptomes. Real samples have varying GC content, fragment lengths, and potentially modified bases. A short commentary acknowledging these factors would show awareness that synthetic standards cannot capture the full complexity of natural RNA opulations.For these reasons, it is suggested that the authors suggest future directions.For instance, how could tool developers incorporate these findings to improve their methods? Could future benchmarking sets include a gradient of tail lengths to better understand length-specific biases?Grammar Mistakes and errors in the discussion section:Sentence: "BoostNano and tailfindr tools provided estimation of the starting and ending positions of the poly(A) tails in event space while this information was absent in Dorado outputs."Issue: "provided estimation" should be "provide estimation" to align with present tense.Correction: "BoostNano and tailfindr tools provide estimation of the starting and ending positions of the poly(A) tails in event space, while this information is absent in Dorado outputs."Sentence: "On the R1 dataset, BoostNano showed a tighter distribution with the smallest SD, but its peak was the furthest from the correct value."The issue here is that the test results are still speaking about genneral truths leading to verb tense inconsistency; "showed" should match other verbs in the section.Correction: "On the R1 dataset, BoostNano shows a tighter distribution with the smallest SD, but its peak is the furthest from the correct value."Sentence: "tailfindr had the most accurate estimation but also the largest error interval."The issue here is the verb tense mismatch; "had" should be consistent with present tense to show truth, not past truth.Correction: "tailfindr has the most accurate estimation but also the largest error interval."Sentence: "Furthermore, Boostnano is more lenient in keeping reads for poly(A) estimation than Dorado."Issue: "Boostnano" capitalization error; it should be "BoostNano."Correction: "Furthermore, BoostNano is more lenient in keeping reads for poly(A) estimation than Dorado."Sentence: "Overall, our results suggest that the four tools investigated in this study - BoostNano, tailfindr, nanopolish and Dorado have similar performance with their accuracy varying from one dataset to the other, with a potential length bias."Issue: Missing commas for clarity; replace "with their accuracy varying from one dataset to the other" for conciseness.Correction: "Overall, our results suggest that the four tools investigated in this

studyâ€"BoostNano, tailfindr, nanopolish, and Doradoâ€"have similar performance, with accuracy varying across datasets and showing potential length bias."Sentence: "Therefore, we expect Dorado to be implemented as the default method of poly(A) tail estimation in the near future, with the rapid estimation timeframe, comparable estimation lengths to other tools, conservative nature and the added benefit of ease of obtaining this information during basecalling."There are several issues here including verbosity and lack of parallelism.Correction: "Therefore, we expect Dorado to be implemented as the default method for poly(A) tail estimation, given its rapid estimation timeframe, comparable accuracy to other tools, conservative nature, and ease of integration with basecalling."Sentence: "This work demonstrates the value of having access to synthetic RNA molecules with known poly(A) tail-lengths for validating the accuracy of poly(A) tail estimation algorithms."Issue: The phrase "validating the accuracy of" could be simplified for readability.Correction: "This work demonstrates the value of synthetic RNA molecules with known poly(A) tail lengths for validating poly(A) tail estimation algorithms."Sentence: "As methods improve, we anticipate that these datasets will be valuable for assessing improvements in estimation of poly(A) tails."Issue: "improvements in estimation of" is awkward.Correction: "As methods improve, we anticipate that these datasets will be valuable for assessing advancements in poly(A) tail estimation."References need to be added to accomodate the suggested material review, but existing references are good-NEEDS REVISIONJesse Daniel Brown PD AASUNote:I previously reviewed this paper previously in Research Hub and you can read these comments via the Research Hub review page here: https://www.researchhub.com/paper/8634403/using-synthetic-rna-to-benchmark-polya-length-inference-from-direct-rna-sequencing/reviews#threadId=55398.The original preprint linked to the Research Hub review is here: https://doi.org/10.1101/2024.10.25.620206

**Level of Interest**

Please indicate how interesting you found the manuscript: Choose an item.

**Quality of Written English**

Please indicate the quality of language in the manuscript: Choose an item.

**Declaration of Competing Interests**

Please complete a declaration of competing interests, considering the following questions:

- Have you in the past five years received reimbursements, fees, funding, or salary from an organisation that may in any way gain or lose financially from the publication of this manuscript, either now or in the future?
- Do you hold any stocks or shares in an organisation that may in any way gain or lose financially from the publication of this manuscript, either now or in the future?
- Do you hold or are you currently applying for any patents relating to the content of the manuscript?

- Have you received reimbursements, fees, funding, or salary from an organization that holds or has applied for patents relating to the content of the manuscript?
- Do you have any other financial competing interests?
- Do you have any non-financial competing interests in relation to this paper?

If you can answer no to all of the above, write 'I declare that I have no competing interests' below. If your reply is yes to any, please give details below.

I declare that I have no competing interests

I agree to the open peer review policy of the journal. I understand that my name will be included on my report to the authors and, if the manuscript is accepted for publication, my named report including any attachments I upload will be posted on the website along with the authors' responses. I agree for my report to be made available under an Open Access Creative Commons CC-BY license (http://creativecommons.org/licenses/by/4.0/). I understand that any comments which I do not wish to be included in my named report can be included as confidential comments to the editors, which will not be published.

Choose an item.

To further support our reviewers, we have joined with Publons, where you can gain additional credit to further highlight your hard work (see: https://publons.com/journal/530/gigascience). On publication of this paper, your review will be automatically added to Publons, you can then choose whether or not to claim your Publons credit. I understand this statement.

Yes Choose an item.
