# Supplementary material for: Using synthetic RNA to benchmark poly(A) length inference from direct RNA sequencing
Source: Gigascience. 2025 Sep 3;14:giaf098. doi: 10.1093/gigascience/giaf098 (PMC12406214; doi:10.1093/gigascience/giaf098)
Supplement: giaf098_Reviewer_3_Report_Original_Submission — Paulo Gattai -- 1/7/2025 [file giaf098_reviewer_3_report_original_submission.pdf]

Reviewer Report

Title: Using synthetic RNA to benchmark poly(A) length inference from direct RNA sequencing

Version: Original Submission **Date:** 1/7/2025

Reviewer name: Paulo Gattai

Reviewer Comments to Author:

The manuscript presents interesting findings, but several points require clarification and further discussion to enhance transparency, statistical robustness, and reproducibility. Addressing the discrepancies in Figure 1, justifying the choice of statistical methods, and providing detailed computational configurations will significantly improve the manuscript. Based on the above stated, my recommendation it is by accepting for publication after a major revision.Regarding the criteria from GigaScience Journal for Technical Notes this technical note comply partially with technical improvement/utility, because while the study demonstrates that Dorado is the fastest and most conservative method, the novelty of technical improvement is somewhat limited because the tools themselves are not newly developed by the authors (exception of BoostNano). That said, the benchmarking study provides value for the research community by helping users choose the most appropriate tool for poly(A) tail-length estimation.The reviewer declare that he does not have competing interests.The abstract provides a concise summary of the study's aim, methodology, and key findings. However, it lacks sufficient emphasis on the broader implications of benchmarking poly(A) tail-length estimation for transcriptomics research. The abstract mentions the advantages of Dorado in terms of speed and accuracy but does not explicitly describe the limitations of other tools or potential use cases of these findings. Expanding the abstract to connect the results to downstream applications such as transcript stability analysis or isoform quantification could improve its utility.The language is clear and concise, with only minor grammatical errors. For instance: "We expect that the poly(A) tails shorter than 10 nt occur due to potential truncation…" could be rephrased as "We hypothesize that poly(A) tails shorter than 10 nt may result from truncation…". And the sentence "BoostNano also offers the option of using the Application Programming Interface (API) call instead of the direct method, which omits the file copy implemented in the direct approach, reducing the run time to 8 m 8 s." is verbose and could be simplified for readability.IntroductionThe introduction effectively outlines the biological significance of polyadenylation and the technical challenges in poly(A) tail-length estimation. It provides a clear rationale for using synthetic RNA (Sequins) as a benchmark. However, the scope of the discussion is narrow, with limited exploration of how poly(A) length affects broader transcriptomics applications. Additionally, the references focus on tool-specific papers, but there is a gap in discussing recent advances in nanopore sequencing or polyadenylation biology. One reference could be referenced and that was not cited is Lima SA, Chipman LB, Nicholson AL, Chen YH, Yee BA, Yeo GW, Coller J, Pasquinelli AE. Short poly(A) tails are a conserved feature of highly expressed genes. Nat Struct Mol Biol. 2017 Dec;24(12):1057-1063. doi: 10.1038/nsmb.3499, this reference discusses the broader impact of poly(A) tail dynamics on mRNA decay and transcript stability, which could frame the importance of accurate estimation tools.Methods3.5/5.0The methods section is well-detailed, with clear descriptions of dataset

preparation, tool execution, and parameter usage. However, there is a lack of explanation regarding the statistical tests used to compare tool performances (e.g., root mean square error minimization). Additionally, the choice of gold-standard datasets (R1 and R2 Sequins) is appropriate, but the lack of diversity in tail lengths (only 30 and 60 nt) limits the generalizability of the findings. Including datasets with intermediate or extreme tail lengths could provide a more comprehensive evaluation. A discussion of potential batch effects (e.g., variability in RNA spiking or sequencing conditions) is also missing. Regarding the statiscs used, the authors estimated the accuracy for each method using the mean and standard deviation. However, it is unclear why these metrics were chosen instead of alternatives such as the median and standard error of the mean. Further, the authors model the greatest peak as a normal distribution but do not clarify whether they tested for normality. To strengthen the statistical rigor, I suggest performing a normality test on the values to justify the choice of statistical measures and tests. The rationale for selecting the final statistical method should also be explained. Moreover regarding the statistics, the authors used a root mean square error (RMSE) approach to fit the normal distribution of predicted poly(A) tail lengths. This is appropriate for measuring deviations but fails to address biases in individual datasets. For instance, the difference in standard deviation (SD) between R1 and R2 sets suggests tool-specific variability that could affect downstream analyses. Incorporating additional tests, such as paired t-tests or nonparametric comparisons (e.g., Wilcoxon signed-rank test), could strengthen the statistical rigor. Finally, the methods section does not provide sufficient details about the computational configurations used (e.g., GPU or CPU specifications, processor type, RAM capacity). These details are critical for reproducibility. I recommend that the authors clearly specify the hardware and software configurations used for both GPU and CPU implementations.ResultsThe results section is structured and includes sufficient detail to support the conclusions. The comparative analysis between tools is informative, with clear summaries in Table 2 and computational time results in Table 3. However, certain findings, such as the over-representation of short poly(A) tails (<10 nt), are not explored in depth. The analysis would benefit from a discussion of whether this phenomenon is due to RNA degradation or mispriming. Additionally, while Dorado is identified as the preferred tool, the implications for specific use cases (e.g., low- versus high-throughput experiments) are not discussed. For the R1 test set, the expected mean is not clearly explained. The authors should include a detailed explanation of how this expected mean was derived, including the calculations or assumptions used.Figure 1: in Figure 1, some graphs (e.g., boostnano_R1, boostnano_R2, dorado_R2, tailfindr_R2, nanopolish_R2) display two density peaks, while others do not. It is unclear why this discrepancy exists. I recommend that the authors address this in the results section by explicitly identifying these observations and discussing possible reasons for the differences in the discussion section.Figure 2: the subset of reads visualized with IGV is useful but underexplored. Additional annotations highlighting the differences between truncated and full-length reads could improve interpretability.Figure 3: while the focus on discarded reads is relevant, the figure does not explore the functional consequences of these differences. Including histograms of read qualities or mapping scores could provide additional insights.Table 1: the descriptions are adequate but could include more technical details about how each tool handles noise or variability in signal.Table 2: the summary statistics are clear but lack confidence intervals for mean and SD, which would provide a better sense of variability.Table 3: the computational time analysis is thorough. However, it would be beneficial to provide details about hardware specifications (e.g., CPU/GPU model) for reproducibility as already suggested.DiscussionThe discussion

appropriately interprets the results, emphasizing Dorado's advantages in speed and accuracy. However, it misses an opportunity to provide deeper insights into the limitations of the tools evaluated, particularly BoostNano's tendency to overestimate shorter tails and tailfindr's larger error intervals. The potential impact of sequencing coverage and RNA integrity on poly(A) tail estimation is also underexplored. Besides that, in the discussion section, the authors do not address why the Dorado method does not evaluate the start and end of poly(A) tails in the event space. This omission may influence the observed differences in performance between Dorado, Boostnano, and Tailfindr. I suggest that the authors discuss this limitation and its potential impact on their findings, such as differences in processing speed. Finally, the authors could elaborate on how these findings influence future tool development or integration into transcriptomics workflows.Note:I previously reviewed this paper previously in Research Hub and you can read these comments via the Research Hub review page here: https://www.researchhub.com/paper/8634403/using-synthetic-rna-to-benchmark-polya-length-inference-from-direct-rna-sequencing/reviews#threadId=55398.The original preprint linked to the Research Hub review is here: https://doi.org/10.1101/2024.10.25.620206.

Level of Interest

Please indicate how interesting you found the manuscript: Choose an item.

Quality of Written English

Please indicate the quality of language in the manuscript: Choose an item.

Declaration of Competing Interests

Please complete a declaration of competing interests, considering the following questions:

- Have you in the past five years received reimbursements, fees, funding, or salary from an organisation that may in any way gain or lose financially from the publication of this manuscript, either now or in the future?
- Do you hold any stocks or shares in an organisation that may in any way gain or lose financially from the publication of this manuscript, either now or in the future?
- Do you hold or are you currently applying for any patents relating to the content of the manuscript?
- Have you received reimbursements, fees, funding, or salary from an organization that holds or has applied for patents relating to the content of the manuscript?
- Do you have any other financial competing interests?
- Do you have any non-financial competing interests in relation to this paper?

If you can answer no to all of the above, write 'I declare that I have no competing interests' below. If your reply is yes to any, please give details below.

I declare that I have no competing interests.

I agree to the open peer review policy of the journal. I understand that my name will be included on my report to the authors and, if the manuscript is accepted for publication, my named report including any attachments I upload will be posted on the website along with the authors' responses. I agree for my report to be made available under an Open Access Creative Commons CC-BY license (http://creativecommons.org/licenses/by/4.0/). I understand that any comments which I do not wish to be included in my named report can be included as confidential comments to the editors, which will not be published.

Choose an item.

To further support our reviewers, we have joined with Publons, where you can gain additional credit to further highlight your hard work (see: https://publons.com/journal/530/gigascience). On publication of this paper, your review will be automatically added to Publons, you can then choose whether or not to claim your Publons credit. I understand this statement.

Yes Choose an item.
